# Supplementary material for: Living fast in the Triassic: New data on life history in Lystrosaurus (Therapsida: Dicynodontia) from northeastern Pangea
Source: PLoS One. 2021 Nov 5;16(11):e0259369. doi: 10.1371/journal.pone.0259369 (PMC8570511; doi:10.1371/journal.pone.0259369)
Supplement: S1 File — (PDF) [file pone.0259369.s001.pdf]

Supplemental File for

Life history in *Lystrosaurus* from northeastern Pangea

Zoe T. Kulik, Jacqueline K. Lungmus, Kenneth D. Angielczyk, and Christian A. Sidor

Corresponding author
Email: zkulik@uw.edu

This file includes:
Figs S1 to S6
Table S1 and S2

Other supplementary materials for this manuscript include the following:

S2 Dataset (separate file):
- Microsoft excel document with 2 associated sheets
- Sheet 1 – cranial measurement data
- Sheet 2 – femoral length measurement data

Full-size, composite photographs of osteological thin sections are available on MorphoBank: **http://morphobank.org/permalink/?P4023**

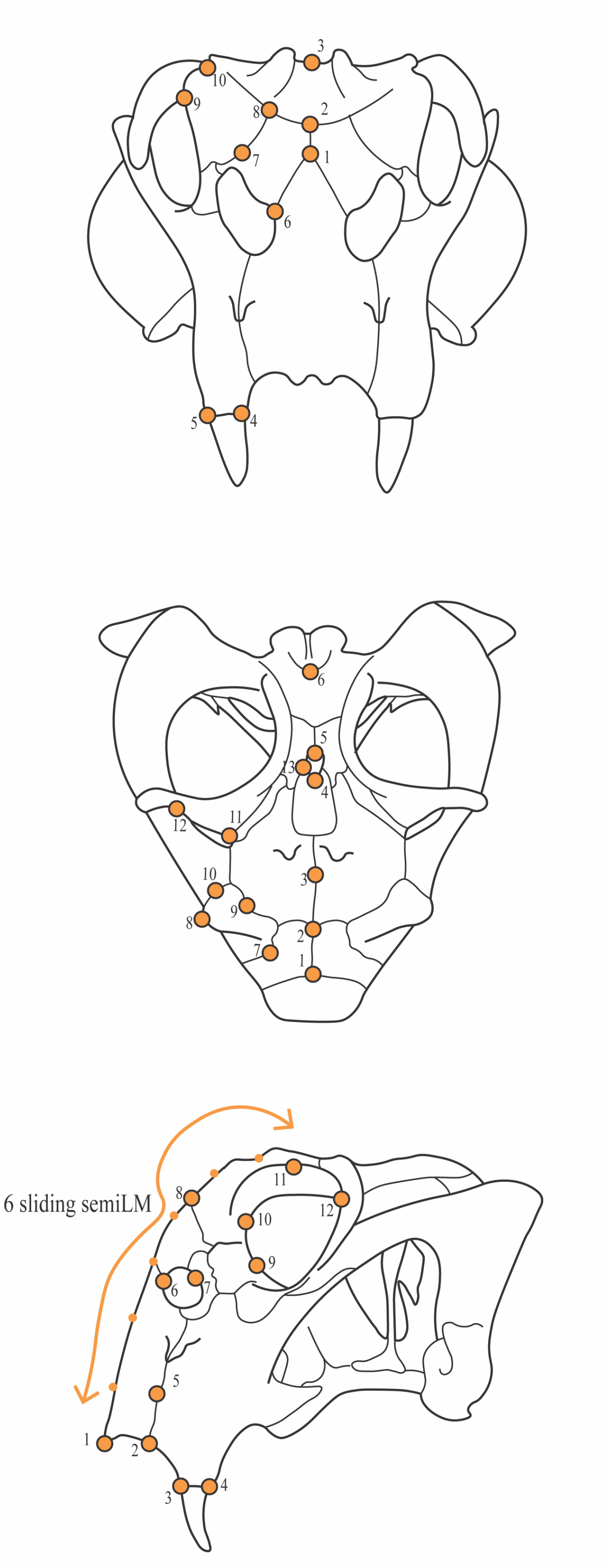

Fig. S1. Landmark selection for geometric morphometric analysis. Orange dots on the *Lystrosaurus* specimens show placement of landmarks used in the 2D geometric morphometric analysis in anterior (top), dorsal (middle), and lateral (bottom) views.

Table S1
Landmark descriptions:

Anterior:
1– premaxilla-nasal triple junction
2 – nasals-frontals quadruple junction
3 – intersection of frontals with transverse frontal ridge
4 – intersection of premaxilla-maxilla suture with oral margin
5 – ventrolateral corner of maxillary process
6 – anteromedial corner of naris
7 – ventral extreme of prefrontal
8 – junction of the prefrontal, nasal and frontal
9 – lateralmost point of prefrontal
10 – intersection of prefrontal-frontal suture with orbital rim

Dorsal:
1 – nasals-premaxilla triple junction
2 – nasals-frontals quadruple junction
3 – middle of sagittal frontal suture
4 – anterior extreme of the pineal foramen
5 – posterior extreme of the pineal foramen
6 – parietals-supraocciptal triple junction
7 – nasal-prefrontal-frontal triple junction
8 – lateral angle of prefrontal
9 – middle of prefrontal-frontal suture
10 – intersection of prefrontal-frontal suture with orbital rim
11 – of frontal-postfrontal suture with orbital rim
12 – lateralmost extent of the postorbital bar

Lateral:
1 – anterior tip of premaxilla
2 – premaxilla-maxilla sutural contact with oral margin
3 – anteroventral-most point of maxillary process
4 – lateroventral-most point of maxillary process
5 – dorsoventral midpoint of premaxilla-maxilla suture
6 – anteriormost point of naris
7 – posterodorsal corner of naris
8 – anteriormost point of the prefrontal
9 – anteroventral corner of orbit
10 – anteriormost point of orbit
11 – dorsalmost point of interior margin of orbit
12 – posteriormost point of orbit

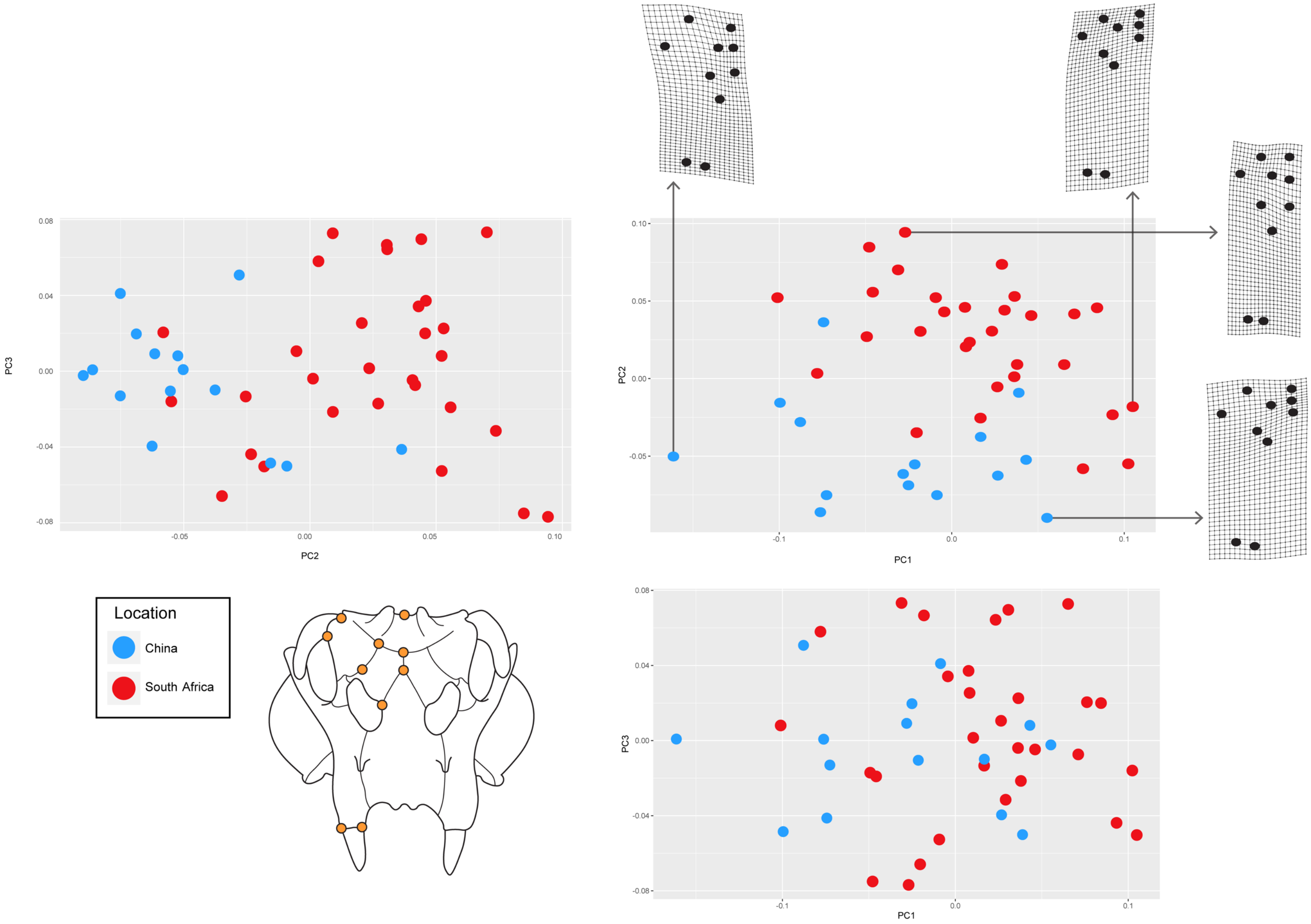

Fig. S2. Principal components plots (PC1-PC3) for the anterior perspective. Skull drawing of *Lystrosaurus* on the lower left correspond to the orientation for this analysis. Orange dots on the specimen show placement of landmarks used in the 2D geometric morphometric analysis. Blue dots represent specimens from China and red dots represent specimens from South Africa. Arrows point to the warp grids showing corresponding deformation along each principal component axis.

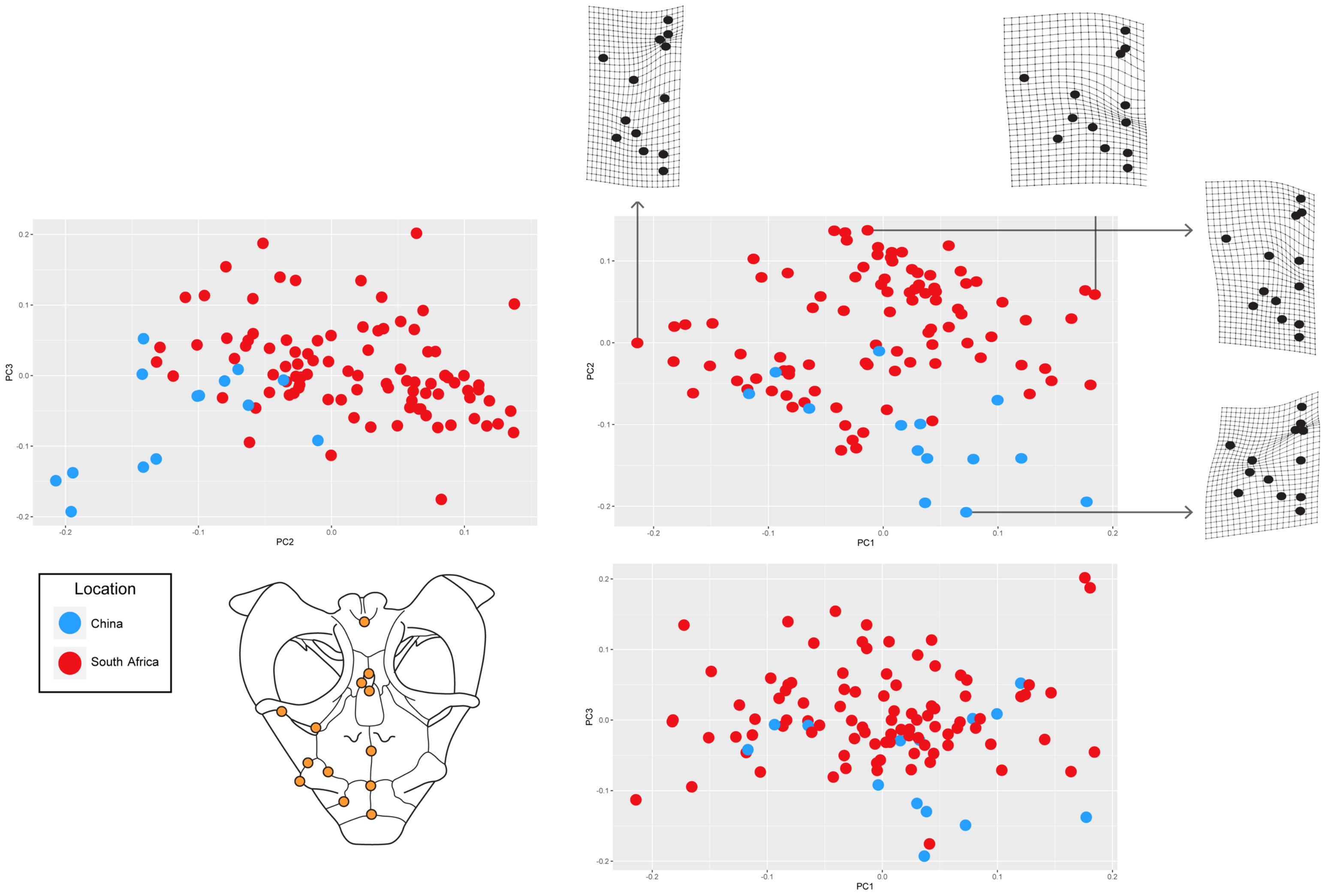

Fig. S3. Principal components plots (PC1-PC3) for the dorsal perspective. Skull drawing of *Lystrosaurus* on the lower left correspond to the orientation for this analysis. Orange dots on the specimen show placement of landmarks used in the 2D geometric morphometric analysis. Blue dots represent specimens from China and red dots represent specimens from South Africa. Arrows point to the warp grids showing corresponding deformation along each principal component axis.

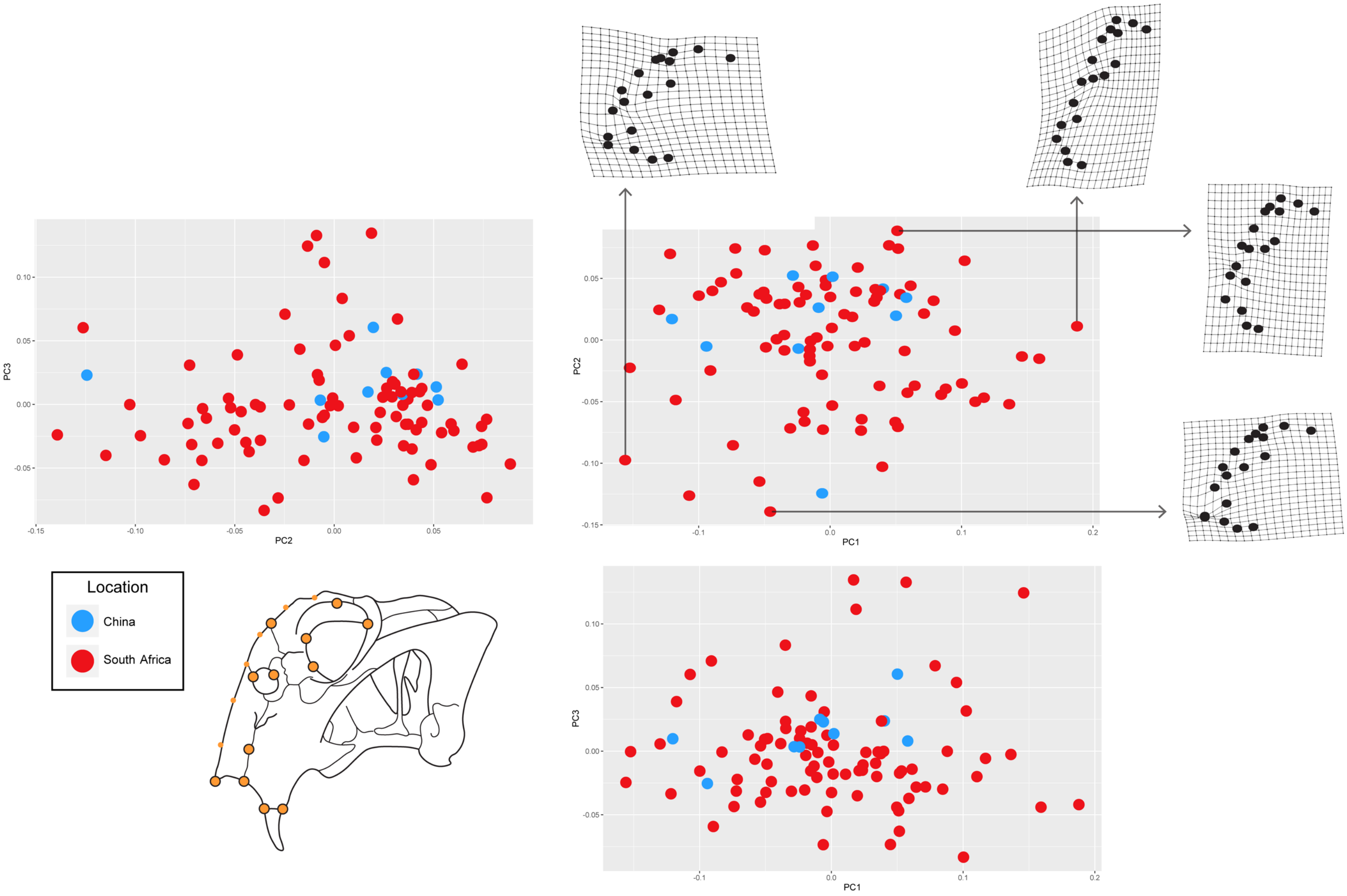

Fig. S4. Principal components plots (PC1-PC3) for the lateral perspective. Skull drawing of *Lystrosaurus* on the lower left correspond to the orientation for this analysis. Orange dots on the specimen show placement of landmarks used in the 2D geometric morphometric analysis. Blue dots represent specimens from China and red dots represent specimens from South Africa. Arrows point to the warp grids showing corresponding deformation along each principal component axis.

Table S2. Cross-sectional area and diameter measurements of femoral thin sections that were used to calculate cortical thickness (*K*), the proportional area of the medullary region relative to the total area of the bone cross-section. We report proportional cortical thickness based on area (K_A) and diameter (K_D). Measurements are shown below and were taken on the smallest femur, IVPP V 27124-7 (Fig S5) and largest femur, IVPP V 27127 (Fig S6) in FIJI.

Specimen Number	Cross-sectional area (mm^2)	Cross-sectional Major axis (mm)	Cross-sectional Minor axis (mm)	Medullary cavity area (mm^2)	Medullary Major axis (mm)	Medullary Minor axis (mm)	K_A	K_D
IVPP V 27124-7	266.75	21.91	15.5	105.64	13.78	9.76	0.40	0.63
IVPP V 27127	654.62	34.52	24.15	233.46	22.12	13.44	0.36	0.64

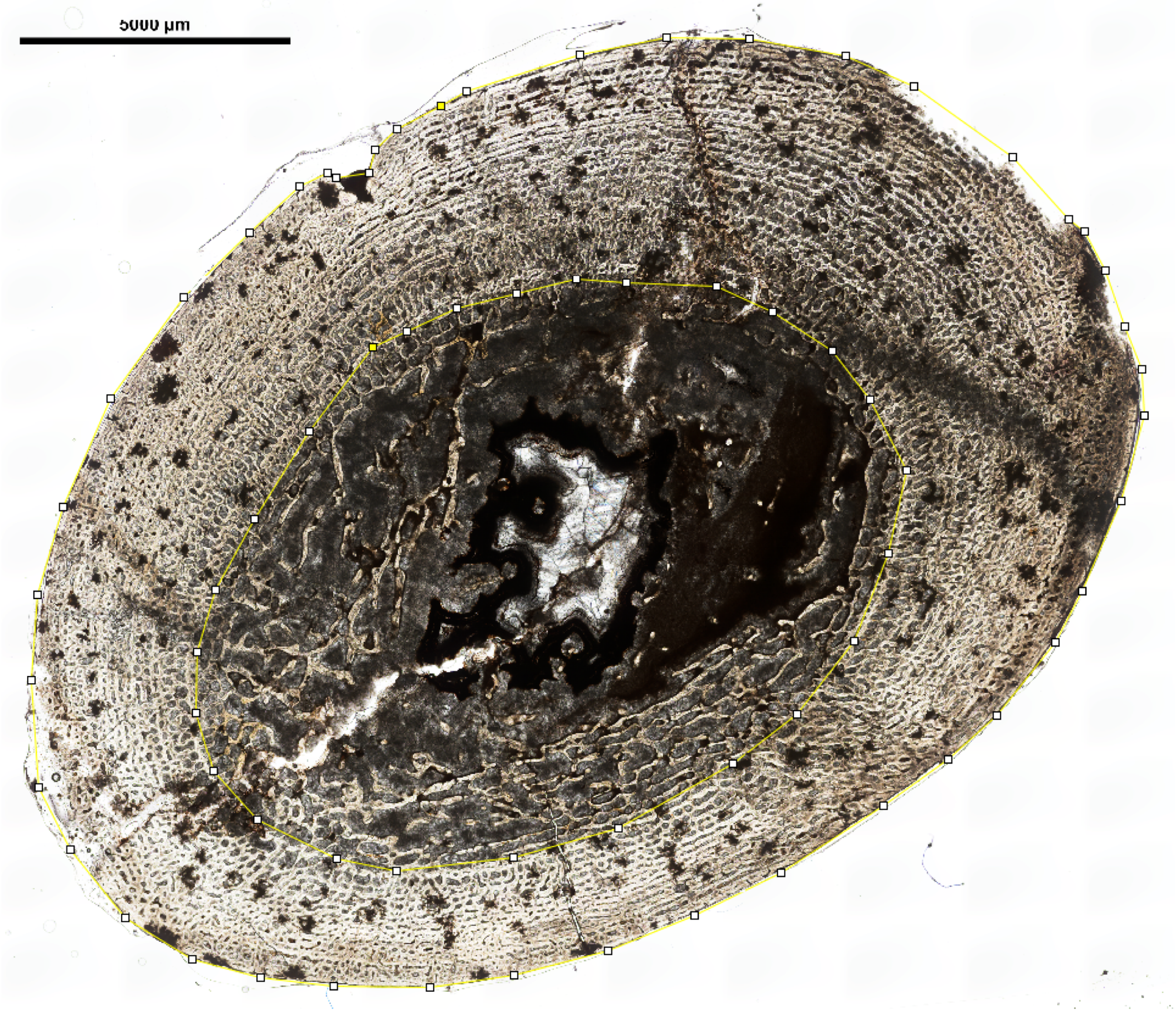

Fig. S5. Cortical thickness measurements of specimen IVPP V 27124-7. Outer and inner yellow polygons trace the region of compact cortical bone. Measurements were taken using FIJI. Scale bar = 5000 μm.

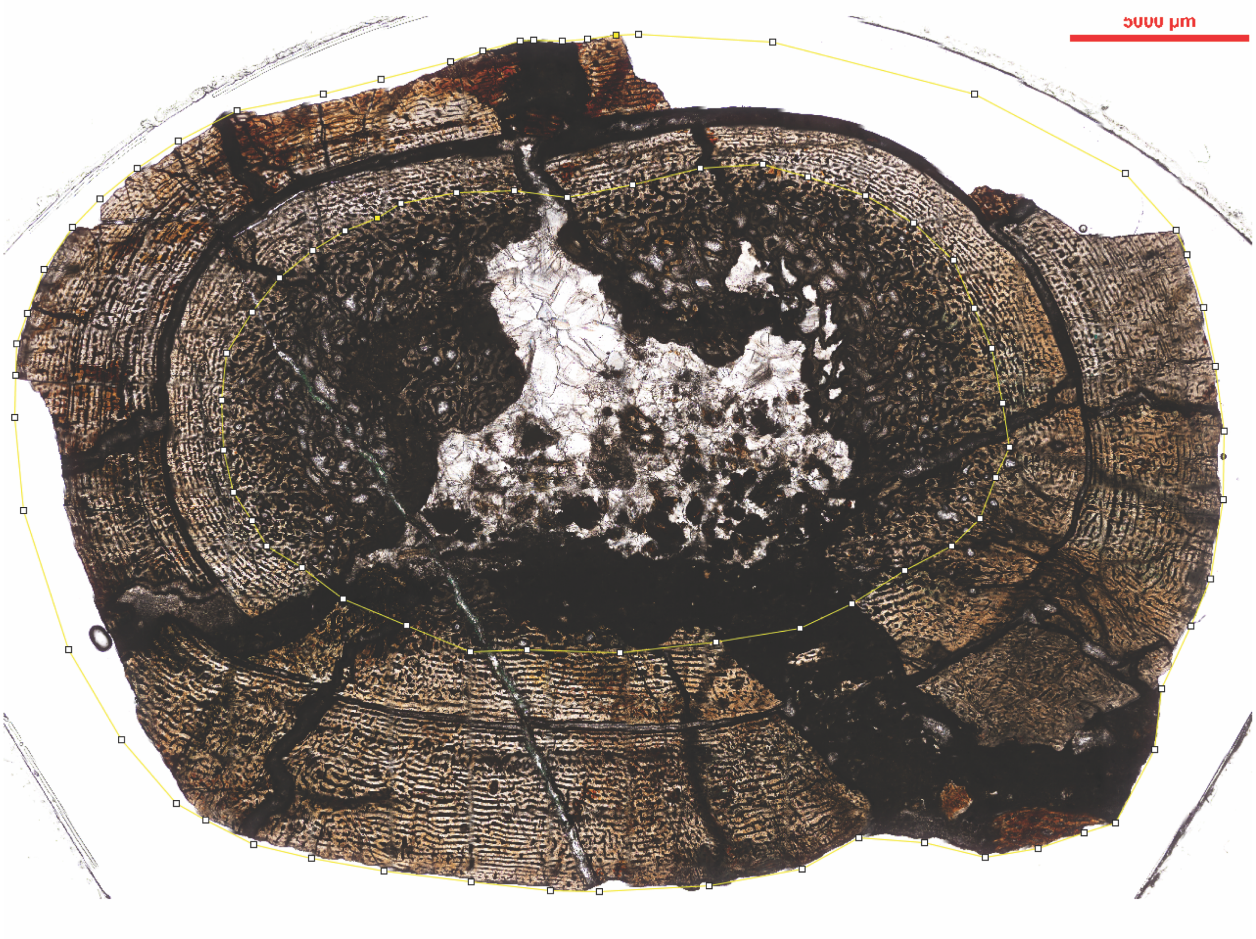

Fig. S6. Cortical thickness measurements of specimen IVPP V 27127. Outer and inner yellow polygons trace the estimated region of compact cortical bone. Measurements were taken using FIJI. Scale bar = 5000 µm.
