# Supplementary material for: Living fast in the Triassic: New data on life history in Lystrosaurus (Therapsida: Dicynodontia) from northeastern Pangea
Source: PLoS One. 2021 Nov 5;16(11):e0259369. doi: 10.1371/journal.pone.0259369 (PMC8570511; doi:10.1371/journal.pone.0259369)
Supplement: Attachment — Submitted filename: PONE-D-21-20672_reviewer comments.pdf [file pone.0259369.s003.pdf]

PLOS ONE

Living Fast in the Triassic: New data on life history in <i>Lystrosaurus</i> (Therapsida: Dicynodontia) from northeastern Pangea

--Manuscript Draft--

**Manuscript Number:**	PONE-D-21-20672
**Article Type:**	Research Article
**Full Title:**	Living Fast in the Triassic: New data on life history in <i>Lystrosaurus</i> (Therapsida: Dicynodontia) from northeastern Pangea
**Short Title:**	Life history in <i>Lystrosaurus</i> from northeastern Pangea
**Corresponding Author:**	Zoe T. Kulik<br>University of Washington Seattle Campus: University of Washington<br>Seattle, WA UNITED STATES
**Keywords:**	Lystrosaurus; bone histology; life history; lines of arrested growth; Triassic; China; Jiucaiyuan Formation
**Abstract:**	<i>Lystrosaurus</i> was one of the few tetrapod genera to survive the Permo-Triassic mass extinction, the most profound biotic crisis in Earth's history. The wide paleolatitudinal range and incredible abundance of <i>Lystrosaurus</i> during the Early Triassic provides a unique opportunity to investigate changes in growth dynamics and longevity following the mass extinction, yet most studies have focused only on species that lived in the southern hemisphere. Here, we present the long bone histology from twenty <i>Lystrosaurus</i> skeletal elements spanning a range of sizes that were collected in the Jiucaiyuan Formation of northwestern China. In addition, we compare the average body size of northern and southern Pangean Triassic-aged species, and conduct cranial geometric morphometric analyses of southern and northern specimens to begin investigating whether populations from China are likely to be distinct from South African populations. We demonstrate that <i>Lystrosaurus</i> from China have larger average body sizes than their southern Pangean relatives and that the Chinese and South African populations have distinct cranial morphologies. Our osteohistological examination revealed sustained, rapid osteogenesis punctuated by growth marks, in some, but not all immature individuals from China. We find that the osteohistology of Chinese <i>Lystrosaurus</i> shares a similar growth pattern to South African species that show sustained growth until death. However, bone growth arrests more frequently in the Chinese sample. Nevertheless, each of the long bones sampled here do not indicate that maximum or asymptotic size was reached, suggesting that the maximum size of <i>Lystrosaurus</i> from the Jiucaiyuan Formation remains unknown.
**Order of Authors:**	Zoe T. Kulik
	Jacqueline K. Lungmus
	Kenneth D. Angielczyk
	Christian A. Sidor
**Additional Information:**	
**Question**	**Response**
**Financial Disclosure**<br><br>Enter a financial disclosure statement that describes the sources of funding for the work included in this submission. Review the submission guidelines for detailed requirements. View published research articles from *PLOS ONE* for specific examples.	This research was supported by National Science Foundation grants EAR 1714829 (to KDA) and EAR 1713787 (to CAS), and UW Biology Department Iuvo and Walker awards (to ZTK). The funders had no role in study design, data collection and analysis, decision to publish, or preparation of the manuscript.

This statement is required for submission and **will appear in the published article** if the submission is accepted. Please make sure it is accurate.<br><br>**Unfunded studies**<br>Enter: *The author(s) received no specific funding for this work.*<br><br>**Funded studies**<br>Enter a statement with the following details:<br>• Initials of the authors who received each award<br>• Grant numbers awarded to each author<br>• The full name of each funder<br>• URL of each funder website<br>• Did the sponsors or funders play any role in the study design, data collection and analysis, decision to publish, or preparation of the manuscript?<br>• **NO** - Include this sentence at the end of your statement: *The funders had no role in study design, data collection and analysis, decision to publish, or preparation of the manuscript.*<br>• **YES** - Specify the role(s) played.<br><br>* typeset	
**Competing Interests**<br><br>Use the instructions below to enter a competing interest statement for this submission. On behalf of all authors, disclose any competing interests that could be perceived to bias this work—acknowledging all financial support and any other relevant financial or non-financial competing interests.<br><br>This statement is required for submission and **will appear in the published article** if the submission is accepted. Please make sure it is accurate and that any funding sources listed in your Funding Information later in the submission form are also declared in your Financial Disclosure statement.<br><br>View published research articles from	The authors have declared that no competing interests exist.

Powered by Editorial Manager® and ProduXion Manager® from Aries Systems Corporation

PLOS ONE for specific examples.<br><br>**NO authors have competing interests**<br><br>Enter: *The authors have declared that no competing interests exist.*<br><br>**Authors with competing interests**<br><br>Enter competing interest details beginning with this statement:<br><br>*I have read the journal's policy and the authors of this manuscript have the following competing interests: [insert competing interests here]*<br><br>* typeset	
**Ethics Statement**<br><br>Enter an ethics statement for this submission. This statement is required if the study involved:<br><br>• Human participants<br>• Human specimens or tissue<br>• Vertebrate animals or cephalopods<br>• Vertebrate embryos or tissues<br>• Field research<br><br>Write "N/A" if the submission does not require an ethics statement.<br><br>General guidance is provided below. Consult the submission guidelines for detailed instructions. **Make sure that all information entered here is included in the Methods section of the manuscript.**	N/A

**Format for specific study types**<br><br>**Human Subject Research (involving human participants and/or tissue)**<br>• Give the name of the institutional review board or ethics committee that approved the study<br>• Include the approval number and/or a statement indicating approval of this research<br>• Indicate the form of consent obtained (written/oral) or the reason that consent was not obtained (e.g. the data were analyzed anonymously)<br><br>**Animal Research (involving vertebrate animals, embryos or tissues)**<br>• Provide the name of the Institutional Animal Care and Use Committee (IACUC) or other relevant ethics board that reviewed the study protocol, and indicate whether they approved this research or granted a formal waiver of ethical approval<br>• Include an approval number if one was obtained<br>• If the study involved *non-human primates*, add *additional details* about animal welfare and steps taken to ameliorate suffering<br>• If anesthesia, euthanasia, or any kind of animal sacrifice is part of the study, include briefly which substances and/or methods were applied<br><br>**Field Research**<br><br>Include the following details if this study involves the collection of plant, animal, or other materials from a natural setting:<br>• Field permit number<br>• Name of the institution or relevant body that granted permission	
**Data Availability**<br><br>Authors are required to make all data underlying the findings described fully available, without restriction, and from the time of publication. PLOS allows rare exceptions to address legal and ethical concerns. See the PLOS Data Policy and FAQ for detailed information.	Yes - all data are fully available without restriction

A Data Availability Statement describing where the data can be found is required at submission. Your answers to this question constitute the Data Availability Statement and **will be published in the article**, if accepted.<br><br>**Important:** Stating 'data available on request from the author' is not sufficient. If your data are only available upon request, select 'No' for the first question and explain your exceptional situation in the text box.<br><br>Do the authors confirm that all data underlying the findings described in their manuscript are fully available without restriction?	
**Describe where the data may be found in full sentences. If you are copying our sample text, replace any instances of XXX with the appropriate details.**<br><br>• If the data are **held or will be held in a public repository**, include URLs, accession numbers or DOIs. If this information will only be available after acceptance, indicate this by ticking the box below. For example: *All XXX files are available from the XXX database (accession number(s) XXX, XXX.).*<br>• If the data are all contained **within the manuscript and/or Supporting Information files**, enter the following: *All relevant data are within the manuscript and its Supporting Information files.*<br>• If neither of these applies but you are able to provide **details of access elsewhere**, with or without limitations, please do so. For example:<br><br>*Data cannot be shared publicly because of [XXX]. Data are available from the XXX Institutional Data Access / Ethics Committee (contact via XXX) for researchers who meet the criteria for access to confidential data.*<br><br>*The data underlying the results presented in the study are available from (include the name of the third party*	All relevant data are within the manuscript and its Supporting Information files including links to composite thin section images available on Morphobank.

and contact information or URL).<br>• This text is appropriate if the data are owned by a third party and authors do not have permission to share the data.<br><br>* typeset	
Additional data availability information:	Tick here if the URLs/accession numbers/DOIs will be available only after acceptance of the manuscript for publication so that we can ensure their inclusion before publication.

Powered by Editorial Manager® and ProduXion Manager® from Aries Systems Corporation

Manuscript Click here to access/download;Manuscript;Kulik_etal_LivingFastTriassic.docx

Living Fast in the Triassic: New data on life history in *Lystrosaurus* (Therapsida: Dicynodontia) from northeastern Pangea

Short Title: Life history in *Lystrosaurus* from northeastern Pangea

Zoe T. Kulik, Jacqueline K. Lungmus, Kenneth D. Angielczyk, and Christian A. Sidor

Zoe T. Kulik [zkulik@uw.edu], Department of Biology and Burke Museum, University of Washington, Seattle, WA 98195

Jacqueline K. Lungmus [lungmusj@si.edu] Smithsonian National Museum of Natural History Washington, D.C. 20560

Kenneth D. Angielczyk [kangielczyk@fieldmuseum.org], Negaunee Integrative Research Center, Field Museum of Natural History, Chicago, IL 60605

Christian A. Sidor [casidor@uw.edu], Department of Biology and Burke Museum, University of Washington, Seattle, WA 98195

Abstract

Lystrosaurus was one of the few tetrapod genera to survive the Permo-Triassic mass extinction, the most profound biotic crisis in Earth's history. The wide paleolatitudinal range and incredible abundance of *Lystrosaurus* during the Early Triassic provides a unique opportunity to investigate changes in growth dynamics and longevity following the mass extinction, yet most studies have focused only on species that lived in the southern hemisphere. Here, we present the long bone histology from twenty *Lystrosaurus* skeletal elements spanning a range of sizes that were collected in the Jiucaiyuan Formation of northwestern China. In addition, we compare the average body size of northern and southern Pangean Triassic-aged species, and conduct cranial geometric morphometric analyses of southern and northern specimens to begin investigating whether populations from China are likely to be distinct from South African populations. We demonstrate that *Lystrosaurus* from China have larger average body sizes than their southern Pangean relatives and that the Chinese and South African populations have distinct cranial morphologies. Our osteohistological examination revealed sustained, rapid osteogenesis punctuated by growth marks, in some, but not all immature individuals from China. We find that the osteohistology of Chinese *Lystrosaurus* shares a similar growth pattern to South African species that show sustained growth until death. However, bone growth arrests more frequently in the Chinese sample. Nevertheless, each of the long bones sampled here do not indicate that maximum or asymptotic size was reached, suggesting that the maximum size of *Lystrosaurus* from the Jiucaiyuan Formation remains unknown.

Introduction

The Permo-Triassic mass extinction was the most devastating biotic crisis in Earth's history and caused communities to collapse in both the terrestrial and marine realms [1-3]. Fluctuating climates accompanied by an overall increase in global temperatures are hypothesized to have forced 70% of terrestrial vertebrate families to extinction [4-8], although plant extinctions were notably dampened [9-11]. Recovery after the Permo-Triassic extinction has been a topic of intense research, but most studies in the terrestrial realm focus on taxonomic diversity and abundance [12-14], or changes in community structure and dynamics following the extinction [6, 3, 15-18]. Few studies have examined how the extremely changed environment of the earliest Triassic impacted growth rate, life history, and longevity of surviving taxa [19-22]. Importantly, most of the studies noted above rely heavily—if not exclusively—on data from the southern hemisphere, in particular the Karoo Basin of South Africa.

Lystrosaurus, a stoutly built dicynodont therapsid that ranged in maximum skull size from approximately 16–39 cm (*L. murrayi* and *L. maccaigi* from South Africa, respectively), is one of the hallmark survivors of the Permo-Triassic extinction due to its remarkable abundance in southern Pangean deposits [18, 23, 24]. The success of *Lystrosaurus* has been variously attributed to its generalist diet of tough plant material (e.g., [25, 26]), broad habitat tolerances (e.g., [27]), a burrowing lifestyle (e.g., [28]), and a developmentally plastic growth strategy that allowed it the ability to weather extreme ecosystem instability (e.g., [29, 20, 22, 30]). These hypothesized advantages/exaptations for a post-extinction environment have been studied almost exclusively using specimens from South Africa, but *Lystrosaurus* is known from across Pangea, including Permo-Triassic deposits in Antarctica, Mongolia, Russia, China, India, and possibly

Mozambique (e.g., [31-38]). The wide paleolatitudinal range of *Lystrosaurus* offers a rare opportunity to investigate differences in lifespan, survivorship, and body size in southern and northern Pangean populations that experienced markedly different environmental conditions in the wake of the Permo-Triassic extinction (e.g., [22, 39]).

Osteohistological Perspectives on Life History

Bone histology provides a wealth of information about an extinct animal's growth and life history. Mineralized bone matrix, vascular canal spaces, and cellular spaces persist in fossilized bone, making it possible to determine the relative rate of bone deposition as well as the maturity of extinct organisms [40]. One particularly useful yet controversial histological feature in determining aspects of an extinct animal's growth are hyper-mineralized lines of arrested growth (LAGs) which appear as cement lines in the cortical bone of most vertebrates when bone deposition periodically stops in seasonal or annual cycles [41-46]. In addition to extrinsically (i.e., environmentally or seasonally) induced LAGs, growth can also arrest during periods of intense stress, such as birth, and during modulations to metabolic activity such as torpor [47, 48]. The periodicity of LAGs (i.e., the extent to which they are regularly spaced throughout the cortex of large, presumably mature individuals) helps to determine whether they represent extrinsic or intrinsic events.

Botha [22] recently conducted a detailed examination of growth and life history patterns in four species of *Lystrosaurus* across the Permian-Triassic boundary using fossils from South Africa. Using a combination of bone histology and body size data, Botha [22] found that Triassic *Lystrosaurus* individuals grew rapidly and had increased mortality at small sizes, as evidenced by the overwhelming abundance of small skulls known from Triassic rocks in the Karoo Basin.

This post-extinction change in life expectancy is also inferred from the scarcity of lines of arrested growth (LAGs) in the rapidly deposited cortical bone of Triassic specimens. In comparison, Permian individuals had larger average skull sizes and had numerous ~~LAGs~~ spaced throughout the cortex, suggesting prolonged periods of growth spanning multiple seasons or years [22]. Similar patterns were previously found across a wider taxonomic range of South African therapsid species by Botha-Brink et al. [20] and in lydekkerinid stereospondyls [49, 50]. The predominance of small *Lystrosaurus* (and other tetrapods) with rapidly deposited bone tissue suggests an overall shift in life history strategy across the Permian-Triassic boundary to an early onset of reproductive maturity [20, 49, 50].

Histological data for *Lystrosaurus* outside of the Karoo Basin of South Africa are more limited, but critical to understanding the relationship between latitude (and by extension climate) and life history. Bones of *Lystrosaurus* from the Early Triassic of India lack LAGs, and have small skulls, consistent with the pattern seen in South Africa [36, 51]. A preliminary report of the bone histology from *Lystrosaurus* from the Turpan-Hami Basin of China suggested that subadult individuals record multiple LAGs [39]. However, in the absence of comparative body size data, it remains unclear if multiple LAGs in the Chinese specimens indicate prolonged periods of growth (i.e., multiple cycles or seasons of growth) or environmental instability (e.g., disruptions to metabolic rate as a result of resource limitations). Here, we analyze bone histology from a size range of *Lystrosaurus* postcrania from the Jiucaiyuan Formation of northwestern China (Fig 1), as well as body size data across Triassic-aged *Lystrosaurus* assemblages, to investigate whether *Lystrosaurus* from northern Pangea had differing life histories than its southern Pangean relatives.

Figure 1. Size distribution of *Lystrosaurus* sp. postcrania from the Jiucaiyuan Formation included in the histologic analysis. Twenty skeletal elements, with gray bars indicating associations of individuals, span three size classes inferred from femoral length: Size Class II spans 47–63% maximum femoral size, Size Class III includes specimens that are ~ 70% maximum size, and Size Class III includes the largest partial femur. Scale bar = 5 cm.

Materials and Methods

Geometric Morphometric Analysis of Skull Shape

An important question to consider when comparing the bone histology of *Lystrosaurus* specimens from China and other regions is whether the same species occur in the different geographic areas. Although the initial material of *Lystrosaurus* collected in Xinjiang was referred to the South African species *L. murrayi* [52], a total of seven endemic *Lystrosaurus* species have also been described [53-57]. Colbert [32], (also see [58-60]), considered some Chinese specimens to represent *L. murrayi* and *L. curvatus*, and others to represent endemic species. More recent authors have been split over whether *Lystrosaurus* specimens from Xinjiang represent exclusively endemic species or a mixture of endemic and more cosmopolitan species, as well as the number of valid endemic species (e.g., [34, 39, 61-64]).

A comprehensive taxonomic revision of *Lystrosaurus* from Xinjiang is beyond the scope of this paper. However, to gain insight into whether Chinese *Lystrosaurus* specimens are likely to be distinct from South African specimens, we conducted a preliminary two-dimensional geometric morphometric analysis of skull shape. The skulls were analyzed in three perspectives - anterior, dorsal, and lateral – following the methodology of [65]; also see [64]. The anterior shape analysis included 45 individual specimens, 15 from China and the remaining 30 from

South Africa. The dorsal shape analysis included 107 specimens, 14 from China and 93 from South Africa. Lastly, the lateral analysis included 95 specimens, 9 from China and 86 from South Africa. Landmarks were collected using the tpsDig2ws software [66]. In the case of the anterior and dorsal views, landmarks were digitized on only the left half of the skull. The lateral view digitization included a combination of Type I and Type II landmarks (12 total) with a single curve of six semilandmarks along the outer edge of the snout between landmarks 1 and 11 (Fig 2). The anterior and dorsal views did not contain any semilandmarks, and have 10 and 13 landmarks, respectively. Because taphonomic deformation can complicate the interpretation of shape variation at low taxonomic levels in dicynodonts [67], data collection focused on specimens that appeared to show relatively little evidence of distortion. See the supplementary files for additional details of the morphometric analysis.

The coordinate data were processed and analyzed further with the Geomorph R package [68], where they underwent a generalized Procrustes superimposition. Principal components analyses were used to visualize major patterns of shape variation, and a Procrustes ANOVA was run on each dataset to assess the statistical relationship between shape and country of origin using the method of [69] and the Geomorph package in R. A canonical variates analysis was run using the CVA function of the Morpho package [70] in R to further explore the association between shape and geographic location, as well as the accuracy with which specimens could be classified using skull shape (see *Results*).

Figure 2. Results of the morphometric analysis comparing specimens of *Lystrosaurus* from China and South Africa. Principal components plots and histogram of canonical variates analysis results for the three perspectives (Anterior – A; Dorsal – B; Lateral – C). Skull drawings on the

right correspond to the orientation for each analysis. Orange dots on the specimens show placement of landmarks used in the 2D geometric morphometric analysis (see supplemental files for more details). Blue dots represent specimens from China and red dots represent specimens from South Africa. Arrows point to the warp grids showing corresponding deformation along each principal component axis. Histograms show frequency of a recovered canonical variate value and show the distinct differences between Chinese and South African specimens, with overlap only occurring in a few specimens in the lateral orientation.

Histological Sample

Twenty skeletal elements (humerus, femur, tibia, radius, ulna, fibula, and ribs; Table 1; Fig 1) of *Lystrosaurus* sp. were selected for consumptive analysis from the collections of the Institute of Vertebrate Paleontology and Paleoanthropology (IVPP). All material was recovered from approximately 30–50 meters above the base of the Jiucaiyuan Formation from the South Taodonggou area, Turpan-Hami Basin, Xinjiang Uygur Autonomous Region, China. The Jiucaiyuan Formation overlies the Guodikeng Formation lithologically (=upper Wutonggou low-order cycle), and its contact is characterized by a facies change from lacustrine to fluvial deposits [71, 72]. This facies change is interpreted as the boundary between the Permian and Triassic [72] and recent work suggests that the Jiucaiyuan Formation is late Induan-early Olenekian in age [72]. As noted above, the lack of clarity regarding Chinese *Lystrosaurus* taxonomy means that we were unable to assign species-level identifications. However, the majority of our histologic samples are associations of four individuals. Skeletal elements are differentiated by specimen numbers followed by a letter, in the case of associated individuals (e.g., IVPP V#-a, -b, etc.) The

remaining eight specimens are disarticulated elements collected from the same locality and are assigned to one specimen number IVPP V27124, differentiated by IVPP V27124-1, -2, etc. In addition to the histologic sample, 50 femora were measured from Early Triassic specimens compiled from the literature [36, 22] and from personal observations to contextualize the range of sizes in this monodominant vertebrate assemblage (S2 Supplementary Dataset). Additional body size proxies, including basal skull length (S2 Supplementary Dataset), were measured from *Lystrosaurus* specimens housed in collections at the National Museum (NMQR), the Evolutionary Studies Institute (ESI), the Iziko South African Museum (SAM), the Field Museum of Natural History (FMNH), the American Museum of Natural history (AMNH), and the Burke Museum of Natural History and Culture (UWBM). Cranial and postcranial measurements were made using Neiko digital calipers.

TABLE 1. Postcranial element length and midshaft diameter for the twenty skeletal elements of *Lystrosaurus* sp. from the Jiucaiyuan Formation that were histologically sampled. Asterisk indicates fragmentary or incomplete specimens with accompanying estimates based on complete specimens in this dataset.

	Spec. No.	Preserved Length (mm)	Estimated Length (mm)	Midshaft Diameter (mm)	Growth Marks	Size Class
**Femur**						
	IVPP V27124-7	122.38		19.23	0	II
	IVPP V27124-6	62.74*	129	16.22	0	II
	IVPP V27126a	62*	162		0	II

	IVPP V27125a	182		13.4	1	III
	IVPP V26548a	192		14.09	2	III
	IVPP V27127	240*	255	16.57	0	IV
**Tibia**						
	IVPP V27126b	92			0	II
	IVPP V27124-4	56.62*	94	15.86	0	II
	IVPP V27124-3	53.26*	98	15.71	0	II
	IVPP V26548b	148		10.47	2	III
	IVPP V27125b	148*	212	10.36	2	III
**Fibula**						
	IVPP V27124-1	46.37*	86	10.12	0	II
	IVPP V27126c	86		58.9	0	II
	IVPP V26548c	140		7.334	2	III
	IVPP V27125c	140		15.84	2-3	III
**Humerus**						
	IVPP V27124-8	151.77		23.96	1	III
**Radius**						
	IVPP V27126d	62			0	II

**Ulna**						
	IVPP V27126e	75			0	II
	IVPP V27124-2	47.32*	93	17.52	0	III
**Ribs**						
	IVPP V27124-5	58.63*	N/A	48.76 - 56.33	EFS	?

Thin Section Preparation

Thin sections were made following standard osteohistological techniques [73]. Eight of the specimens were sectioned at the University of Washington (UW); the remaining twelve were sectioned at the Institute of Vertebrate Paleontology and Paleoanthropology (IVPP). At the UW, mid-diaphyses were embedded in Epothin Epoxy/Resin 2, sectioned to a thickness of approximately 2 mm on a high-precision diamond-edged saw (Isomet 1000) and glued to glass slides using Devcon 2-Ton epoxy. Slides were ground on a Metaserv 3000 lapidary plate until the specimen was 80 µm thick or until optical clarity was reached. At the IVPP, midshafts were cut and embedded in polyester resin, sectioned into blocks using an Exact 300CP band saw, and glued to glass slides using Technovit 4000 resin. Slides were ground to optical clarity using an automated lapidary plate (Exakt 400CS). High magnification and composite images were taken using a Nikon Eclipse LV100POL microscope under plain and cross-polarized light with a lambda filter. Composite images were processed using Nikon NIS-Elements AR (version 5.20.02) imaging software. High-resolution images are available at Morphobank under project number 4023.

Ontogenetic maturity was assessed through bone tissue microstructure, articular surface texture and morphology, and size compared to other specimens of *Lystrosaurus* from the Jiucaiyuan Formation. The largest femur, estimated at 255 mm, is of comparable length to the largest known femur (*L. declivis* SAM-PK-K8038) from the Triassic of South Africa, which is 202 mm long. Therefore, we follow the size classes established by Botha [22] for southern Pangean *Lystrosaurus*, but note that our sample lacks Size Class I, or elements less than 40% maximum known size. The remainder of the elements were split into three size categories for analysis (Table 1; Fig 1). Size Class II includes the smallest and presumably most immature specimens in the current sample, ranging in femoral lengths that are approximately 120–160 mm long, tibiae and fibulae that are less than 100 mm long, and associated forelimb elements, or 47–63% of the maximum femur length observed. All but the proximal tibia fragment (IVPP V27124-3) have poorly formed joint surfaces and show pitting indicative of epiphyseal cartilage attachment typical of immature individuals [74]. By comparison, the joint surfaces of elements in the larger size classes are more robustly built and more completely ossified. The relative degree of epiphyseal ossification combined with histological features caused us to deviate slightly from Botha's [22] size classes by grouping specimens that are 40-65% maximum known size as Size Class II, rather than her 60% MKS cutoff. Our reasoning for this slight change is that the bone tissue is consistently and characteristically immature (i.e., highly vascularized plexiform to radial tissue without LAGs) in all of the associated elements that are 63% MKS. However, the incomplete tibia (IVPP V27124-3, estimated length of 94 mm) that is estimated to be within Size Class II has a more developed and ossified joint surface compared to similarly sized tibiae (IVPP V27124-4, 94 mm in length) so it is likely that with more sampling, the split between Size Class II and III will be refined. Size Class III includes immature specimens that are approximately

70% maximum known size (again, based on femoral lengths from larger, complete, Jiucaiyuan specimens). Finally, Size Class IV includes a single femur with an estimated length of 255 mm (the specimen measures 240 mm and is missing the proximal shaft and head of the femur). To our knowledge, it is the largest femur of *Lystrosaurus* yet recovered from the Jiucaiyuan Formation, and is ~ 5 cm longer than the largest femur (discussed above) recovered from the Early Triassic of South Africa. Therefore, it presumably represents the most skeletally mature individual in the current sample. However, histological analysis will provide critical context for the relative maturity of this, and all other specimens sampled [75].

Results

Geometric Morphometric Analysis of Skull Shape

Anterior view: The first five principal components capture 81.41% of the total shape variance in anterior view (PC1 - 30.57%; PC2 - 22.03%; PC3 - 13.82%; PC4 - 8.71%; PC5 - 6.26%) (Fig 2A). Shape is a statistically significant predictor of geographic location for the anterior view dataset ($p = 0.001$), and the canonical variates analysis correctly assigned specimen location 100% of the time.

PC1 primarily describes relative proportions of the snout. The subnarial region of the snout and caniniform process are dorsoventrally shorter relative to the height of the nasals and the anterior orbital margin in specimens with low PC1 scores, and there is greater lateral projection of the prefrontals relative to the caniniform process (Fig 2A). In contrast, the subnarial region and the caniniform process are proportionally much deeper relative to the anterior orbital margin and nasals in specimens with high PC1 scores, and the prefrontals and caniniform process are more closely aligned along a vertical axis. PC2 also captures aspects of skull

widening and deepening, although in this case the differences are concentrated in the region of the nasals and prefrontals. Specifically, the prefrontal-nasal region is dorsoventrally shorter in specimens with low PC2 scores, and the dorsal and ventral extrema of the prefrontal are displaced laterally relative to the external naris and the lateral end of the nasofrontal suture. Specimens with high PC2 scores have dorsoventrally taller prefrontal-nasal regions, and the dorsal and ventral extrema of the prefrontal are more closely aligned with the external naris and the lateral edge of the nasofrontal suture. The Chinese and South African specimens are most clearly differentiated along PC2, with the Chinese specimens generally having lower PC2 scores.

Dorsal view: The first five principal components capture 79.75% of the shape variance in dorsal view (PC1 - 26.77%; PC2 - 23.57%; PC3 - 17.95%; PC4 - 6.18%; PC5 - 5.26%) (Fig 2B). Shape is a statistically significant predictor of geographic location for the dorsal view dataset ($p = 0.001$), and the canonical variates analysis correctly assigned specimen location 100% of the time.

Shape variation along PC1 is dominated by differences in the relative lengths of the interorbital region and the pineal foramen, and by the degree of lateral projection of the orbital rim. Specimens with low PC1 scores are characterized by an anteroposteriorly longer interorbital region and a shorter pineal foramen, and an orbital rim that does not project strongly laterally. The pineal foramen is relatively longer anteroposteriorly in specimens with high PC1 scores, and the anterior and posterior corners of the orbital rim (formed by the prefrontal and postorbital bar, respectively) project laterally away from the skull roof. PC2 also captures shape variation associated with relative proportions of the skull roof, as well as orbit shape. Specimens with low PC2 scores have an anteroposteriorly shorter pineal foramen and longer frontals, and the orbit is

anteroposteriorly shorter, with anterior and posterior corners that are relatively aligned. The pineal foramen and orbit are relatively longer in specimens with high PC2 scores, and the posterior corner of the orbit (formed by the postorbital bar) projects farther laterally than the anterior orbital margin (formed by the prefrontal). As with the anterior view dataset, the Chinese and South African specimens are most clearly differentiated along PC2, with the Chinese specimens generally having lower PC2 scores.

Lateral view: The first five principal components capture 77.23% of the shape variance in lateral view (PC1 - 32.64%; PC2 - 18.57%; PC3 - 11.66%; PC4 - 8.58%; PC5 - 5.76%) (Fig 2C). Shape is a statistically significant predictor of geographic location ($p = 0.001$), and the canonical variates analysis had an overall classification accuracy of 95.78%, misclassifying two Chinese specimens in the dataset.

PC1 describes differences in the profile of the snout, position of the caniniform process relative to the orbit, and the anteroposterior dimensions of the orbit (Fig 2C). Specimens with low PC1 scores have a lower, more anteriorly-convex snout profile with prefrontals that do not project much above the level of the skull roof, and the ventral edge of the caniniform process is located below the anterior orbital margin. The anteroposterior length of the orbit is relatively long. In contrast, specimens with high PC1 scores, have much taller, flatter snout profiles and more dorsally-projecting prefrontals, as well as anteroposteriorly shorter orbits and caniniform processes that are located anterior to the anterior orbital margin. PC2 also captures aspects of snout profile, including the degree of anterior projection of anteroventral corner of the premaxilla and the length of the caniniform process, as well as the dorsoventral height of the orbit. The tip of the premaxilla projects more anteriorly in specimens with low PC2 scores, the caniniform

processes are relatively short, and the orbits are dorsoventrally shorter. Specimens with high PC2 scores have taller orbits, flatter snout profiles, and longer caniniform processes. There is little differentiation of the Chinese and South African specimens on the first three PC axes.

Taken together, our morphometric analyses suggest that there are consistent differences in skull shape between *Lystrosaurus* specimens from China and from South Africa, particularly in anterior and dorsal view. Based on these results, we tentatively suggest that the Chinese and South African populations were distinct at the species level. However, because our analyses do not directly address potential synonymies among the named Chinese species, and the fact that not all of the specimens we sampled histologically are associated with cranial material, we conservatively refer all of our histological samples to *Lystrosaurus* sp.

Osteohistology Overview

Lystrosaurus limb elements, regardless of size, have characteristically thick cortices, with bone wall thicknesses ranging from 1.8 mm thick in the smallest femoral thin section, to 4 mm thick in the largest femoral thin section. Medullary cavities are typically infilled with trabecular bone that gradually transitions to a compact cortex. Similar to the bone histology of other dicynodonts and therapsids more broadly, the dominant tissue type in all sampled size classes of *Lystrosaurus* is ~~woven, fibrolamellar to laminar bone with a plexiform to longitudinal vascular orientation, indicative of rapid osteogenesis~~ [76-80, 19, 29]. Very little of the cortex is remodeled and when secondary osteons are present, they are restricted to the deepest cortex. In the small individuals of *Lystrosaurus* studied here, rapidly deposited tissue is interrupted by either lines of arrested growth (LAGs) or annuli. LAGs are found in multiple regions of the cortex, including

deep in the cortex and along the sub-periosteal margin. In some individuals, there is variability in the type of growth mark that is deposited among the skeletal elements sampled. For example, the first (i.e., osteologically deeper) growth mark of IVPP V26548 varies from a LAG in the femur and tibia, to an annulus in the fibula. Interestingly, the largest and presumably most skeletally mature femur that was histologically sampled, shows uninterrupted cortical growth, suggesting that *Lystrosaurus* from the Jiucaiyuan Formation had a high intrinsic rate of growth that could periodically arrest.

Our results are consistent with a recent report of *Lystrosaurus* bone histology from the same formation with respect to the preponderance of highly vascularized, rapidly deposited cortical bone [39]. However, contrary to Han et al. [39], we do not find evidence of peripherally slowed growth rates in individuals that are as large (~60% MKS) and larger than the maximum size sampled in their study. Additionally, we pose an alternative interpretation to the assignment of LAGs in the laminar to plexiform tissue reported in ([39]:fig 2G-H, fig 3C, fig 5, fig 6D, G-H) as bright lines that appear in the context of laminar bone formation ([81]:fig3C; [82]:fig3). Finally, we report fewer instances of LAGs, particularly in the smallest size class where no evidence of episodic growth marks is seen (contra [39]). When present, we can confidently trace 1 to 3 growth marks (as either LAGs or annuli) around the circumference of individuals that are approximately 70% maximum known size.

Size Class II

The smallest association of elements includes cranial and postcranial material (hind limb and forelimb elements) that was collected in situ. A femur, tibia, fibula, ulna and radius were selected for consumptive sampling (composite images available on Morphobank project

4023). The femur measures 122.38 mm in length. When compared to femora from larger, presumably more mature individuals, this femur (IVPP V27126a) is approximately 50% maximum known size. The bone tissue microstructure in each of the five associated limb elements is extremely well-vascularized with ~~plexiform to radial vascular canals~~ (Fig 3). Woven fibered bone matrix and large, densely distributed osteocyte lacunae surround the primary vascular canals. Vascular canal size and density remain consistently high from the endosteal to the periosteal margin indicating that this individual grew rapidly until death (Fig 3D-E). There is no evidence of episodic growth arrests (as either LAGs or annuli) in this individual.

Figure 3. Representative bone histology of Size Class I from the mid-shafts of *Lystrosaurus* sp. forelimb and hind limb elements. **A**, fibula (IVPP V27124-1) consisting of well-vascularized ~~plexiform tissue~~. **B**, 10X magnification of **A**, arrows indicate regions ~~of woven bone matrix~~ in the ~~laminar, fibrolamellar~~ complex that should not be confused as ~~LAGs~~. **C**, 20X magnification of **B**. **D**, fibula (IVPP V27126c) cross section consisting of ~~plexiform~~ canals and dense osteocyte lacunae. **E**, cross section of the ulna from the same individual (IVPP V27126e) similarly shows a cortex of highly vascularized plexiform tissue indicating rapid bone deposition at death. **F**, cross section of a proximal tibia (IVPP V27124-3) with a slight decrease in vascular canal sizes towards the sub-periosteal edge, magnified in **G**. po= primary osteon. **B**, left side of **C**, and **E** show cross-polarized light with a lambda filter. Scale bars = 1 mm in **A** & **F**, 500 μm in **D**, **E**, **G**, 100 μm in **C**&**E**.

A right proximal fibula (IVPP V27124-1), right proximal tibia (IVPP V27124-3), distal tibia (IVPP V27124-4), distal femur (IVPP V27124-6) and complete femur (IVPP V27124-7)

were collected individually but are analyzed together here as immature individuals because limb dimensions and histological features are indicative of sustained, active bone growth at death. The complete femur is approximately 50% maximum known size, similar to IVPP V27126a. In thin section, each of these disassociated elements has a large medullary cavity infilled with a loose trabecular network. Cortical tissue consists of fibrolamellar bone with plexiform to longitudinal canal orientations. In the proximal tibia, vascular canal size decreases substantially in a regionalized band deep to the sub-periosteal edge but rebounds to the typical highly vascularized tissue more peripherally (Fig 3F-G). In the fibula, there are regional differences in vascular canal size and organization that likely represent functional differences in bone apposition rates [83]. For example, longitudinal vascular canals are organized into stacked, circumferential layers on the medial side of the proximal fibula (IVPP V27124-1) whereas the lateral side is less organized (Fig 3A). In more organized areas of the highly vascularized fibrolamellar tissue, the mineralized matrix surrounding the reticular vascular canals should not be confused for lines of arrested growth (Fig 3B-C). At low magnifications, delineated bands of matrix separate the primary vascular space in laminar bone [82]. However, at high magnifications, these bright bands do not form continuous rings around the cortex, nor are they hyper-mineralized, two of the most diagnostic characteristics of lines of arrested growth [41]. Highly vascularized, reticular fibrolamellar bone continues to the sub-periosteal edge in these specimens in Size Class II, indicating that individuals died while actively growing.

Size Class III

A femur (IVPP V27125a), tibia (IVPP V27125b), and fibula (IVPP V27125c) are associated as one individual that is approximately 70% maximum size based on femoral length.

Unlike smaller sampled elements, the cortex is interrupted by two growth marks (Fig 4). In all associated elements, a deep cortical LAG is sandwiched between large vascular canals towards the endosteal margin and smaller vascular canals more peripherally (Fig 4A-B, lowermost arrows in F, K). In the fibula, the LAG is superficial to a region of immature secondary osteons that sit in the deep cortex (Fig 4J). The second of the two growth marks is more variable. In the femur, a region of stacked, circumferentially organized, and poorly vascularized tissue sits along the sub-periosteal edge in thin areas of the cortex (Fig 4D). By contrast, thicker regions of the outer cortex show continuous laminar to fibrolamellar bone tissue (Fig 4C). In the tibia and fibula, the outer cortical growth mark is made up of two LAGs in the tibia, and an annulus ~~of~~ stacked LAGs that vary in number around the cross-section of the fibula (Fig 4E, J-K). Interestingly, the mineral preservation of the tibia and fibula are such that the mineralized bone matrix is clearly visible between each layer of vascular canals (Fig 4F, K, L). These thin, mineralized lines of extracellular matrix should not be confused for LAGs and instead are the mineralizing front of ~~laminar, fibrolamellar bone~~ ~~deposition and growth~~ [41, 81]. The vascular orientation shifts in the hindlimb elements of this individual from plexiform to longitudinal in the middle to outer cortex. In addition, vascular canal size decreases towards the outermost periosteal margin but remains open to the sub-periosteal edge (Fig 4F-G). The only exception to this is in the thinnest region of the cortices, particularly in the fibula, where the sub-periosteal edge is scarcely vascularized and instead, consists of well organized, lamellar tissue (Fig 4D, I, K). However, this lamellar tissue is discontinuous around the cross section, suggesting that bone growth slowed at differential rates in regions of the outer cortex, but did not fully arrest

Figure 4. Bone histology of associated hind limb elements of *Lystrosaurus* sp. representative of Size Class III. **A**, femur (IVPP V27125a) with a deep cortical LAG in **B** (blue box). The outer cortex has regions of high vascularity seen in **C** (yellow box in **A**), and regions of lower vascularity seen in D (orange box in **A**). **E**, tibia (IVPP V27125b) has two cortical growth marks seen in **F** (yellow box in **E**; yellow arrows in **F**). The outer growth mark can variably be seen as two LA[illegible] **G** (green box in **E**; yellow arrows in **G**). **H**, the fibula (IVPP V27125c) has a thinner cortex interrupted by two LAGs seen in **I** (yellow box in **H**; yellow arrows in **I**). **J**, magnification of yellow box in **I** shows the cross-cutting inner cortical LAG (arrow in **J**). **K**, thinner regions of the cortex appear avascular (purple box in **H**), with stacked mineralized lamellae (yellow bracket), whereas thicker regions (blue box in **H**) show peripheral pulses of vascularized growth, within the lamellated tissue, seen in **L** (yellow bracket). **C**, **D**, **I**, and **J**, left sides of **F**, **G**, **K**, and **L** show cross-polarized light with a lambda filter. Scale bars = 5 mm in **A**, 1 mm in **E**, 2 mm in **H**, 500 µm in **B**, **C**, **F** & **I**, and 100 µm in **D**, **G**, **J**-**L**.

A slightly larger specimen (IVPP V26548) is an association of hind limb and forelimb elements as well as at least one rib that was previously histologically sampled by Han et al. 2021 [39]. The femur (IVPP V26548a), tibia (IVPP V26548b), and fibula (IVPP V26548c) show plexiform tissue interrupted by two growth marks, with some variation in the outer growth mark, especially in the fibula where it splits into two LAGs (Fig 5). In the femur and tibia, the deeper growth mark is distinguishable as a dramatic reduction in vascular canal size, immediately followed by a return to the typical, large vascular canals in the middle of the cortex (Fig 5A, B, D). Throughout the middle cortex of the femur, the deeper growth mark changes in thickness. In thinner regions of the cortex, the growth mark appears as a cross-cutting LAG separating smaller

vascular canals from larger canals more superficially. In the thicker regions of the cortex, the growth mark appears as an annulus, or region of little to no vasculature that is not accompanied by a hyper-mineralized line (Fig 5D). In both the femur and tibia, vascular canals reduce in size again towards the sub-periosteal margin, making up the second growth mark of stacked lamellar tissue (Fig 5C). In contrast, the fibula shows a mid-cortical growth mark that is made up of multiple stacked LAGs in a circumferential band of reduced vascularity (Fig 5G, D[illegible] Similar to the femur and tibia, there is a sub-periosteal reduction in vascular canal size with at least two LAGs that can be traced around the entire cortex of the fibula, indicating variation in the type of growth mark deposited in hind limb elements of the same individual (Fig 5E, G, H). We tentatively interp[illegible] the presence of stacked lamellar layers in this individual as a cessation in growth. However, small, simple vascular canals sit within the context of this organized lamellar tissue, which is atyp[illegible] for an EFS, making it more likely that this lamellar tissue represents a temporary cessation in growth, similar to the annuli recorded in the deeper cortex.

Figure 5. Bone histology of associated elements of *Lystrosaurus* sp. representative of Size Class III. **A**, femur (IVPP V26548a) has two growth marks. **B**, a deep, mid-cortical annulus (yellow box in **A**; lower yellow bracket in **B**), stacked lamellar tissue makes up the second growth mark (upper yellow bracket in **B**). **C**, magnification of peripheral lamellar tissue (blue box in **A**; yellow bracket in **C**). **D**, first (osteologically deeper) annulus (yellow bracket). **E**, magnification of blue box in **A** of outer cortical growth mark that varies in thickness around the periphery of the thin section, here consisting of two closely spaced LAGs. **F**, associated fibula (IVPP V26548c) highlights a similar arrangement of deep and more superficial growth marks consisting of, at times, numerous layers of lamellar tissue in an annulus. **G**, magnification of yellow box in

F showing two growth marks; annulus (yellow bracket) and peripheral LAGs (yellow arrows). **H**, magnification of green box in **F** showing variation in the outer growth mark such that in some regions it appears as a thicker region of lamellar tissue with one cross-cutting LAG. **I**, magnification of blue box in **F**; deep cortical annulus (bracket). **J**, humerus with mid-cortical annulus of reduced vascularity. **K**, magnification of yellow box in **J** of growth mark (yellow bracket). **L**, ulna (IVPP V27124-2) with thinner overall cortex and reduced vasculature. **M**, magnification of yellow box in **L** highlighting the disorganized cortex in plane and cross-polarized light with a lambda filter. Left side of **C**, **D**, **H**, **I**, and upper portion of **M** show cross-polarized light and a lambda filter. Scale bars = 1 mm in **A**, **B**, **F**, **J**, **L**, 500 μm in **E**, **G**, **I**, **K**, and 100 μm in **C**, **D**, **H**, **M**.

A complete humerus (IVPP V27124-8) of comparable size to IVPP V26548, has an exceedingly thick cortex with one mid-cortical growth mark (Fig 5J). The medullary cavity is nearly infilled by a dense network of trabecular bone. The cortex increases in thickness in the anterodorsal region (up in Fig 5J) and consists of highly vascularized laminar to fibrolamellar bone. Vascular canals have a circular to plexiform orientation and show a marked decrease in size coincident with a mid-cortical LAG (Fig 5K). The LAG is traceable around the entire cross-section and is followed by a pulse of highly vascularized reticular tissue with large canal spaces (Fig 5K). Vascular canal size reduces slightly towards the sub-periosteal margin, which could indicate the beginning of another growth mark, similar to the mid-cortical growth mark.

A fragment of a distal ulna (IVPP V27124-2) is estimated to be 93 mm long and has a thin cortex with a large medullary cavity infilled with a loose network of trabecular bone (Fig 5L). The transition from trabeculae to compact cortical bone includes a region of enlarged

resorption cavities and immature secondary osteons. The middle and outer cortex is composed of small longitudinal and reticular canals that sit in a parallel-fibered matrix where small, lenticular osteocyte lacunae are sparsely distributed. Along the sub-periosteal margin, vascular canals are reduced but occasionally open to the bone edge, suggesting that this individual died while still growing (Fig 5M). The overall construction of this element is suggestive of a slower growth rate when compared to other long bones, but there is no evidence to suggest somatic maturity or attainment of maximum size.

Size Class IV

Unlike the middle size class, the largest sampled femur (IVPP V27127) does not have LAGs or annuli (Fig 6A). Despite cracks in the cortex, the bone tissue architecture is easily discernible and consists of ~~longitudinal~~ canals in plexiform to circular organization. The fiber-orientation is less clear due to the poor preservation of this specimen, but it appears parallel-fibered in the deep cortex and woven in the outer cortex, of which, only a small portion remains intact (Fig 6A-C). There is no indication of slowed bone deposition in the small regions of sub-periosteal bone, suggesting a sustained rate of growth at death.

Figure 6. Bone histology of the largest femur (IVPP V27127) of *Lystrosaurus* sp. that represents Size Class IV and rib fragments of unknown size. **A**, the femur is cracked and incomplete but preserves regions of the entire cortex (yellow box). **B**, same view of the yellow box in **A** shows parallel-fibered bone in the mid cortex, a large crack (yellow arrow), and well-vascularized, plexiform tissue along the sub-periosteal margin (yellow box in **B**). **C**, magnification of yellow box in **B** shows sustained, rapid growth at death. **D**, rib fragments (IVPP V27124-5) have infilled

medullary cavities, secondary osteons within the deep cortex and a largely avascular compact outer cortex consisting of an EFS. **E**, magnification of yellow box in **D** showing an EFS in the yellow bracket. **F**, magnification of the yellow box in **E**. **G**, magnification of blue box in **D** similarly shows that an EFS is present (yellow bracket). **B**, left side of **C**, **F**, and **G**, show cross-polarized light with a lambda filter. Scale bars = 10 mm in **A**, 1 mm in **B** & **D**, 500 μm in **C** & **G**, 100 μm in **E** & **F**.

Unknown Size Class

Four partial ribs (IVPP V27124-5) belonging to an individual of unknown size class are preserved in a block of sediment (Fig 6D). In thin section, they have large, infilled medullary cavities that grade into thin, compact cortices. Scarce vascular canals and immature secondary osteons sit in the deeper cortex whereas the outer cortex is avascular with an EFS of stacked, circumferential LAGs (Fig 6E-G). Osteocyte lacunae are well organized in this lamellar tissue (Fig 6F).

Discussion

All *Lystrosaurus* individuals sampled from the Jiucaiyuan Formation show abundant, highly vascularized primary bone tissue throughout the majority of the cortices of the hind limb, humerus, and radius. The ulna and ribs showed fewer vascular canals, overall thinner cortices, and more organized primary bone tissue. Three size classes were identified and histologically described as: II) a highly vascularized cortex of radial to plexiform vascular canals in ~50% max known size individuals: III) highly vascularized plexiform tissue interrupted by one to two growth marks that range from a single LAG or set of closely spaced LAGs to annuli in ~70%

maximum known size individuals; and IV) uninterrupted plexiform tissue in a 100% maximum known size individual. These results force us to reconsider what an adult-sized specimen might look like for *Lystrosaurus* sp., as the current sample lacks histologic indicators of somatic maturity (i.e., an external fundamental system) [75]. Botha [22] found similar results for South African species of *Lystrosaurus*, suggesting that species typified by small body size (viz. *L. murrayi*) were very likely capable of reaching much larger sizes, but never lived long enough to do so.

Han et al. [39] suggested that sub-adult *Lystrosaurus* from northern Pangea may have reached some level of somatic maturity inferred from parallel-fibered bone and peripheral LAGs in a dorsal rib. However, histological data from the femur, tibia, and fibula of the same individual indicate episodic cessations in growth instead of an overall pattern of slowed growth. The presence of slower growing bone tissue in the ribs likely reflects differential growth rates seen throughout the skeleton, rather than somatic maturity [84, 45].

Growth Marks

Unlike the bone histology reported from South African and Indian *Lystrosaurus*, in which growth marks are rare and often limited to a single LAG or annulus, we find one, and more commonly, two growth marks in immature *Lystrosaurus* from the Jiucaiyuan Formation. The number of growth marks is mostly consistent across associated elements from the same individual as well as across individuals of the same size class (Table 1). Interestingly, the type of growth mark, as either a LAG, a series of closely spaced and occasionally bifurcating LAGs, annulus consisting of stacked LAGs or annulus consisting of avascular tissue, varied throughout the current sample. In most cases, the first (i.e., osteologically deeper) growth mark, or singular

growth mark in the case of the humerus, was often recorded as an annulus accompanied by a reduction of vascular canal sizes. The outer cortical growth marks were more variable, and often varied within the same thin section.

We did not find evidence of growth marks in individuals in Size Class II. These individuals had characteristic juvenile bone histology (i.e., large radial to reticular vascular canals); therefore our analysis disagrees with the assignment of LAGs in juvenile *Lystrosaurus* by Han et al. [39]:fig 3. In some cases, the mineralizating front can be mis-identified as a LAG in the context of laminar or plexiform tissue, wherein one unit of laminar bone is constructed by a mineralized line upon which woven bone is deposited [81]. This mineralized line differs from a LAG as it is not continuous around the cortex and is not hyper-mineralized but instead appears as a bright line often devoid of osteocyte lacunae [81]. A LAG on the other hand, is a continuous mineralized line around the cortex, unless there is clear evidence for secondary remodeling or cortical remodeling that disrupts (i.e., erodes) it.

All growth marks observed in our analysis come from Size Class III, which corresponds to individuals that are approximately 70% maximum known size. We can confidently trace up to two growth marks (occasionally made up of multiple LAGs) in the current Chinese sample, which is higher than reports from South African and Indian specimens of *Lystrosaurus,* which generally lack growth marks. However, the remainder of the cortical bone tissue in the current Chinese sample reflects the similarly high and consistent rate of bone growth reported from South African and Indian *Lystrosaurus* [22, 51]. A slight increase in the number of growth marks in northern Pangean *Lystrosaurus* could be explained by one or two possible factors that are not mutually exclusive: (i) by an increase in their lifespan or (ii) by environmental stressors.

There is a longstanding hypothesis that the Permo-Triassic extinction caused a Lilliput effect, a persistent reduction in body size, in surviving terrestrial lineages [85-88, 19]. This claim has been investigated histologically in therocephalians, which support an overall reduction in body size following the Permo-Triassic extinction [88, 19]. A Lilliput effect was also posited for *Lystrosaurus*, based in large part on the overwhelming abundance of small skulls in the Karoo Basin and museum collections in South Africa [89, 90, 20, 21]. However, Botha [22] later rejected a strict Lilliput effect for southern Pangean *Lystrosaurus* (based on fossils from South Africa and India) based on the lack of mature histological tissue in all of the species examined [22]. Instead, she [22] suggested that the large proportion of small skulls in the Triassic was better explained by high mortality in immature individuals, as assessed by their long bone histology.

Body size

In order to better understand how *Lystrosaurus* from the Jiucaiyuan Formation compare to congeners from more southerly parts of Pangea, we gathered body size data (based on basal skull length and femur length) from the literature [51, 22, 39] and personal observations from Early Triassic-aged specimens collected in China, South Africa, and India. From this sample, it is clear that Chinese *Lystrosaurus* have larger average body size (Fig 7, Fig 8). However, cranial size distributions suggest that populations from both South Africa and China had the potential to grow to similar maximum sizes (Fig 7). The lack of large specimens of *Lystrosaurus* in South Africa aligns with previous interpretations for increased mortality at small sizes there by Botha [22]; also see [20]. Our data also provide evidence suggesting that northern Pangean *Lystrosaurus* could have reached larger sizes more readily or easily. However, it is unclear the

degree to which differences in sampling practices might affect the body size distributions seen here. A decade of detailed stratigraphic work targeting the Permo-Triassic boundary in South Africa has led to more comprehensive fossil sampling, which should provide a relatively unbiased view of the vertebrate fauna [91, 92]. However, historical collections from the Karoo likely systematically emphasized rare components of the assemblage (e.g., [93]). Likewise, the Permo-Triassic vertebrate record from Xinjiang has not been subject to the same intensive collecting as that of the Karoo Basin, and therefore it currently may not provide an unbiased view of body size distributions or relative abundances of taxa.

Figure 7. Interspecific body size distributions from Early Triassic *Lystrosaurus* show an abundance of large individuals from China. South African and Chinese species overlap in maximum basal skull length but individuals from China have larger average size. See S2 Supplementary Dataset for more information.

Figure 8. Interspecific femoral lengths from Early Triassic *Lystrosaurus* show a higher frequency of large sizes from China. Size Classes denote femora that are less than 40% maximum known size (MKS) in I, between 40-65% MKS in II, approximately 70-75% MKS in III, and greater than 79% in IV. See S2 Supplementary Dataset for more information.

The presence of large individuals with episodic growth marks in all of the subadult-sized elements suggests that *Lystrosaurus* from the Jiucaiyuan Formation were able to reach large sizes through prolonged and sustained rapid growth, unlike Triassic *Lystrosaurus* from South Africa that rarely lived long enough to reach large body sizes despite growing rapidly (Fig 7).

There are several possible explanations for this pattern. First, physiological differences among the species of *Lystrosaurus* could be responsible, especially given our morphometric results, which suggest that the South African and Chinese populations were distinct. This proposal is difficult to test at this time, as the alpha-taxonomy of Chinese *Lystrosaurus* is in need of detailed revision and phylogenetic studies of the genus have typically only included *L. hedini* as a sole northern hemisphere representative (e.g., [94]). However, to the degree that patterns preserved in hard tissue histology can shed light on physiology, the current data suggests that *L. curvatus*, *L. declivis*, *L. maccaigi*, and *L. murrayi* are effectively indistinguishable [22, 30], which suggests a degree of consistency among species within the genus.

Climatic differences in the high-southern (60°S) versus mid-northern (45°N) paleolatitudes [95, 96] are another possible explanation for the observed difference in body size and inferred lifespan in *Lystrosaurus*. Paleoclimate reconstructions of the latest Permian to earliest Triassic show extreme increases in temperature and aridity [97-100], mega monsoons [101, 102], chemical weathering, soil erosion, and geochemical and isotopic signatures of mass wasting and greenhouse conditions [8, 96, 103, 104]. Facies that span the terrestrial Permo-Triassic boundary of the Karoo Basin are interpreted as a change from meandering rivers to low-sinuosity braid-plains, resulting from increased aridity and a die-off of rooted plants that supported river morphologies [105, 106]. However, more recent evidence from isotopic and geochemical data produced differing estimates of the degree to which aridity increased in the Karoo Basin, complicating our understanding of seasonal variation in precipitation and resource availability [102, 107, 108]. Nevertheless, conditions were disruptive enough to have destabilized terrestrial communities and caused a prolonged interval of faunal turnover in southern Pangea during the extinction interval [109, 15, 18].

The extreme environmental and climatic conditions of southern Pangea are not reflected in the paleoclimate reconstructions of the Jiucaiyuan Formation [72]. Here, stable subhumid to semiarid conditions are interpreted from cyclostratigraphic and paleosol analyses in the South Taodonggou study area [71, 72]. Furthermore, fluvial and lacustrine strata host a diversity of plant, root, and wood fossils that are occasionally preserved in situ and do not preserve cyclical growth marks when thin-sectioned [110-112, 72]. The absence of growth rings in these woody pieces provides some evidence for stable environmental and climatic conditions. In sum, evidence from the paleobotanical record of the Jiucaiyuan Formation suggests a lush and vegetated environment that hosted a diverse terrestrial fauna in earliest Triassic times [61, 39]. Taken together, there is mounting evidence from sedimentologic, isotopic, and cyclostratigraphic data that points to seasonal, semi-arid conditions in the Karoo Basin and stable, sub-humid conditions in the Turpan-Hami Basin.

The extreme environmental and climatic conditions inferred from the Karoo Basin likely truncated *Lystrosaurus* lifespans in the Early Triassic. Similar environmental constraints on maximum size and longevity are also known from Early Triassic therocephalians and terrestrial temnospondyls, which also lack LAGs [19, 49]. By contrast, we propose that the more favorable environmental conditions and vegetated landscape interpreted from the Jiucaiyuan Formation could have hosted longer-lived *Lystrosaurus* during the Early Triassic. Relatively stable environmental conditions could have allowed populations of *Lystrosaurus* to reach larger average body sizes than their southern Pangean relatives. The prevalence of multiple growth marks in some, but not all, immature individuals, further supports developmentally plastic growth in *Lystrosaurus* first proposed for southern Pangean populations [22]. It also suggests that some individuals experienced periodic arrests of growth, perhaps associated with

environmental disruptions, but that these disturbances may not have caused widespread mortality on the scale implied by the South African record. Similarly, some individuals apparently experienced sufficiently stable conditions for them to grow to comparatively large size without interruption. Finally, the absence of peripheral parallel-fibered bone or consistent, circumferential lamellar tissue in the current sample indicates that the maximum size of *Lystrosaurus* from the Jiucaiyuan Formation remains unknown.

Conclusions

We present bone histology, cranial geometric morphometric analyses, and body size data for *Lystrosaurus* from the Jiucaiyuan Formation, and report statistically different cranial morphologies, an extended lifespan, and larger average body size compared to *Lystrosaurus* living in southern Pangea during Early Triassic times. In addition, we report up to two growth marks in immature individuals, which is inconsistent with previous reports of *Lystrosaurus* life histories from the Turpan Basin [39]. We suggest that it is unlikely that the growth marks reported here represent regular, cyclical patterns and more likely represent instances where bone growth stopped due to unfavorable environmental conditions. Indeed, the absence of growth marks in the largest sampled individual indicates an intrinsically high rate of growth that persisted throughout ontogeny. In addition, the presence of two cessations in growth in individuals from Size Class III indicates that smaller, likely more immature, individuals may have been more susceptible to fluctuations in environmental conditions. However, appositional bone growth rebounded to a rapid rate following these cessations, indicating a flexible physiology, as previously proposed for higher-latitude populations of *Lystrosaurus* [22, 30]. A better sample of large limb bones (>250 mm) as well as an analysis of tusk dentine deposition,

are needed to clarify what impact the Early Triassic environment may have had on *Lystrosaurus* growth and development in northern Pangea.

Acknowledgements. —This research was supported by National Science Foundation grants EAR 1714829 (to KDA) and EAR 1713787 (to CAS), and UW Biology Department Iuvo and Walker awards (to ZTK). At the IVPP, we thank Yi Jian and Shukang Zhang for assistance with thin section preparation as well as Jun Liu for access to field data and specimen collections. We also thank Alida Bailleul for helpful discussion and microscope access. Collecting and compiling the entirety of the comparative datasets would not have been possible if it weren't for the following museum curators, collection managers, colleagues, and friends who we deeply thank: Zaituna Skosan, Claire Browning, Christian Kammerer, Roger Smith, Jennifer Botha, Elize Butler, Bernhard Zipfel, Bruce Rubidge, Sifelani Jirah, Viktor Radermacher, Carl Mehling, and Mark Norell. For their helpful discussion on earlier versions of this manuscript, we thank Bryan Gee, Savannah Olroyd, and Elliott Armour Smith. Christian Kammerer provided images of South African Lystrosaurus specimens that were utilized in the morphometric analyses. Finally, we are indebted to Wan Yang for collecting some of the fossils analyzed here, organizing fieldwork in 2019 and leading the multidisciplinary project that instigated this research.

References

1. Raup DM, Sepkoski JJ. Periodicity of extinctions in the geologic past. Proc Natl Acad Sci. 1984 Feb 1;81(3):801–5.

2. Erwin DH. The Permo–Triassic extinction. Nature. 1994 Jan;367(6460):231–6.

3. Chen Z-Q, Benton MJ. The timing and pattern of biotic recovery following the end-Permian mass extinction. Nat Geosci. 2012 Jun;5(6):375–83.

4. Benton M. Diversification and extinction in the history of life. Science. 1995 Apr 7;268(5207):52–8.

5. Benton MJ, Twitchett RJ. How to kill (almost) all life: the end-Permian extinction event. Trends Ecol Evol. 2003 Jul;18(7):358–65.

6. Sahney S, Benton MJ. Recovery from the most profound mass extinction of all time. Proc R Soc B Biol Sci. 2008 Apr 7;275(1636):759–65.

7. Sobolev SV, Sobolev AV, Kuzmin DV, Krivolutskaya NA, Petrunin AG, Arndt NT, et al. Linking mantle plumes, large igneous provinces and environmental catastrophes. Nature. 2011 Sep;477(7364):312–6.

8. Benton MJ, Newell AJ. Impacts of global warming on Permo-Triassic terrestrial ecosystems. Gondwana Res. 2014 May;25(4):1308–37.

9. Retallack GJ, Krull ES. Carbon isotopic evidence for terminal-Permian methane outbursts and their role in extinctions of animals, plants, coral reefs, and peat swamps. In: Greb SF, DiMichele WA, editors. Wetlands Through Time: Geological Society of America Special Paper. 2006. p. 249–68.

10. Nowak H, Schneebeli-Hermann E, Kustatscher E. No mass extinction for land plants at the Permian–Triassic transition. Nat Commun. 2019 Dec;10(1):384.

11. Mishra S, Jha N, Stebbins A, Brookfield M, Hannigan R. Palaeoenvironments, flora, and organic carbon and nitrogen isotope changes across the non-marine Permian-Triassic boundary at Wybung Head, Australia. Palaeogeogr Palaeoclimatol Palaeoecol. 2019 Nov;534:109292.

12. Botha J, Smith RMH. Rapid vertebrate recuperation in the Karoo Basin of South Africa following the End-Permian extinction. J Afr Earth Sci. 2006 Aug 1;45(4):502–14.

13. Botha J, Abdala F, Smith R. The oldest cynodont: new clues on the origin and early diversification of the Cynodontia. Zool J Linn Soc. 2007;149(3):477–92.

14. Fröbisch J. Vertebrate diversity across the end-Permian mass extinction — Separating biological and geological signals. Palaeogeogr Palaeoclimatol Palaeoecol. 2013 Feb;372:50–61.

15. Roopnarine PD, Angielczyk KD, Weik A, Dineen A. Ecological persistence, incumbency and reorganization in the Karoo Basin during the Permian-Triassic transition. Earth-Sci Rev. 2019 Feb;189:244–63.

16. Bernardi M, Petti FM, Benton MJ. Tetrapod distribution and temperature rise during the Permian–Triassic mass extinction. Proc R Soc B Biol Sci. 2018 Jan 10;285(1870):20172331.

17. Allen BJ, Wignall PB, Hill DJ, Saupe EE, Dunhill AM. The latitudinal diversity gradient of tetrapods across the Permo-Triassic mass extinction and recovery interval. Proc R Soc B Biol Sci. 2020 Jun 24;287(1929):20201125.

18. Viglietti PA, Benson RBJ, Smith RMH, Botha J, Kammerer CF, Skosan Z, et al. Evidence from South Africa for a protracted end-Permian extinction on land. Proc Natl Acad Sci. 2021 Apr 27;118(17):e2017045118.

19. Huttenlocker AK, Botha-Brink J. Bone microstructure and the evolution of growth patterns in Permo-Triassic therocephalians (Amniota, Therapsida) of South Africa. PeerJ. 2014 Apr 8;2:e325.

20. Botha-Brink J, Codron D, Huttenlocker AK, Angielczyk KD, Ruta M. Breeding Young as a Survival Strategy during Earth's Greatest Mass Extinction. Sci Rep. 2016 Jul;6(1). Available from: http://www.nature.com/articles/srep24053

21. Codron J, Botha-Brink J, Codron D, Huttenlocker AK, Angielczyk KD. Predator-prey interactions amongst Permo-Triassic terrestrial vertebrates as a deterministic factor influencing faunal collapse and turnover. J Evol Biol. 2017 Jan;30(1):40–54.

22. Botha J. The paleobiology and paleoecology of South African *Lystrosaurus*. PeerJ. 2020 Nov 24;8:e10408.

23. Nicolas M, Rubidge BS. Changes in Permo-Triassic terrestrial tetrapod ecological representation in the Beaufort Group (Karoo Supergroup) of South Africa. Lethaia. 2010 Mar;43(1):45–59.

24. Smith R, Rubidge BS, van der Walt M. Therapsid Biodiversity Patterns and Paleoenvironments of the Karoo Basin, South Africa (pp. 31-63) Roger Smith, Bruce Rubidge and Merrill van der Walt. In: The Forerunners of Mammals. Indiana University Press; 2012.

25. King GM, Cluver MA. The Aquatic *Lystrosaurus*: An Alternative Lifestyle. Hist Biol. 1991;4:323–41.

26. Jasinoski SC, Rayfield EJ, Chinsamy A. Comparative Feeding Biomechanics of *Lystrosaurus* and the Generalized Dicynodont *Oudenodon*. Anat Rec Adv Integr Anat Evol Biol. 2009 Jun 1;292(6):862–74.

27. Retallack GJ, Smith RMH, Ward PD. Vertebrate extinction across Permian–Triassic boundary in Karoo Basin, South Africa. Geol Soc Am Bull. 2003;115(9):1133.

28. Botha-Brink J. Burrowing in *Lystrosaurus*: preadaptation to a postextinction environment? J Vertebr Paleontol. 2017 Sep 3;37(5):e1365080.

29. Botha-Brink J, Angielczyk KD. Do extraordinarily high growth rates in Permo-Triassic dicynodonts (Therapsida, Anomodontia) explain their success before and after the end-Permian extinction? Zool J Linn Soc. 2010 Oct 1;160(2):341–65.

30. Whitney MR, Sidor CA. Evidence of torpor in the tusks of *Lystrosaurus* from the Early Triassic of Antarctica. Commun Biol. 2020;3(1). Available from: http://www.nature.com/articles/s42003-020-01207-6

31. Tripathi C, Puri SN. On the remains of *Lystrosaurus* from the Panchets of the Raniganj coalfield. Rec Geol Surv India. 1961;89:407–19.

32. Colbert EH. *Lystrosaurus* from Antarctica. Am Mus Novit. 1974;(2535):44.

33. Gubin Y, Sinitza S. Triassic terrestrial tetrapods of Mongolia and the geological structure of the Sain-Sar-Bulak locality. In: The Nonmarine Triassic. 1993. p. 169–70.

34. Liu J, Li J, Cheng Z. The *Lystrosaurus* fossils from Xinjiang and their bearing on the terrestrial Permian Triassic boundary. Vertebr Pal Asiat. 2002;40(4):267–75.

35. Sun A. Late Permian and Triassic Terrestrial Tetrapods of North China. Vertebr Palasiat. 1980;XVIII(2):11.

36. Ray S. *Lystrosaurus* (Therapsida, Dicynodontia) from India: Taxonomy, relative growth and Cranial dimorphism. J Syst Palaeontol. 2005 Jan;3(2):203–21.

37. Surkov MV, Kalandadze NN, Benton MJ. *Lystrosaurus georgi*, a dicynodont from the Lower Triassic of Russia. J Vertebr Paleontol. 2005 Jun 27;25(2):402–13.

38. Araújo R, Fernandez V, Polcyn MJ, Fröbisch J, Martins RMS. Aspects of gorgonopsian paleobiology and evolution: insights from the basicranium, occiput, osseous labyrinth, vasculature, and neuroanatomy. PeerJ. 2017 Apr 11;5:e3119.

39. Han F, Zhao Q, Liu J. Preliminary bone histological analysis of *Lystrosaurus* (Therapsida: Dicynodontia) from the Lower Triassic of North China, and its implication for lifestyle and environments after the end-Permian extinction. Fröbisch J, editor. PLOS ONE. 2021 Mar 18;16(3):e0248681.

40. Padian K, Lamm E-T, editors. Bone histology of fossil tetrapods: advancing methods, analysis, and interpretation. Berkeley: University of California Press; 2013. 285 p.

41. Francillon-Vieillot H, de Buffrénil V, Castanet J, Géraudie J, Meunier FJ, Sire JY, et al. Microstructure and Mineralization of Vertebrate Skeletal Tissues. In: Skeletal

Biomineralization: Patterns, Processes and Evolutionary Trends. American Geophysical Union; 1990. p. 175–234. Available from: http://dx.doi.org/10.1029/SC005p0175

42. Enlow H, Brown SO. A Comparative Histological Study of Fossil and Recent Bone Tissues. Part II. Tex J Sci. 1957;9(2):30.

43. Enlow H, Brown SO. A comparative histological study of fossil and recent bone tissues, Part III. Tex J Sci. 1958;10(2):187–230.

44. Castanet J, Newman DG, Girons HS. Skeletochronological Data on the Growth, Age, and Population Structure of the Tuatara, *Sphenodon punctatus*, on Stephens and Lady Alice Islands, New Zealand. 1988;14.

45. Castanet J. Time recording in bone microstructures of endothermic animals; functional relationships. Comptes Rendus Palevol. 2006 Mar;5(3–4):629–36.

46. Köhler M, Marín-Moratalla N, Jordana X, Aanes R. Seasonal bone growth and physiology in endotherms shed light on dinosaur physiology. Nature. 2012 Jul;487(7407):358–61.

47. Nacarino-Meneses C, Köhler M. Limb bone histology records birth in mammals. PLOS ONE. 2018 Jun 20;13(6):e0198511.

48. Sinsch U, Oromi N, Sanuy D. Growth marks in natterjack toad (*Bufo calamita*) bones: histological correlates of hibernation and aestivation periods. 2007;9.

49. Canoville A, Chinsamy A. Bone Microstructure of the Stereospondyl *Lydekkerina Huxleyi* Reveals Adaptive Strategies to the Harsh Post Permian-Extinction Environment. Anat Rec. 2015 Jul;298(7):1237–54.

50. Mchugh JB. Paleohistology of *Micropholis stowi* (Dissorophoidea) and *Lydekkerina huxleyi* (Lydekkerinidae) humeri from the Karoo Basin of South Africa, and implications for bone microstructure evolution in temnospondyl amphibians. J Vertebr Paleontol. 2015 Jan 2;35(1):e902845.

51. Ray S, Chinsamy A, Bandyopadhyay S. *Lystrosaurus murrayi* (therapsida, Dicynodontia): Bone Histology, Growth and Lifestyle Adaptations. Palaeontology. 2005 Nov 1;48(6):1169–85.

52. Yuan PL, Young CC. On the Occurrence of *Lystrosaurus* in Sinkiang. Bull Geol Soc China. 1934;13(1):575–80.

53. Young CC. On two skeletons of Dicynodontia. Bull Geol Soc China. 1935;(14):483–517.

54. Young CC. Additional Dicynodontia remains from Sinkiang. Bull Geol Soc China. 1939;(19):111–36.

55. Sun A-L. Preliminary report on a new species of *Lystrosaurus* of Sinkiang. Vertebr Pal Asiat. 1964;8:216–7.

56. Sun A-L. Permo-Triassic dicynodonts from Turfan, Sinkiang. Permo-Triassic Vertebr Foss Turfan Basin. 1973;53–68.

57. Cheng Z. Vertebrates. In: Permian and Triassic Strata and Fossil Assemblages in the Dalongkou Area of Jimsar, Xinjiang. 1986. p. 207–18. (People’s Republic of China Ministry of Geology and Mineral Resources Geological Memoirs).

58. Cluver MA. The cranial morphology of the dicynodont genus *Lystrosaurus* [Doctoral dissertation]. [Stellenbosch]: Stellenbosch University; 1970.

59. Cosgriff JW, Hammer WR, Ryan WJ. The Pangaean Reptile, *Lystrosaurus maccaigi*, in the Lower Triassic of Antarctica. J Paleontol. 1982;56(2):371–85.

60. King G. Anomodontia. Encycl Paleoherpetology. 1988;1–74.

61. Lucas SG. Chinese Fossil Vertebrates. Columbia University Press; 2001. 390 p.

62. Li J, Sun A. Subclass Synapsida. In: The Chinese Fossil Reptiles and Their Kin. Sci. Press; 2008. p. 379–409.

63. Fröbisch J. Composition and similarity of global anomodont-bearing tetrapod faunas. Earth-Sci Rev. 2009 Aug;95(3–4):119–57.

64. Camp J, Liu J. The taxonomy and cranial morphology of Chinese *Lystrosaurus*. J Vertebr Paleontol. 2011;31(3).

65. Camp J. Morphological variation and disparity in *Lystrosaurus*. University of Iowa; 2010.

66. Rohlf FJ. tpsDig. Stony Brook, NY: Department of Ecology and Evolution, State University of New York at Stony Brook; 2010.

67. Kammerer CF, Deutsch M, Lungmus JK, Angielczyk KD. Effects of taphonomic deformation on geometric morphometric analysis of fossils: a study using the dicynodont *Diictodon feliceps* (Therapsida, Anomodontia). PeerJ. 2020 Oct 7;8:e9925.

68. Adams D, Collyer M, Kaliontzopoulou A, Sherratt E. Geomorph: Software for geometric morphometric analyses. 2016.

69. Adams DC, Collyer ML. Multivariate Phylogenetic Comparative Methods: Evaluations, Comparisons, and Recommendations. Syst Biol. 2018 Jan 1;67(1):14–31.

70. Schlager S. Morpho and Rvcg – Shape Analysis in R: R-Packages for Geometric Morphometrics, Shape Analysis and Surface Manipulations. In: Zheng G, Li S, Székely G, editors. Statistical Shape and Deformation Analysis. Academic Press; 2017. p. 217–56. Available from: https://www.sciencedirect.com/science/article/pii/B9780128104934000110

71. Yang W, Feng Q, Liu Y, Tabor N, Miggins D, Crowley JL, et al. Depositional environments and cyclo- and chronostratigraphy of uppermost Carboniferous–Lower Triassic fluvial–lacustrine deposits, southern Bogda Mountains, NW China — A terrestrial paleoclimatic record of mid-latitude NE Pangea. Glob Planet Change. 2010 Aug;73(1–2):15–113.

72. Yang W, Wan M, Crowley JL, Wang J, Luo X, Tabor NJ, et al. Paleoenvironmental and paleoclimatic and cyclo- and chrono-stratigraphy of upper Permian–Lower Triassic fluvio-lacustrine deposits in Bogda Mountains, NW China – implications for diachronous plant evolution across Permian-Triassic boundary. Earth-Sci Rev. :Forthcoming.

73. Lamm E-T. 4. Preparation and Sectioning of Specimens. Bone Histology of Fossil Tetrapods. University of California Press; 2013.

74. Holliday CM, Ridgely RC, Sedlmayr JC, Witmer LM. Cartilaginous Epiphyses in Extant Archosaurs and Their Implications for Reconstructing Limb Function in Dinosaurs. Farke AA, editor. PLoS ONE. 2010 Sep 30;5(9):e13120.

75. Griffin CT, Stefanic CM, Lessner EJ, Riegler M, Formoso K, Koeller K, et al. Assessing ontogenetic maturity in extinct saurian reptiles. Biol Rev. 2020;56.

76. de Ricqlès A. Recherches paleohistologiques sur les os longs des tetrapodes; II, Quelques observations sur la structure des os longs des theriodontes. Ann Pale´ontologie. 1969;55(1):1–51.

77. Ray S, Botha J, Chinsamy A. Bone histology and growth patterns of some nonmammalian therapsids. J Vertebr Paleontol. 2004 Sep 10;24(3):634–48.

78. Ray S, Bandyopadhyay S, Bhawal D. Growth patterns as deduced from bone microstructure of some selected neotherapsids with special emphasis on dicynodonts: Phylogenetic implications. Palaeoworld. 2009 Mar;18(1):53–66.

79. Ray S, Botha-Brink J. Dicynodont Growth Dynamics and Lifestyle Adaptations. In: Forerunners of Mammals. Indiana University Press; 2012. p. 29.

80. Olivier C, Houssaye A, Jalil N-E, Cubo J. First palaeohistological inference of resting metabolic rate in an extinct synapsid, *Moghreberia nmachouensis* (Therapsida: Anomodontia). Biol J Linn Soc. 2017 Jun 1;121(2):409–19.

81. Mori R, Kodaka T, Sano T, Yamagishi N, Asari M, Naito Y. Comparative Histology of the Laminar Bone between Young Calves and Foals. Cells Tissues Organs. 2003;175(1):43–50.

82. Currey JD. Differences in the Blood-Supply of Bone of Different Histological Types. J Cell Sci. 1960 Sep 1;s3-101(55):351–70.

83. Currey JD. The many adaptations of bone. J Biomech. 2003 Oct;36(10):1487–95.

84. de Margerie E, Cubo J, Castanet J. Bone typology and growth rate: testing and quantifying 'Amprino's rule' in the mallard (*Anas platyrhynchos*). C R Biol. 2002 Mar 1;325(3):221–30.

85. Urbanek A. Biotic crises in the history of Upper Silurian graptoloids: A Palaeobiological model. Hist Biol. 1993 Jul;7(1):29–50.

86. Harries PJ, Knorr PO. What does the 'Lilliput Effect' mean? Palaeogeogr Palaeoclimatol Palaeoecol. 2009 Dec;284(1–2):4–10.

87. Huttenlocker AK. Body Size Reductions in Nonmammalian Eutheriodont Therapsids (Synapsida) during the End-Permian Mass Extinction. PLOS ONE. 2014 Feb 3;9(2):e87553.

88. Huttenlocker AK, Botha-Brink J. Body size and growth patterns in the therocephalian *Moschorhinus kitchingi* (Therapsida: Eutheriodontia) before and after the end-Permian extinction in South Africa. Paleobiology. 2013;39(2):253–77.

89. Grine FE, Forster CA, Cluver MA, Georgi JA. Cranial Variability, Ontogeny, and Taxonomy of *Lystrosaurus* from the Karoo Basin of South Africa. In: Amniote Paleobiology. University of Chicago Press; 2006. p. 432–506.

90. Botha J, Smith RMH. *Lystrosaurus* species composition across the Permo–Triassic boundary in the Karoo Basin of South Africa. Lethaia. 2007;40(2):125–37.

91. Smith RMH, Botha-Brink J. Anatomy of a mass extinction: Sedimentological and taphonomic evidence for drought-induced die-offs at the Permo-Triassic boundary in the main Karoo Basin, South Africa. Palaeogeogr Palaeoclimatol Palaeoecol. 2014 Feb;396:99–118.

92. Botha J, Huttenlocker AK, Smith RMH, Prevec R, Viglietti P, Modesto SP. New geochemical and palaeontological data from the Permian-Triassic boundary in the South African Karoo Basin test the synchronicity of terrestrial and marine extinctions. Palaeogeogr Palaeoclimatol Palaeoecol. 2020 Feb;540:109467.

93. Kitching JW. The distribution of the Karroo vertebrate fauna: with special reference to certain genera and the bearing of this distribution on the zoning of the Beaufort Beds. University of the Witwatersrand; 1977. 1–131 p.

94. Kammerer CF, Viglietti PA, Hancox PJ, Butler RJ, Choiniere JN. A new kannemeyeriiform dicynodont (*Ufudocyclops mukanelai*, gen. et sp. nov.) from Subzone C of the *Cynognathus* Assemblage Zone, Triassic of South Africa, with implications for biostratigraphic correlation with other African Triassic faunas. J Vertebr Paleontol. 2019;39(2):e1596921.

95. Scotese CR, Wright N. PALEOMAP paleodigital elevation models (PaleoDEMS) for the Phanerozoic. 2018. Available from: https://www.earthbyte.org/paleodem-resource-scotese-and-wright-2018/

96. Scotese CR. An Atlas of Phanerozoic Paleogeographic Maps: The Seas Come In and the Seas Go Out. Annu Rev Earth Planet Sci. 2021 May 30;49(1):annurev-earth-081320-064052.

97. Kidder DL, Worsley TR. Causes and consequences of extreme Permo-Triassic warming to globally equable climate and relation to the Permo-Triassic extinction and recovery. Palaeogeogr Palaeoclimatol Palaeoecol. 2004 Feb;203(3–4):207–37.

98. Kiehl JT, Shields CA. Climate simulation of the latest Permian: Implications for mass extinction. Geology. 2005;33(9):757.

99. Winguth AME, Shields CA, Winguth C. Transition into a Hothouse World at the Permian–Triassic boundary—A model study. Palaeogeogr Palaeoclimatol Palaeoecol. 2015 Dec 15;440:316–27.

100. Rey K, Amiot R, Fourel F, Abdala F, Fluteau F, Jalil N-E, et al. Oxygen isotopes suggest elevated thermometabolism within multiple Permo-Triassic therapsid clades. Elife. 2017;6:e28589.

101. Winguth A, Winguth C. Precession-driven monsoon variability at the Permian–Triassic boundary — Implications for anoxia and the mass extinction. Glob Planet Change. 2013 Jun 1;105:160–70.

102. Tabor NJ, Sidor CA, Smith RMH, Nesbitt SJ, Angielczyk KD. Paleosols of the Permian-Triassic: proxies for rainfall, climate change and major changes in terrestrial tetrapod diversity. J Vertebr Paleontol. 2017 Nov 29;37(sup1):240–53.

103. Sun Y, Joachimski MM, Wignall PB, Yan C, Chen Y, Jiang H, et al. Lethally Hot Temperatures During the Early Triassic Greenhouse. Science. 2012 Oct 19;338(6105):366–70.

104. Frank TD, Fielding CR, Winguth AME, Savatic K, Tevyaw A, Winguth C, et al. Pace, magnitude, and nature of terrestrial climate change through the end-Permian extinction in southeastern Gondwana. Geology. 2021 May 19; Available from: https://doi.org/10.1130/G48795.1

105. Smith RMH. Changing fluvial environments across the Permian-Triassic boundary in the Karoo Basin, South Africa and possible causes of tetrapod extinctions. Palaeogeogr Palaeoclimatol Palaeoecol. 1995 Aug;117(1–2):81–104.

106. Ward PD. Altered River Morphology in South Africa Related to the Permian-Triassic Extinction. Science. 2000 Sep 8;289(5485):1740–3.

107. Viglietti PA, Smith RMH, Rubidge BS. Changing palaeoenvironments and tetrapod populations in the *Daptocephalus* Assemblage Zone (Karoo Basin, South Africa) indicate early onset of the Permo-Triassic mass extinction. J Afr Earth Sci. 2018 Feb;138:102–11.

108. Gastaldo RA, Kamo SL, Neveling J, Geissman JW, Looy CV, Martini AM. The base of the *Lystrosaurus* Assemblage Zone, Karoo Basin, predates the end-Permian marine

extinction. Nat Commun. 2020 Dec;11(1). Available from: http://www.nature.com/articles/s41467-020-15243-7

109. Roopnarine PD, Angielczyk KD. Community stability and selective extinction during the Permian-Triassic mass extinction. Science. 2015 Oct 2;350(6256):90–3.

110. Metcalfe I, Foster CB, Afonin SA, Nicoll RS, Mundil R, Xiaofeng W, et al. Stratigraphy, biostratigraphy and C-isotopes of the Permian–Triassic non-marine sequence at Dalongkou and Lucaogou, Xinjiang Province, China. J Asian Earth Sci. 2009 Nov;36(6):503–20.

111. Wan M, Yang W, Wang J. *Amyelon bogdense* sp. nov., a silicified gymnospermous root from the Changhsingian–Induan (?) in southern Bogda Mountains, northwestern China. Rev Palaeobot Palynol. 2019 Apr;263:12–27.

112. Wan M-L, Yang W, Wang J. *Palaeocupressinoxylon uniseriale* n. gen. n. sp., a gymnospermous wood from the upper Permian of Central Taodonggou, southern Bogda Mountains, northwestern China. Palaeoworld. 2020 Mar;29(1):117–25.

Fig1

Click here to access/download;Figure;Fig1.tif

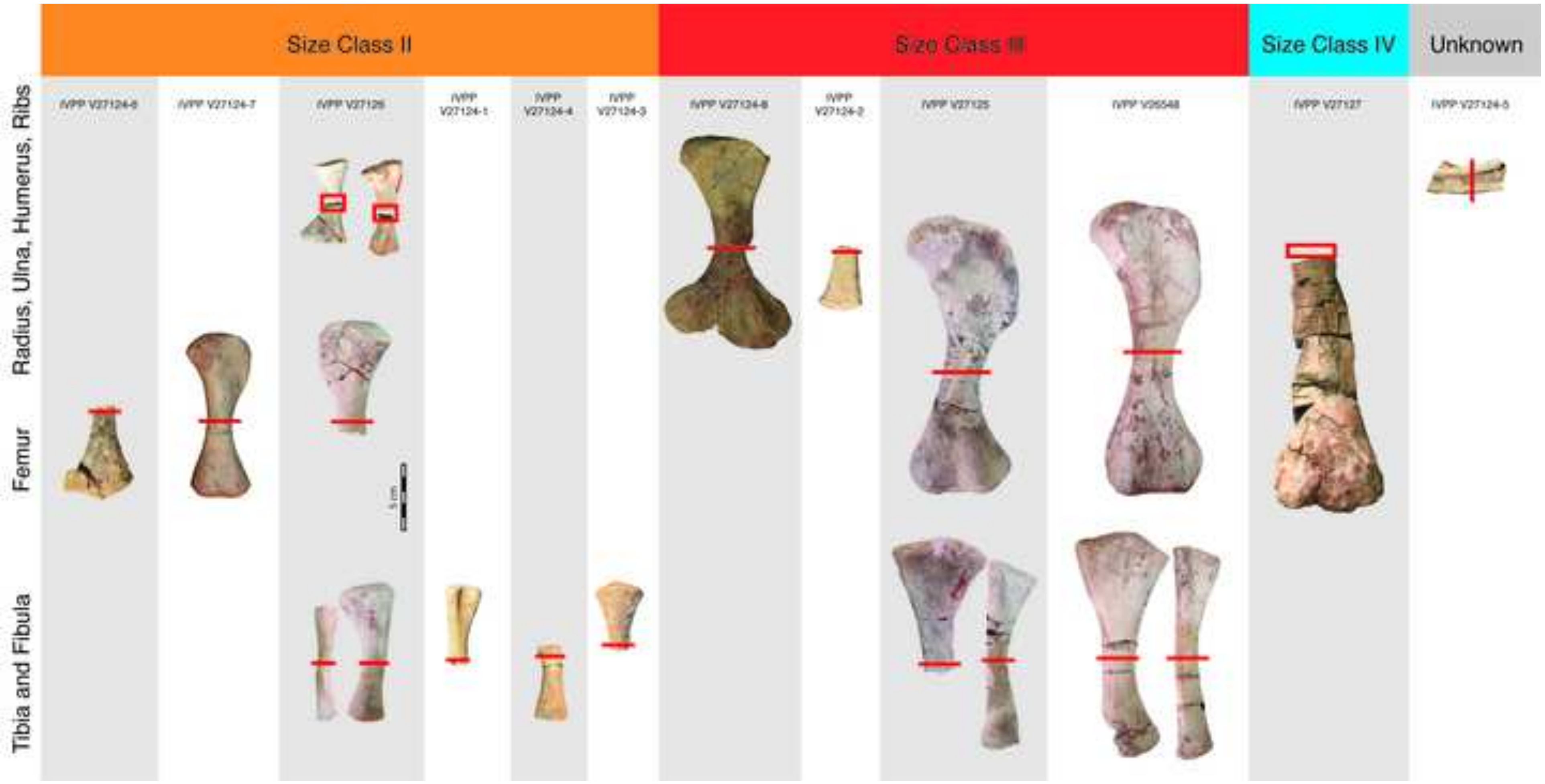

Fig2

Click here to access/download;Figure;Fig2.tiff

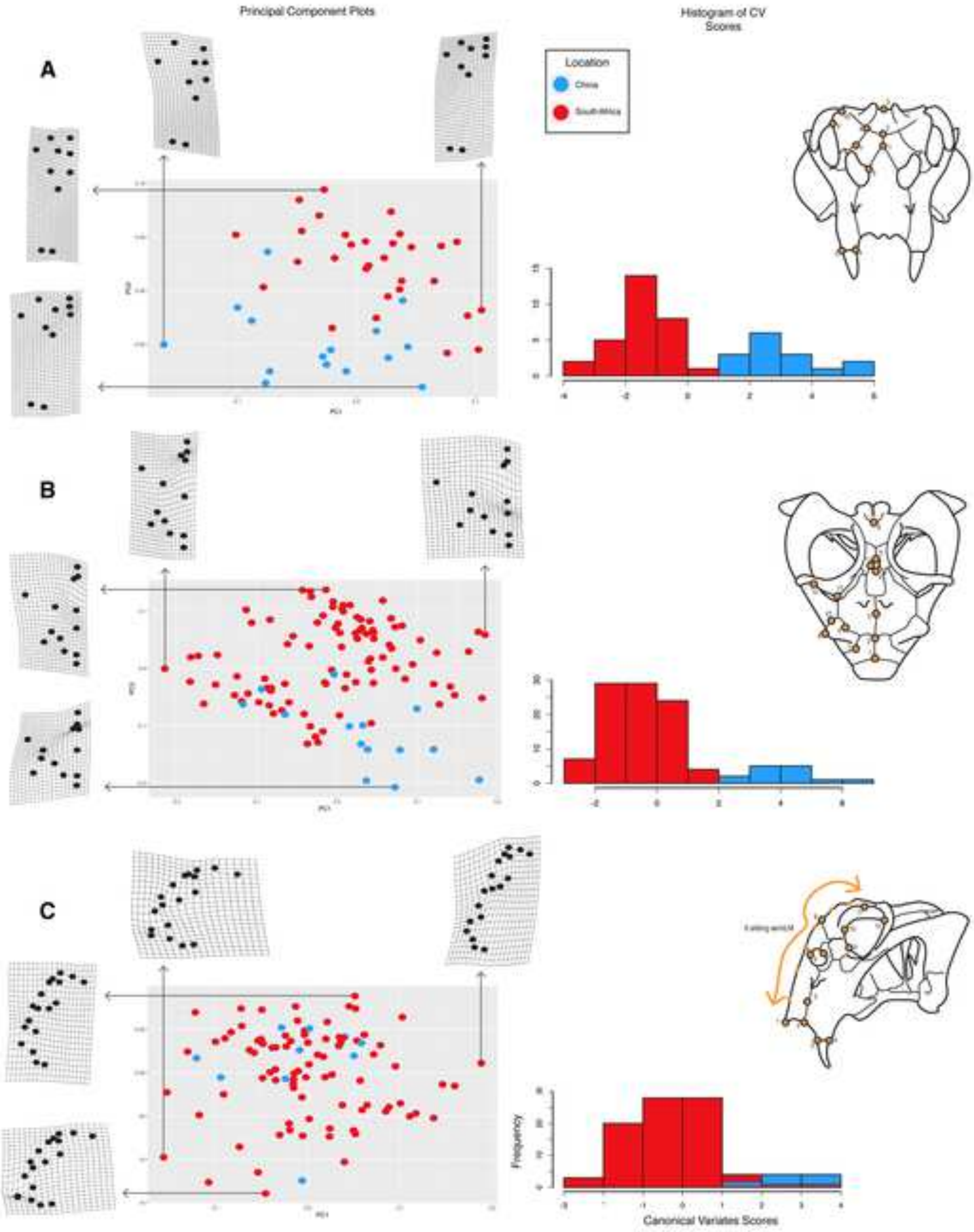

 Click here to access/download;Figure;Fig3.tiff

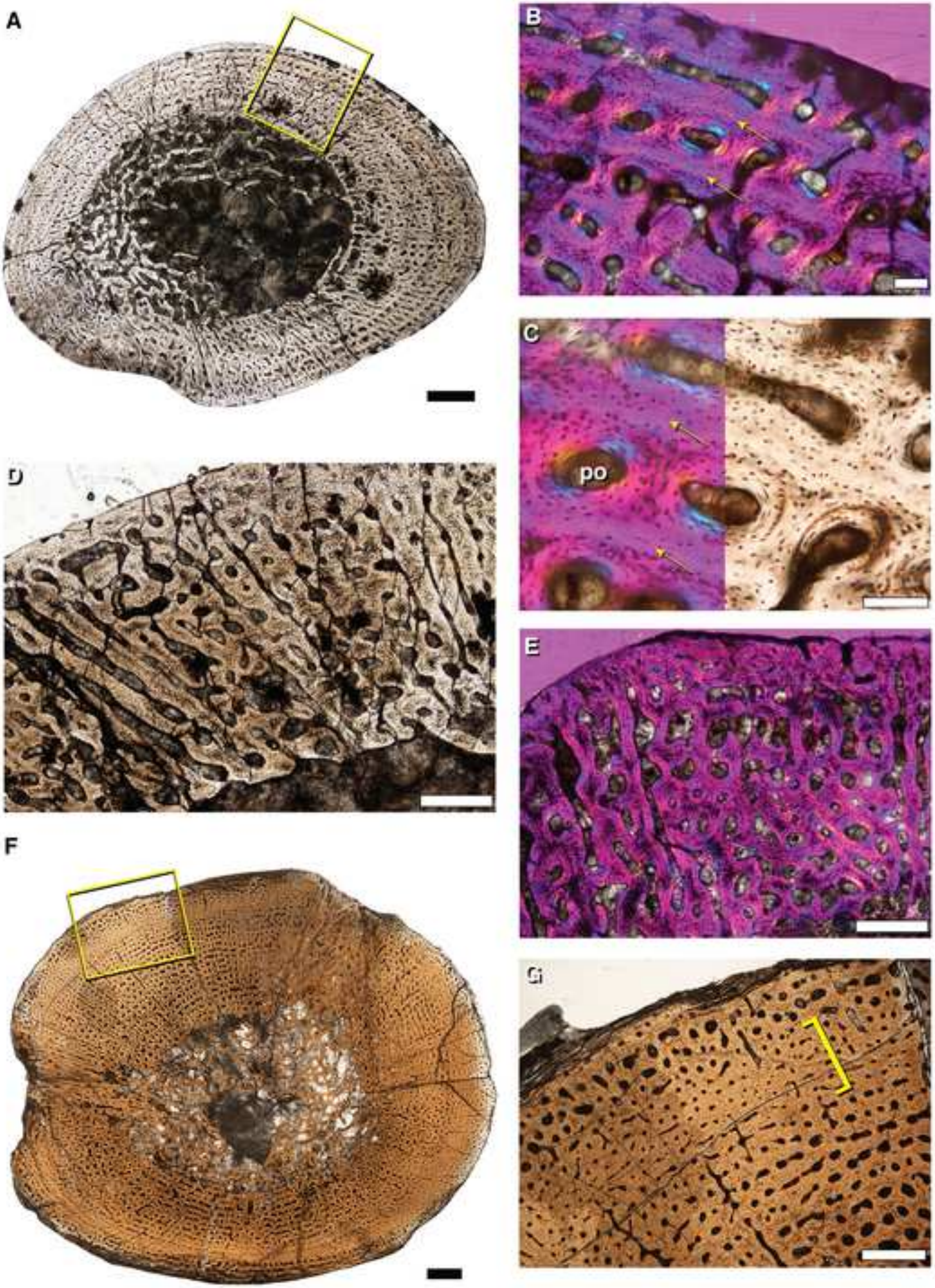

 Click here to access/download;Figure;Fig4.tiff

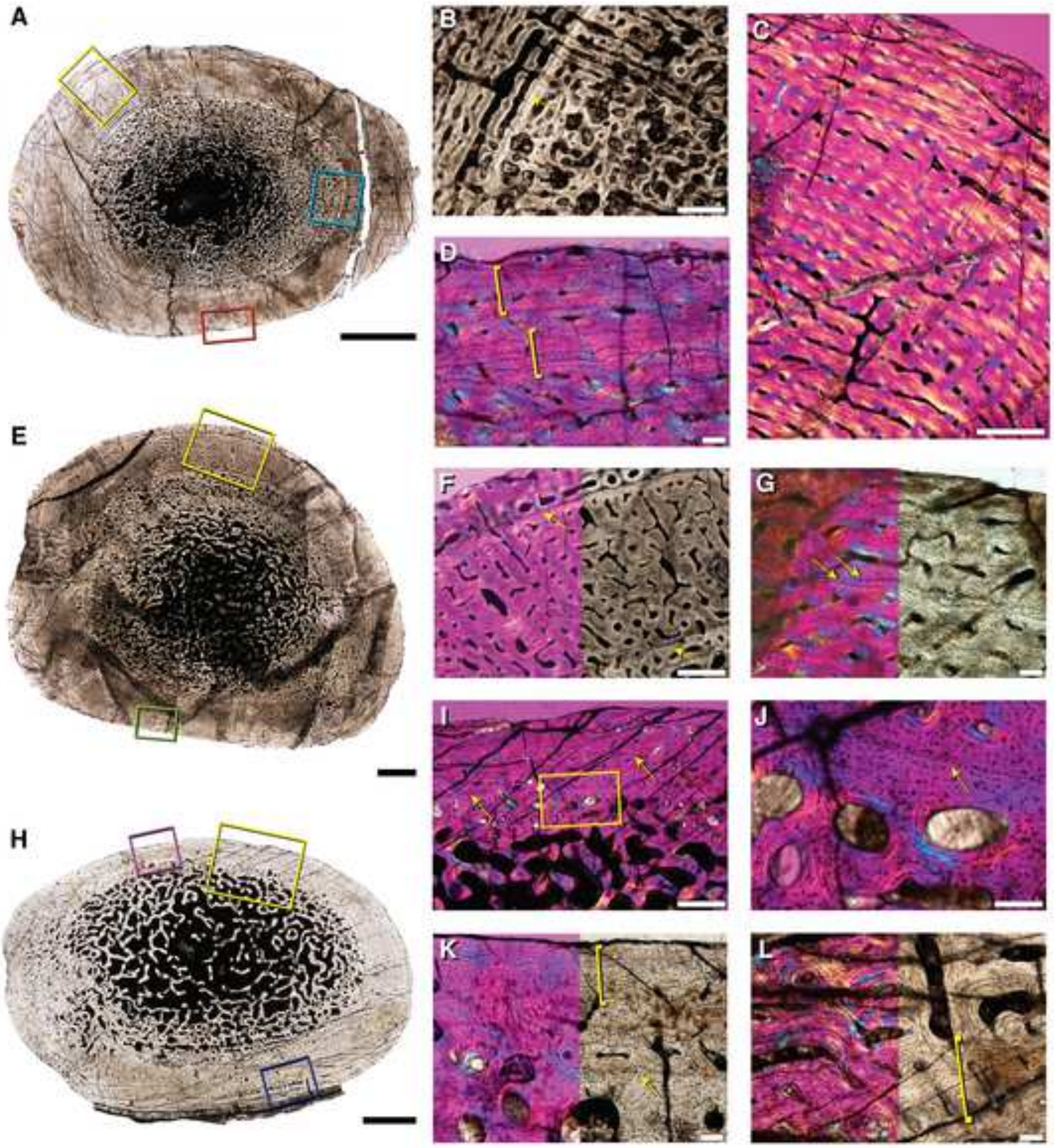

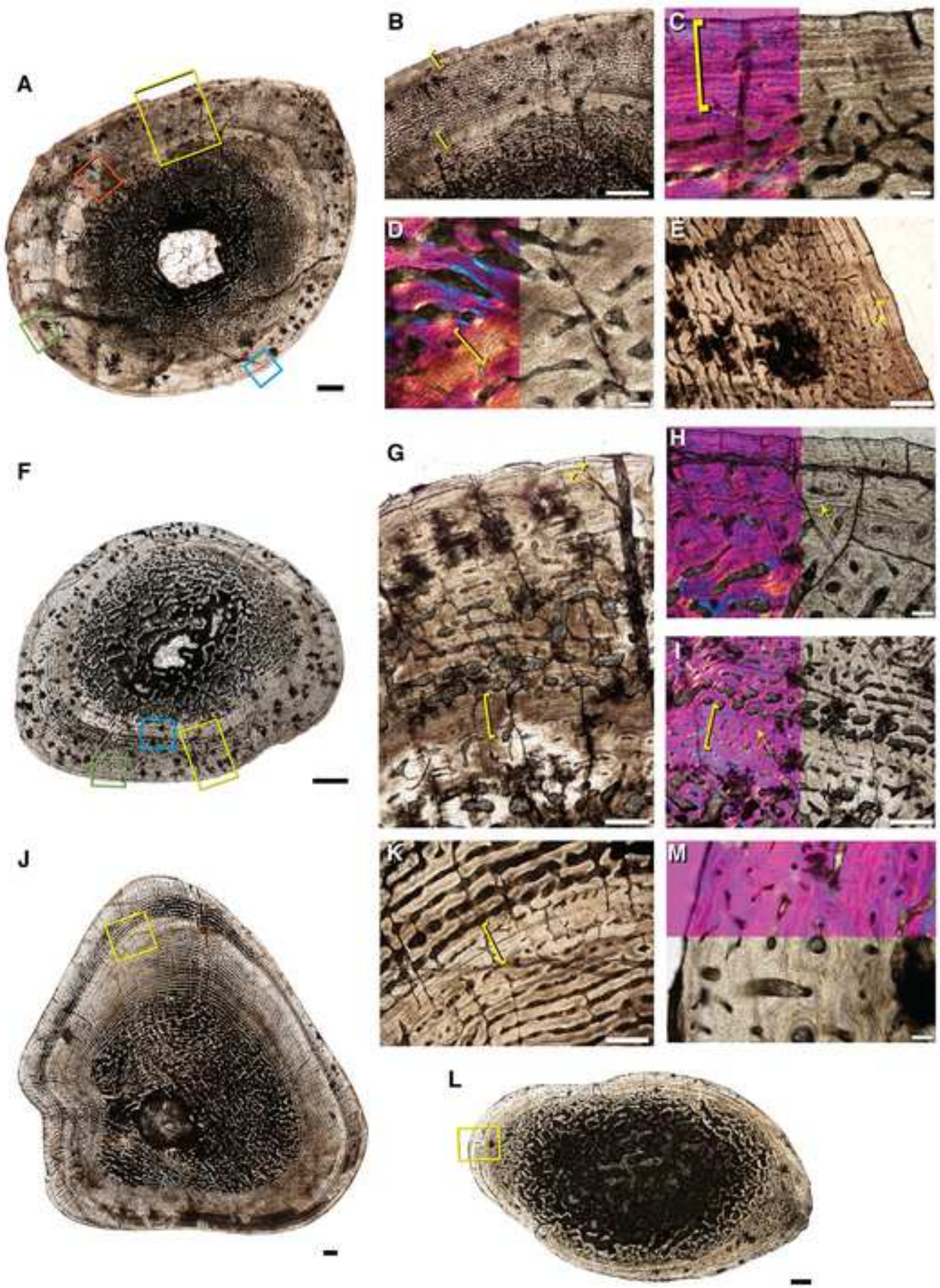
A
B
C
D
E
F
G
H
I
J
K
L
M

Fig6

Click here to access/download;Figure;Fig6.tiff

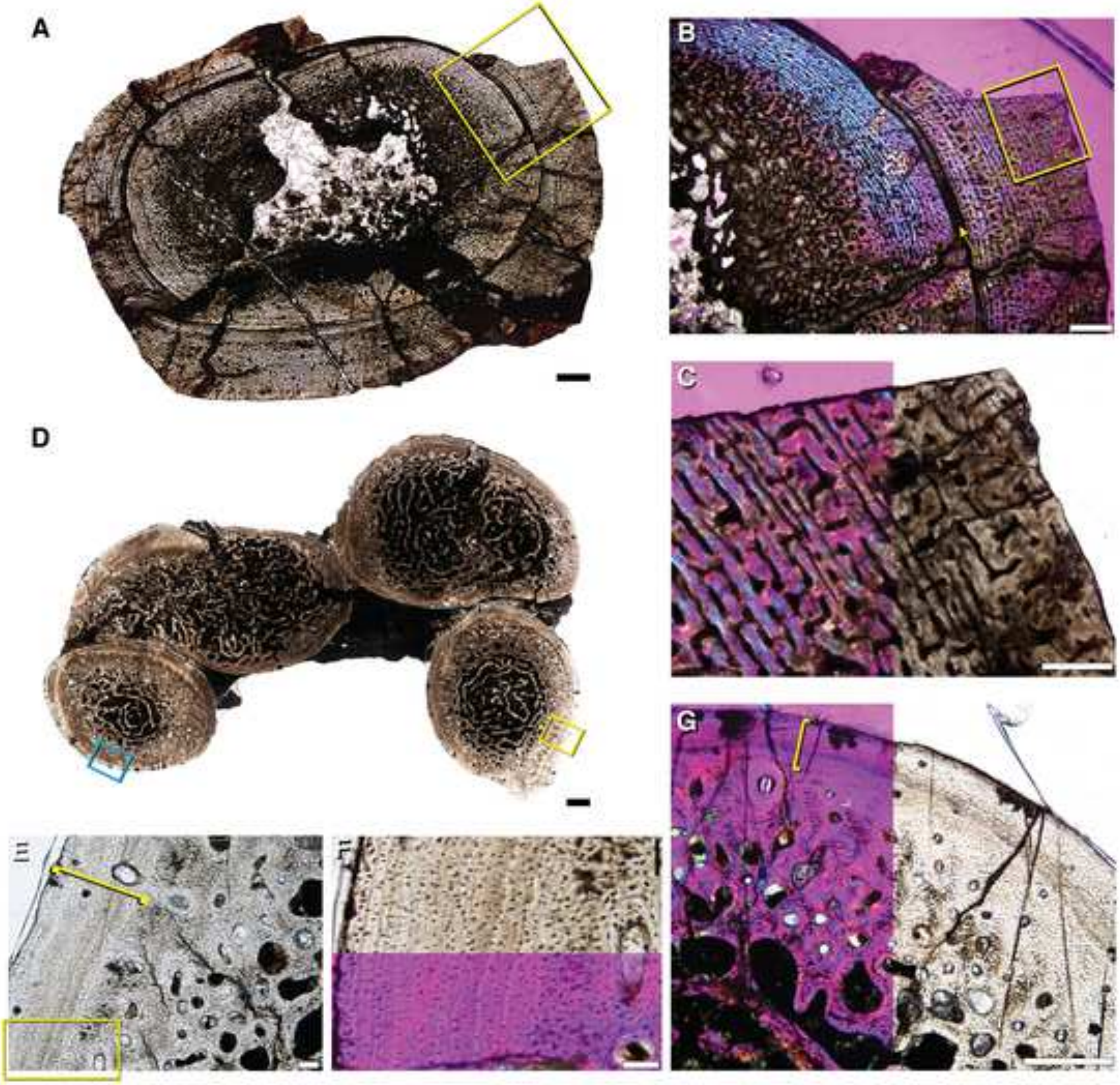

Fig7

Click here to access/download;Figure;Fig7.tiff

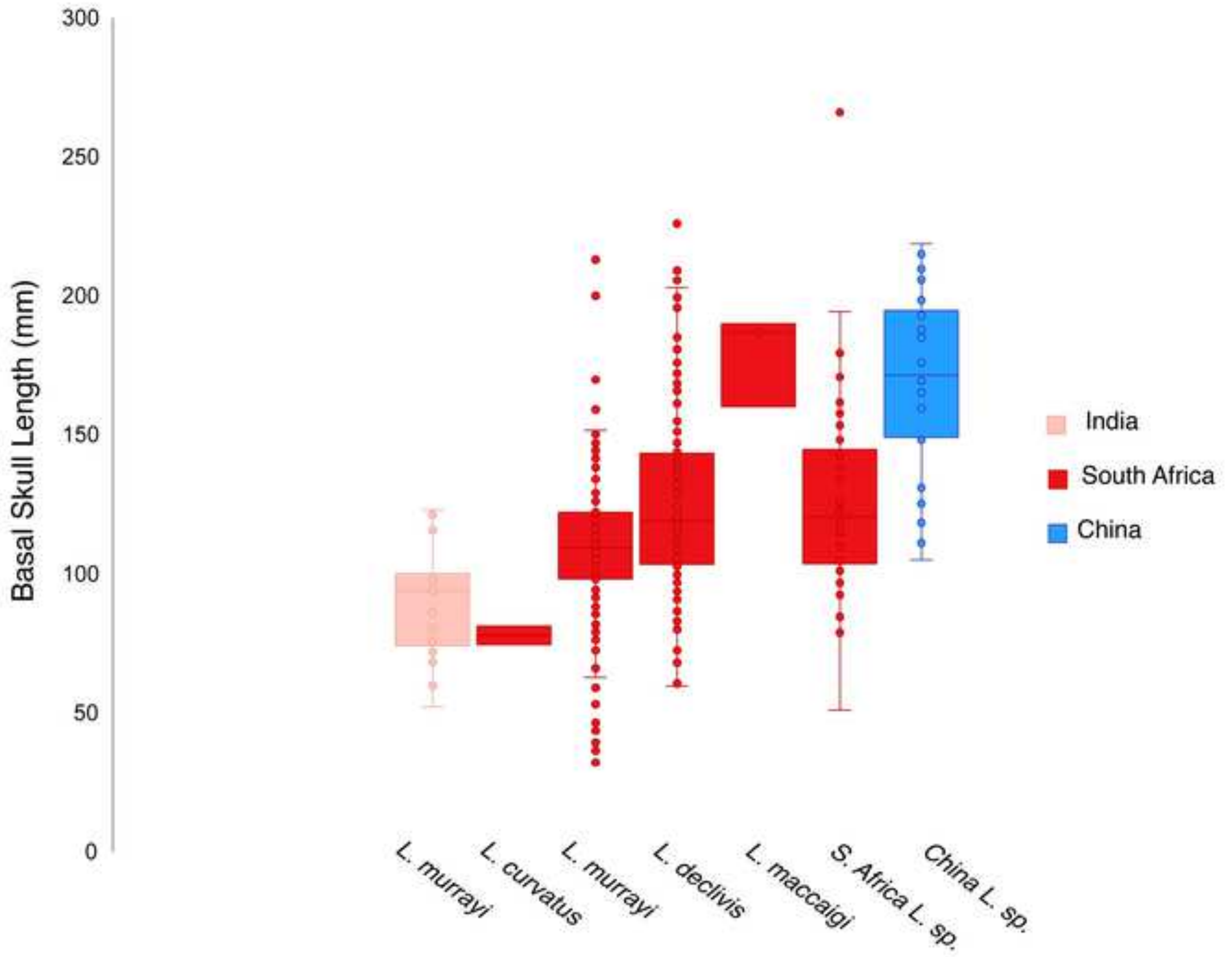

Click here to access/download;Figure;Fig8.tiff

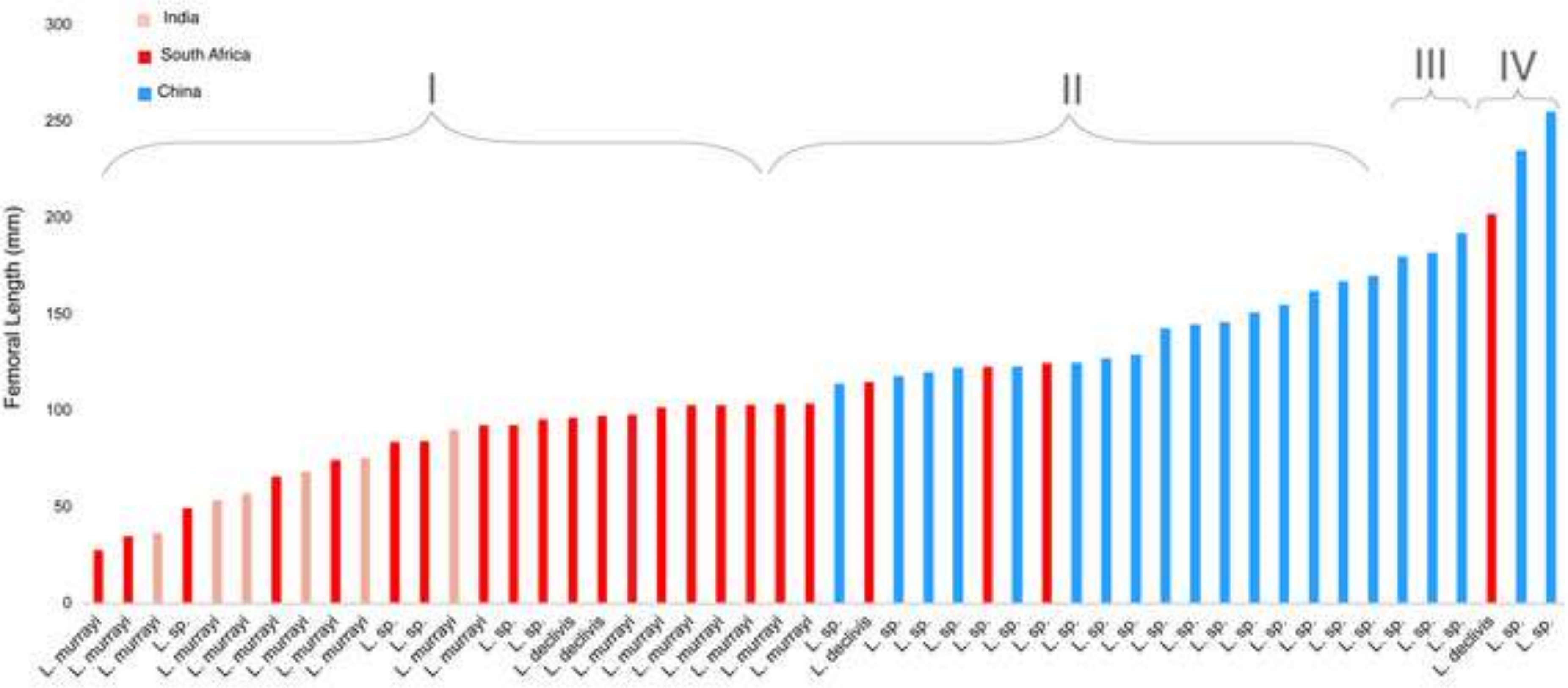

S1 File

Click here to access/download
Supporting Information
S1 File.docx

S2 Dataset

Click here to access/download
Supporting Information
S2 Supplementary Data.xlsx

Composite thin sections available on Morphobank

Click here to download Data Review URL
http://morphobank.org/permalink/?P4023
