# Supplementary material for: Living fast in the Triassic: New data on life history in Lystrosaurus (Therapsida: Dicynodontia) from northeastern Pangea
Source: PLoS One. 2021 Nov 5;16(11):e0259369. doi: 10.1371/journal.pone.0259369 (PMC8570511; doi:10.1371/journal.pone.0259369)
Supplement: Attachment — Submitted filename: PONE-D-21-20672_R1_reviewer.pdf [file pone.0259369.s006.pdf]

PLOS ONE

Living Fast in the Triassic: New data on life history in <i>Lystrosaurus</i> (Therapsida: Dicynodontia) from northeastern Pangea

--Manuscript Draft--

**Manuscript Number:**	PONE-D-21-20672R1
**Article Type:**	Research Article
**Full Title:**	Living Fast in the Triassic: New data on life history in <i>Lystrosaurus</i> (Therapsida: Dicynodontia) from northeastern Pangea
**Short Title:**	Life history in <i>Lystrosaurus</i> from northeastern Pangea
**Corresponding Author:**	Zoe T. Kulik<br>University of Washington Seattle Campus: University of Washington<br>Seattle, WA UNITED STATES
**Keywords:**	<i>Lystrosaurus</i>; bone histology; life history; lines of arrested growth; Triassic; China; Jiucaiyuan Formation
**Abstract:**	<i>Lystrosaurus</i> was one of the few tetrapods to survive the Permo-Traissic mass extinction, the most profound biotic crisis in Earth's history. The wide paleolatitudinal range and high abundance of <i>Lystrosaurus</i> during the Early Triassic provide a unique opportunity to investigate changes in growth dynamics and longevity following the mass extinction, yet most studies have focused only on species that lived in the southern hemisphere. Here, we present the long bone histology from twenty <i>Lystrosaurus</i> skeletal elements spanning a range of sizes that were collected in the Jiucaiyuan Formation of northwestern China. In addition, we compare the average body size of northern and southern Pangean Triassic-aged species and conduct cranial geometric morphometric analyses of southern and northern taxa to begin investigating whether specimens from China are likely to be taxonomically distinct from South African specimens. We demonstrate that <i>Lystrosaurus</i> from China have larger average body sizes than their southern Pangean relatives and that cranial morphologies are distinctive. The osteohistological examination revealed sustained, rapid osteogenesis punctuated by growth marks in some, but not all, immature individuals from China. We find that the osteohistology of Chinese <i>Lystrosaurus</i> shares a similar growth pattern with South African species that show sustained growth until death. However, bone growth arrests more frequently in the Chinese sample. Nevertheless, none of the long bones sampled here indicate that maximum or asymptotic size was reached, suggesting that the maximum size of <i>Lystrosaurus</i> from the Jiucaiyuan Formation remains unknown.
**Order of Authors:**	Zoe T. Kulik
	Jacqueline K. Lungmus
	Kenneth D. Angielczyk
	Christian A. Sidor
**Response to Reviewers:**	Dear Editors and Reviewers,<br><br>Thank you for your reviews and feedback. We have revised a large portion of the manuscript in response to Reviewer 1's comments and concerns and have included addition stratigraphic information and relevant discussion in response to Reviewer 2. Please find a detailed list of our changes to the text, figures, and supplemental information below.<br><br>Kind regards,<br><br>Zoe Kulik<br><br><br>Comments from the Journal<br>1. Please ensure that your manuscript meets PLOS ONE's style requirements, including those for file naming. The PLOS ONE style templates can be found at

Powered by Editorial Manager® and ProduXion Manager® from Aries Systems Corporation

https://journals.plos.org/plosone/s/file?id=wjVg/PLOSOne_formatting_sample_main_body.pdf and
https://journals.plos.org/plosone/s/file?id=ba62/PLOSOne_formatting_sample_title_authors_affiliations.pdf

Done

3. Please include captions for your Supporting Information files at the end of your manuscript, and update any in-text citations to match accordingly. Please see our Supporting Information guidelines for more information: http://journals.plos.org/plosone/s/supporting-information.

Done

Comments from Reviewer 1:

Reviewer #1: This paper focuses on the osteohistology of Lystrosaurus from the Jiucaiyuan Formation of northwestern China and thus, gives a (relatively) new perspective on Lystrosaurus life history from a northern hemisphere perspective. Lystrosaurus is an ideal study taxon for examining life history changes during mass extinction events as it survived the end-Permian mass extinction to become the most abundant vertebrate in the post-extinction ecosystem. Thus, any new data on Lystrosaurus is globally relevant. This paper is very similar to a recently published paper on Chinese Lystrosaurus osteohistology by Han et al. 2020 even finding very similar conclusions, so the value in this research is not necessarily novel data, but increasing the sample size of Chinese Lystrosaurus to support Han et al.'s 2020 findings. The authors do, however, dispute some of the details of Han et al.'s 2020 paper (which I partially agree with - they may have misidentified a few growth marks, especially in the youngest individuals, but they are correct for the most part) and the authors in this paper provide a very good dataset on the basal skull length of Lystrosaurus skulls from China.

The paper is very well written and detailed and although very similar to the Han et al. 2020 paper, it does increase the sample size of Chinese Lystrosaurus and so should be published once the osteohistological details have been sorted out. There are some issues with identifications and descriptions that need to be fixed or clarified before the paper can be ready for publication.

I have provided numerous comments on the pdf itself, but there are some issues that need to be explained here and corrected in the manuscript.

1. Despite Prondvai et al. 2014 updating bone tissue terminology very few paleohistologists accepted the change in terminology (including myself) until the recent publication of a new osteohistology textbook by Buffrenil et al. 2021. This book finally accepts that the osteohistology terminology should be updated and more correct definitions be attributed to bone tissue types from a developmental perspective. This gives more meaning to the descriptions. The book is very new and only became available during the submission of this paper so it is unlikely the authors have seen it or even have access to it. They are welcome to contact me as I have a pdf copy that I can send them, but this and the Prondvai reference really should be used to describe the bone tissues. I'm not judging, I've been remiss myself, but we need to start using the correct terminology now and this will correct or update both the Botha 2020 paper and the Han et al. 2020 paper on what Lystrosaurus bone histology really looks like. I'm sending you a table of definitions (see attachment) that will help you to identify the bone tissue types in the various ontogenetic stages. I can see certain aspects of the extracellular matrix in the high magnification images but neither the figures nor the images on Morphobank are high enough resolution for me to confirm static or dynamic osteogenesis – and you need this to decide what bone tissue type you are looking at. The term fibrolamellar bone is no longer used. Instead bone tissue types have been replaced with woven bone, parallel-fibered bone and the woven-parallel complex. Typically only embryonic or neonates have pure woven bone, which is SO (static osteogenesis) derived. Thus, what you are looking at in the Lystrosaurus bone tissues is the woven-parallel complex, which consists of both SO and DO (dynamic

osteogenesis) derived bone tissues. This is where the PFB (parallel-fibered bone) as the lamellae around the primary osteons and within the extracellular matrix is deposited on a scaffold of woven bone. The term fibrolamellar complex (FLC) has been retained to describe tissues that are predominantly woven, but have the PFB component in the form of primary osteons. This is highly vascularized fast growing tissue that you will only see in young juveniles, but falls under the woven-parallel complex. Thus you can still use FLC if you explain what you mean, but from the images I am unsure if you do have FLC in any of your age classes. You may have had it in Age Class I (but you don't have this), but I am unable to see enough of the osteocytes to see if you have predominantly SO or an equal amount of SO-DO in your Age Class II. In order to prove FLC you will need a very high magnification image of woven bone and it's possible it's present in the inner cortex of the bones in Figure 3, but unfortunately Fig 3B and C do not provide enough of the cortex to know if it's FLC or WPC. These two images show WPC, for example in Fig 3C just under the letter C there is a nodule of woven bone, but unless I saw more of the cortex at this magnification, I cannot tell from your images if you have FLC or WPC. To prove FLC you need to replace Fig 3C with something showing more woven bone. I realise you had a point to 3C, so it might be more useful to replace Fig 3B with tissues showing a higher degree of SO. This follows through all of your age classes – you are not likely to find FLC in your older individuals, even though they are still far from being fully grown, it is likely that you have WPC as the dominant bone tissue type. This is the case for the South African and Indian Lystrosaurus. If it is difficult to redo some of the figures you could add several images to your Morphobank data – high magnification images. The reason why it's important to get this description right is because, for example, you mention parallel-fibered bone in one of your elements – for an overall change from WPC to PFB, which has not been found in any Lystrosaurus specimens studied to date, would imply that you've found an individual that is showing the possible onset of reproductive maturity, or at least that it's a late subadult, which is older than any Lystrosaurus specimen found to date. I do not see your parallel-fibered bone in the ulna, it still looks like WPC but with a much lower degree of the woven component, i.e. there is still static osteogenesis. The vascular canals in Figure 5M still looks like WPC and not PFB. Thus, if you're right it has far reaching implications, but you have not shown enough evidence for a change from WPC to PFB. Another reason why it's important to be absolutely sure you're dealing with FLC (a subcategory of WPC) is that it indicates very high growth rates compared to WPC (although WPC still indicates high growth rates, depending on the proportion of SO). So if you still have FLC in your older age classes it's suggesting that these animals were growing faster than Lystrosaurus in other parts of the world or were younger – so again it's important to differentiate.

Prondvai, E., K. H. Stein, A. de Ricqles and J. Cubo. 2014. Development-based revision of bone tissue classification: the importance of semantics for science. Biological Journal of the Linnean Society 112, 799–816.

Buffrénil, de V., A. de Ricqlès, L. Zylberberg and K. Padian (eds.), Comparative skeletal histology and paleohistology, CRC Press, Taylor and Francis, Boca Raton, 824 pp. (the chapter by Buffrenil and Quilhac on bone tissue types).

I think the paper would benefit from some polarized light images showing the woven look.

2. A very big issue are the vascular orientations. To clear something up that I see in a couple of places in the text, the phrase – laminar to fibrolamellar bone – is incorrect. Laminar bone is a kind of vascular orientation found within the fibrolamellar complex (previously known as FLB), so it IS FLC. I have seen descriptions written like this in papers before, but it is incorrect. The old Francillon et al 1990 book of definitions or the latest osteohistology book by Buffrenil et al 2021 gives you all the definitions of various vascular orientations within the woven-parallel complex. According to the images shown in this paper the younger individuals contain predominantly longitudinally-oriented primary osteons with perhaps some short anastomoses (not enough to be reticular), and as the individuals become older a predominance of plexiform or laminar bone appears. This is important because – although there is an overlap – the rule of thumb is that longitudinal canals indicate faster growth rates than laminar bone – and this would make sense as the older individuals become more laminar. There is also the

hypothesis – based on extant studies – that laminar bone is stronger, which you would expect in the older individuals. Most of the vascular orientations for each bone are incorrect and this needs to be corrected. I won't give you definitions to all the vascular orientations here.

3. The first definition of a growth mark is described as a LAG and annuli are not mentioned until they are described in the description part of the paper, with no definition. Growth marks include both annuli and LAGs (and even changes in vascular orientation), both annuli and LAGs need to be defined when they are first introduced as the bones contain both annuli and LAGs.

4. Lastly I would not suggest you identify the peripheral regions as an EFS in the places mentioned - you can mention it as a possibility but, particularly if you are basing this on ribs – or at least say it is just as likely (more than likely really) that you're seeing a temporary decrease or cessation in growth. As one is generally dealing with a rib fragment, one doesn't even know from where along the rib the section is taken, so the histology can change along the bone. You might tend to find more growth marks in ribs, but you really do not know if you're dealing with an EFS or not – and you make this case in your discussion – that you haven't found any senescent individuals yet. So I would suggest that you represent these regions as temporary changes in growth, but say that it's not impossible they represent an EFS (so change it around, because it is highly likely you are not looking at an EFS).

We have read the suggested publications and chapters from the recent textbook and have added a Bone Histology Terminology section to the Materials and Methods section to add additional information relevant to the recent changes in terminology. We have also updated the histological descriptions throughout and added high magnification images to Figs 3, 5, and 6 to address the majority of Reviewer 1's questions and comments. Details of our changes are explained below.

Line 79: annuli also represent annual changes in growth and you need to include them here too, even if your samples only show LAGs, the SA samples also have annuli, so you need to explain to the reader that you are talking about growth marks (which includes LAGs and annuli)

The Osteohistological Perspectives on Life History section has been expanded to include descriptions and definitions for both annuli and LAGs.

Line 92: you need to refer to growth marks (general term) here - not just LAGs, as it's the scarcity of annuli AND LAGs. Here you are only referring to one type of growth mark, annuli are still deemed annual

Corrected for clarity

Table 1 Column 5: this is the first time you mention growth marks - you haven't defined this in the text. This is why you need to define growth marks - not just LAGs when you first mention them

Updated definitions in Osteohistological Perspectives on Life History section

Line 323: how did you measure this? This is important for comparisons as you will notice that Fig 3F, all in Fig 4, Fig 5 and fig 6 have a gradual transition between medullary cavity and compact cortex. One needs to be consistent in defining where the edge of the medullary cavity is, which is not possible in Lystrosaurus (juding it by eye). You need to explain exactly how you measured these sections - from open medullary cavity, from where there was 50% more bone than cavity space, from where you deemed the cortex to be compact (which can be subjective). You need to show a diagram, at least, if you going to do it by eye. Otherwise, in order to do it properly you need to use ImageJ or Bone Profiler to get K and thus the cortical thickness

Based on your recommendation, we have replaced the bone wall thickness measurements with cortical thickness (K). This resulted in some updated measurements and corrections of clerical errors to Table 1. We attempted to use BoneJ to determine the relative porosity in the gradual transition of trabecular

Powered by Editorial Manager® and ProduXion Manager® from Aries Systems Corporation

medullary infilling to cortical bone but dark minerals that infilled the medullary and vascular spaces resulted in unreliable values. We proceeded to use a visual estimation of the transition to compact cortical bone and include two supplemental figures that illustrate where our measurements were taken (S1 Supplementary Information SF5 and SF6).

Line 326: I will give you a detailed explanation in my overall comments as to why I've changed the details here to reflect more up to date terminology

Thank you for these detailed comments.

Line 326: you're mixing up vascular canal orientation - laminar is a type of vascular orientation, you can have laminar or plexiform bone but you cannot have laminar bone with a plexiform orientation

Edited for clarity

Line 330: this is why you need to define growth marks earlier - this is the first time you mention annuli and you don't define what they are

Updated to include both, see above

Line 357: you need to prove that the extracellcular matrix has a woven component. Looks for areas between the osteons - outside of the PFB lamellae that have bunched, large rounded osteocyte lacunae. They will look like nodules or bunches. You need to show a high magnification image of these osteocytes to prove this is not PFB. you could use 3C and point to the area just below the C - otherwise in B at the lower left corner there is some woven bone, otherwise you can take a new image

Added text labels to Fig 3B and replaced one of the images with a high-magnification image illustrating woven-fibered bone in an associated fibula (Fig 3E).

Line 361: rephrase - an annulus is not a growth arrest, but a growth decrease, you can say growth cycles

Rephrased

Line 363: none of these elements are showing laminar or plexiform tissue (you see it in the older individuals but not here), here the dominant vascularization is longitudinally-oriented primary osteons in circumferential rows, with some short anastomoses - but they're not laminar or plexiform - redo the vascularization here

Done

Line 365: you need to say this is the yellow square in A.

Done

Line 365: be careful here - your arrows aren't actually pointing to woven bone. I understand what you're meaning here and you are correct, but the arrows are just pointing to areas where the osteocyte lacunae are not preserved - and may be woven or parallel-fibred. In 3C there is a woven patch just under the letter C in the image - but your arrows are pointing to something we do not know. A woven-parallel complex has both woven and parallel components

Edited figure caption and accompanying text. We refer to these lines as bright lines in the text and have updated the figure caption for clarity.

Line 369: no same as D - this is not plexiform

This image has been replaced to show a high magnification image of woven-fibered bone matrix.

Line 381: no - see my comments in your Figure 3 caption. Yes you are seeing fast

growth but this is a woven-fibered complex, and there is no plexiform bone here

Edited

Line 385: This band seems to run around most of the cortex and is likely a temporary decrease in growth rate (and you need to introduce the possibility that it could be annual). Growth marks needn't be narrow annuli of PFB of LB, but they are can also be represented by changes in vascular canal orientation or a temporary decrease in vascular size - this has to mean a temporary decrease in growth rate

With respect to the smaller vascular canals in the proximal tibia, we have edited the text to refer to this temporary decrease in canal sizes as a temporary shift in growth rate. We do not interpret the differences in vascular canal size or orientations as temporary decreases in growth rate in various quadrants of the fibula and maintain that the different tissue textures seen in Fig 3A are not indicative of shifting bone growth rates in annual or cyclical patterns.

Line 388: and what is the woven versus parallel-fibered component - is there more PFB in the organized region and more woven bone in the less organized region, or does the proportion remain the same and it's only the vascular canal orientation that changes? I can't see from the Figure

Edited to add a description of the proportion of woven-fibered bone in this element.

Line 393: you need to add here why they shouldn't be mistaken for annuli either - i.e. if you don't see a cement line, why shouldn't you call them annuli? so you need to point out that these bright lines do not contain bands of slower forming tissues like parallel-fibered or lamellar bone.

Good point. We have added more to the description.

Line 393: not reticular - you need to check your definitions, I'll explain in the overall comments - Your Stage II mostly comprises longitudinally-oriented primary osteons in circumferential rows - Fig 3A, 3F. Definitely not enough anastomoses to call this reticular and the canals are not circumferentially arranged (for laminar or plexiform). Longitudinal canals arranged circumferentially is different to canals arranged circumferentially around the bone

'Reticular' and 'plexiform' have been replaced with 'longitudinally-oriented primary osteons' throughout.

Line 407: no - laminar is a kind of vascularization that forms part of the traditional fibrolamellar complex. You cannot have laminar to fibrolamellar bone - this is comparing apples to oranges. The vascularization is not truely laminar yet like you get in Figure 5J. The canals are a mixture of sub-laminar and longitudinally-oriented primary osteons in circumferential rows

Edited for clarity. It is our preference to refer to this tissue as a mixture of longitudinal and circular canals that approaches a laminar organization.

Line 408: what bone tissue makes up the annulus? PFB or LB?

Edited to add: parallel-fibered. (None of the osteocyte lacunae are lenticular-shaped so it seems these lacunae were cut orthogonally)

Line 414: hind limb

Done

Line 414: check these orientations - you haven't been accurate about the previous descriptions and the resolution of the renders are not high enough for me to see clearly what's happening in E and H - but F and G look sub-reticular not plexiform . There are longitudinal canals at the subperiosteal surface of F and I and L

We have updated the vascular orientation to reflect differences in each of the 3 elements, rather than try to combine the differences in one sentence.

Line 420: then it's probably the PFB part of the WPC where woven bone is becoming discontinuous

Edited to replace lamellar with parallel-fibered bone.

Line 425: This definitely looks like a LAG in the inner yellow bracket - how far does it go? I cannot see if the lines are cement lines or lamellae - but either way if they go most of the way around the cortex they are likely growth marks and should be pointed out in this image

Updated figure caption and accompanying text. The thickness of the avascular PFB region is pretty variable and the subperiosteal edge is not complete around the entire section, but the parallel-fibered tissue goes most of the way around the cortex and is certainly a growth mark.

Line 427: it's a double LAG - there's no bone tissue between them and they likely represent one season

Agreed.

Line 429: The image isn't quite high resolution enough - but it looks like what you're seeing is the PFB component of the WPC increasing towards the outer cortex. As these are older individuals you should see a progressive decrease in the amount of woven bone and an increase in PFB towards the outer cortex. If that outer region in K goes around the whole cortex then it's likely a growth mark (annulus)

Yes, there is a shift towards more organized parallel-fibered bone in some regions of the outermost cortex, but it is likely not an annulus. In the vascularized regions (4L), the mineralized lines wrap around primary osteons rather than cross-cutting, so we are interpreting these lines as the mineralizing front of periosteally deposited bone.

Line 450: you need a higher magnification of Fig 5I showing the "multiple stacked LAGs". The current image shows a cycle from too low magnification so one cannot see that there are LAGs - from this it looks like an annulus. If you zoom in on your yellow bracket more you can show them properly

Edited to refer to growth mark as an annulus.

Line 454: no take this out - you've just said in the next sentence what it most likely represents. These animals are no way near fully grown

Done

Line 456: exactly

Thank you

Line 472: now this is proper laminar vascularization - do you see how the canals are more connected to one another circumferentially compared to the younger elements where the vascular canals are more longitudinal with less connections?

Agreed

Line 480: are you absolutely sure there isn't one closer to the medullary cavity? You don't show a close up so I can't tell from the overall render, it's either just a change in colour or it could be an annulus - just double check

It is just a color change.

Line 482: laminar bone IS fibrolamellar bone. Yes it's laminar but that refers to the

vascularization. You would call a woven-parallel complex with laminar vascularization. I'll explain in my overall review

Edited

Line 483: no - they are laminar, just like you said

Edited

Line 492: Are you absolutely sure you are only seeing dynamic osteogenesis and there is no static osteogenesis? You need to be really sure about this before you call it PFB - this has huge implications, because none of your other elements show this and neither do the South African samples, so if you're right you seeing the beginning of what is probably reproductive maturity - but you have to be absolutely sure - this is very important

We have updated the figures to show a region in the outer cortex that reflects the overall tissue type that is largely parallel-fibered but has small areas of woven-fibered bone in the ulna. The osteocyte lacunae are less densely packed than in the hind limb elements but do show areas of static osteogenesis.

Line 496: no but if you're seeing an overall change from the woven-parallel complex to parallel-fibered bone then you could be seeing reproductive maturity. But the amount of woven bone decrease dramatically with age, but if there are still patches of it, then it's not PFB like you've said above. I'm afraid I cannot see the osteocytes well enough in M to help you here, and you'd need to check around the whole of the outer cortex

The change is not dramatic enough to suggest maturity, rather, there is just less woven-fibered bone

Line 501: This bone shows continued rapid growth but is bigger and presumably older than the ulna in Size Class III - which is why I'm hesitant to call that PFB in the ulna. You need to check this as it has important repercussions. If you're right then it suggests a decoupling of size and reproductive maturity. It is more likely that the ulna is woven-parallel all the way to the edge

Edited above (see line 492)

Line 503: OK - so this makes no sense. The inner cortex represents younger ontogeny and shouldn't have been growing slower than the outer cortex which represents the older ontogeny. This all looks like woven-parallel complex, where you'll have varying degrees of woven versus parallel-fibred bone.

The birefringence under cross-polarized light was really difficult to diagnose. After looking more closely at the osteocyte lacunae, we see woven- and parallel-fibered bone throughout the middle and outer cortex. There seems to be more parallel-fibered bone in the outer cortex compared to the deep and middle cortex, but not an overall shift from one tissue type to another.

Line 511: no it doesn't - and if it's laminar vascularization this is not going to be PFB but rather WPC

Corrected, see above

Line 515: you don't know this - as you've seen, a thick slower growing region at the sub-periosteal surface is likely a seasonal annulus. Yes it's not impossible it's an EFS, but if you nor Han nor myself have ever found fully grown individuals of Lystrosaurus - what is more likely? That this individual represents a fully grown animal, or that it represents a temporary decrease in growth rate? You need to put both possibilities out there and the latter is far more likely

We have removed references to an EFS and have added to the description to explain the uncertainty raised by your comments.

Powered by Editorial Manager® and ProduXion Manager® from Aries Systems Corporation

Line 517: same comment - you don't know this is an EFS

We have removed references to an EFS.

Line 526: I don't see a stack of LAGs - I see a region of PFB that may or may not represent an EFS - you really need to rather think of the more likely possibility (as explained above) that these bones do not represent fully grown individuals. The only way we will find definitive evidence of fully grown Lystrosaurus is if we find it in the limb bones. I really would not base this on the ribs, as I've seen many ribs like this where the associated limb bones show that the animal is still growing

Perhaps the emphasis was lost here. We agree that ribs alone, and especially fragmentary ones, do not indicate whether an animal is fully grown since ribs grow at different rates than limbs. We have edited the description to explain the uncertainty raised by your comment.

Line 534: I disagree with your vascular orientations - see earlier comments

Edited throughout

Line 537: I would call this more laminar

Edited

Line 558: no - an annulus cannot consist of stacked LAGs. I was wondering what you were meaning by stacked LAGs in the description. There's no high enough magnification for me to confirm - but what I think youré actually seeing is an annulus consisting of lamellar tissue - and the "stacked LAGs"are the individual lamellae. You can have an annulus and a LAG together - where the annulus occurs before or after the LAG - but you cannot have an annulus consisting of multiple LAGs. If you are indeed seeing LAGs and not lamellae, you need to show a high magnification image of this, so far I haven't seen any evidence of this

Edited throughout

Line 558: avascular what? tissue? lamellar tissue? parallel-fibered tissue? It can be avascular but what tissue type is it?

Edited to add: parallel-fibered to lamellar tissue

Line 568: you need to cite Buffrenil et al 2021 for this - they give a detailed account (first given by de Ricqles) regarding the "unit". Woven bone is deposited first as a scaffold on which parallel-fibered bone (in the form of primary osteons) is deposited. So it's not really that bone is deposited first and then woven bone is deposited - it's more the other way around, this is why you need this book. You can also look at Prondvai et al 2014

The type of bone deposition we are referring to here is the accretion of periosteal bone, rather than the formation of primary osteons. Currey (1960) and others (e.g., Mori et al., Cells Tissues Organs. 2003) describe laminar bone formation where each subsequent circumferential layer of bone (made up of a woven scaffold that is later infilled with PFB or LB) is bordered by the periosteum. As the periosteum jumps peripherally when new bone is accreted, a bright line is left between each circumferential layer and represents the place at which the periosteum was located before new bone was deposited.

601: I'm not sure I understand your meaning of maximum sizes here. We don't know what the maximum size of any of these species was, so how can one say they could grow to similar MAXIMUM sizes? Please clarify

Edited to add: maximum recorded size

Line 656: no it doesn't - unless you can provide evidence that you get plants/trees that

Powered by Editorial Manager® and ProduXion Manager® from Aries Systems Corporation

take multiple years to reach maturity and do not produce growth rings? Otherwise isn't this more likely that the wood fossils represent immature plants that are less than a year old. If you are correct please provide references that show that trees can grow multiple years without laying down growth rings.

We have removed this statement based on updated reports of fossil wood with growth marks and charcoal from the study area.
Wan M-L, Yang W, Wan S, Wang J. Wildfires in the Early Triassic of northeastern Pangaea: Evidence from fossil charcoal in the Bogda Mountains, northwestern China. Palaeoworld. 2021 Jul

Line 668: agreed

No change

Line 711: italix

Done

Comments from Reviewer 2:

IVPP V 27127=19SS26 This is a specimen from Shanshan, not Turfan!
It is the only one from the upper portion of the Jiucaiyuan Formation. It should be caution to discuss it. Because it is so different from others!
Also, could IVPP V 26548 represent an adult?
Do you have the original photo of 19SS26, I check the preserved part is less than 20 cm, not 240mm (L241)?

We have updated stratigraphic information for V 27127. It does come from higher in the section which we have added to the discussion section as a possible explanation for why the bone tissue is different from some of the smaller specimens. We have an original photo with a scale bar from the field for 19SS26 and, while incomplete, it is over 25cm long. For specimen IVPP V 26548, we do not see evidence of somatic maturity (i.e., EFS) which makes it extremely unlikely that this individual was an adult.

Abbrev, for IVPP V appears first in Fig 1, anterior to L172

We don’t believe we need an explanation of the abbreviation in this figure based on the explanation currently in the text.

L182 Chang the format as IVPP V 27126.1 not – (a blank after V)
Table 1 For same specimen, you need not add letter behind it.

We changed the formatting of the specimen numbers throughout. Our preference is to keep the letter following each element from associations of the same specimen for clarity.

Fig.1 move below the paragraph begin from L 170
Change Size Distribution Figure to FIG 2. Put it after Geometric Morphometric Fig

Our preference is to keep the limb size distribution as Fig 1 since the emphasis of this paper is on the bone histology of that sample that is first introduced in the Introduction section.

L 30 ‘Formation of northwestern China’ to ‘Formation of Xinjiang, China’

Done

L62 Lystrosaurus has a low preferred temperature than other tetrapods (Liu et al., submitted)
Liu, J., Angielczyk, K.D. and Abdala, F., 2021. Permo-Triassic tetrapods and their

	climate implications. Global and Planetary Change.<br>It is almost accepted now.<br><br>This manuscript was still in review at the time we submitted the manuscript. However, a revised version has been resubmitted, and we now cite the paper and note that the unusual inferred thermal tolerances of Lystrosaurus could have contributed to its survival.<br><br>L109 northwestern to 'Xinjiang,'<br><br>Done<br><br>shows uninterrupted cortical growth, suggesting<br>336 that Lystrosaurus from the Jiucaiyuan Formation had a high intrinsic rate of growth that could<br>337 periodically arrest.<br><br>No change<br><br>L702 For Chinese names, you can list like YI Jian, LIU Jun, but all in same sequence<br><br>Done, thank you<br><br>L711 Lystrosaurus change to italicize<br><br>Done
**Additional Information:**	
**Question**	**Response**
**Financial Disclosure**<br><br>Enter a financial disclosure statement that describes the sources of funding for the work included in this submission. Review the submission guidelines for detailed requirements. View published research articles from *PLOS ONE* for specific examples.<br><br>This statement is required for submission and **will appear in the published article** if the submission is accepted. Please make sure it is accurate.	This research was supported by National Science Foundation grants EAR 1714829 (to KDA) and EAR 1713787 (to CAS), and UW Biology Department Iuvo and Walker awards (to ZTK). The funders had no role in study design, data collection and analysis, decision to publish, or preparation of the manuscript.

**Unfunded studies**<br>Enter: *The author(s) received no specific funding for this work.*<br><br>**Funded studies**<br>Enter a statement with the following details:<br>• Initials of the authors who received each award<br>• Grant numbers awarded to each author<br>• The full name of each funder<br>• URL of each funder website<br>• Did the sponsors or funders play any role in the study design, data collection and analysis, decision to publish, or preparation of the manuscript?<br>• **NO** - Include this sentence at the end of your statement: *The funders had no role in study design, data collection and analysis, decision to publish, or preparation of the manuscript.*<br>• **YES** - Specify the role(s) played.<br><br>* typeset	
**Competing Interests**<br><br>Use the instructions below to enter a competing interest statement for this submission. On behalf of all authors, disclose any competing interests that could be perceived to bias this work—acknowledging all financial support and any other relevant financial or non-financial competing interests.<br><br>This statement is required for submission and **will appear in the published article** if the submission is accepted. Please make sure it is accurate and that any funding sources listed in your Funding Information later in the submission form are also declared in your Financial Disclosure statement.<br><br>View published research articles from *PLOS ONE* for specific examples.	The authors have declared that no competing interests exist.

**NO authors have competing interests**<br><br>Enter: *The authors have declared that no competing interests exist.*<br><br>**Authors with competing interests**<br><br>Enter competing interest details beginning with this statement:<br><br>*I have read the journal's policy and the authors of this manuscript have the following competing interests: [insert competing interests here]*<br><br>* typeset	
**Ethics Statement**<br><br>Enter an ethics statement for this submission. This statement is required if the study involved:<br><br>• Human participants<br>• Human specimens or tissue<br>• Vertebrate animals or cephalopods<br>• Vertebrate embryos or tissues<br>• Field research<br><br>Write "N/A" if the submission does not require an ethics statement.<br><br>General guidance is provided below. Consult the submission guidelines for detailed instructions. **Make sure that all information entered here is included in the Methods section of the manuscript.**	N/A

Powered by Editorial Manager® and ProduXion Manager® from Aries Systems Corporation

**Format for specific study types**<br><br>**Human Subject Research (involving human participants and/or tissue)**<br>• Give the name of the institutional review board or ethics committee that approved the study<br>• Include the approval number and/or a statement indicating approval of this research<br>• Indicate the form of consent obtained (written/oral) or the reason that consent was not obtained (e.g. the data were analyzed anonymously)<br><br>**Animal Research (involving vertebrate animals, embryos or tissues)**<br>• Provide the name of the Institutional Animal Care and Use Committee (IACUC) or other relevant ethics board that reviewed the study protocol, and indicate whether they approved this research or granted a formal waiver of ethical approval<br>• Include an approval number if one was obtained<br>• If the study involved *non-human primates*, add *additional details* about animal welfare and steps taken to ameliorate suffering<br>• If anesthesia, euthanasia, or any kind of animal sacrifice is part of the study, include briefly which substances and/or methods were applied<br><br>**Field Research**<br><br>Include the following details if this study involves the collection of plant, animal, or other materials from a natural setting:<br>• Field permit number<br>• Name of the institution or relevant body that granted permission	
**Data Availability**<br><br>Authors are required to make all data underlying the findings described fully available, without restriction, and from the time of publication. PLOS allows rare exceptions to address legal and ethical concerns. See the PLOS Data Policy and FAQ for detailed information.	Yes - all data are fully available without restriction

A Data Availability Statement describing where the data can be found is required at submission. Your answers to this question constitute the Data Availability Statement and **will be published in the article**, if accepted.<br><br>**Important:** Stating 'data available on request from the author' is not sufficient. If your data are only available upon request, select 'No' for the first question and explain your exceptional situation in the text box.<br><br>Do the authors confirm that all data underlying the findings described in their manuscript are fully available without restriction?	
**Describe where the data may be found in full sentences. If you are copying our sample text, replace any instances of XXX with the appropriate details.**<br><br>• If the data are **held or will be held in a public repository**, include URLs, accession numbers or DOIs. If this information will only be available after acceptance, indicate this by ticking the box below. For example: *All XXX files are available from the XXX database (accession number(s) XXX, XXX.*).<br>• If the data are all contained **within the manuscript and/or Supporting Information files**, enter the following: *All relevant data are within the manuscript and its Supporting Information files.*<br>• If neither of these applies but you are able to provide **details of access elsewhere**, with or without limitations, please do so. For example:<br><br>*Data cannot be shared publicly because of [XXX]. Data are available from the XXX Institutional Data Access / Ethics Committee (contact via XXX) for researchers who meet the criteria for access to confidential data.*<br><br>*The data underlying the results presented in the study are available from (include the name of the third party*	All relevant data are within the manuscript and its Supporting Information files including thin section images available on Morphobank at project number P4023 http://morphobank.org/permalink/?P4023.

and contact information or URL).<br>• This text is appropriate if the data are owned by a third party and authors do not have permission to share the data.<br><br>* typeset	
Additional data availability information:	

Living Fast in the Triassic: New data on life history in *Lystrosaurus* (Therapsida: Dicynodontia) from northeastern Pangea

Short Title: Life history in *Lystrosaurus* from northeastern Pangea

Zoe T. Kulik, Jacqueline K. Lungmus, Kenneth D. Angielczyk, and Christian A. Sidor

Zoe T. Kulik [zkulik@uw.edu], Department of Biology and Burke Museum, University of Washington, Seattle, WA 98195

Jacqueline K. Lungmus [lungmusj@si.edu] Smithsonian National Museum of Natural History Washington, D.C. 20560

Kenneth D. Angielczyk [kangielczyk@fieldmuseum.org], Negaunee Integrative Research Center, Field Museum of Natural History, Chicago, IL 60605

Christian A. Sidor [casidor@uw.edu], Department of Biology and Burke Museum, University of Washington, Seattle, WA 98195

Abstract

Lystrosaurus was one of the few tetrapod genera to survive the Permo-Triassic mass extinction, the most profound biotic crisis in Earth's history. The wide paleolatitudinal range and incredible abundance of *Lystrosaurus* during the Early Triassic provides a unique opportunity to investigate changes in growth dynamics and longevity following the mass extinction, yet most studies have focused only on species that lived in the southern hemisphere. Here, we present the long bone histology from twenty *Lystrosaurus* skeletal elements spanning a range of sizes that were collected in the Jiucaiyuan Formation of northwestern China. In addition, we compare the average body size of northern and southern Pangean Triassic-aged species, and conduct cranial geometric morphometric analyses of southern and northern specimens to begin investigating whether populations from China are likely to be distinct from South African populations. We demonstrate that *Lystrosaurus* from China have larger average body sizes than their southern Pangean relatives and that the Chinese and South African populations have distinct cranial morphologies. Our osteohistological examination revealed sustained, rapid osteogenesis punctuated by growth marks, in some, but not all immature individuals from China. We find that the osteohistology of Chinese *Lystrosaurus* shares a similar growth pattern to South African species that show sustained growth until death. However, bone growth arrests more frequently in the Chinese sample. Nevertheless, each of the long bones sampled here do not indicate that maximum or asymptotic size was reached, suggesting that the maximum size of *Lystrosaurus* from the Jiucaiyuan Formation remains unknown.

Introduction

The Permo-Triassic mass extinction was the most devastating biotic crisis in Earth's history and caused communities to collapse in both the terrestrial and marine realms [1-3]. Fluctuating climates accompanied by an overall increase in global temperatures are hypothesized to have forced 70% of terrestrial vertebrate families to extinction [4-8], although plant extinctions were notably dampened [9-11]. Recovery after the Permo-Triassic extinction has been a topic of intense research, but most studies in the terrestrial realm focus on taxonomic diversity and abundance [12-14], or changes in community structure and dynamics following the extinction [6, 3, 15-18]. Few studies have examined how the extremely changed environment of the earliest Triassic impacted growth rate, life history, and longevity of surviving taxa [19-22]. Importantly, most of the studies noted above rely heavily—if not exclusively—on data from the southern hemisphere, in particular the Karoo Basin of South Africa.

Lystrosaurus, a stoutly built dicynodont therapsid that ranged in maximum skull size from approximately 16–39 cm (*L. murrayi* and *L. maccaigi* from South Africa, respectively), is one of the hallmark survivors of the Permo-Triassic extinction due to its remarkable abundance in southern Pangean deposits [18, 23, 24]. The success of *Lystrosaurus* has been variously attributed to its generalist diet of tough plant material (e.g., [25, 26]), broad habitat tolerances (e.g., [27]), a burrowing lifestyle (e.g., [28]), and a developmentally plastic growth strategy that allowed it the ability to weather extreme ecosystem instability (e.g., [29, 20, 22, 30]). These hypothesized advantages/exaptations for a post-extinction environment have been studied almost exclusively using specimens from South Africa, but *Lystrosaurus* is known from across Pangea, including Permo-Triassic deposits in Antarctica, Mongolia, Russia, China, India, and possibly

Mozambique (e.g., [31-38]). The wide paleolatitudinal range of *Lystrosaurus* offers a rare opportunity to investigate differences in lifespan, survivorship, and body size in southern and northern Pangean populations that experienced markedly different environmental conditions in the wake of the Permo-Triassic extinction (e.g., [22, 39]).

Osteohistological Perspectives on Life History

Bone histology provides a wealth of information about an extinct animal's growth and life history. Mineralized bone matrix, vascular canal spaces, and cellular spaces persist in fossilized bone, making it possible to determine the relative rate of bone deposition as well as the maturity of extinct organisms [40]. One particularly useful yet controversial histological feature in determining aspects of an extinct animal's growth are hyper-mineralized lines of arrested growth (LAGs), which appear as cement lines in the cortical bone of most vertebrates when bone deposition periodically stops in seasonal or annual cycles [41-46]. In addition to extrinsically (i.e., environmentally or seasonally) induced LAGs, growth can also arrest during periods of intense stress, such as birth, and during modulations to metabolic activity such as torpor [47, 48]. The periodicity of LAGs (i.e., the extent to which they are regularly spaced throughout the cortex of large, presumably mature individuals) helps to determine whether they represent extrinsic or intrinsic events.

Botha [22] recently conducted a detailed examination of growth and life history patterns in four species of *Lystrosaurus* across the Permian-Triassic boundary using fossils from South Africa. Using a combination of bone histology and body size data, Botha [22] found that Triassic *Lystrosaurus* individuals grew rapidly and had increased mortality at small sizes, as evidenced by the overwhelming abundance of small skulls known from Triassic rocks in the Karoo Basin.

This post-extinction change in life expectancy is also inferred from the scarcity of lines of arrested growth (LAGs) in the rapidly deposited cortical bone of Triassic specimens. In comparison, Permian individuals had larger average skull sizes and had numerous LAGs spaced throughout the cortex, suggesting prolonged periods of growth spanning multiple seasons or years [22]. Similar patterns were previously found across a wider taxonomic range of South African therapsid species by Botha-Brink et al. [20] and in lydekkerinid stereospondyls [49, 50]. The predominance of small *Lystrosaurus* (and other tetrapods) with rapidly deposited bone tissue suggests an overall shift in life history strategy across the Permian-Triassic boundary to an early onset of reproductive maturity [20, 49, 50].

Histological data for *Lystrosaurus* outside of the Karoo Basin of South Africa are more limited, but critical to understanding the relationship between latitude (and by extension climate) and life history. Bones of *Lystrosaurus* from the Early Triassic of India lack LAGs, and have small skulls, consistent with the pattern seen in South Africa [36, 51]. A preliminary report of the bone histology from *Lystrosaurus* from the Turpan-Hami Basin of China suggested that subadult individuals record multiple LAGs [39]. However, in the absence of comparative body size data, it remains unclear if multiple LAGs in the Chinese specimens indicate prolonged periods of growth (i.e., multiple cycles or seasons of growth) or environmental instability (e.g., disruptions to metabolic rate as a result of resource limitations). Here, we analyze bone histology from a size range of *Lystrosaurus* postcrania from the Jiucaiyuan Formation of northwestern China (Fig 1), as well as body size data across Triassic-aged *Lystrosaurus* assemblages, to investigate whether *Lystrosaurus* from northern Pangea had differing life histories than its southern Pangean relatives.

Figure 1. Size distribution of *Lystrosaurus* sp. postcrania from the Jiucaiyuan Formation included in the histologic analysis. Twenty skeletal elements, with gray bars indicating associations of individuals, span three size classes inferred from femoral length: Size Class II spans 47–63% maximum femoral size, Size Class III includes specimens that are ~ 70% maximum size, and Size Class III includes the largest partial femur. Scale bar = 5 cm.

Materials and Methods

Geometric Morphometric Analysis of Skull Shape

An important question to consider when comparing the bone histology of *Lystrosaurus* specimens from China and other regions is whether the same species occur in the different geographic areas. Although the initial material of *Lystrosaurus* collected in Xinjiang was referred to the South African species *L. murrayi* [52], a total of seven endemic *Lystrosaurus* species have also been described [53-57]. Colbert [32], (also see [58-60]), considered some Chinese specimens to represent *L. murrayi* and *L. curvatus*, and others to represent endemic species. More recent authors have been split over whether *Lystrosaurus* specimens from Xinjiang represent exclusively endemic species or a mixture of endemic and more cosmopolitan species, as well as the number of valid endemic species (e.g., [34, 39, 61-64]).

A comprehensive taxonomic revision of *Lystrosaurus* from Xinjiang is beyond the scope of this paper. However, to gain insight into whether Chinese *Lystrosaurus* specimens are likely to be distinct from South African specimens, we conducted a preliminary two-dimensional geometric morphometric analysis of skull shape. The skulls were analyzed in three perspectives - anterior, dorsal, and lateral – following the methodology of [65]; also see [64]. The anterior shape analysis included 45 individual specimens, 15 from China and the remaining 30 from

South Africa. The dorsal shape analysis included 107 specimens, 14 from China and 93 from South Africa. Lastly, the lateral analysis included 95 specimens, 9 from China and 86 from South Africa. Landmarks were collected using the tpsDig2ws software [66]. In the case of the anterior and dorsal views, landmarks were digitized on only the left half of the skull. The lateral view digitization included a combination of Type I and Type II landmarks (12 total) with a single curve of six semilandmarks along the outer edge of the snout between landmarks 1 and 11 (Fig 2). The anterior and dorsal views did not contain any semilandmarks, and have 10 and 13 landmarks, respectively. Because taphonomic deformation can complicate the interpretation of shape variation at low taxonomic levels in dicynodonts [67], data collection focused on specimens that appeared to show relatively little evidence of distortion. See the supplementary files for additional details of the morphometric analysis.

The coordinate data were processed and analyzed further with the Geomorph R package [68], where they underwent a generalized Procrustes superimposition. Principal components analyses were used to visualize major patterns of shape variation, and a Procrustes ANOVA was run on each dataset to assess the statistical relationship between shape and country of origin using the method of [69] and the Geomorph package in R. A canonical variates analysis was run using the CVA function of the Morpho package [70] in R to further explore the association between shape and geographic location, as well as the accuracy with which specimens could be classified using skull shape (see *Results*).

Figure 2. Results of the morphometric analysis comparing specimens of *Lystrosaurus* from China and South Africa. Principal components plots and histogram of canonical variates analysis results for the three perspectives (Anterior – A; Dorsal – B; Lateral – C). Skull drawings on the

right correspond to the orientation for each analysis. Orange dots on the specimens show placement of landmarks used in the 2D geometric morphometric analysis (see supplemental files for more details). Blue dots represent specimens from China and red dots represent specimens from South Africa. Arrows point to the warp grids showing corresponding deformation along each principal component axis. Histograms show frequency of a recovered canonical variate value and show the distinct differences between Chinese and South African specimens, with overlap only occurring in a few specimens in the lateral orientation.

Histological Sample

Twenty skeletal elements (humerus, femur, tibia, radius, ulna, fibula, and ribs; Table 1; Fig 1) of *Lystrosaurus* sp. were selected for consumptive analysis from the collections of the Institute of Vertebrate Paleontology and Paleoanthropology (IVPP). All material was recovered from approximately 30–50 meters above the base of the Jiucaiyuan Formation from the South Taodonggou area, Turpan-Hami Basin, Xinjiang Uygur Autonomous Region, China. The Jiucaiyuan Formation overlies the Guodikeng Formation lithologically (=upper Wutonggou low-order cycle), and its contact is characterized by a facies change from lacustrine to fluvial deposits [71, 72]. This facies change is interpreted as the boundary between the Permian and Triassic [72] and recent work suggests that the Jiucaiyuan Formation is late Induan-early Olenekian in age [72]. As noted above, the lack of clarity regarding Chinese *Lystrosaurus* taxonomy means that we were unable to assign species-level identifications. However, the majority of our histologic samples are associations of four individuals. Skeletal elements are differentiated by specimen numbers followed by a letter, in the case of associated individuals (e.g., IVPP V#-a, -b, etc.) The

remaining eight specimens are disarticulated elements collected from the same locality and are assigned to one specimen number IVPP V27124, differentiated by IVPP V27124-1, -2, etc. In addition to the histologic sample, 50 femora were measured from Early Triassic specimens compiled from the literature [36, 22] and from personal observations to contextualize the range of sizes in this monodominant vertebrate assemblage (S2 Supplementary Dataset). Additional body size proxies, including basal skull length (S2 Supplementary Dataset), were measured from *Lystrosaurus* specimens housed in collections at the National Museum (NMQR), the Evolutionary Studies Institute (ESI), the Iziko South African Museum (SAM), the Field Museum of Natural History (FMNH), the American Museum of Natural history (AMNH), and the Burke Museum of Natural History and Culture (UWBM). Cranial and postcranial measurements were made using Neiko digital calipers.

TABLE 1. Postcranial element length and midshaft diameter for the twenty skeletal elements of *Lystrosaurus* sp. from the Jiucaiyuan Formation that were histologically sampled. Asterisk indicates fragmentary or incomplete specimens with accompanying estimates based on complete specimens in this dataset.

	Spec. No.	Preserved Length (mm)	Estimated Length (mm)	Midshaft Diameter (mm)	Growth Marks	Size Class
**Femur**						
	IVPP V27124-7	122.38		19.23	0	II
	IVPP V27124-6	62.74*	129	16.22	0	II
	IVPP V27126a	62*	162		0	II

	IVPP V27125a	182		13.4	1	III
	IVPP V26548a	192		14.09	2	III
	IVPP V27127	240*	255	16.57	0	IV
**Tibia**						
	IVPP V27126b	92			0	II
	IVPP V27124-4	56.62*	94	15.86	0	II
	IVPP V27124-3	53.26*	98	15.71	0	II
	IVPP V26548b	148		10.47	2	III
	IVPP V27125b	148*	212	10.36	2	III
**Fibula**						
	IVPP V27124-1	46.37*	86	10.12	0	II
	IVPP V27126c	86		58.9	0	II
	IVPP V26548c	140		7.334	2	III
	IVPP V27125c	140		15.84	2-3	III
**Humerus**						
	IVPP V27124-8	151.77		23.96	1	III
**Radius**						
	IVPP V27126d	62			0	II

**Ulna**						
	IVPP V27126e	75			0	II
	IVPP V27124-2	47.32*	93	17.52	0	III
**Ribs**						
	IVPP V27124-5	58.63*	N/A	48.76 - 56.33	EFS	?

Thin Section Preparation

Thin sections were made following standard osteohistological techniques [73]. Eight of the specimens were sectioned at the University of Washington (UW); the remaining twelve were sectioned at the Institute of Vertebrate Paleontology and Paleoanthropology (IVPP). At the UW, mid-diaphyses were embedded in Epothin Epoxy/Resin 2, sectioned to a thickness of approximately 2 mm on a high-precision diamond-edged saw (Isomet 1000) and glued to glass slides using Devcon 2-Ton epoxy. Slides were ground on a Metaserv 3000 lapidary plate until the specimen was 80 µm thick or until optical clarity was reached. At the IVPP, midshafts were cut and embedded in polyester resin, sectioned into blocks using an Exact 300CP band saw, and glued to glass slides using Technovit 4000 resin. Slides were ground to optical clarity using an automated lapidary plate (Exakt 400CS). High magnification and composite images were taken using a Nikon Eclipse LV100POL microscope under plain and cross-polarized light with a lambda filter. Composite images were processed using Nikon NIS-Elements AR (version 5.20.02) imaging software. High-resolution images are available at Morphobank under project number 4023.

Ontogenetic maturity was assessed through bone tissue microstructure, articular surface texture and morphology, and size compared to other specimens of *Lystrosaurus* from the Jiucaiyuan Formation. The largest femur, estimated at 255 mm, is of comparable length to the largest known femur (*L. declivis* SAM-PK-K8038) from the Triassic of South Africa, which is 202 mm long. Therefore, we follow the size classes established by Botha [22] for southern Pangean *Lystrosaurus*, but note that our sample lacks Size Class I, or elements less than 40% maximum known size. The remainder of the elements were split into three size categories for analysis (Table 1; Fig 1). Size Class II includes the smallest and presumably most immature specimens in the current sample, ranging in femoral lengths that are approximately 120–160 mm long, tibiae and fibulae that are less than 100 mm long, and associated forelimb elements, or 47–63% of the maximum femur length observed. All but the proximal tibia fragment (IVPP V27124-3) have poorly formed joint surfaces and show pitting indicative of epiphyseal cartilage attachment typical of immature individuals [74]. By comparison, the joint surfaces of elements in the larger size classes are more robustly built and more completely ossified. The relative degree of epiphyseal ossification combined with histological features caused us to deviate slightly from Botha's [22] size classes by grouping specimens that are 40-65% maximum known size as Size Class II, rather than her 60% MKS cutoff. Our reasoning for this slight change is that the bone tissue is consistently and characteristically immature (i.e., highly vascularized plexiform to radial tissue without LAGs) in all of the associated elements that are 63% MKS. However, the incomplete tibia (IVPP V27124-3, estimated length of 94 mm) that is estimated to be within Size Class II has a more developed and ossified joint surface compared to similarly sized tibiae (IVPP V27124-4, 94 mm in length) so it is likely that with more sampling, the split between Size Class II and III will be refined. Size Class III includes immature specimens that are approximately

70% maximum known size (again, based on femoral lengths from larger, complete, Jiucaiyuan specimens). Finally, Size Class IV includes a single femur with an estimated length of 255 mm (the specimen measures 240 mm and is missing the proximal shaft and head of the femur). To our knowledge, it is the largest femur of *Lystrosaurus* yet recovered from the Jiucaiyuan Formation, and is ~ 5 cm longer than the largest femur (discussed above) recovered from the Early Triassic of South Africa. Therefore, it presumably represents the most skeletally mature individual in the current sample. However, histological analysis will provide critical context for the relative maturity of this, and all other specimens sampled [75].

Results

Geometric Morphometric Analysis of Skull Shape

Anterior view: The first five principal components capture 81.41% of the total shape variance in anterior view (PC1 - 30.57%; PC2 - 22.03%; PC3 - 13.82%; PC4 - 8.71%; PC5 - 6.26%) (Fig 2A). Shape is a statistically significant predictor of geographic location for the anterior view dataset ($p = 0.001$), and the canonical variates analysis correctly assigned specimen location 100% of the time.

PC1 primarily describes relative proportions of the snout. The subnarial region of the snout and caniniform process are dorsoventrally shorter relative to the height of the nasals and the anterior orbital margin in specimens with low PC1 scores, and there is greater lateral projection of the prefrontals relative to the caniniform process (Fig 2A). In contrast, the subnarial region and the caniniform process are proportionally much deeper relative to the anterior orbital margin and nasals in specimens with high PC1 scores, and the prefrontals and caniniform process are more closely aligned along a vertical axis. PC2 also captures aspects of skull

widening and deepening, although in this case the differences are concentrated in the region of the nasals and prefrontals. Specifically, the prefrontal-nasal region is dorsoventrally shorter in specimens with low PC2 scores, and the dorsal and ventral extrema of the prefrontal are displaced laterally relative to the external naris and the lateral end of the nasofrontal suture. Specimens with high PC2 scores have dorsoventrally taller prefrontal-nasal regions, and the dorsal and ventral extrema of the prefrontal are more closely aligned with the external naris and the lateral edge of the nasofrontal suture. The Chinese and South African specimens are most clearly differentiated along PC2, with the Chinese specimens generally having lower PC2 scores.

Dorsal view: The first five principal components capture 79.75% of the shape variance in dorsal view (PC1 - 26.77%; PC2 - 23.57%; PC3 - 17.95%; PC4 - 6.18%; PC5 - 5.26%) (Fig 2B). Shape is a statistically significant predictor of geographic location for the dorsal view dataset ($p = 0.001$), and the canonical variates analysis correctly assigned specimen location 100% of the time.

Shape variation along PC1 is dominated by differences in the relative lengths of the interorbital region and the pineal foramen, and by the degree of lateral projection of the orbital rim. Specimens with low PC1 scores are characterized by an anteroposteriorly longer interorbital region and a shorter pineal foramen, and an orbital rim that does not project strongly laterally. The pineal foramen is relatively longer anteroposteriorly in specimens with high PC1 scores, and the anterior and posterior corners of the orbital rim (formed by the prefrontal and postorbital bar, respectively) project laterally away from the skull roof. PC2 also captures shape variation associated with relative proportions of the skull roof, as well as orbit shape. Specimens with low PC2 scores have an anteroposteriorly shorter pineal foramen and longer frontals, and the orbit is

anteroposteriorly shorter, with anterior and posterior corners that are relatively aligned. The pineal foramen and orbit are relatively longer in specimens with high PC2 scores, and the posterior corner of the orbit (formed by the postorbital bar) projects farther laterally than the anterior orbital margin (formed by the prefrontal). As with the anterior view dataset, the Chinese and South African specimens are most clearly differentiated along PC2, with the Chinese specimens generally having lower PC2 scores.

Lateral view: The first five principal components capture 77.23% of the shape variance in lateral view (PC1 - 32.64%; PC2 - 18.57%; PC3 - 11.66%; PC4 - 8.58%; PC5 - 5.76%) (Fig 2C). Shape is a statistically significant predictor of geographic location ($p = 0.001$), and the canonical variates analysis had an overall classification accuracy of 95.78%, misclassifying two Chinese specimens in the dataset.

PC1 describes differences in the profile of the snout, position of the caniniform process relative to the orbit, and the anteroposterior dimensions of the orbit (Fig 2C). Specimens with low PC1 scores have a lower, more anteriorly-convex snout profile with prefrontals that do not project much above the level of the skull roof, and the ventral edge of the caniniform process is located below the anterior orbital margin. The anteroposterior length of the orbit is relatively long. In contrast, specimens with high PC1 scores, have much taller, flatter snout profiles and more dorsally-projecting prefrontals, as well as anteroposteriorly shorter orbits and caniniform processes that are located anterior to the anterior orbital margin. PC2 also captures aspects of snout profile, including the degree of anterior projection of anteroventral corner of the premaxilla and the length of the caniniform process, as well as the dorsoventral height of the orbit. The tip of the premaxilla projects more anteriorly in specimens with low PC2 scores, the caniniform

processes are relatively short, and the orbits are dorsoventrally shorter. Specimens with high PC2 scores have taller orbits, flatter snout profiles, and longer caniniform processes. There is little differentiation of the Chinese and South African specimens on the first three PC axes.

Taken together, our morphometric analyses suggest that there are consistent differences in skull shape between *Lystrosaurus* specimens from China and from South Africa, particularly in anterior and dorsal view. Based on these results, we tentatively suggest that the Chinese and South African populations were distinct at the species level. However, because our analyses do not directly address potential synonymies among the named Chinese species, and the fact that not all of the specimens we sampled histologically are associated with cranial material, we conservatively refer all of our histological samples to *Lystrosaurus* sp.

Osteohistology Overview

Lystrosaurus limb elements, regardless of size, have characteristically thick cortices, with bone wall thicknesses ranging from 1.8 mm thick in the smallest femoral thin section, to 4 mm thick in the largest femoral thin section. Medullary cavities are typically infilled with trabecular bone that gradually transitions to a compact cortex. Similar to the bone histology of other dicynodonts and therapsids more broadly, the dominant tissue type in all sampled size classes of *Lystrosaurus* is woven, fibrolamellar to laminar bone with a plexiform to longitudinal vascular orientation, indicative of rapid osteogenesis [76-80, 19, 29]. Very little of the cortex is remodeled and when secondary osteons are present, they are restricted to the deepest cortex. In the small individuals of *Lystrosaurus* studied here, rapidly deposited tissue is interrupted by either lines of arrested growth (LAGs) or annuli. LAGs are found in multiple regions of the cortex, including

deep in the cortex and along the sub-periosteal margin. In some individuals, there is variability in the type of growth mark that is deposited among the skeletal elements sampled. For example, the first (i.e., osteologically deeper) growth mark of IVPP V26548 varies from a LAG in the femur and tibia, to an annulus in the fibula. Interestingly, the largest and presumably most skeletally mature femur that was histologically sampled, shows uninterrupted cortical growth, suggesting that *Lystrosaurus* from the Jiucaiyuan Formation had a high intrinsic rate of growth that could periodically arrest.

Our results are consistent with a recent report of *Lystrosaurus* bone histology from the same formation with respect to the preponderance of highly vascularized, rapidly deposited cortical bone [39]. However, contrary to Han et al. [39], we do not find evidence of peripherally slowed growth rates in individuals that are as large (~60% MKS) and larger than the maximum size sampled in their study. Additionally, we pose an alternative interpretation to the assignment of LAGs in the laminar to plexiform tissue reported in ([39]:fig 2G-H, fig 3C, fig 5, fig 6D, G-H) as bright lines that appear in the context of laminar bone formation ([81]:fig3C; [82]:fig3). Finally, we report fewer instances of LAGs, particularly in the smallest size class where no evidence of episodic growth marks is seen (contra [39]). When present, we can confidently trace 1 to 3 growth marks (as either LAGs or annuli) around the circumference of individuals that are approximately 70% maximum known size.

Size Class II

The smallest association of elements includes cranial and postcranial material (hind limb and forelimb elements) that was collected in situ. A femur, tibia, fibula, ulna and radius were selected for consumptive sampling (composite images available on Morphobank project

4023). The femur measures 122.38 mm in length. When compared to femora from larger, presumably more mature individuals, this femur (IVPP V27126a) is approximately 50% maximum known size. The bone tissue microstructure in each of the five associated limb elements is extremely well-vascularized with plexiform to radial vascular canals (Fig 3). Woven fibered bone matrix and large, densely distributed osteocyte lacunae surround the primary vascular canals. Vascular canal size and density remain consistently high from the endosteal to the periosteal margin indicating that this individual grew rapidly until death (Fig 3D-E). There is no evidence of episodic growth arrests (as either LAGs or annuli) in this individual.

Figure 3. Representative bone histology of Size Class II from the mid-shafts of *Lystrosaurus* sp. forelimb and hind limb elements. **A**, fibula (IVPP V27124-1) consisting of well-vascularized plexiform tissue. **B**, 10X magnification of **A**, arrows indicate regions of woven bone matrix in the laminar, fibrolamellar complex that should not be confused as LAGs. **C**, 20X magnification of **B**. **D**, fibula (IVPP V27126c) cross section consisting of plexiform canals and dense osteocyte lacunae. **E**, cross section of the ulna from the same individual (IVPP V27126e) similarly shows a cortex of highly vascularized plexiform tissue indicating rapid bone deposition at death. **F**, cross section of a proximal tibia (IVPP V27124-3) with a slight decrease in vascular canal sizes towards the sub-periosteal edge, magnified in **G**. po= primary osteon. **B**, left side of **C**, and **E** show cross-polarized light with a lambda filter. Scale bars = 1 mm in **A** & **F**, 500 μm in **D**, **E**, **G**, 100 μm in **C&E**.

A right proximal fibula (IVPP V27124-1), right proximal tibia (IVPP V27124-3), distal tibia (IVPP V27124-4), distal femur (IVPP V27124-6) and complete femur (IVPP V27124-7)

were collected individually but are analyzed together here as immature individuals because limb dimensions and histological features are indicative of sustained, active bone growth at death. The complete femur is approximately 50% maximum known size, similar to IVPP V27126a. In thin section, each of these disassociated elements has a large medullary cavity infilled with a loose trabecular network. Cortical tissue consists of fibrolamellar bone with plexiform to longitudinal canal orientations. In the proximal tibia, vascular canal size decreases substantially in a regionalized band deep to the sub-periosteal edge but rebounds to the typical highly vascularized tissue more peripherally (Fig 3F-G). In the fibula, there are regional differences in vascular canal size and organization that likely represent functional differences in bone apposition rates [83]. For example, longitudinal vascular canals are organized into stacked, circumferential layers on the medial side of the proximal fibula (IVPP V27124-1) whereas the lateral side is less organized (Fig 3A). In more organized areas of the highly vascularized fibrolamellar tissue, the mineralized matrix surrounding the reticular vascular canals should not be confused for lines of arrested growth (Fig 3B-C). At low magnifications, delineated bands of matrix separate the primary vascular space in laminar bone [82]. However, at high magnifications, these bright bands do not form continuous rings around the cortex, nor are they hyper-mineralized, two of the most diagnostic characteristics of lines of arrested growth [41]. Highly vascularized, reticular fibrolamellar bone continues to the sub-periosteal edge in these specimens in Size Class II, indicating that individuals died while actively growing.

Size Class III

A femur (IVPP V27125a), tibia (IVPP V27125b), and fibula (IVPP V27125c) are associated as one individual that is approximately 70% maximum size based on femoral length.

Unlike smaller sampled elements, the cortex is interrupted by two growth marks (Fig 4). In all associated elements, a deep cortical LAG is sandwiched between large vascular canals towards the endosteal margin and smaller vascular canals more peripherally (Fig 4A-B, lowermost arrows in F, K). In the fibula, the LAG is superficial to a region of immature secondary osteons that sit in the deep cortex (Fig 4J). The second of the two growth marks is more variable. In the femur, a region of stacked, circumferentially organized, and poorly vascularized tissue sits along the sub-periosteal edge in thin areas of the cortex (Fig 4D). By contrast, thicker regions of the outer cortex show continuous laminar to fibrolamellar bone tissue (Fig 4C). In the tibia and fibula, the outer cortical growth mark is made up of two LAGs in the tibia, and an annulus of stacked LAGs that vary in number around the cross-section of the fibula (Fig 4E, J-K). Interestingly, the mineral preservation of the tibia and fibula are such that the mineralized bone matrix is clearly visible between each layer of vascular canals (Fig 4F, K, L). These thin, mineralized lines of extracellular matrix should not be confused for LAGs and instead are the mineralizing front of laminar, fibrolamellar bone deposition and growth [41, 81]. The vascular orientation shifts in the hindlimb elements of this individual from plexiform to longitudinal in the middle to outer cortex. In addition, vascular canal size decreases towards the outermost periosteal margin but remains open to the sub-periosteal edge (Fig 4F-G). The only exception to this is in the thinnest region of the cortices, particularly in the fibula, where the sub-periosteal edge is scarcely vascularized and instead, consists of well organized, lamellar tissue (Fig 4D, I, K). However, this lamellar tissue is discontinuous around the cross section, suggesting that bone growth slowed at differential rates in regions of the outer cortex, but did not fully arrest.

Figure 4. Bone histology of associated hind limb elements of *Lystrosaurus* sp. representative of Size Class III. **A**, femur (IVPP V27125a) with a deep cortical LAG in **B** (blue box). The outer cortex has regions of high vascularity seen in **C** (yellow box in **A**), and regions of lower vascularity seen in **D** (orange box in **A**). **E**, tibia (IVPP V27125b) has two cortical growth marks seen in **F** (yellow box in **E**; yellow arrows in **F**). The outer growth mark can variably be seen as two LAGs, **G** (green box in **E**; yellow arrows in **G**). **H**, the fibula (IVPP V27125c) has a thinner cortex interrupted by two LAGs seen in **I** (yellow box in **H**; yellow arrows in **I**). **J**, magnification of yellow box in **I** shows the cross-cutting inner cortical LAG (arrow in **J**). **K**, thinner regions of the cortex appear avascular (purple box in **H**), with stacked mineralized lamellae (yellow bracket), whereas thicker regions (blue box in **H**) show peripheral pulses of vascularized growth, within the lamellated tissue, seen in **L** (yellow bracket). **C**, **D**, **I**, and **J**, left sides of **F**, **G**, **K**, and **L** show cross-polarized light with a lambda filter. Scale bars = 5 mm in **A**, 1 mm in **E**, 2 mm in **H**, 500 µm in **B**, **C**, **F** & **I**, and 100 µm in **D**, **G**, **J**-**L**.

A slightly larger specimen (IVPP V26548) is an association of hind limb and forelimb elements as well as at least one rib that was previously histologically sampled by Han et al. 2021 [39]. The femur (IVPP V26548a), tibia (IVPP V26548b), and fibula (IVPP V26548c) show plexiform tissue interrupted by two growth marks, with some variation in the outer growth mark, especially in the fibula where it splits into two LAGs (Fig 5). In the femur and tibia, the deeper growth mark is distinguishable as a dramatic reduction in vascular canal size, immediately followed by a return to the typical, large vascular canals in the middle of the cortex (Fig 5A, B, D). Throughout the middle cortex of the femur, the deeper growth mark changes in thickness. In thinner regions of the cortex, the growth mark appears as a cross-cutting LAG separating smaller

vascular canals from larger canals more superficially. In the thicker regions of the cortex, the growth mark appears as an annulus, or region of little to no vasculature that is not accompanied by a hyper-mineralized line (Fig 5D). In both the femur and tibia, vascular canals reduce in size again towards the sub-periosteal margin, making up the second growth mark of stacked lamellar tissue (Fig 5C). In contrast, the fibula shows a mid-cortical growth mark that is made up of multiple stacked LAGs in a circumferential band of reduced vascularity (Fig 5G, I). Similar to the femur and tibia, there is a sub-periosteal reduction in vascular canal size with at least two LAGs that can be traced around the entire cortex of the fibula, indicating variation in the type of growth mark deposited in hind limb elements of the same individual (Fig 5E, G, H). We tentatively interpret the presence of stacked lamellar layers in this individual as a cessation in growth. However, small, simple vascular canals sit within the context of this organized lamellar tissue, which is atypical for an EFS, making it more likely that this lamellar tissue represents a temporary cessation in growth, similar to the annuli recorded in the deeper cortex.

Figure 5. Bone histology of associated elements of *Lystrosaurus* sp. representative of Size Class III. **A**, femur (IVPP V26548a) has two growth marks. **B**, a deep, mid-cortical annulus (yellow box in **A**; lower yellow bracket in **B**), stacked lamellar tissue makes up the second growth mark (upper yellow bracket in **B**). **C**, magnification of peripheral lamellar tissue (blue box in **A**; yellow bracket in **C**). **D**, first (osteologically deeper) annulus (yellow bracket). **E**, magnification of blue box in **A** of outer cortical growth mark that varies in thickness around the periphery of the thin section, here consisting of two closely spaced LAGs. **F**, associated fibula (IVPP V26548c) highlights a similar arrangement of deep and more superficial growth marks consisting of, at times, numerous layers of lamellar tissue in an annulus. **G**, magnification of yellow box in

F showing two growth marks; annulus (yellow bracket) and peripheral LAGs (yellow arrows). **H**, magnification of green box in **F** showing variation in the outer growth mark such that in some regions it appears as a thicker region of lamellar tissue with one cross-cutting LAG. **I**, magnification of blue box in **F**; deep cortical annulus (bracket). **J**, humerus with mid-cortical annulus of reduced vascularity. **K**, magnification of yellow box in **J** of growth mark (yellow bracket). **L**, ulna (IVPP V27124-2) with thinner overall cortex and reduced vasculature. **M**, magnification of yellow box in **L** highlighting the disorganized cortex in plane and cross-polarized light with a lambda filter. Left side of **C**, **D**, **H**, **I**, and upper portion of **M** show cross-polarized light and a lambda filter. Scale bars = 1 mm in **A**, **B**, **F**, **J**, **L**, 500 µm in **E**, **G**, **I**, **K**, and 100 µm in **C**, **D**, **H**, **M**.

A complete humerus (IVPP V27124-8) of comparable size to IVPP V26548, has an exceedingly thick cortex with one mid-cortical growth mark (Fig 5J). The medullary cavity is nearly infilled by a dense network of trabecular bone. The cortex increases in thickness in the anterodorsal region (up in Fig 5J) and consists of highly vascularized laminar to fibrolamellar bone. Vascular canals have a circular to plexiform orientation and show a marked decrease in size coincident with a mid-cortical LAG (Fig 5K). The LAG is traceable around the entire cross-section and is followed by a pulse of highly vascularized reticular tissue with large canal spaces (Fig 5K). Vascular canal size reduces slightly towards the sub-periosteal margin, which could indicate the beginning of another growth mark, similar to the mid-cortical growth mark.

A fragment of a distal ulna (IVPP V27124-2) is estimated to be 93 mm long and has a thin cortex with a large medullary cavity infilled with a loose network of trabecular bone (Fig 5L). The transition from trabeculae to compact cortical bone includes a region of enlarged

resorption cavities and immature secondary osteons. The middle and outer cortex is composed of small longitudinal and reticular canals that sit in a parallel-fibered matrix where small, lenticular osteocyte lacunae are sparsely distributed. Along the sub-periosteal margin, vascular canals are reduced but occasionally open to the bone edge, suggesting that this individual died while still growing (Fig 5M). The overall construction of this element is suggestive of a slower growth rate when compared to other long bones, but there is no evidence to suggest somatic maturity or attainment of maximum size.

Size Class IV

Unlike the middle size class, the largest sampled femur (IVPP V27127) does not have LAGs or annuli (Fig 6A). Despite cracks in the cortex, the bone tissue architecture is easily discernible and consists of longitudinal canals in plexiform to circular organization. The fiber-orientation is less clear due to the poor preservation of this specimen, but it appears parallel-fibered in the deep cortex and woven in the outer cortex, of which, only a small portion remains intact (Fig 6A-C). There is no indication of slowed bone deposition in the small regions of sub-periosteal bone, suggesting a sustained rate of growth at death.

Figure 6. Bone histology of the largest femur (IVPP V27127) of *Lystrosaurus* sp. that represents Size Class IV and rib fragments of unknown size. **A**, the femur is cracked and incomplete but preserves regions of the entire cortex (yellow box). **B**, same view of the yellow box in **A** shows parallel-fibered bone in the mid cortex, a large crack (yellow arrow), and well-vascularized, plexiform tissue along the sub-periosteal margin (yellow box in **B**). **C**, magnification of yellow box in **B** shows sustained, rapid growth at death. **D**, rib fragments (IVPP V27124-5) have infilled

medullary cavities, secondary osteons within the deep cortex and a largely avascular compact outer cortex consisting of an EFS. **E**, magnification of yellow box in **D** showing an EFS in the yellow bracket. **F**, magnification of the yellow box in **E**. **G**, magnification of blue box in **D** similarly shows that an EFS is present (yellow bracket). **B**, left side of **C**, **F**, and **G**, show cross-polarized light with a lambda filter. Scale bars = 10 mm in **A**, 1 mm in **B** & **D**, 500 µm in **C** & **G**, 100 µm in **E** & **F**.

Unknown Size Class

Four partial ribs (IVPP V27124-5) belonging to an individual of unknown size class are preserved in a block of sediment (Fig 6D). In thin section, they have large, infilled medullary cavities that grade into thin, compact cortices. Scarce vascular canals and immature secondary osteons sit in the deeper cortex whereas the outer cortex is avascular with an EFS of stacked, circumferential LAGs (Fig 6E-G). Osteocyte lacunae are well organized in this lamellar tissue (Fig 6F).

Discussion

All *Lystrosaurus* individuals sampled from the Jiucaiyuan Formation show abundant, highly vascularized primary bone tissue throughout the majority of the cortices of the hind limb, humerus, and radius. The ulna and ribs showed fewer vascular canals, overall thinner cortices, and more organized primary bone tissue. Three size classes were identified and histologically described as: II) a highly vascularized cortex of radial to plexiform vascular canals in ~50% max known size individuals: III) highly vascularized plexiform tissue interrupted by one to two growth marks that range from a single LAG or set of closely spaced LAGs to annuli in ~70%

maximum known size individuals; and IV) uninterrupted plexiform tissue in a 100% maximum known size individual. These results force us to reconsider what an adult-sized specimen might look like for *Lystrosaurus* sp., as the current sample lacks histologic indicators of somatic maturity (i.e., an external fundamental system) [75]. Botha [22] found similar results for South African species of *Lystrosaurus*, suggesting that species typified by small body size (viz. *L. murrayi*) were very likely capable of reaching much larger sizes, but never lived long enough to do so.

Han et al. [39] suggested that sub-adult *Lystrosaurus* from northern Pangea may have reached some level of somatic maturity inferred from parallel-fibered bone and peripheral LAGs in a dorsal rib. However, histological data from the femur, tibia, and fibula of the same individual indicate episodic cessations in growth instead of an overall pattern of slowed growth. The presence of slower growing bone tissue in the ribs likely reflects differential growth rates seen throughout the skeleton, rather than somatic maturity [84, 45].

Growth Marks

Unlike the bone histology reported from South African and Indian *Lystrosaurus*, in which growth marks are rare and often limited to a single LAG or annulus, we find one, and more commonly, two growth marks in immature *Lystrosaurus* from the Jiucaiyuan Formation. The number of growth marks is mostly consistent across associated elements from the same individual as well as across individuals of the same size class (Table 1). Interestingly, the type of growth mark, as either a LAG, a series of closely spaced and occasionally bifurcating LAGs, annulus consisting of stacked LAGs, or annulus consisting of avascular tissue, varied throughout the current sample. In most cases, the first (i.e., osteologically deeper) growth mark, or singular

growth mark in the case of the humerus, was often recorded as an annulus accompanied by a reduction of vascular canal sizes. The outer cortical growth marks were more variable, and often varied within the same thin section.

We did not find evidence of growth marks in individuals in Size Class II. These individuals had characteristic juvenile bone histology (i.e., large radial to reticular vascular canals); therefore our analysis disagrees with the assignment of LAGs in juvenile *Lystrosaurus* by Han et al. [39]:fig 3. In some cases, the mineralizating front can be mis-identified as a LAG in the context of laminar or plexiform tissue, wherein one unit of laminar bone is constructed by a mineralized line upon which woven bone is deposited [81]. This mineralized line differs from a LAG as it is not continuous around the cortex and is not hyper-mineralized but instead appears as a bright line often devoid of osteocyte lacunae [81]. A LAG on the other hand, is a continuous mineralized line around the cortex, unless there is clear evidence for secondary remodeling or cortical remodeling that disrupts (i.e., erodes) it.

All growth marks observed in our analysis come from Size Class III, which corresponds to individuals that are approximately 70% maximum known size. We can confidently trace up to two growth marks (occasionally made up of multiple LAGs) in the current Chinese sample, which is higher than reports from South African and Indian specimens of *Lystrosaurus,* which generally lack growth marks. However, the remainder of the cortical bone tissue in the current Chinese sample reflects the similarly high and consistent rate of bone growth reported from South African and Indian *Lystrosaurus* [22, 51]. A slight increase in the number of growth marks in northern Pangean *Lystrosaurus* could be explained by one or two possible factors that are not mutually exclusive: (i) by an increase in their lifespan or (ii) by environmental stressors.

There is a longstanding hypothesis that the Permo-Triassic extinction caused a Lilliput effect, a persistent reduction in body size, in surviving terrestrial lineages [85-88, 19]. This claim has been investigated histologically in therocephalians, which support an overall reduction in body size following the Permo-Triassic extinction [88, 19]. A Lilliput effect was also posited for *Lystrosaurus*, based in large part on the overwhelming abundance of small skulls in the Karoo Basin and museum collections in South Africa [89, 90, 20, 21]. However, Botha [22] later rejected a strict Lilliput effect for southern Pangean *Lystrosaurus* (based on fossils from South Africa and India) based on the lack of mature histological tissue in all of the species examined [22]. Instead, she [22] suggested that the large proportion of small skulls in the Triassic was better explained by high mortality in immature individuals, as assessed by their long bone histology.

Body size

In order to better understand how *Lystrosaurus* from the Jiucaiyuan Formation compare to congeners from more southerly parts of Pangea, we gathered body size data (based on basal skull length and femur length) from the literature [51, 22, 39] and personal observations from Early Triassic-aged specimens collected in China, South Africa, and India. From this sample, it is clear that Chinese *Lystrosaurus* have larger average body size (Fig 7, Fig 8). However, cranial size distributions suggest that populations from both South Africa and China had the potential to grow to similar maximum sizes (Fig 7). The lack of large specimens of *Lystrosaurus* in South Africa aligns with previous interpretations for increased mortality at small sizes there by Botha [22]; also see [20]. Our data also provide evidence suggesting that northern Pangean *Lystrosaurus* could have reached larger sizes more readily or easily. However, it is unclear the

degree to which differences in sampling practices might affect the body size distributions seen here. A decade of detailed stratigraphic work targeting the Permo-Triassic boundary in South Africa has led to more comprehensive fossil sampling, which should provide a relatively unbiased view of the vertebrate fauna [91, 92]. However, historical collections from the Karoo likely systematically emphasized rare components of the assemblage (e.g., [93]). Likewise, the Permo-Triassic vertebrate record from Xinjiang has not been subject to the same intensive collecting as that of the Karoo Basin, and therefore it currently may not provide an unbiased view of body size distributions or relative abundances of taxa.

Figure 7. Interspecific body size distributions from Early Triassic *Lystrosaurus* show an abundance of large individuals from China. South African and Chinese species overlap in maximum basal skull length but individuals from China have larger average size. See S2 Supplementary Dataset for more information.

Figure 8. Interspecific femoral lengths from Early Triassic *Lystrosaurus* show a higher frequency of large sizes from China. Size Classes denote femora that are less than 40% maximum known size (MKS) in I, between 40-65% MKS in II, approximately 70-75% MKS in III, and greater than 79% in IV. See S2 Supplementary Dataset for more information.

The presence of large individuals with episodic growth marks in all of the subadult-sized elements suggests that *Lystrosaurus* from the Jiucaiyuan Formation were able to reach large sizes through prolonged and sustained rapid growth, unlike Triassic *Lystrosaurus* from South Africa that rarely lived long enough to reach large body sizes despite growing rapidly (Fig 7).

There are several possible explanations for this pattern. First, physiological differences among the species of *Lystrosaurus* could be responsible, especially given our morphometric results, which suggest that the South African and Chinese populations were distinct. This proposal is difficult to test at this time, as the alpha-taxonomy of Chinese *Lystrosaurus* is in need of detailed revision and phylogenetic studies of the genus have typically only included *L. hedini* as a sole northern hemisphere representative (e.g., [94]). However, to the degree that patterns preserved in hard tissue histology can shed light on physiology, the current data suggests that *L. curvatus*, *L. declivis*, *L. maccaigi*, and *L. murrayi* are effectively indistinguishable [22, 30], which suggests a degree of consistency among species within the genus.

Climatic differences in the high-southern (60°S) versus mid-northern (45°N) paleolatitudes [95, 96] are another possible explanation for the observed difference in body size and inferred lifespan in *Lystrosaurus*. Paleoclimate reconstructions of the latest Permian to earliest Triassic show extreme increases in temperature and aridity [97-100], mega monsoons [101, 102], chemical weathering, soil erosion, and geochemical and isotopic signatures of mass wasting and greenhouse conditions [8, 96, 103, 104]. Facies that span the terrestrial Permo-Triassic boundary of the Karoo Basin are interpreted as a change from meandering rivers to low-sinuosity braid-plains, resulting from increased aridity and a die-off of rooted plants that supported river morphologies [105, 106]. However, more recent evidence from isotopic and geochemical data produced differing estimates of the degree to which aridity increased in the Karoo Basin, complicating our understanding of seasonal variation in precipitation and resource availability [102, 107, 108]. Nevertheless, conditions were disruptive enough to have destabilized terrestrial communities and caused a prolonged interval of faunal turnover in southern Pangea during the extinction interval [109, 15, 18].

The extreme environmental and climatic conditions of southern Pangea are not reflected in the paleoclimate reconstructions of the Jiucaiyuan Formation [72]. Here, stable subhumid to semiarid conditions are interpreted from cyclostratigraphic and paleosol analyses in the South Taodonggou study area [71, 72]. Furthermore, fluvial and lacustrine strata host a diversity of plant, root, and wood fossils that are occasionally preserved in situ and do not preserve cyclical growth marks when thin-sectioned [110-112, 72]. The absence of growth rings in these woody pieces provides some evidence for stable environmental and climatic conditions. In sum, evidence from the paleobotanical record of the Jiucaiyuan Formation suggests a lush and vegetated environment that hosted a diverse terrestrial fauna in earliest Triassic times [61, 39]. Taken together, there is mounting evidence from sedimentologic, isotopic, and cyclostratigraphic data that points to seasonal, semi-arid conditions in the Karoo Basin and stable, sub-humid conditions in the Turpan-Hami Basin.

The extreme environmental and climatic conditions inferred from the Karoo Basin likely truncated *Lystrosaurus* lifespans in the Early Triassic. Similar environmental constraints on maximum size and longevity are also known from Early Triassic therocephalians and terrestrial temnospondyls, which also lack LAGs [19, 49]. By contrast, we propose that the more favorable environmental conditions and vegetated landscape interpreted from the Jiucaiyuan Formation could have hosted longer-lived *Lystrosaurus* during the Early Triassic. Relatively stable environmental conditions could have allowed populations of *Lystrosaurus* to reach larger average body sizes than their southern Pangean relatives. The prevalence of multiple growth marks in some, but not all, immature individuals, further supports developmentally plastic growth in *Lystrosaurus* first proposed for southern Pangean populations [22]. It also suggests that some individuals experienced periodic arrests of growth, perhaps associated with

environmental disruptions, but that these disturbances may not have caused widespread mortality on the scale implied by the South African record. Similarly, some individuals apparently experienced sufficiently stable conditions for them to grow to comparatively large size without interruption. Finally, the absence of peripheral parallel-fibered bone or consistent, circumferential lamellar tissue in the current sample indicates that the maximum size of *Lystrosaurus* from the Jiucaiyuan Formation remains unknown.

Conclusions

We present bone histology, cranial geometric morphometric analyses, and body size data for *Lystrosaurus* from the Jiucaiyuan Formation, and report statistically different cranial morphologies, an extended lifespan, and larger average body size compared to *Lystrosaurus* living in southern Pangea during Early Triassic times. In addition, we report up to two growth marks in immature individuals, which is inconsistent with previous reports of *Lystrosaurus* life histories from the Turpan Basin [39]. We suggest that it is unlikely that the growth marks reported here represent regular, cyclical patterns and more likely represent instances where bone growth stopped due to unfavorable environmental conditions. Indeed, the absence of growth marks in the largest sampled individual indicates an intrinsically high rate of growth that persisted throughout ontogeny. In addition, the presence of two cessations in growth in individuals from Size Class III indicates that smaller, likely more immature, individuals may have been more susceptible to fluctuations in environmental conditions. However, appositional bone growth rebounded to a rapid rate following these cessations, indicating a flexible physiology, as previously proposed for higher-latitude populations of *Lystrosaurus* [22, 30]. A better sample of large limb bones (>250 mm) as well as an analysis of tusk dentine deposition,

are needed to clarify what impact the Early Triassic environment may have had on *Lystrosaurus* growth and development in northern Pangea.

Acknowledgements. —This research was supported by National Science Foundation grants EAR 1714829 (to KDA) and EAR 1713787 (to CAS), and UW Biology Department Iuvo and Walker awards (to ZTK). At the IVPP, we thank Yi Jian and Shukang Zhang for assistance with thin section preparation as well as Jun Liu for access to field data and specimen collections. We also thank Alida Bailleul for helpful discussion and microscope access. Collecting and compiling the entirety of the comparative datasets would not have been possible if it weren't for the following museum curators, collection managers, colleagues, and friends who we deeply thank: Zaituna Skosan, Claire Browning, Christian Kammerer, Roger Smith, Jennifer Botha, Elize Butler, Bernhard Zipfel, Bruce Rubidge, Sifelani Jirah, Viktor Radermacher, Carl Mehling, and Mark Norell. For their helpful discussion on earlier versions of this manuscript, we thank Bryan Gee, Savannah Olroyd, and Elliott Armour Smith. Christian Kammerer provided images of South African Lystrosaurus specimens that were utilized in the morphometric analyses. Finally, we are indebted to Wan Yang for collecting some of the fossils analyzed here, organizing fieldwork in 2019 and leading the multidisciplinary project that instigated this research.

References

1. Raup DM, Sepkoski JJ. Periodicity of extinctions in the geologic past. Proc Natl Acad Sci. 1984 Feb 1;81(3):801–5.

2. Erwin DH. The Permo–Triassic extinction. Nature. 1994 Jan;367(6460):231–6.

3. Chen Z-Q, Benton MJ. The timing and pattern of biotic recovery following the end-Permian mass extinction. Nat Geosci. 2012 Jun;5(6):375–83.

4. Benton M. Diversification and extinction in the history of life. Science. 1995 Apr 7;268(5207):52–8.

5. Benton MJ, Twitchett RJ. How to kill (almost) all life: the end-Permian extinction event. Trends Ecol Evol. 2003 Jul;18(7):358–65.

6. Sahney S, Benton MJ. Recovery from the most profound mass extinction of all time. Proc R Soc B Biol Sci. 2008 Apr 7;275(1636):759–65.

7. Sobolev SV, Sobolev AV, Kuzmin DV, Krivolutskaya NA, Petrunin AG, Arndt NT, et al. Linking mantle plumes, large igneous provinces and environmental catastrophes. Nature. 2011 Sep;477(7364):312–6.

8. Benton MJ, Newell AJ. Impacts of global warming on Permo-Triassic terrestrial ecosystems. Gondwana Res. 2014 May;25(4):1308–37.

9. Retallack GJ, Krull ES. Carbon isotopic evidence for terminal-Permian methane outbursts and their role in extinctions of animals, plants, coral reefs, and peat swamps. In: Greb SF, DiMichele WA, editors. Wetlands Through Time: Geological Society of America Special Paper. 2006. p. 249–68.

10. Nowak H, Schneebeli-Hermann E, Kustatscher E. No mass extinction for land plants at the Permian–Triassic transition. Nat Commun. 2019 Dec;10(1):384.

11. Mishra S, Jha N, Stebbins A, Brookfield M, Hannigan R. Palaeoenvironments, flora, and organic carbon and nitrogen isotope changes across the non-marine Permian-Triassic boundary at Wybung Head, Australia. Palaeogeogr Palaeoclimatol Palaeoecol. 2019 Nov;534:109292.

12. Botha J, Smith RMH. Rapid vertebrate recuperation in the Karoo Basin of South Africa following the End-Permian extinction. J Afr Earth Sci. 2006 Aug 1;45(4):502–14.

13. Botha J, Abdala F, Smith R. The oldest cynodont: new clues on the origin and early diversification of the Cynodontia. Zool J Linn Soc. 2007;149(3):477–92.

14. Fröbisch J. Vertebrate diversity across the end-Permian mass extinction — Separating biological and geological signals. Palaeogeogr Palaeoclimatol Palaeoecol. 2013 Feb;372:50–61.

15. Roopnarine PD, Angielczyk KD, Weik A, Dineen A. Ecological persistence, incumbency and reorganization in the Karoo Basin during the Permian-Triassic transition. Earth-Sci Rev. 2019 Feb;189:244–63.

16. Bernardi M, Petti FM, Benton MJ. Tetrapod distribution and temperature rise during the Permian–Triassic mass extinction. Proc R Soc B Biol Sci. 2018 Jan 10;285(1870):20172331.

17. Allen BJ, Wignall PB, Hill DJ, Saupe EE, Dunhill AM. The latitudinal diversity gradient of tetrapods across the Permo-Triassic mass extinction and recovery interval. Proc R Soc B Biol Sci. 2020 Jun 24;287(1929):20201125.

18. Viglietti PA, Benson RBJ, Smith RMH, Botha J, Kammerer CF, Skosan Z, et al. Evidence from South Africa for a protracted end-Permian extinction on land. Proc Natl Acad Sci. 2021 Apr 27;118(17):e2017045118.

19. Huttenlocker AK, Botha-Brink J. Bone microstructure and the evolution of growth patterns in Permo-Triassic therocephalians (Amniota, Therapsida) of South Africa. PeerJ. 2014 Apr 8;2:e325.

20. Botha-Brink J, Codron D, Huttenlocker AK, Angielczyk KD, Ruta M. Breeding Young as a Survival Strategy during Earth's Greatest Mass Extinction. Sci Rep. 2016 Jul;6(1). Available from: http://www.nature.com/articles/srep24053

21. Codron J, Botha-Brink J, Codron D, Huttenlocker AK, Angielczyk KD. Predator-prey interactions amongst Permo-Triassic terrestrial vertebrates as a deterministic factor influencing faunal collapse and turnover. J Evol Biol. 2017 Jan;30(1):40–54.

22. Botha J. The paleobiology and paleoecology of South African *Lystrosaurus*. PeerJ. 2020 Nov 24;8:e10408.

23. Nicolas M, Rubidge BS. Changes in Permo-Triassic terrestrial tetrapod ecological representation in the Beaufort Group (Karoo Supergroup) of South Africa. Lethaia. 2010 Mar;43(1):45–59.

24. Smith R, Rubidge BS, van der Walt M. Therapsid Biodiversity Patterns and Paleoenvironments of the Karoo Basin, South Africa (pp. 31-63) Roger Smith, Bruce Rubidge and Merrill van der Walt. In: The Forerunners of Mammals. Indiana University Press; 2012.

25. King GM, Cluver MA. The Aquatic *Lystrosaurus*: An Alternative Lifestyle. Hist Biol. 1991;4:323–41.

26. Jasinoski SC, Rayfield EJ, Chinsamy A. Comparative Feeding Biomechanics of *Lystrosaurus* and the Generalized Dicynodont *Oudenodon*. Anat Rec Adv Integr Anat Evol Biol. 2009 Jun 1;292(6):862–74.

27. Retallack GJ, Smith RMH, Ward PD. Vertebrate extinction across Permian–Triassic boundary in Karoo Basin, South Africa. Geol Soc Am Bull. 2003;115(9):1133.

28. Botha-Brink J. Burrowing in *Lystrosaurus*: preadaptation to a postextinction environment? J Vertebr Paleontol. 2017 Sep 3;37(5):e1365080.

29. Botha-Brink J, Angielczyk KD. Do extraordinarily high growth rates in Permo-Triassic dicynodonts (Therapsida, Anomodontia) explain their success before and after the end-Permian extinction? Zool J Linn Soc. 2010 Oct 1;160(2):341–65.

30. Whitney MR, Sidor CA. Evidence of torpor in the tusks of *Lystrosaurus* from the Early Triassic of Antarctica. Commun Biol. 2020;3(1). Available from: http://www.nature.com/articles/s42003-020-01207-6

31. Tripathi C, Puri SN. On the remains of *Lystrosaurus* from the Panchets of the Raniganj coalfield. Rec Geol Surv India. 1961;89:407–19.

32. Colbert EH. *Lystrosaurus* from Antarctica. Am Mus Novit. 1974;(2535):44.

33. Gubin Y, Sinitza S. Triassic terrestrial tetrapods of Mongolia and the geological structure of the Sain-Sar-Bulak locality. In: The Nonmarine Triassic. 1993. p. 169–70.

34. Liu J, Li J, Cheng Z. The *Lystrosaurus* fossils from Xinjiang and their bearing on the terrestrial Permian Triassic boundary. Vertebr Pal Asiat. 2002;40(4):267–75.

35. Sun A. Late Permian and Triassic Terrestrial Tetrapods of North China. Vertebr Palasiat. 1980;XVIII(2):11.

36. Ray S. *Lystrosaurus* (Therapsida, Dicynodontia) from India: Taxonomy, relative growth and Cranial dimorphism. J Syst Palaeontol. 2005 Jan;3(2):203–21.

37. Surkov MV, Kalandadze NN, Benton MJ. *Lystrosaurus georgi*, a dicynodont from the Lower Triassic of Russia. J Vertebr Paleontol. 2005 Jun 27;25(2):402–13.

38. Araújo R, Fernandez V, Polcyn MJ, Fröbisch J, Martins RMS. Aspects of gorgonopsian paleobiology and evolution: insights from the basicranium, occiput, osseous labyrinth, vasculature, and neuroanatomy. PeerJ. 2017 Apr 11;5:e3119.

39. Han F, Zhao Q, Liu J. Preliminary bone histological analysis of *Lystrosaurus* (Therapsida: Dicynodontia) from the Lower Triassic of North China, and its implication for lifestyle and environments after the end-Permian extinction. Fröbisch J, editor. PLOS ONE. 2021 Mar 18;16(3):e0248681.

40. Padian K, Lamm E-T, editors. Bone histology of fossil tetrapods: advancing methods, analysis, and interpretation. Berkeley: University of California Press; 2013. 285 p.

41. Francillon-Vieillot H, de Buffrénil V, Castanet J, Géraudie J, Meunier FJ, Sire JY, et al. Microstructure and Mineralization of Vertebrate Skeletal Tissues. In: Skeletal

Biomineralization: Patterns, Processes and Evolutionary Trends. American Geophysical Union; 1990. p. 175–234. Available from: http://dx.doi.org/10.1029/SC005p0175

42. Enlow H, Brown SO. A Comparative Histological Study of Fossil and Recent Bone Tissues. Part II. Tex J Sci. 1957;9(2):30.

43. Enlow H, Brown SO. A comparative histological study of fossil and recent bone tissues, Part III. Tex J Sci. 1958;10(2):187–230.

44. Castanet J, Newman DG, Girons HS. Skeletochronological Data on the Growth, Age, and Population Structure of the Tuatara, *Sphenodon punctatus*, on Stephens and Lady Alice Islands, New Zealand. 1988;14.

45. Castanet J. Time recording in bone microstructures of endothermic animals; functional relationships. Comptes Rendus Palevol. 2006 Mar;5(3–4):629–36.

46. Köhler M, Marín-Moratalla N, Jordana X, Aanes R. Seasonal bone growth and physiology in endotherms shed light on dinosaur physiology. Nature. 2012 Jul;487(7407):358–61.

47. Nacarino-Meneses C, Köhler M. Limb bone histology records birth in mammals. PLOS ONE. 2018 Jun 20;13(6):e0198511.

48. Sinsch U, Oromi N, Sanuy D. Growth marks in natterjack toad (*Bufo calamita*) bones: histological correlates of hibernation and aestivation periods. 2007;9.

49. Canoville A, Chinsamy A. Bone Microstructure of the Stereospondyl *Lydekkerina Huxleyi* Reveals Adaptive Strategies to the Harsh Post Permian-Extinction Environment. Anat Rec. 2015 Jul;298(7):1237–54.

50. Mchugh JB. Paleohistology of *Micropholis stowi* (Dissorophoidea) and *Lydekkerina huxleyi* (Lydekkerinidae) humeri from the Karoo Basin of South Africa, and implications for bone microstructure evolution in temnospondyl amphibians. J Vertebr Paleontol. 2015 Jan 2;35(1):e902845.

51. Ray S, Chinsamy A, Bandyopadhyay S. *Lystrosaurus murrayi* (therapsida, Dicynodontia): Bone Histology, Growth and Lifestyle Adaptations. Palaeontology. 2005 Nov 1;48(6):1169–85.

52. Yuan PL, Young CC. On the Occurrence of *Lystrosaurus* in Sinkiang. Bull Geol Soc China. 1934;13(1):575–80.

53. Young CC. On two skeletons of Dicynodontia. Bull Geol Soc China. 1935;(14):483–517.

54. Young CC. Additional Dicynodontia remains from Sinkiang. Bull Geol Soc China. 1939;(19):111–36.

55. Sun A-L. Preliminary report on a new species of *Lystrosaurus* of Sinkiang. Vertebr Pal Asiat. 1964;8:216–7.

56. Sun A-L. Permo-Triassic dicynodonts from Turfan, Sinkiang. Permo-Triassic Vertebr Foss Turfan Basin. 1973;53–68.

57. Cheng Z. Vertebrates. In: Permian and Triassic Strata and Fossil Assemblages in the Dalongkou Area of Jimsar, Xinjiang. 1986. p. 207–18. (People’s Republic of China Ministry of Geology and Mineral Resources Geological Memoirs).

58. Cluver MA. The cranial morphology of the dicynodont genus *Lystrosaurus* [Doctoral dissertation]. [Stellenbosch]: Stellenbosch University; 1970.

59. Cosgriff JW, Hammer WR, Ryan WJ. The Pangaean Reptile, *Lystrosaurus maccaigi*, in the Lower Triassic of Antarctica. J Paleontol. 1982;56(2):371–85.

60. King G. Anomodontia. Encycl Paleoherpetology. 1988;1–74.

61. Lucas SG. Chinese Fossil Vertebrates. Columbia University Press; 2001. 390 p.

62. Li J, Sun A. Subclass Synapsida. In: The Chinese Fossil Reptiles and Their Kin. Sci. Press; 2008. p. 379–409.

63. Fröbisch J. Composition and similarity of global anomodont-bearing tetrapod faunas. Earth-Sci Rev. 2009 Aug;95(3–4):119–57.

64. Camp J, Liu J. The taxonomy and cranial morphology of Chinese *Lystrosaurus*. J Vertebr Paleontol. 2011;31(3).

65. Camp J. Morphological variation and disparity in *Lystrosaurus*. University of Iowa; 2010.

66. Rohlf FJ. tpsDig. Stony Brook, NY: Department of Ecology and Evolution, State University of New York at Stony Brook; 2010.

67. Kammerer CF, Deutsch M, Lungmus JK, Angielczyk KD. Effects of taphonomic deformation on geometric morphometric analysis of fossils: a study using the dicynodont *Diictodon feliceps* (Therapsida, Anomodontia). PeerJ. 2020 Oct 7;8:e9925.

68. Adams D, Collyer M, Kaliontzopoulou A, Sherratt E. Geomorph: Software for geometric morphometric analyses. 2016.

69. Adams DC, Collyer ML. Multivariate Phylogenetic Comparative Methods: Evaluations, Comparisons, and Recommendations. Syst Biol. 2018 Jan 1;67(1):14–31.

70. Schlager S. Morpho and Rvcg – Shape Analysis in R: R-Packages for Geometric Morphometrics, Shape Analysis and Surface Manipulations. In: Zheng G, Li S, Székely G, editors. Statistical Shape and Deformation Analysis. Academic Press; 2017. p. 217–56. Available from: https://www.sciencedirect.com/science/article/pii/B9780128104934000110

71. Yang W, Feng Q, Liu Y, Tabor N, Miggins D, Crowley JL, et al. Depositional environments and cyclo- and chronostratigraphy of uppermost Carboniferous–Lower Triassic fluvial–lacustrine deposits, southern Bogda Mountains, NW China — A terrestrial paleoclimatic record of mid-latitude NE Pangea. Glob Planet Change. 2010 Aug;73(1–2):15–113.

72. Yang W, Wan M, Crowley JL, Wang J, Luo X, Tabor NJ, et al. Paleoenvironmental and paleoclimatic and cyclo- and chrono-stratigraphy of upper Permian–Lower Triassic fluvio-lacustrine deposits in Bogda Mountains, NW China – implications for diachronous plant evolution across Permian-Triassic boundary. Earth-Sci Rev. :Forthcoming.

73. Lamm E-T. 4. Preparation and Sectioning of Specimens. Bone Histology of Fossil Tetrapods. University of California Press; 2013.

74. Holliday CM, Ridgely RC, Sedlmayr JC, Witmer LM. Cartilaginous Epiphyses in Extant Archosaurs and Their Implications for Reconstructing Limb Function in Dinosaurs. Farke AA, editor. PLoS ONE. 2010 Sep 30;5(9):e13120.

75. Griffin CT, Stefanic CM, Lessner EJ, Riegler M, Formoso K, Koeller K, et al. Assessing ontogenetic maturity in extinct saurian reptiles. Biol Rev. 2020;56.

76. de Ricqlès A. Recherches paleohistologiques sur les os longs des tetrapodes; II, Quelques observations sur la structure des os longs des theriodontes. Ann Pale´ontologie. 1969;55(1):1–51.

77. Ray S, Botha J, Chinsamy A. Bone histology and growth patterns of some nonmammalian therapsids. J Vertebr Paleontol. 2004 Sep 10;24(3):634–48.

78. Ray S, Bandyopadhyay S, Bhawal D. Growth patterns as deduced from bone microstructure of some selected neotherapsids with special emphasis on dicynodonts: Phylogenetic implications. Palaeoworld. 2009 Mar;18(1):53–66.

79. Ray S, Botha-Brink J. Dicynodont Growth Dynamics and Lifestyle Adaptations. In: Forerunners of Mammals. Indiana University Press; 2012. p. 29.

80. Olivier C, Houssaye A, Jalil N-E, Cubo J. First palaeohistological inference of resting metabolic rate in an extinct synapsid, *Moghreberia nmachouensis* (Therapsida: Anomodontia). Biol J Linn Soc. 2017 Jun 1;121(2):409–19.

81. Mori R, Kodaka T, Sano T, Yamagishi N, Asari M, Naito Y. Comparative Histology of the Laminar Bone between Young Calves and Foals. Cells Tissues Organs. 2003;175(1):43–50.

82. Currey JD. Differences in the Blood-Supply of Bone of Different Histological Types. J Cell Sci. 1960 Sep 1;s3-101(55):351–70.

83. Currey JD. The many adaptations of bone. J Biomech. 2003 Oct;36(10):1487–95.

84. de Margerie E, Cubo J, Castanet J. Bone typology and growth rate: testing and quantifying ‘Amprino’s rule’ in the mallard (*Anas platyrhynchos*). C R Biol. 2002 Mar 1;325(3):221–30.

85. Urbanek A. Biotic crises in the history of Upper Silurian graptoloids: A Palaeobiological model. Hist Biol. 1993 Jul;7(1):29–50.

86. Harries PJ, Knorr PO. What does the ‘Lilliput Effect’ mean? Palaeogeogr Palaeoclimatol Palaeoecol. 2009 Dec;284(1–2):4–10.

87. Huttenlocker AK. Body Size Reductions in Nonmammalian Eutheriodont Therapsids (Synapsida) during the End-Permian Mass Extinction. PLOS ONE. 2014 Feb 3;9(2):e87553.

88. Huttenlocker AK, Botha-Brink J. Body size and growth patterns in the therocephalian *Moschorhinus kitchingi* (Therapsida: Eutheriodontia) before and after the end-Permian extinction in South Africa. Paleobiology. 2013;39(2):253–77.

89. Grine FE, Forster CA, Cluver MA, Georgi JA. Cranial Variability, Ontogeny, and Taxonomy of *Lystrosaurus* from the Karoo Basin of South Africa. In: Amniote Paleobiology. University of Chicago Press; 2006. p. 432–506.

90. Botha J, Smith RMH. *Lystrosaurus* species composition across the Permo–Triassic boundary in the Karoo Basin of South Africa. Lethaia. 2007;40(2):125–37.

91. Smith RMH, Botha-Brink J. Anatomy of a mass extinction: Sedimentological and taphonomic evidence for drought-induced die-offs at the Permo-Triassic boundary in the main Karoo Basin, South Africa. Palaeogeogr Palaeoclimatol Palaeoecol. 2014 Feb;396:99–118.

92. Botha J, Huttenlocker AK, Smith RMH, Prevec R, Viglietti P, Modesto SP. New geochemical and palaeontological data from the Permian-Triassic boundary in the South African Karoo Basin test the synchronicity of terrestrial and marine extinctions. Palaeogeogr Palaeoclimatol Palaeoecol. 2020 Feb;540:109467.

93. Kitching JW. The distribution of the Karroo vertebrate fauna: with special reference to certain genera and the bearing of this distribution on the zoning of the Beaufort Beds. University of the Witwatersrand; 1977. 1–131 p.

94. Kammerer CF, Viglietti PA, Hancox PJ, Butler RJ, Choiniere JN. A new kannemeyeriiform dicynodont (*Ufudocyclops mukanelai*, gen. et sp. nov.) from Subzone C of the *Cynognathus* Assemblage Zone, Triassic of South Africa, with implications for biostratigraphic correlation with other African Triassic faunas. J Vertebr Paleontol. 2019;39(2):e1596921.

95. Scotese CR, Wright N. PALEOMAP paleodigital elevation models (PaleoDEMS) for the Phanerozoic. 2018. Available from: https://www.earthbyte.org/paleodem-resource-scotese-and-wright-2018/

96. Scotese CR. An Atlas of Phanerozoic Paleogeographic Maps: The Seas Come In and the Seas Go Out. Annu Rev Earth Planet Sci. 2021 May 30;49(1):annurev-earth-081320-064052.

97. Kidder DL, Worsley TR. Causes and consequences of extreme Permo-Triassic warming to globally equable climate and relation to the Permo-Triassic extinction and recovery. Palaeogeogr Palaeoclimatol Palaeoecol. 2004 Feb;203(3–4):207–37.

98. Kiehl JT, Shields CA. Climate simulation of the latest Permian: Implications for mass extinction. Geology. 2005;33(9):757.

99. Winguth AME, Shields CA, Winguth C. Transition into a Hothouse World at the Permian–Triassic boundary—A model study. Palaeogeogr Palaeoclimatol Palaeoecol. 2015 Dec 15;440:316–27.

100. Rey K, Amiot R, Fourel F, Abdala F, Fluteau F, Jalil N-E, et al. Oxygen isotopes suggest elevated thermometabolism within multiple Permo-Triassic therapsid clades. Elife. 2017;6:e28589.

101. Winguth A, Winguth C. Precession-driven monsoon variability at the Permian–Triassic boundary — Implications for anoxia and the mass extinction. Glob Planet Change. 2013 Jun 1;105:160–70.

102. Tabor NJ, Sidor CA, Smith RMH, Nesbitt SJ, Angielczyk KD. Paleosols of the Permian-Triassic: proxies for rainfall, climate change and major changes in terrestrial tetrapod diversity. J Vertebr Paleontol. 2017 Nov 29;37(sup1):240–53.

103. Sun Y, Joachimski MM, Wignall PB, Yan C, Chen Y, Jiang H, et al. Lethally Hot Temperatures During the Early Triassic Greenhouse. Science. 2012 Oct 19;338(6105):366–70.

104. Frank TD, Fielding CR, Winguth AME, Savatic K, Tevyaw A, Winguth C, et al. Pace, magnitude, and nature of terrestrial climate change through the end-Permian extinction in southeastern Gondwana. Geology. 2021 May 19; Available from: https://doi.org/10.1130/G48795.1

105. Smith RMH. Changing fluvial environments across the Permian-Triassic boundary in the Karoo Basin, South Africa and possible causes of tetrapod extinctions. Palaeogeogr Palaeoclimatol Palaeoecol. 1995 Aug;117(1–2):81–104.

106. Ward PD. Altered River Morphology in South Africa Related to the Permian-Triassic Extinction. Science. 2000 Sep 8;289(5485):1740–3.

107. Viglietti PA, Smith RMH, Rubidge BS. Changing palaeoenvironments and tetrapod populations in the *Daptocephalus* Assemblage Zone (Karoo Basin, South Africa) indicate early onset of the Permo-Triassic mass extinction. J Afr Earth Sci. 2018 Feb;138:102–11.

108. Gastaldo RA, Kamo SL, Neveling J, Geissman JW, Looy CV, Martini AM. The base of the *Lystrosaurus* Assemblage Zone, Karoo Basin, predates the end-Permian marine

extinction. Nat Commun. 2020 Dec;11(1). Available from: http://www.nature.com/articles/s41467-020-15243-7

109. Roopnarine PD, Angielczyk KD. Community stability and selective extinction during the Permian-Triassic mass extinction. Science. 2015 Oct 2;350(6256):90–3.

110. Metcalfe I, Foster CB, Afonin SA, Nicoll RS, Mundil R, Xiaofeng W, et al. Stratigraphy, biostratigraphy and C-isotopes of the Permian–Triassic non-marine sequence at Dalongkou and Lucaogou, Xinjiang Province, China. J Asian Earth Sci. 2009 Nov;36(6):503–20.

111. Wan M, Yang W, Wang J. *Amyelon bogdense* sp. nov., a silicified gymnospermous root from the Changhsingian–Induan (?) in southern Bogda Mountains, northwestern China. Rev Palaeobot Palynol. 2019 Apr;263:12–27.

112. Wan M-L, Yang W, Wang J. *Palaeocupressinoxylon uniseriale* n. gen. n. sp., a gymnospermous wood from the upper Permian of Central Taodonggou, southern Bogda Mountains, northwestern China. Palaeoworld. 2020 Mar;29(1):117–25.

Fig1

Click here to access/download;Figure;Fig1.tif

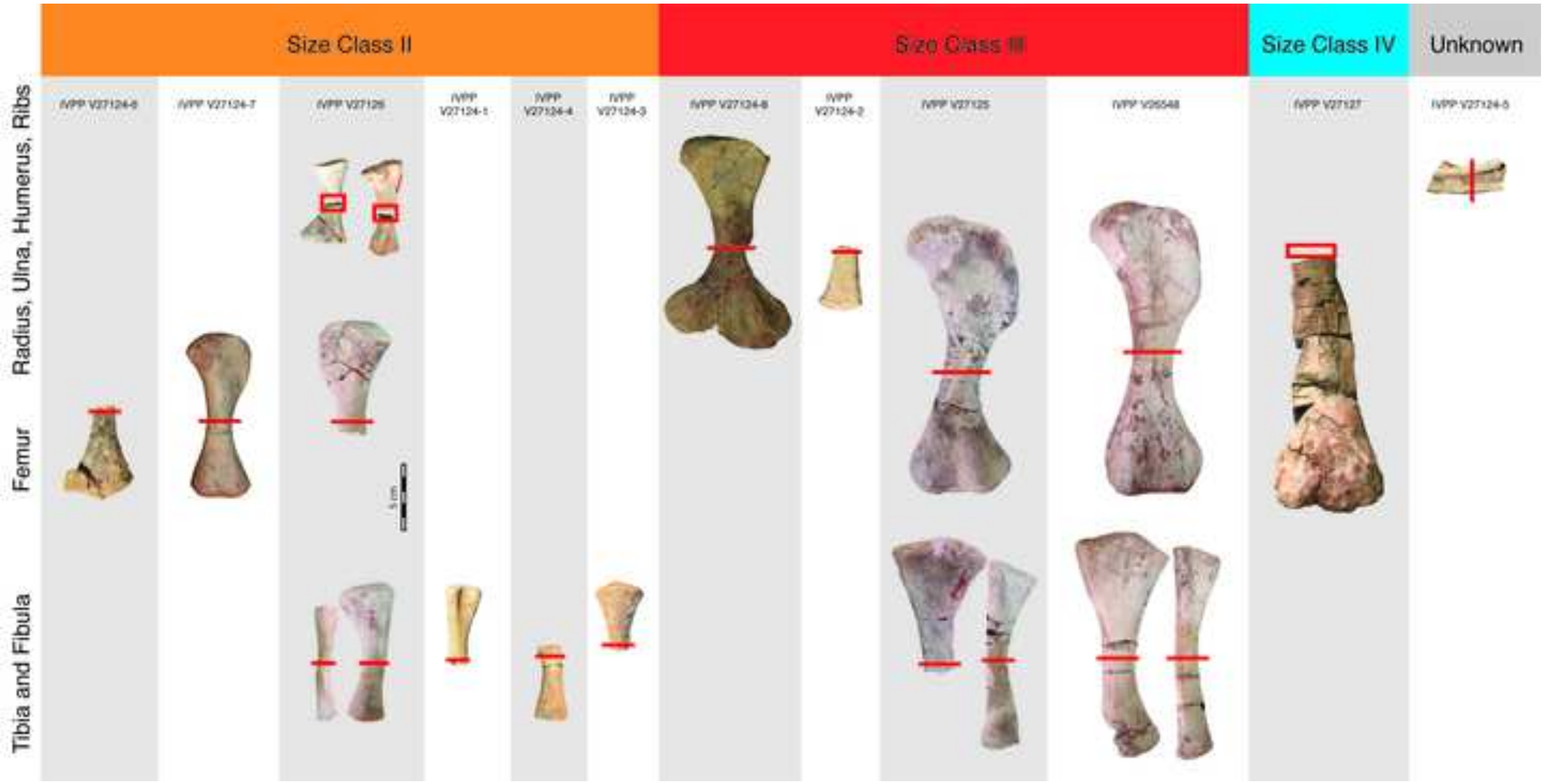

Click here to access/download;Figure;Fig2.tiff

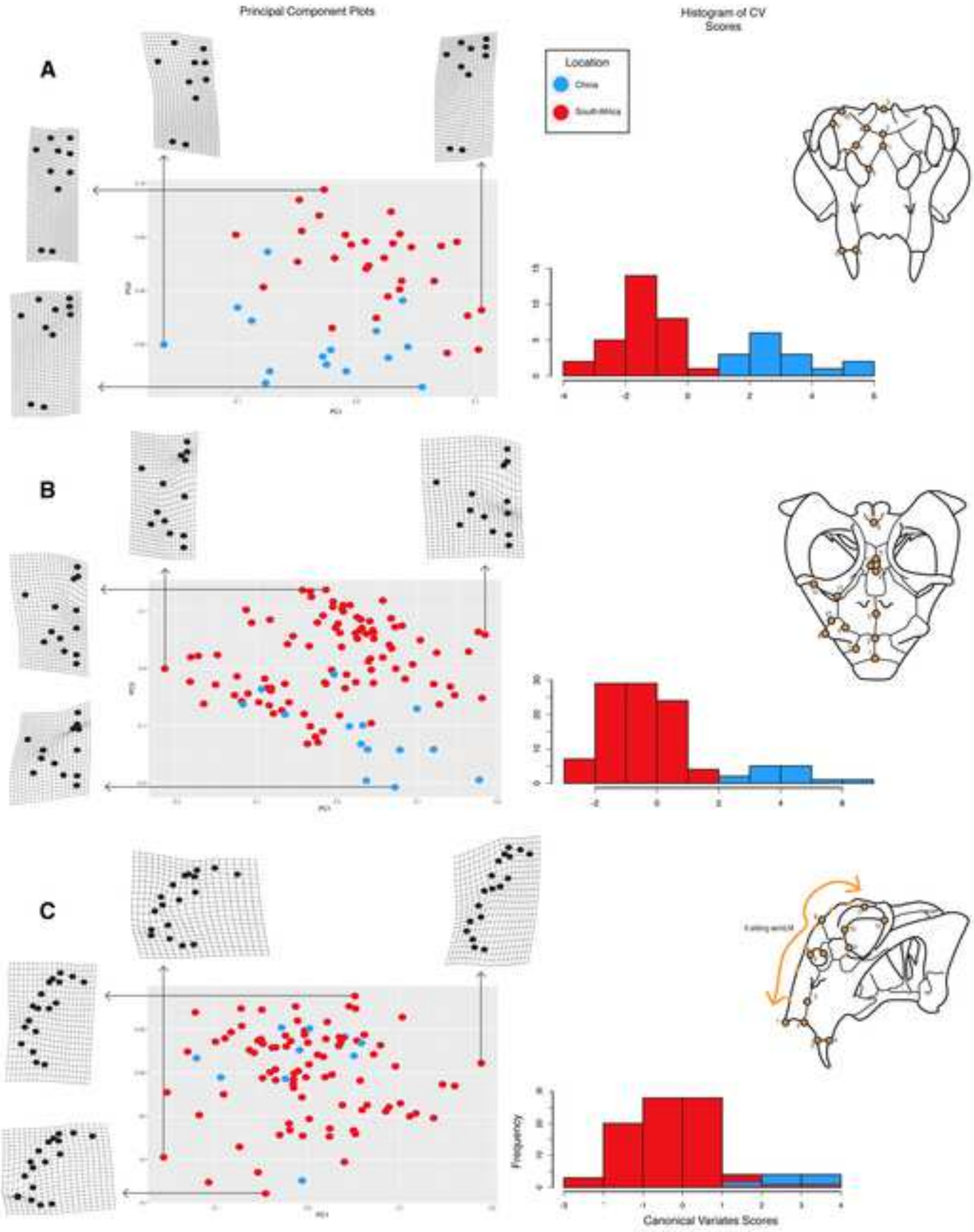

Fig8 Click here to access/download;Figure;Fig8.tiff

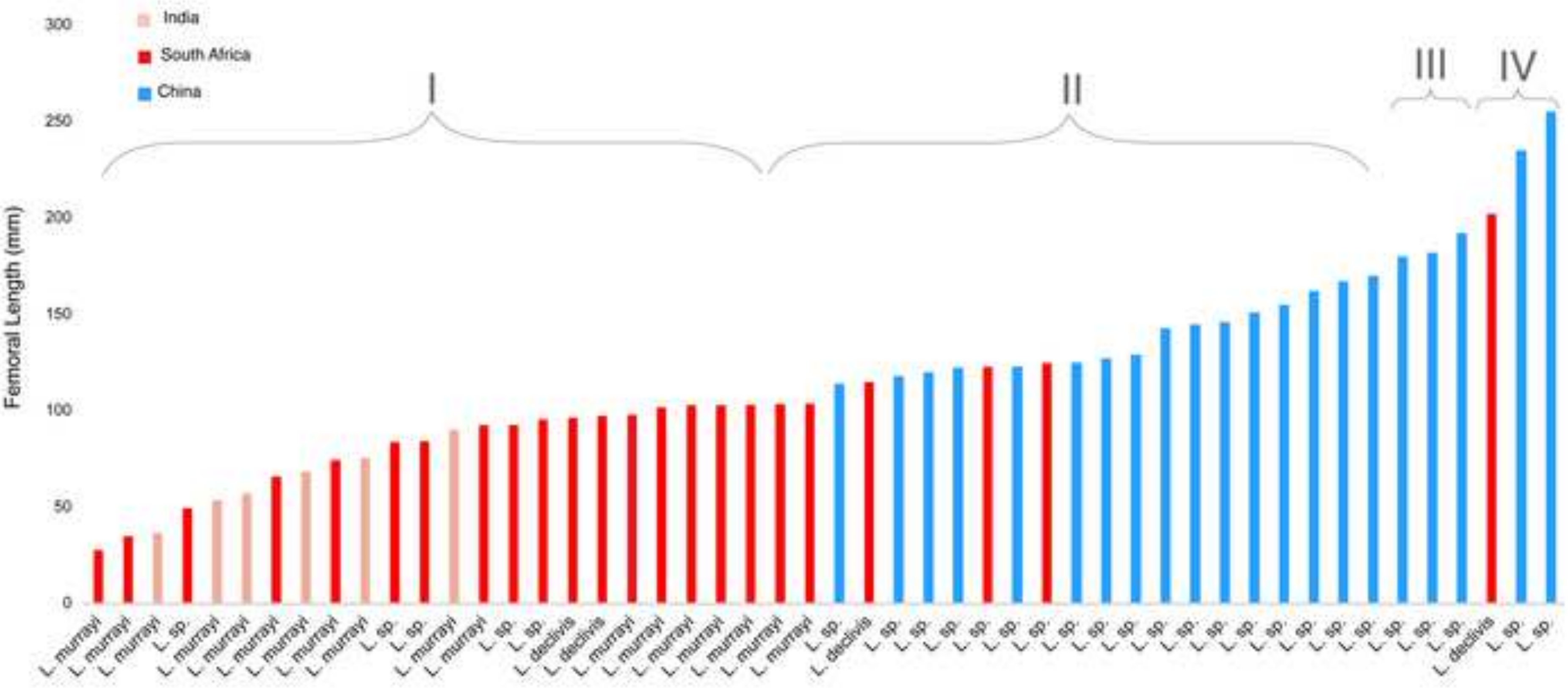

Fig 7

Click here to access/download;Figure;Fig7.tiff

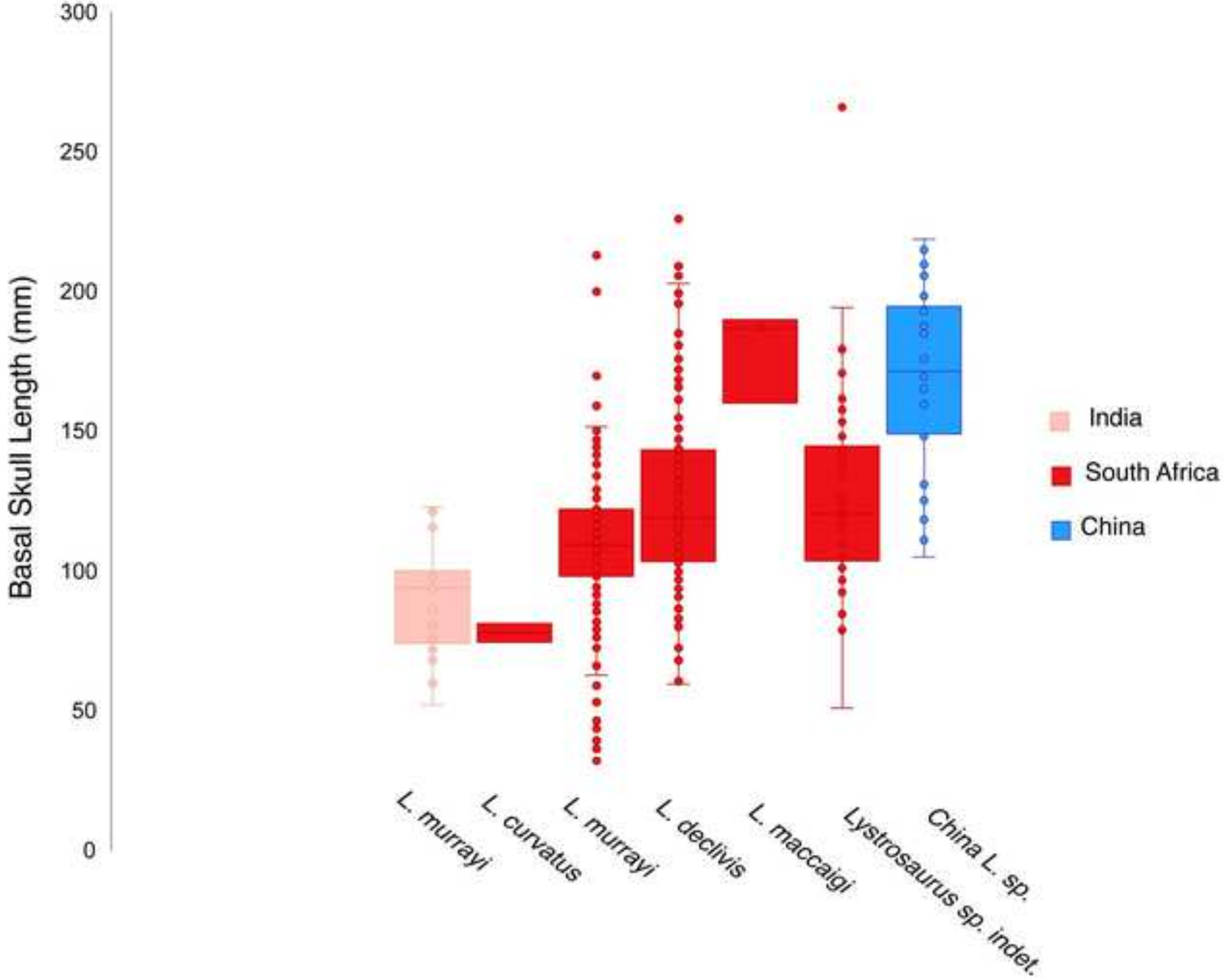

Fig 3

Click here to access/download;Figure;Fig3.tiff

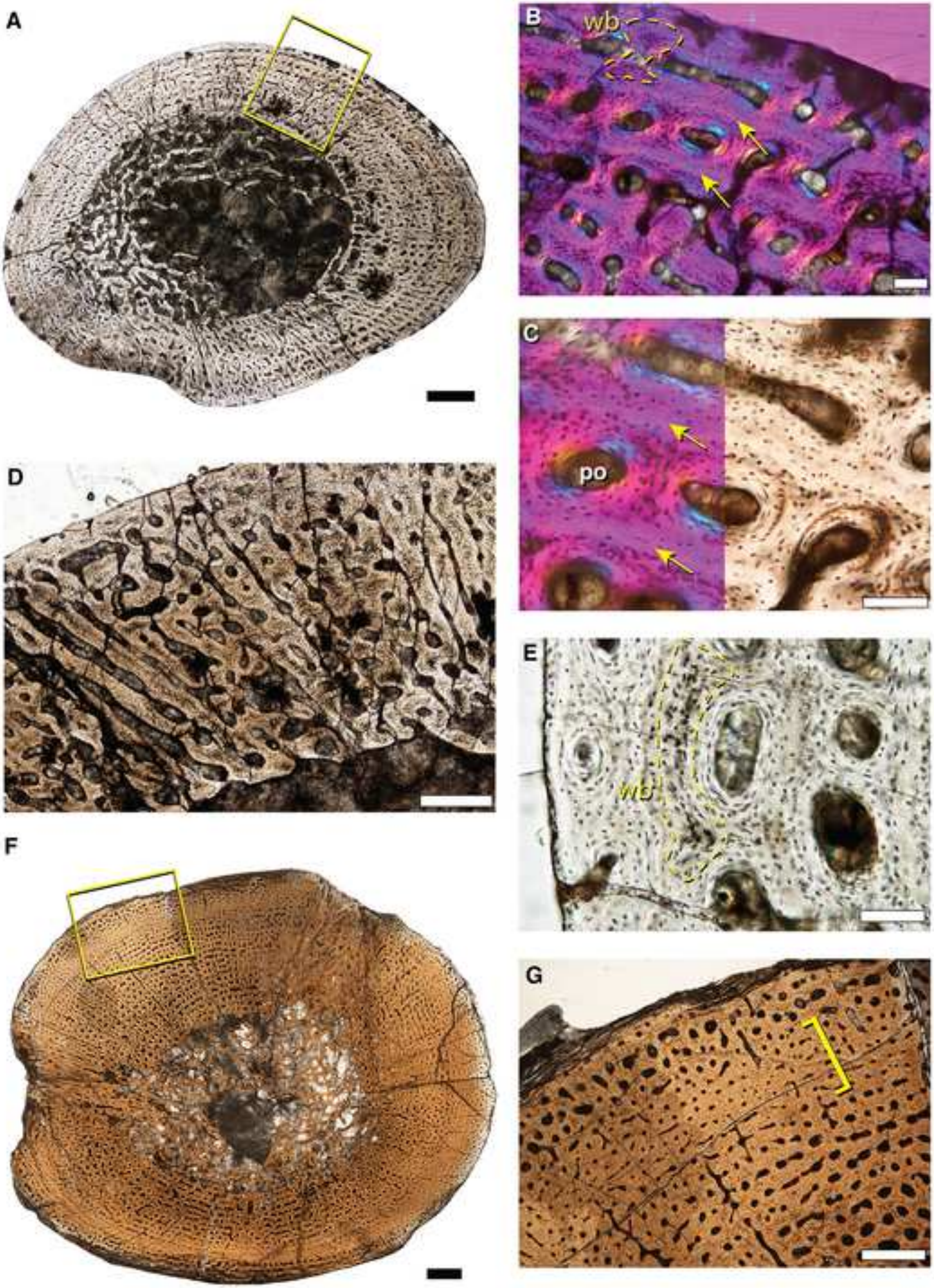

Fig 5

Click here to access/download;Figure;Fig5.tiff

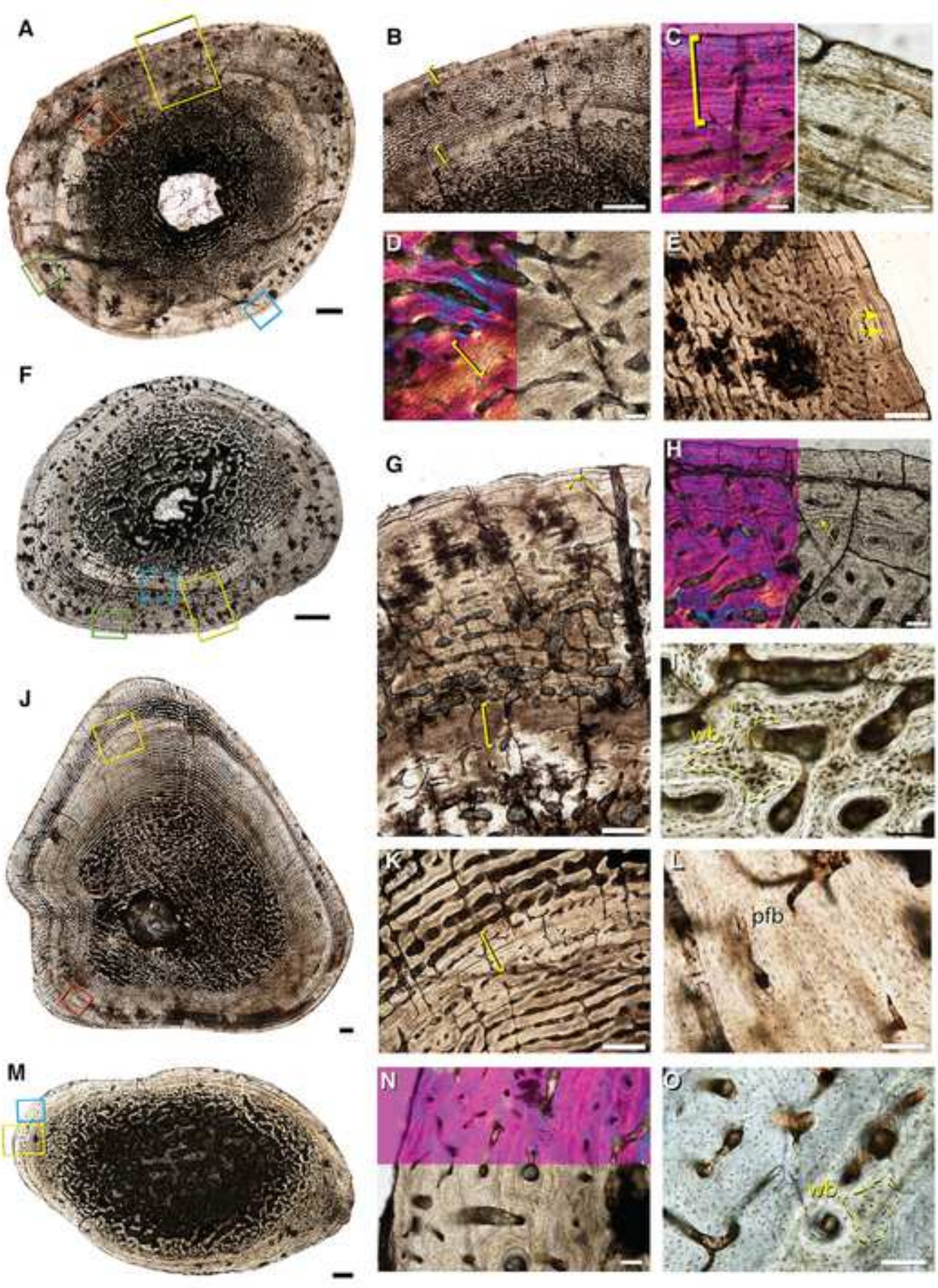

Fig 6

Click here to access/download;Figure;Fig6.tiff

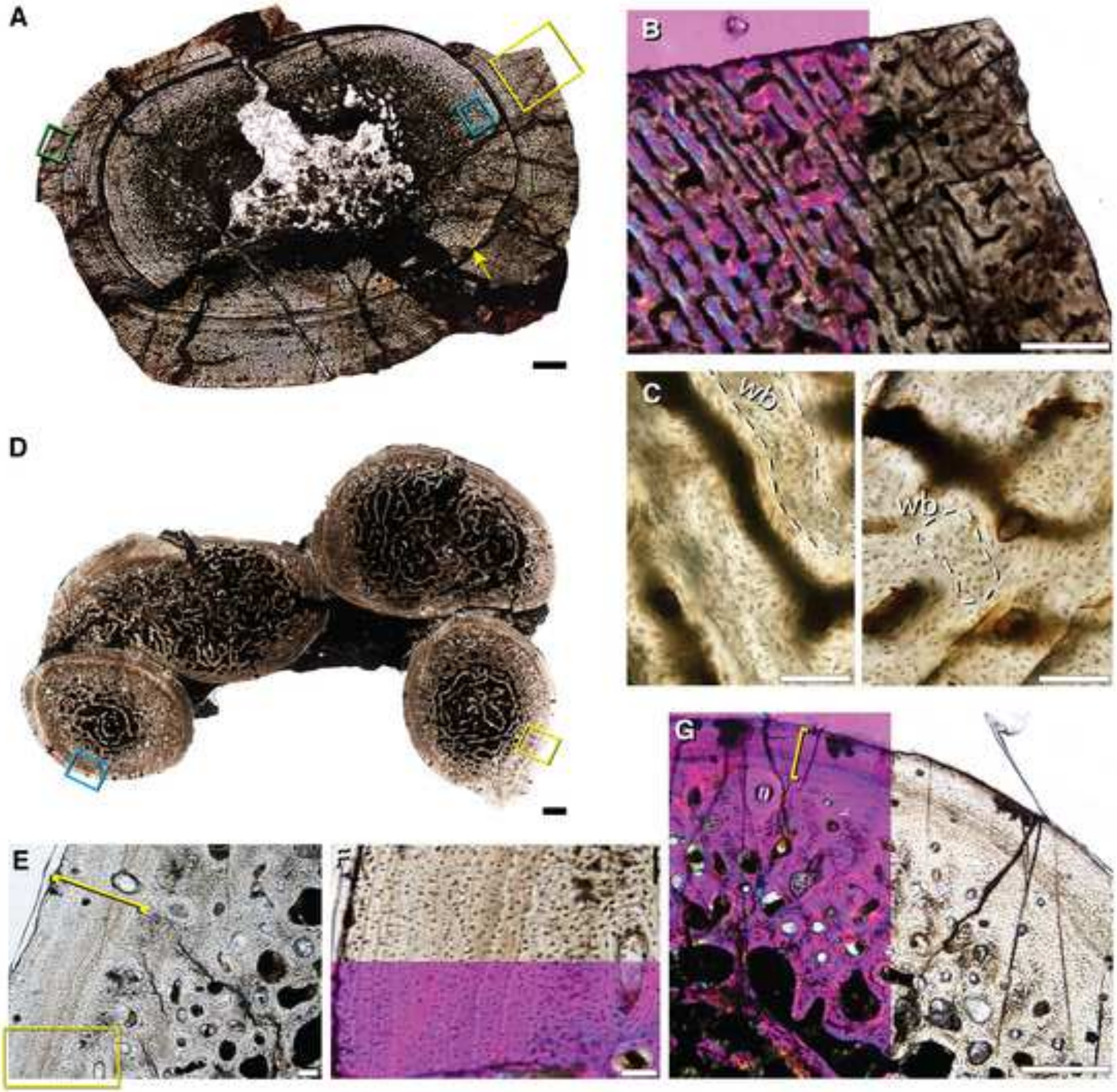

Fig 4 Click here to access/download;Figure;Fig4.tiff

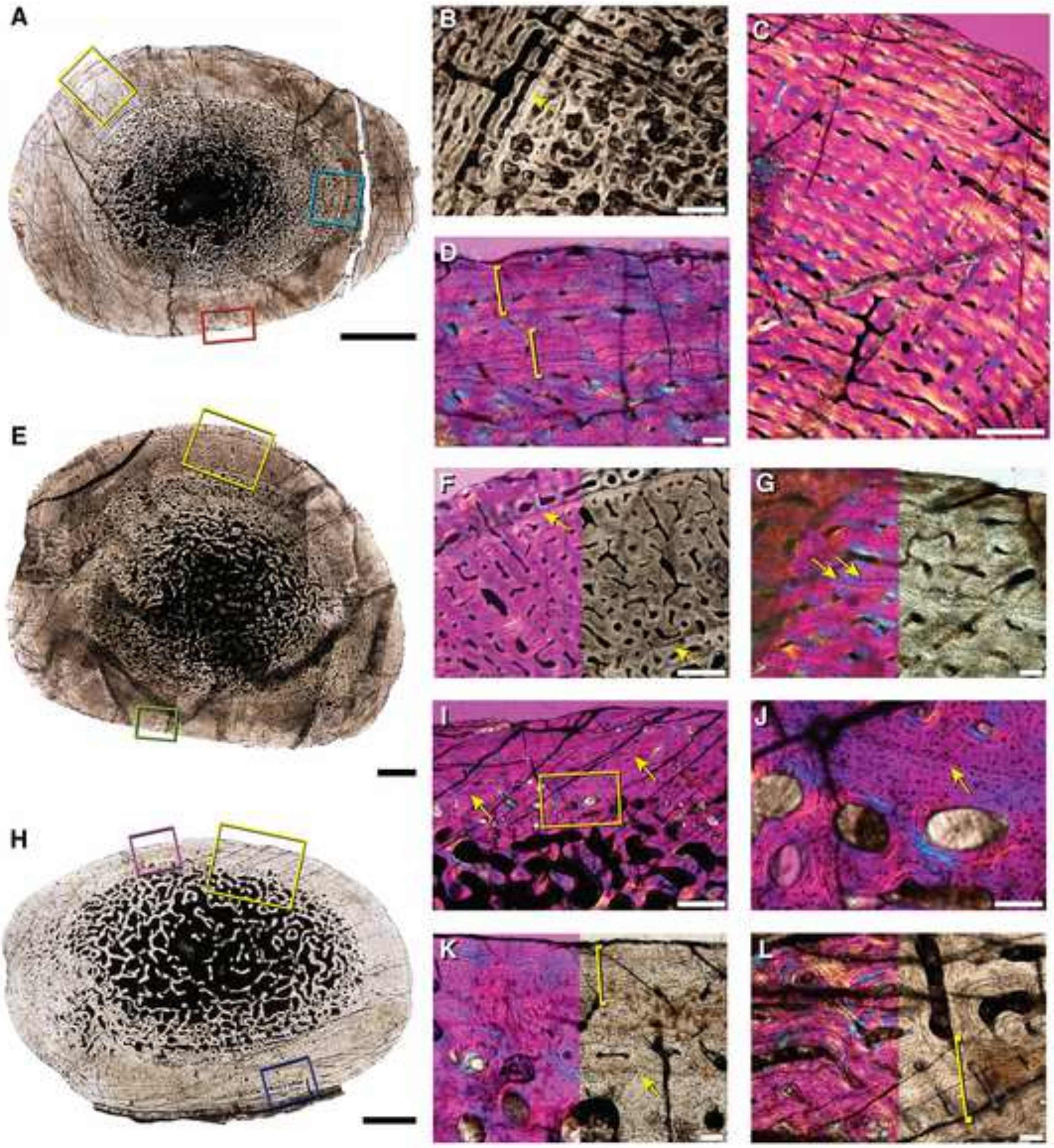

S1 Supporting Information

Click here to access/download
Supporting Information
S1 Supporting Information.pdf

S2 Dataset

Click here to access/download
Supporting Information
S2 Dataset.xlsx

Living Fast in the Triassic: New data on life history in *Lystrosaurus* (Therapsida: Dicynodontia) from northeastern Pangea

Short Title: Life history in *Lystrosaurus* from northeastern Pangea

Zoe T. Kulik[1*], Jacqueline K. Lungmus[2], Kenneth D. Angielczyk[3], and Christian A. Sidor[1]

[1] Department of Biology and Burke Museum, University of Washington, Seattle, WA, United States of America

[2] Smithsonian National Museum of Natural History Washington, D.C., United States of America

[3]Negaunee Integrative Research Center, Field Museum of Natural History, Chicago, IL, United States of America

* Corresponding author

E-mail: zkulik@uw.edu

Abstract

Lystrosaurus was one of the few tetrapods to survive the Permo-Traissic mass extinction, the most profound biotic crisis in Earth's history. The wide paleolatitudinal range and high abundance of *Lystrosaurus* during the Early Triassic provide a unique opportunity to investigate changes in growth dynamics and longevity following the mass extinction, yet most studies have focused only on species that lived in the southern hemisphere. Here, we present the long bone histology from twenty *Lystrosaurus* skeletal elements spanning a range of sizes that were collected in the Jiucaiyuan Formation of northwestern China. In addition, we compare the average body size of northern and southern Pangean Triassic-aged species and conduct cranial geometric morphometric analyses of southern and northern taxa to begin investigating whether specimens from China are likely to be taxonomically distinct from South African specimens. We demonstrate that *Lystrosaurus* from China have larger average body sizes than their southern Pangean relatives and that cranial morphologies are distinctive. The osteohistological examination revealed sustained, rapid osteogenesis punctuated by growth marks in some, but not all, immature individuals from China. We find that the osteohistology of Chinese *Lystrosaurus* shares a similar growth pattern with South African species that show sustained growth until death. However, bone growth arrests more frequently in the Chinese sample. Nevertheless, none of the long bones sampled here indicate that maximum or asymptotic size was reached, suggesting that the maximum size of *Lystrosaurus* from the Jiucaiyuan Formation remains unknown.

Introduction

The Permo-Triassic mass extinction was the most devastating biotic crisis in Earth's history and caused communities to collapse in both the terrestrial and marine realms (1-3]. Fluctuating climates accompanied by an overall increase in global temperatures are hypothesized to have forced 70% of terrestrial vertebrate families to extinction [4-8], although plant extinctions were notably damped [9-11]. Recovery after the Permo-Triassic extinction has been a topic of intense research, but most studies in the terrestrial realm focus on taxonomic diversity and abundance [12-14], or changes in community structure and dynamics following the extinction [6, 3, 15-18]. Few studies have examined how the extremely changed environment of the earliest Triassic impacted growth rate, life history, and longevity of surviving taxa [19-22]. Importantly, most of the studies noted above rely heavily—if not exclusively—on data from the southern hemisphere, in particular the Karoo Basin of South Africa.

Lystrosaurus, a stoutly built dicynodont therapsid that ranged in maximum skull size from approximately 16–39 cm (*L. murrayi* and *L. maccaigi* from South Africa, respectively), is one of the hallmark survivors of the Permo-Triassic extinction due to its remarkable abundance in southern Pangean deposits (23-24, 18]. The success of *Lystrosaurus* has been variously attributed to its generalist diet of tough plant material (e.g., [25, 26]), broad habitat tolerances [27], a burrowing lifestyle [28], unusual thermal tolerances [29], and a developmentally plastic growth strategy that allowed it to weather extreme ecosystem instability [30, 20, 22, 31]. These hypothesized advantages/exaptations for a post-extinction environment have been studied almost exclusively using specimens from South Africa, but *Lystrosaurus* is known from across Pangea: including Permo-Triassic aged deposits in Antarctica, Mongolia, Russia, China, India, and possibly Mozambique (e.g., [32-39]). The wide paleolatitudinal range of *Lystrosaurus* offers a

rare opportunity to investigate differences in lifespan, survivorship, and body size in southern and northern Pangean populations that experienced markedly different environmental conditions in the wake of the Permo-Triassic extinction (e.g., [22, 40]).

Osteohistological Perspectives on Life History

Bone histology provides a wealth of information about an extinct animal’s growth and life history. Mineralized bone matrix, vascular canal spaces, and cellular spaces persist in fossilized bone, making it possible to determine the relative rate of bone deposition as well as maturity of extinct organisms [41]. Cyclical ~~slowdowns~~ in skeletal growth rate leave histological markers, termed growth marks, in primary cortical bone. Annuli and lines of arrested growth (LAGs) are two types of cyclical growth marks that are deposited in annual or seasonal cycles [42-44]. Annuli represent periodic decreases in growth rate and are usually identified based on specific, contextual changes in the bone tissue architecture, such as a dramatic decrease of vascular and cellular density, or a temporary shift to parallel-fibered or lamellar bone. LAGs, on the other hand, represent periodic cessations in growth and appear as hyperminerlized lines in the primary cortical bone of most vertebrates when bone deposition stops in unfavorable seasons [44-49, 42]. In addition to extrinsically (i.e., environmentally or seasonally) induced growth marks, growth can also arrest during periods of intense stress, such as birth, and during modulations to metabolic activity such as torpor [50, 51]. The periodicity of growth marks (i.e., the extent to which they are regularly spaced throughout the cortex of large, presumably mature individuals) helps to determine whether they represent extrinsic or intrinsic events.

Botha [22] recently conducted a detailed examination of growth and life history patterns in four species of *Lystrosaurus* across the Permian-Triassic boundary using fossils from South

Africa. Using a combination of bone histology and body size data, Botha [22] found that Triassic *Lystrosaurus* individuals grew rapidly and had increased mortality at small sizes, as evidenced by the overwhelming abundance of small skulls known from Triassic rocks in the Karoo Basin. This post-extinction change in life expectancy is also inferred from the scarcity of LAGs and annuli in the rapidly deposited cortical bone of Triassic specimens. In comparison, Permian individuals had larger average skull sizes and had numerous growth marks spaced throughout the cortex, suggesting prolonged periods of growth spanning multiple seasons or years [22]. Similar patterns were previously found across a wider taxonomic range of South African therapsid species [20] and lydekkerinid stereospondyls [52, 53]. The predominance of small *Lystrosaurus* (and other tetrapods) with rapidly deposited bone tissue suggests an overall shift in life history strategy across the Permian-Triassic boundary to an early onset of reproductive maturity [20, 52, 53].

Histological data for *Lystrosaurus* outside of the South African Karoo Basin are more limited, but critical to understanding the relationship between latitude (and by extension climate) and life history. Bones of *Lystrosaurus* from the Early Triassic of India lack LAGs and annuli, and have small skulls, consistent with the pattern seen in South Africa [37, 54]. A preliminary report of the bone histology from *Lystrosaurus* from the Turpan-Hami Basin of China suggested that subadult individuals record multiple LAGs [40]. However, in the absence of comparative body size data, it remains unclear if multiple LAGs in the Chinese specimens indicate prolonged periods of growth (i.e., multiple cycles or seasons of growth) or environmental instability (e.g., disruptions to the metabolic rate as a result of resource limitations). Here, we analyze bone histology from a size range of *Lystrosaurus* postcrania from the Jiucaiyuan Formation of Xinjiang, China (Fig 1) as well as body size data across Triassic-aged *Lystrosaurus* assemblages

to investigate whether *Lystrosaurus* from northern Pangea had differing life histories than its southern Pangean relatives.

Figure 1. Size distribution of *Lystrosaurus* sp. postcrania from the Jiucaiyuan formation included in the histologic analysis. Twenty skeletal elements, with gray bars indicating associations of individuals, span three size classes inferred from femoral length: Size Class II spans 47–63% maximum femoral size, Size Class III includes specimens that are ~ 70% maximum size, and Size Class III includes the largest partial femur. Scale bar = 5 cm.

Materials and Methods

Geometric Morphometric Analysis of Skull Shape

An important question to consider when comparing the bone histology of *Lystrosaurus* specimens from China and other regions is whether the same species occur in the different geographic areas. Although the first material of *Lystrosaurus* collected in Xinjiang was referred to the South African species *L. murrayi* [55], a total of seven endemic *Lystrosaurus* species also have been described [56-60]. Colbert [33]; also see [61-63], considered some Chinese specimens to represent *L. murrayi* and *L. curvatus,* and others to represent endemic species. More recent authors have been split over whether *Lystrosaurus* specimens from Xinjiang represent exclusively endemic species or a mixture of endemic and more cosmopolitan species, as well as the number of valid endemic species (e.g., [35, 64-67, 40]).

A comprehensive taxonomic revision of *Lystrosaurus* from Xinjiang is beyond the scope of this paper. However, to gain insight into whether Chinese *Lystrosaurus* specimens are likely to be taxonomically distinct from South African specimens, we conducted a preliminary two-

dimensional geometric morphometric analysis of skull shape. The skulls were analyzed in three perspectives - anterior, dorsal, and lateral – following the methodology of [68]; also see [67]. The anterior shape analysis included 45 individual specimens, 15 from China and the remaining 30 from South Africa. The dorsal shape analysis included 107 specimens, 14 from China and 93 from South Africa. Lastly, the lateral analysis included 95 specimens, 9 from China and 86 from South Africa. Landmarks were collected using the tpsDig2ws software [69]. In the case of the anterior and dorsal views, landmarks were digitized on only the left half of the skull. The lateral view digitization included a combination of Type I and Type II landmarks (12 total) with a single curve of six semilandmarks along the outer edge of the snout between landmarks number 1 and 11 (Fig 2). The anterior and dorsal views did not contain any semilandmarks, and have 10 and 13 landmarks, respectively. Because taphonomic deformation can complicate the interpretation of shape variation at low taxonomic levels in dicynodonts [70], data collection focused on specimens that appeared to show relatively little evidence of distortion. See the supplementary files for additional details of the morphometric analysis.

The coordinate data were processed and analyzed further with the Geomorph R package [71], where they underwent a generalized Procrustes superimposition. Principal components analyses were used to visualize major patterns of shape variation, and a Procrustes ANOVA was run on each dataset to assess the statistical relationship between shape and country of origin using the method of [72] and the Geomorph package in R. A canonical variates analysis was run using the CVA function of the Morpho package [73] in R to further explore the association between shape and geographic location, as well as the accuracy with which specimens could be classified using skull shape (see *Results*).

Figure 2. Results of the morphometric analysis comparing specimens of *Lystrosaurus* from China and South Africa. Principal components plots and histogram of canonical variates analysis results for the three perspectives (Anterior – A; Dorsal – B; Lateral – C). Skull drawings on the right correspond to the orientation for each analysis. Orange dots on the specimens show placement of landmarks used in the 2D geometric morphometric analysis (see S1 Supporting Information for more details). Blue dots represent specimens from China and red dots represent specimens from South Africa. Arrows point to the warp grids showing corresponding deformation along each principal component axis. Histograms show frequency of a recovered canonical variate value and show the distinct differences between Chinese and South African specimens, with overlap only occurring in a few specimens in the lateral orientation.

Histological Sample

Twenty skeletal elements (humerus, femur, tibia, radius, ulna, fibula, and ribs; Table 1; Fig 1) of *Lystrosaurus* sp. Were selected for consumptive analysis from the collections of the Institute of Vertebrate Paleontology and Paleoanthropology (IVPP). The majority of the fossils were recovered from strata approximately 30–50 meters above the base of the Jiucaiyuan Formation in the South Taodonggou area, Turpan-Hami Basin, Xinjiang Uygur Autonomous Region, China. One element (IVPP V 27127) came from a nearby locality in Zhaobishan and was recovered about 312m above the base of the Jiucaiyuan Formation [74]. The Jiucaiyuan Formation overlies the Guodikeng Formation lithologically (=upper Wutonggou low-order cycle) and the Wutonggou low-order cycle, and its lower contact is characterized by a facies change from lacustrine to fluvial deposits [75, 74]. This facies change is interpreted as the boundary between the Permian and Triassic [74] and recent work suggests that the Jiucaiyuan

Formation is late Induan-early Olenekian in age [74]. As noted above, the lack of clarity regarding Chinese *Lystrosaurus* taxonomy means that we were unable to assign species-level identifications. However, the majority of our histologic samples come from four individuals. Skeletal elements are differentiated by specimen numbers followed by a letter, in the case of associated individuals (e.g., IVPP V #-a, -b, etc.) The remaining eight specimens are disarticulated elements collected from the same locality and are assigned to one specimen number IVPP V 27124, differentiated by IVPP V 27124-1, -2, etc. In addition to the histologic sample, 50 femora were measured from Early Triassic specimens compiled from the literature [37, 22] and from personal observations to contextualize the range of sizes in this monodominant vertebrate assemblage (S2 Dataset). Additional body size proxies, including basal skull length (S2 Dataset), were measured from *Lystrosaurus* specimens housed in collections at the National Museum (NMQR), the Evolutionary Studies Institute (ESI), the Iziko South African Museum (SAM), the Field Museum of Natural History (FMNH), the American Museum of Natural history (AMNH), and the Burke Museum of Natural History and Culture (UWBM). Cranial and postcranial measurements were made using Neiko digital calipers.

TABLE 1. Postcranial element length and maximum midshaft diameter for the twenty skeletal elements of *Lystrosaurus* sp. from the Jiucaiyuan Formation that were histologically sampled. Asterisk indicates fragmentary or incomplete specimens with accompanying estimates based on complete specimens in this dataset.

**Spec. No.**	**Preserved Length (mm)**	**Estimated Length (mm)**	**Max Midshaft Diameter (mm)**	**Growth Marks**	**Size Class**

**Femur**						
	IVPP V 27124-7	122.38		21.43	0	II
	IVPP V 27124-6	62.74*	129	16.22	0	II
	IVPP V 27126a	62*	162		0	II
	IVPP V 27125a	182		28	2	III
	IVPP V 26548a	192		14.09	2	III
	IVPP V 27127	240*	255	33.5	0	IV
**Tibia**						
	IVPP V 27126b	92			0	II
	IVPP V 27124-4	56.62*	94	15.86	0	II
	IVPP V 27124-3	53.26*	98	15.71	0	II
	IVPP V 26548b	148		10.47	2	III
	IVPP V 27125b	148*	212	10.36	2	III
**Fibula**						
	IVPP V 27124-1	46.37*	86	9.76	0	II
	IVPP V 27126c	86		5.89	0	II
	IVPP V 26548c	140		9.02	2	III

	IVPP V 27125c	140		15.84	2-3	III
**Humerus**						
	IVPP V 27124-8	151.77		23.96	1	III
**Radius**						
	IVPP V 27126d	62			0	II
**Ulna**						
	IVPP V 27126e	75			0	II
	IVPP V 27124-2	47.32*	93	17.52	0	III
**Ribs**						
	IVPP V 27124-5	58.63*	N/A	48.76 - 56.33	EFS?	?

Thin Section Preparation

Thin sections were made following standard osteohistological techniques [76]. Eight of the specimens were sectioned at the University of Washington (UW); the remaining twelve were sectioned at the Institute of Vertebrate Paleontology and Paleoanthropology (IVPP). At the UW, mid-diaphyses were embedded in Epothin Epoxy/Resin 2, sectioned to a thickness of approximately 2 mm on a high-precision diamond-edged saw (Isomet 1000) and glued to glass slides using Devcon 2-Ton epoxy. Slides were ground on a Metaserv 3000 lapidary plate until the specimen was 80 µm thick or until optical clarity was reached. At the IVPP, midshafts were

cut and embedded in polyester resin, sectioned into blocks using an Exact 300CP band saw, and glued to glass slides using Technovit 4000 resin. Slides were ground to optical clarity using an automated lapidary plate (Exakt 400CS). High magnification and composite images were taken using a Nikon Eclipse LV100POL microscope under plain and cross-polarized light with a lambda filter. Composite images were processed using Nikon NIS-Elements AR (version 5.20.02) imaging software. High-resolution images are available at Morphobank under project number P4023.

Ontogenetic maturity was assessed through bone tissue microstructure, articular surface texture and morphology, and size compared to other specimens of *Lystrosaurus* from the Jiucaiyuan Formation. The largest femur, estimated at 255 mm, is of comparable length to the largest known femur (*L. declivis* SAM-PK-K8038) from the Triassic of South Africa, which is 202 mm long. Therefore, we follow the size classes established by Botha [22] for southern Pangean *Lystrosaurus*, but note that our sample lacks Size Class I, or elements less than 40% maximum known size. The remainder of the elements were split into three size categories for analysis (Table 1; Fig 1). Size Class II includes the smallest and presumably most immature specimens in the current sample, with femoral lengths that are approximately 120–160 mm long (47–63% of the maximum femur length observed), tibiae and fibulae that are less than 100 mm long, and associated forelimb elements. All but the proximal tibia fragment (IVPP V 27124-3) have poorly formed joint surfaces and show pitting indicative of epiphyseal cartilage attachment in immature individuals [77]. By comparison, the joint surfaces of elements in the larger size classes are more robustly built and more completely ossified. The relative degree of epiphyseal ossification combined with histological features caused us to deviate slightly from Botha's [22] size classes by grouping specimens that are 40-65% maximum known size as Size Class II,

rather than her 60% MKS cutoff. Our reasoning for this slight change is that the bone tissue is consistently and characteristically immature (i.e., highly vascularized plexiform to radial tissue without LAGs) in all of the associated elements that are 63% MKS. However, the incomplete tibia (IVPP V 27124-3, estimated length of 94 mm) that is estimated to be within Size Class II has a more developed and ossified joint surface compared to similarly sized tibiae (IVPP V 27124-4, 94 mm in length) so it is likely that with more sampling, the split between Size Class II and III will be refined. Size Class III includes immature specimens that are approximately 70% maximum known size (again, based on femoral lengths from larger, complete, Jiucaiyuan specimens). Finally, Size Class IV includes a single femur with an estimated length of 255 mm (the specimen measures 240 mm and is missing the proximal shaft and head of the femur). To our knowledge, it is the largest femur of *Lystrosaurus* yet recovered from the Jiucaiyuan Formation, and is ~ 5 cm longer than the largest femur (discussed above) recovered from the Early Triassic of South Africa. Therefore, it presumably represents the most skeletally mature individual in the current sample. However, histological analysis will provide critical context for the relative maturity of this, and all other specimens sampled [78].

Bone Tissue Terminology

Bone tissue textures seen in *Lystrosaurus* and other therapsids vary widely from highly vascularized and disorganized woven-fibered bone to organized vascular canals in circumferential layers of parallel-fibered to lamellar bone [79-83, 30, 19]. The overall rate of bone growth affects the degree of organization of the extracellular matrix, with mineral fibers in a lamellar organization under slower growth, and disorganized woven-fibered textures under fast growth. Parallel-fibered bone is an intermediate tissue-type but it frequently occurs in the context

of woven-fibered and lamellar bone. When large portions of the cortex consist of parallel-fibered bone, the mineralized extracellular matrix has an alternating anisotropic pattern that looks streaky under cross-polarized light. In many cases, the portion of woven- or parallel-fibered bone that sits between vascular canal spaces is so small that birefringent properties are difficult to discern. When this is the case, it is possible to use the morphology and density of osteocyte lacunae (spaces where bone cells sit in living bone) to approximate the relative organization of mineralized fibers [84, 85]. Woven-fibered bone is statically-derived and includes large, globular, and often densely packed osteocyte lacunae within the mineralized matrix [86, 84]. In contrast, lamellar and parallel-fibered bone are dynamically-derived, and have lenticular or spindle-shaped osteocyte lacunae that are evenly distributed and parallel to the layers of the mineralized matrix [86, 84, 87]. Importantly, in long bone cortices, the fiber network of parallel-fibered bone can either be aligned to the bone's long-axis (i.e., longitudinal fibers) or perpendicular to it (i.e., circular fibers) which can cause differences in lacunar morphology and fiber birefringence in polarized light depending on the plane of section [44]. When parallel fibers are perpendicular to the sectioned plane, they show mass isotropy or monorefringence, similar to woven-fibered bone [85]. Non-lamellar tissues (parallel- and woven-fibered bone) frequently incorporate large vascular spaces that later become infilled with concentric lamellae of parallel-fibered to lamellar bone as primary osteons. The resulting tissue is a fibrolamellar bone complex (sensu Francillon-Vieillot [45]) wherein a disorganized, nonlamellar matrix incorporates a network of centripetally infilled primary osteons. Ricqlès [88] coined much of the terminology used by paleohistologists and used a restricted definition of the fibrolamellar complex to apply only to bone tissue that strictly had woven-fibered matrix, rather than the combination of woven- and parallel-fibered bone used in other applications of the term. More recently, Stein and

Prondvai [85] reinterpreted cortical bone structures using polarized-light microscopy of sauropod thin-sections cut from multiple planes. They concluded that the non-osteonal portion of cortical bone was largely parallel-fibered rather than woven and proposed new terminology for what they interpreted as highly organized primary bone (HOPB). They also proposed an additional term, the woven-parallel complex, to combine the organized portions of HOPB with the more traditional terminology used in bone development and growth (for more, see [89]). The term woven-parallel complex describes a broad range of bone tissues with nonlamellar matrices and the term fibrolamellar complex is maintained as a subset of the woven-parallel complex where the cortical bone is densely vascularized with abundant woven-fibered bone forming the scaffold between vascular canals which is then surrounded by parallel-fibered or lamellar bone [89, 44]. This new terminology recognizes the developmental aspects of osteogenesis and the accumulation of osteoblasts within mineralizing osteoid. However, it may unnecessarily limit the role of woven-fibered bone to a transient tissue type typical of juvenile bone. Woven-fibered bone is known to be present in the typical development, maintenance, and repair of bone tissue throughout ontogeny in vertebrate groups and its abundance in cortical bone may depend on physiological processes rather than development alone [87]. In our histological descriptions below, we follow the recommendation [44, 89] to include lacunar morphology to estimate the proportion of woven- and parallel-fibered bone within the fibrolamellar and woven-parallel complexes and acknowledge the histovariability of parallel- and woven-fibered bone that can occur simultaneously and often in different proportions within the same section forming a spectrum of tissue types that can be difficult to define.

Results

Geometric Morphometric Analysis of Skull Shape

Anterior view: The first five principal components capture 81.41% of the total shape variance in anterior view (PC1 - 30.57%; PC2 - 22.03%; PC3 - 13.82%; PC4 - 8.71%; PC5 - 6.26%) (Fig 2A). Shape is a statistically significant predictor of geographic location for the anterior view dataset ($p = 0.001$), and the canonical variates analysis correctly assigned specimen location 100% of the time.

PC1 primarily describes relative proportions of the snout. The subnarial region of the snout and caniniform process are dorsoventrally shorter relative to the height of the nasals and the anterior orbital margin in specimens with low PC1 scores, and there is greater lateral projection of the prefrontals relative to the caniniform process (Fig 2A). In contrast, the subnarial region and the caniniform process are proportionally much deeper relative to the anterior orbital margin and nasals in specimens with high PC1 scores, and the prefrontals and caniniform process are more closely aligned along a vertical axis. PC2 also captures aspects of skull widening and deepening, although in this case the differences are concentrated in the region of the nasals and prefrontals. Specifically, the prefrontal-nasal region is dorsoventrally shorter in specimens with low PC2 scores, and the dorsal and ventral extrema of the prefrontal are displaced laterally relative to the external naris and the lateral end of the nasofrontal suture. Specimens with high PC2 scores have dorsoventrally taller prefrontal-nasal regions, and the dorsal and ventral extrema of the prefrontal are more closely aligned with the external naris and the lateral edge of the nasofrontal suture. The Chinese and South African specimens are most clearly differentiated along PC2, with the Chinese specimens generally having lower PC2 scores.

Dorsal view: The first five principal components capture 79.75% of the shape variance in dorsal view (PC1 - 26.77%; PC2 - 23.57%; PC3 - 17.95%; PC4 - 6.18%; PC5 - 5.26%) (Fig 2B). Shape is a statistically significant predictor of geographic location for the dorsal view dataset ($p = 0.001$), and the canonical variates analysis correctly assigned specimen location 100% of the time.

Shape variation along PC1 is dominated by differences in the relative lengths of the interorbital region and the pineal foramen, and by the degree of lateral projection of the orbital rim. Specimens with low PC1 scores are characterized by an anteroposteriorly longer interorbital region and a shorter pineal foramen, and an orbital rim that does not project strongly laterally (Fig 2). The pineal foramen is relatively longer anteroposteriorly in specimens with high PC1 scores, and the anterior and posterior corners of the orbital rim (formed by the prefrontal and postorbital bar, respectively) project laterally away from the skull roof. PC2 also captures shape variation associated with relative proportions of the skull roof, as well as orbit shape. Specimens with low PC2 scores have an anteroposteriorly shorter pineal foramen and longer frontals, and the orbit is anteroposteriorly shorter, with anterior and posterior corners that are relatively aligned. The pineal foramen and orbit are relatively longer in specimens with high PC2 scores, and the posterior corner of the orbit (formed by the postorbital bar) projects farther laterally than the anterior orbital margin (formed by the prefrontal). As with the anterior view dataset, the Chinese and South African specimens are most clearly differentiated along PC2, with the Chinese specimens generally having lower PC2 scores.

Lateral view: The first five principal components capture 77.23% of the shape variance in lateral view (PC1 - 32.64%; PC2 - 18.57%; PC3 - 11.66%; PC4 - 8.58%; PC5 - 5.76%) (Fig 2C).

Shape is a statistically significant predictor of geographic location (p = 0.001), and the canonical variates analysis had an overall classification accuracy of 95.78%, misclassifying two Chinese specimens in the dataset.

PC1 describes differences in the profile of the snout, position of the caniniform process relative to the orbit, and the anteroposterior dimensions of the orbit (Fig 2C). Specimens with low PC1 scores have a lower, more anteriorly-convex snout profile with prefrontals that do not project much above the level of the skull roof, and the ventral edge of the caniniform process is located below the anterior orbital margin. The anteroposterior length of the orbit is relatively long. In contrast, specimens with high PC1 scores, have much taller, flatter snout profiles and more dorsally-projecting prefrontals, as well as anteroposteriorly shorter orbits and caniniform processes that are located anterior to the anterior orbital margin. PC2 also captures aspects of snout profile, including the degree of anterior projection of anteroventral corner of the premaxilla and the length of the caniniform process, as well as the dorsoventral height of the orbit. The tip of the premaxilla projects more anteriorly in specimens with low PC2 scores, the caniniform processes are relatively short, and the orbits are dorsoventrally shorter. Specimens with high PC2 scores have taller orbits, flatter snout profiles, and longer caniniform processes. There is little differentiation of the Chinese and South African specimens on the first three PC axes.

Taken together, our morphometric analyses suggest that there are consistent differences in skull shape between *Lystrosaurus* specimens from China and from South Africa, particularly in anterior and dorsal view. Based on these results, we tentatively suggest that the Chinese and South African populations were distinct at the species level. However, because our analyses do not directly address potential synonymies among the named Chinese species, and the fact that

not all of the specimens we sampled histologically are associated with cranial material, we conservatively refer all of our histological samples to *Lystrosaurus* sp.

Osteohistology Overview

Lystrosaurus limb elements, regardless of size, have characteristically thick cortices. Cortical thickness (*K*) measures the proportional diameter of the medullary region relative to the total cross-sectional diameter and remained nearly consistent at 0.63 and 0.64 in the smallest and largest femora in our dataset, respectively (see S1 Supporting Information). Medullary cavities are typically infilled with trabecular bone that gradually transitions to a compact cortex. Similar to the bone histology of other dicynodonts and therapsids more broadly, the dominant bone tissue in all sampled size classes of *Lystrosaurus* is well-vascularized cortical bone made up of varying proportions of woven- and parallel-fibered bone with laminar, plexiform, and longitudinally-oriented primary osteons, indicative of rapid osteogenesis [79, 80-83, 30, 19]. Longitudinal canals that frequently anastomose are arranged in circumferential layers in Size Class II whereas laminar vascular canal orientations are common in Size Class III. In Size Class III and IV, very little of the cortex is remodeled and when secondary osteons are present, they are restricted to the deepest portion of the cortex.

In the small individuals of *Lystrosaurus* studied here, rapidly deposited tissue is interrupted by either LAGs or annuli. These growth marks are found in multiple regions of the cortex, including deep in the cortex and along the sub-periosteal margin. In some individuals, there is variability in the type of growth mark that is deposited among the skeletal elements sampled. For example, the first (i.e., osteologically deeper) growth mark of IVPP V 26548 varies from a LAG in the femur and tibia, to an annulus in the fibula. Interestingly, the largest and

presumably most skeletally mature femur that was histologically sampled, shows uninterrupted cortical growth, suggesting that *Lystrosaurus* from the Jiucaiyuan Formation had a high intrinsic rate of growth that could periodically arrest.

Our results are consistent with a recent report of *Lystrosaurus* bone histology from the same formation with respect to the preponderance of highly vascularized, rapidly deposited cortical bone [40]. However, contrary to Han et al. [40], we do not find evidence of peripherally slowed growth rates in individuals that are as large (~60% MKS) and larger than the maximum size sampled in their study. Additionally, we pose an alternative interpretation to the assignment of LAGs in the laminar to plexiform tissue reported in ([40]:fig 2G-H, fig 3C, fig 5, fig 6D, G-H) as bright lines that appear in the context of periosteal bone formation ([90]:fig3C; [91]:fig3). Finally, we report fewer instances of growth marks, particularly in the smallest size class where no evidence of episodic growth marks is seen (contra [40]). When present, we can confidently trace 1 to 3 growth marks (as either LAGs or annuli) around the circumference of individuals that are approximately 70% maximum known size.

Size Class II

The smallest association of elements includes cranial and postcranial material (hind limb and forelimb elements) that was collected in situ. A femur, tibia, fibula, ulna, and radius were selected for consumptive sampling (composite images available on Morphobank under project number P4023). The femur measures 122.38 mm in length. When compared to femora from larger, presumably more mature individuals, this femur (IVPP V 27126a) is approximately 50% maximum known size. The bone tissue in each of the five associated limb elements is extremely well-vascularized with longitudinally-oriented primary osteons arranged in circumferential layers

with some radial anastomoses (Fig 3). Woven fibered bone matrix and large, densely distributed osteocyte lacunae surround the primary vascular canals (Fig 3B, E). Vascular canal size and density remain consistently high from the endosteal to the periosteal margin indicating that this individual grew rapidly until death (Fig 3D-E). There is no evidence of episodic growth cycles (as either LAGs or annuli) in this individual.

Figure 3. Representative bone histology of Size Class II from the mid-shafts of *Lystrosaurus* sp. hind limb elements. **A**, fibula (IVPP V 27124-1) consisting of well-vascularized tissue with longitudinally-oriented primary osteons in circumferential layers. **B**, 10X magnification of yellow square in **A**, arrows indicate bright lines in the extracellular matrix of periosteally accreted bone and should not be confused as growth marks. **C**, 20X magnification of **B**. **D**, fibula (IVPP V 27126c) cross section consisting of longitudinally and radially oriented primary osteons and dense osteocyte lacunae. **E**, 20X magnification of fibula (IVPP V 27126c) shows woven-fibered bone (yellow arrows) in the peripheral cortex indicating rapid bone deposition at death. **F**, cross section of a proximal tibia (IVPP V 27124-3) with a slight decrease in vascular canal sizes towards the sub-periosteal edge, magnified in **G**. wb= woven-fibered bone; po= primary osteon. Scale bars = 1 mm in **A** & **F**, 500 µm in **D** & **G**, 100 µm in **B**, **C** & **E**.

A right proximal fibula (IVPP V 27124-1), right proximal tibia (IVPP V 27124-3), distal tibia (IVPP V 27124-4), distal femur (IVPP V 27124-6) and complete femur (IVPP V 27124-7) were collected individually but are analyzed together here as immature individuals because limb dimensions and histological features are indicative of sustained, active bone growth at death. The complete femur is approximately 50% maximum known size, similar to IVPP V 27126a. In thin

section, each of these disassociated elements has a large medullary cavity infilled with a loose trabecular network. Cortical tissue consists of woven-fibered bone with longitudinally oriented primary osteons loosely arranged in circumferential rows. In the proximal tibia, vascular canal size decreases substantially in a regionalized band deep to the sub-periosteal edge but it rebounds to the typical highly vascularized tissue more peripherally, likely representing a temporary shift in growth rate (Fig 3F-G). In the fibula, there are regional differences in vascular canal size and organization that likely represent functional differences in bone apposition rates [92]. For example, longitudinal vascular canals are organized into stacked, circumferential layers on the medial side of the proximal fibula (IVPP V 27124-1) (Fig 3B-C) whereas the lateral side is less organized bone (Fig 3A). The amount of densely packed osteocyte lacunae in woven-fibered bone is higher in the less organized areas, but many large, globular lacunae are still apparent between primary osteons and in thin sheets in the more organized regions (Fig 3B-C). In more organized areas of the highly vascularized tissue, the mineralized matrix surrounding the vascular canals should not be confused with lines of arrested growth (Fig 3B-C). At low magnifications, delineated bands of extracellular matrix separate the primary vascular space in periosteally accreted bone [91]. However, at high magnifications, these accretion lines, or bright lines (sensu [91]), do not form continuous rings around the cortex and should not be confused as growth marks or as slower forming tissues like parallel-fibered or lamellar bone [45]. Bright lines lack osteocyte lacunae and represent the saltatory activity of the periosteum when new bone is accreted [45, 91]. In specimens in Size Class II, highly vascularized longitudinal canals in woven- to parallel-fibered matrix continue to the sub-periosteal edge, indicating that individuals died while actively growing.

Size Class III

A femur (IVPP V 27125a), tibia (IVPP V 27125b), and fibula (IVPP V 27125c) are associated as one individual that is approximately 70% maximum size based on femoral length. Unlike smaller sampled elements, the cortex is interrupted by two growth marks (Fig 4). In all associated elements, a deep cortical LAG is sandwiched between large vascular canals towards the endosteal margin and smaller vascular canals more peripherally (Fig 4A-B, lowermost arrows in F, K). In the fibula, the LAG is superficial to a region of immature secondary osteons that sit in the deep cortex (Fig 4J). The second of the two growth marks is more variable, in part due to the fragmentary nature of the subperiosteal edge of the femur. In the femur, a poorly vascularized annulus of parallel-fibered to lamellar tissue sits along the sub-periosteal edge and is more apparent in thin areas of the cortex (Fig 4D). By contrast, thicker regions of the outer cortex show a mixture of longitudinal and circular primary osteons in circumferential layers that approaches a laminar organization that continues to the incomplete sub-periosteal edge (Fig 4C). In the tibia and fibula, the outer cortical growth mark is made up of two LAGs in the tibia, and an annulus of avascular parallel-fibered tissue in the fibula (Fig 4E, J-K). Interestingly, the mineral preservation of the tibia and fibula are such that the mineralized bone matrix is clearly visible between each circumferential layer of vascular canals (Fig 4F, K, L). These thin, mineralized lines of extracellular matrix should not be confused for growth marks and instead are the mineralizing front of periosteally accreted bone [45, 90]. The vascular orientation shifts in the hind limb elements of this individual from reticular to laminar in the femur (Fig 4B), and from disorganized longitudinally oriented primary osteons to circumferentially organized longitudinal primary osteons in the tibia and fibula (Fig 4F, I). In addition, primary osteon size decreases towards the outermost periosteal margin but vascular canals remain open to the sub-

periosteal edge (Fig 4F-G). The only exception to this pattern is in the thinnest region of the cortices, particularly in the fibula, where the sub-periosteal edge is avascular and consists of well organized, parallel-fibered bone (Fig 4D, I, K). However, this organized tissue is discontinuous around the cross section, suggesting that bone growth slowed at differential rates in regions of the outer cortex, but did not fully arrest.

Figure 4. Bone histology of associated hind limb elements of *Lystrosaurus* sp. representative of Size Class III. **A**, femur (IVPP V 27125a) with a deep cortical LAG in **B** (blue box in **A**). The outer cortex has regions of high vascularity seen in **C** (yellow box in **A**), and regions marked by a nearly continuous peripheral growth mark of parallel-fibered tissue (denoted by yellow brackets) in **D** (orange box in **A**). **E**, tibia (IVPP V 27125b) has two cortical growth marks seen in **F** (yellow box in **E**; yellow arrows in **F**). The outer growth mark can variably be seen as a double LAG in **G** (green box in **E**; yellow arrows in **G**). **H**, the fibula (IVPP V 27125c) has a thinner cortex interrupted by ~~a~~ two LAGs seen in **I** (yellow box in **H**; yellow arrows in **I**). **J**, magnification of yellow box in **I** shows the cross-cutting inner cortical LAG (arrow in **J**). **K**, thinner regions of the cortex appear avascular (purple box in **H**), with stacked mineralized lamellae of parallel-fibered bone (yellow bracket), whereas thicker regions (blue box in **H**) show peripheral pulses of vascularized growth, within the lamellated tissue, seen in **L** (yellow bracket). Scale bars = 5 mm in **A**, 1 mm in **E**, 2 mm in **H**, 500 μm in **B**, **C**, **F** & **I**, and 100 μm in **D**, **G**, **J** - **L**.

A slightly larger specimen (IVPP V 26548) is an association of hind limb and forelimb elements as well as at least one rib that was previously histologically sampled by Han et al.

2021[40]. The femur (IVPP V 26548a), tibia (IVPP V 26548b), and fibula (IVPP V 26548c) show plexiform tissue interrupted by two growth marks, with some variation in the outer growth mark, especially in the fibula where it splits into two LAGs (Fig 5). In the femur and tibia, the deeper growth mark is distinguishable as a dramatic reduction in vascular canal size, immediately followed by a return to the typical, large vascular canals in the middle of the cortex (Fig 5A, B, D). Throughout the middle cortex of the femur, the deeper growth mark changes in thickness. In thinner regions of the cortex, the growth mark appears as a cross-cutting LAG separating smaller vascular canals from larger canals more superficially. In the thicker regions of the cortex, the growth mark appears as an annulus, or region of little to no vasculature that is not accompanied by a hyper-mineralized line (Fig 5D). In both the femur and tibia, vascular canals reduce in size again towards the sub-periosteal margin, making up the second growth mark of an annulus of avascular parallel-fibered to lamellar tissue (Fig 5C). The fibula shows a mid-cortical annulus that is made up of avascular parallel-fibered to lamellar tissue (Fig 5G). Similar to the femur and tibia, there is a sub-periosteal reduction in vascular canal size with at least two LAGs that can be traced around the entire cortex of the fibula, indicating variation in the type of growth mark deposited in hind limb elements of the same individual (Fig 5E, G, H). Small, simple vascular canals sit within the context of this peripheral lamellar tissue, which is atypical for an external fundamental system (EFS) indicative of somatic maturity, making it more likely that this lamellar tissue represents a temporary cessation in growth, similar to the annulus recorded in the deeper cortex.

Figure 5. Bone histology of associated elements of *Lystrosaurus* sp. representative of Size Class III. **A**, femur (IVPP V 26548a) has two growth marks. **B**, a deep, mid-cortical annulus (yellow

box in **A**; inner yellow bracket in **B**), avascular lamellar tissue makes up the second growth mark (outer yellow bracket in **B**). **C**, magnification of peripheral lamellar tissue, left panel reflects blue box in **A**, right panel reflects green box in **A**. **D**, first (osteologically deeper) annulus (yellow bracket) in plane and cross-polarized light with a lambda filter. **E**, magnification of blue box in **A** of outer cortical growth mark that varies in thickness around the periphery of the thin section, here consisting of two LAGs. **F**, associated fibula (IVPP V 26548c) highlights a similar arrangement of deep and more superficial growth marks consisting of, at times, numerous layers of lamellar tissue in an annulus. **G**, magnification of yellow box in **F** showing two growth marks; annulus (yellow bracket) and peripheral LAGs (yellow arrows). **H**, magnification of green box in **F** showing variation in the outer growth mark such that in some regions it appears as a thicker region of lamellar tissue with one cross-cutting LAG. **I**, high magnification image of the associated tibia (IVPP V 26548b) showing the presence of woven-fibered bone between primary osteons within the middle cortex. **J**, humerus with mid-cortical annulus of reduced vascularity. **K**, magnification of yellow box in **J** of annulus (yellow bracket). **L**, high magnification of parallel-fibered bone making up the annulus in the humerus (orange box in J). **M**, ulna (IVPP V 27124-2) with a thinner overall cortex made up of parallel-fibered and small amounts of woven-fibered bone. **N**, magnification of yellow box in **M** highlighting the thin cortex in plane and cross-polarized light with a lambda filter. **O**, high magnification of outer cortex of the ulna showing small areas of woven-fibered bone (blue box in **M**). wb= woven-fibered bone; pfb= parallel-fibered bone. Scale bars = 1 mm in **A**, **B**, **F**, **J**, & **M**, 500 µm in **E**, **G** & **K**, and 100 µm in **C**, **D**, **H**, **I**, **L**, **N**, & **O**.

A complete humerus (IVPP V 27124-8) of comparable size to IVPP V 26548 [40], has an exceedingly thick cortex with one mid-cortical growth mark (Fig 5J). The medullary cavity is nearly infilled by a dense network of trabecular bone. The cortex increases in thickness in the anterodorsal region (up in Fig 5J) and consists of highly vascularized laminar bone in woven- and parallel-fibered bone matrix. Vascular canals have a circular orientation and show a marked decrease in size coincident with a mid-cortical LAG (Fig 5K). The LAG is traceable around the entire cross-section and is followed by a pulse of highly vascularized tissue with large canal spaces (Fig 5K). Vascular canal size reduces slightly towards the sub-periosteal margin which could indicate the beginning of another growth mark, similar to the mid-cortical growth mark.

A distal fragment of an ulna (IVPP V 27124-2; original complete length estimated to be 93mm) has a thin cortex with a large medullary cavity infilled with a loose network of trabecular bone (Fig 5M). The transition from trabeculae to compact cortical bone includes a region of enlarged resorption cavities and immature secondary osteons. The middle and outer cortex is composed of small longitudinal canals that sit in a parallel-fibered matrix where small regions of woven-fibered bone persist (Fig 5N, O). Along the sub-periosteal margin, vascular canals are reduced but occasionally open to the bone edge, suggesting that this individual died while still growing (Fig 5M). The proportion of parallel-fibered bone in this element is higher than in hind limb elements, however, there is no evidence to suggest somatic maturity or attainment of maximum size.

Size Class IV

Unlike the middle size class, the largest sampled femur (IVPP V 27127) does not have LAGs or annuli (Fig 6A). Despite cracks in its cortex, [illegible]he bone tissue architecture is easily

discernible and consists of laminar canals in woven- and parallel-fibered matrix based on the lacunar morphologies at high magnification (Fig 6B, C). Woven-fibered bone makes up more of the extracellular matrix in the deep and middle cortex whereas the outer cortex consists of parallel-fibered bone with small areas of woven-fibered bone (Fig 6C). There is no indication of transition to slowed bone deposition in the small regions of sub-periosteal bone that remain intact, suggesting a sustained rate of growth at death (Fig 6B).

Figure 6. Bone histology of the largest femur (IVPP V 27127) of *Lystrosaurus* sp. that represents Size Class IV and rib fragments of unknown size. **A**, the femur is cracked (yellow arrow is a large circular crack) but preserves regions of the entire cortex (yellow box). **B**, the same view of the yellow box in **A** using cross-polarized light with a lambda filter shows well-vascularized, plexiform tissue along the sub-periosteal margin (yellow box in **A**). **C**, left panel shows woven-fibered bone in the deep-middle cortex (blue box in **A**) and outer cortex, right panel (green box in **A**). **D**, rib fragments (IVPP V 27124-5) have infilled medullary cavities, secondary osteons within the deep cortex and a largely avascular compact outer cortex consisting of organized lamellar tissue. **E**, magnification of yellow box in **D** showing the peripheral ~~lamellar~~ tissue in the yellow bracket. **F**, magnification of the yellow box in **E** under plane and cross-polarized light with a lambda filter. **G**, magnification of blue box in **D** similarly shows that peripheral ~~lamellar~~ tissue is present (yellow bracket). wb= woven-fibered bone. Scale bars = 10 mm in **A**, 1 mm in **D**, 500 μm in **B** & **G**, 100 μm in **C**, **E** & **F**.

Unknown Size Class

Four partial ribs (IVPP V 27124-5) belonging to an individual of unknown size class are preserved in a block of sediment (Fig 6D). In thin section, they have large, infilled medullary cavities that grade into thin, compact cortices. Scarce vascular canals and immature secondary osteons sit in the deeper cortex whereas the outer cortex is avascular and consists of parallel-fibered to lamellar tissue with well-organized osteocyte lacunae (Fig 6E–F). Due to the fragmentary nature of this specimen and the fact that it is not associated with limb bones, it is unclear whether the peripheral ~~lamellar~~ tissue is indicative of ~~a~~ EFS or a temporary cessation in growth. Additionally, ribs grow at different rates than limbs and do not preserve the same record of life history information compared to a midshaft hind limb thin-section.

Discussion

All *Lystrosaurus* individuals sampled from the Jiucaiyuan Formation show abundant, highly vascularized primary bone tissue throughout the majority of the cortices of the hind limb, humerus, and radius. The ulna and ribs showed fewer vascular canals, overall thinner cortices, and more organized primary bone tissue. Three size classes were identified and histologically described as: II) a highly vascularized cortex of circumferential layers of radial and longitudinal vascular canals in ~50% max known size individuals: III) highly vascularized plexiform tissue interrupted by one to two growth marks that range from one or two closely spaced LAGs or annuli in ~70% maximum known size individuals; and IV) uninterrupted laminar tissue in a 100% maximum known size individual. These results force us to reconsider what an adult-sized specimen might look like for *Lystrosaurus* sp., as the current sample lacks histologic indicators of somatic~~ally~~ maturity (e.g., an external fundamental system) [78]. Botha [22] found similar results for South African species of *Lystrosaurus*, suggesting that species typified by small body

size (viz. *L. murrayi*) were very likely capable of reaching much larger sizes, but never lived long enough to do so.

Han et al. [40] suggested that sub-adult *Lystrosaurus* from northern Pangea may have reached some level of somatic maturity inferred from parallel-fibered bone and peripheral LAGs in a dorsal rib. However, histological data from the femur, tibia, and fibula of the same individual indicate episodic cessations in growth instead of an overall pattern of slowed growth. The presence of slower growing bone tissue in the ribs likely reflects differential growth rates seen throughout the skeleton, rather than somatic maturity [93, 49].

Growth Marks

Unlike the bone histology reported from South African and Indian *Lystrosaurus*, in which growth marks are rare and often limited to a single LAG or annulus, we find one, and more commonly, two growth marks in immature *Lystrosaurus* from the Jiucaiyuan Formation. The number of growth marks is mostly consistent across associated elements from the same individual as well as across individuals of the same size class (Table 1). Interestingly, the type of growth mark, as either a LAG, a series of closely spaced and occasionally bifurcating LAGs, an annulus consisting of lamellar tissue, or an annulus consisting of parallel-fibered to lamellar tissue, varied throughout the current sample. In most cases, the first (i.e., osteologically deeper) growth mark, or singular growth mark in the case of the humerus, was often recorded as an annulus accompanied by a reduction of vascular canal sizes. The outer cortical growth marks were more variable, and often varied within the same thin section.

We did not find evidence of growth marks in individuals in Size Class II. These individuals had characteristic juvenile bone histology (i.e., large radial to longitudinal vascular

canals that frequently anastomose); therefore our analysis disagrees with the assignment of LAGs in juvenile *Lystrosaurus* by Han et al. [40]:fig 3. In some cases, the mineralization front can be mis-identified as a growth mark in the context of periosteally deposited bone. Currey [91] and others (e.g., [90]), describe laminar bone formation where acretted circumferential layers of bone (made up of a woven scaffold that is later infilled with parallel-fibered or lamellar bone in the form of primary osteons) is bordered by the periosteum. As the periosteum jumps peripherally when new bone is accreted, a bright line is left between each circumferential layer and represents the place at which the periosteum was located before new bone was added. This mineralized line differs from a growth mark as it is not continuous around the cortex and is not hyper-mineralized but appears as a bright line often devoid of osteocyte lacunae [90]. Growth marks, on the other hand, are continuous around the cortex, unless secondary remodeling or cortical remodeling causes discontinuities (i.e., by erosion).

All growth marks observed in our analysis come from Size Class III, which corresponds to individuals that are approximately 70% maximum known size. We can confidently trace up to two growth marks in the current Chinese sample, which is higher than reports from South African and Indian specimens of *Lystrosaurus*, which generally lack growth marks. However, the remainder of the cortical bone tissue in the current Chinese sample reflects the similarly high and consistent rate of bone growth reported from South African and Indian *Lystrosaurus* [22] [54]. A slight increase in the number of growth marks in northern Pangean *Lystrosaurus* could be explained by one or two possible factors that are not mutually exclusive: (i) by an increase in their lifespan or (ii) by environmental stressors.

There is a longstanding hypothesis that the Permo-Triassic extinction caused a Lilliput effect, a persistent reduction in body size, in surviving terrestrial lineages [94-97, 19]. This claim

has been investigated histologically in therocephalians, which support an overall reduction in body size following the extinction [97, 19]. A Lilliput effect was also posited for *Lystrosaurus*, based in large part on the overwhelming abundance of small skulls in the Karoo Basin and museum collections in South Africa [98, 99, 20, 21]. However, Botha [22] later rejected a strict Lilliput effect for southern Pangean *Lystrosaurus* (based on fossils from South Africa and India) because of the lack of mature histological tissue in all of the species examined [22]. Instead, she [22] suggested that the large proportion of small skulls in the Triassic was better explained by high mortality in immature individuals, as assessed by their long bone histology.

Body size

To better understand how *Lystrosaurus* from the Jiucaiyuan Formation compare to congeners from more southerly parts of Pangea, we gathered body size data (based on basal skull length and femur length) from the literature [54, 22, 40] and personal observations of Early Triassic-aged specimens collected in China, South Africa, and India. From this sample, it is clear that Chinese *Lystrosaurus* have a larger average body size (Fig 7, Fig 8). However, cranial size distributions suggest that both populations from South Africa and China had the potential to grow to similar maximum recorded sizes (Fig 7). The lack of large specimens of *Lystrosaurus* in South Africa aligns with previous interpretations for increased mortality at small sizes there by Botha [22]; also see [20]. Our data provide evidence suggesting that northern Pangean *Lystrosaurus* could have reached larger sizes more readily or easily. However, it is unclear the degree to which differences in sampling practices might affect the body size distributions seen here. A decade of detailed stratigraphic work targeting the Permo-Triassic boundary in South Africa has led to more comprehensive fossil sampling, which should provide a relatively

unbiased view of the vertebrate fauna [100, 101]. However, historical collections from the Karoo likely systematically emphasized rare components of the assemblage (e.g., [102]). Likewise, the Permo-Triassic vertebrate record from Xinjiang has not been subject to the same intensive collecting as that of the Karoo Basin, and therefore it currently may not provide an unbiased view of body size distributions or relative abundances of taxa.

Figure 7. Interspecific body size distributions from Early Triassic *Lystrosaurus* show an abundance of large individuals from China. Species from South Africa and China overlap in maximum basal skull length but individuals from China have larger average size. See S2 Dataset for more information.

Figure 8. Interspecific femoral lengths from Early Triassic *Lystrosaurus* show a higher frequency of large sizes from China. Size Classes denote femora that are less than 40% maximum known size (MKS) in I, between 40-65% MKS in II, approximately 70-75% MKS in III, and greater than 79% in IV. See S2 Dataset for more information.

The presence of large individuals with episodic growth marks in all of the subadult-sized elements suggests that *Lystrosaurus* from the Jiucaiyuan Formation were able to reach large sizes through prolonged and sustained rapid growth, unlike Triassic *Lystrosaurus* from South Africa that rarely lived long enough to reach large body sizes despite growing rapidly (Fig 7). There are several possible explanations for this pattern. First, physiological differences among the species of *Lystrosaurus* could be responsible. This proposal is difficult to test, as the alpha-taxonomy of Chinese *Lystrosaurus* is in need of revision and phylogenetic studies of the genus

have typically only included *L. hedini* as a sole northern hemisphere representative (e.g., [103]). However, to the degree that patterns preserved in hard tissue histology can shed light on physiology, the current data suggests that *L. curvatus*, *L. declivis*, *L. maccaigi*, and *L. murrayi* are effectively indistinguishable [22, 31], which suggests a degree of consistency within the genus.

Climatic differences in the high-southern (60°S) versus mid-northern (45°N) paleolatitudes [104, 105] is another possible explanation for the observed difference in body size and inferred lifespan in *Lystrosaurus*. Paleoclimate reconstructions of the latest Permian to earliest Triassic show extreme increases in temperature and aridity [106-109], mega monsoons [110, 111], chemical weathering, soil erosion, and geochemical and isotopic signatures of mass wasting and greenhouse conditions [112, 8,7/30/2021 10:05:00 PM113]. Facies that span the terrestrial Permo-Triassic boundary of the Karoo Basin are interpreted as a change from meandering rivers to low-sinuosity braid-plains, resulting from increased aridity and a die-off of rooted plants that supported river morphologies [114, 115]. However, more recent evidence from isotopic and geochemical data produced differing estimates of the degree to which aridity increased in the Karoo Basin, complicating our understanding of seasonal variation in precipitation and resource availability [111, 116, 117]. Nevertheless, conditions were disruptive enough to have destabilized terrestrial communities and caused a prolonged interval of faunal turnover in southern Pangea during the extinction interval [118, 15, 18].

The extreme environmental and climatic conditions of southern Pangea are not reflected in the paleoclimate reconstructions of the Juicaiyuan Formation [74]. Here, relatively stable subhumid to semiarid conditions are interpreted from cyclostratigraphic and paleosol analyses in the South Taodonggou study area [75, 74]. Furthermore, fluvial and lacustrine strata host a

diversity of plant, root, and wood fossils that are occasionally preserved in situ and show signatures of wildfires during the Early Triassic [74, 119-121]. In sum, evidence from the paleobotanical record of the Jiucaiyuan Formation suggests a lush and vegetated environment that hosted a diverse terrestrial fauna in earliest Triassic times [64, 40]. Taken together, there is mounting evidence from sedimentologic, isotopic, and cyclostratigraphic data that points to seasonal, semi-arid conditions in the Karoo Basin and stable, sub-humid conditions in the Turpan-Hami Basin.

The extreme environmental and climatic conditions inferred from the Karoo Basin likely truncated *Lystrosaurus* lifespans in the Early Triassic. Similar environmental constraints on maximum size and longevity are also known from Early Triassic therocephalians and terrestrial temnospondyls, which also lack growth marks [19, 52]. By contrast, we propose that the more favorable environmental conditions and vegetated landscape interpreted from the Jiucaiyuan Formation could have hosted longer-lived *Lystrosaurus* during the Early Triassic. Relatively stable environmental conditions could have allowed populations of *Lystrosaurus* to reach larger average body sizes than their southern Pangean relatives. The prevalence of multiple growth marks in some, but not all, immature individuals, further supports developmentally plastic growth in *Lystrosaurus* first proposed for southern Pangean populations [22]. It also suggests that some individuals experienced periodic arrests of growth, perhaps associated with environmental disruptions, but that these disturbances may not have caused widespread mortality on the scale suggested by the South African record. Similarly, some individuals apparently experienced sufficiently stable conditions for them to grow to comparatively large size without interruption. Alternatively, the uninterrupted growth record of the largest specimen sampled here could also be due to the higher stratigraphic position of this individual; it is possible that upper

Juicaiyuan rocks represent more favorable environmental conditions. Finally, the absence of peripheral parallel-fibered bone or consistent, circumferential lamellar tissue in the current sample indicates that the maximum size of *Lystrosaurus* from the Jiucaiyuan Formation remains unknown.

Conclusions

We present bone histology, cranial geometric morphometric analyses, and body size data for *Lystrosaurus* from the Jiucaiyuan Formation of northwestern China and report statistically different cranial morphologies, an extended lifespan, and larger average body size compared to *Lystrosaurus* living in southern Pangea during Early Triassic times. In addition, we report up to two growth marks in immature individuals, which is inconsistent with previous reports of *Lystrosaurus* life histories from the Turpan-Hami Basin [40]. We suggest that it is unlikely that the growth marks reported here represent regular, cyclical patterns and more likely represent instances where bone growth stopped due to unfavorable environmental conditions. Indeed, the absence of growth marks in the largest sampled individual indicates an intrinsically high rate of growth that persisted throughout ontogeny. In addition, the presence of two cessations in growth in individuals from Size Class III indicates that smaller, likely more immature, individuals may have been more susceptible to fluctuations in environmental conditions. However, appositional bone growth rebounded to a rapid rate following these cessations, indicating a flexible physiology, as previously proposed for higher-latitude populations of *Lystrosaurus* [22, 31]. A better sample of large limb bones (>250 mm) as well as an analysis of tusk dentine deposition, are needed to clarify what impact the Early Triassic environment may have had on *Lystrosaurus* growth and development in northern Pangea.

Supporting Information

S1 Supporting Information. Figures and corresponding tables for cranial geometric morphometric analysis and bone wall thickness measurements. (PDF)

S2 Dataset. Basal skull length measurements (BSL) and femoral length measurements for Early Triassic *Lystrosaurus*. (Excel Spreadsheet)

Acknowledgements. — At the IVPP, we thank YI Jian and SHUKANG Zhang for assistance with thin section preparation as well as LIU Jun for access to field data and specimen collections. We also thank Alida Bailleul for helpful discussion and microscope access. Collecting and compiling the entirety of the comparative datasets would not have been possible if it weren't for the following museum curators, collection managers, colleagues, and friends who we deeply thank: Zaituna Skosan, Claire Browning, Christian Kammerer, Roger Smith, Jennifer Botha, Elize Butler, Bernhard Zipfel, Bruce Rubidge, Sifelani Jirah, Viktor Radermacher, Carl Mehling, and Mark Norell. For their helpful discussion on earlier versions of this manuscript, we thank Bryan Gee, Savannah Olroyd, and Elliott Armour Smith. Helpful comments that greatly improved the quality of this work were given by Jennifer Botha and one anonymous reviewer. Finally, we are indebted to Wan Yang for collecting some of the fossils analyzed here, organizing fieldwork in 2019 and leading the multidisciplinary project that instigated this research.

References

1. Raup DM, Sepkoski JJ. Periodicity of extinctions in the geologic past. Proc Natl Acad Sci. 1984 Feb 1;81(3):801–5.

2. Erwin DH. The Permo–Triassic extinction. Nature. 1994 Jan;367(6460):231–6.

3. Chen Z-Q, Benton MJ. The timing and pattern of biotic recovery following the end-Permian mass extinction. Nat Geosci. 2012 Jun;5(6):375–83.

4. Benton M. Diversification and extinction in the history of life. Science. 1995 Apr 7;268(5207):52–8.

5. Benton MJ, Twitchett RJ. How to kill (almost) all life: the end-Permian extinction event. Trends Ecol Evol. 2003 Jul;18(7):358–65.

6. Sahney S, Benton MJ. Recovery from the most profound mass extinction of all time. Proc R Soc B Biol Sci. 2008 Apr 7;275(1636):759–65.

7. Sobolev SV, Sobolev AV, Kuzmin DV, Krivolutskaya NA, Petrunin AG, Arndt NT, et al. Linking mantle plumes, large igneous provinces and environmental catastrophes. Nature. 2011 Sep;477(7364):312–6.

8. Benton MJ, Newell AJ. Impacts of global warming on Permo-Triassic terrestrial ecosystems. Gondwana Res. 2014 May;25(4):1308–37.

9. Retallack GJ, Krull ES. Carbon isotopic evidence for terminal-Permian methane outbursts and their role in extinctions of animals, plants, coral reefs, and peat swamps. In: Greb SF, DiMichele WA, editors. Wetlands Through Time: Geological Society of America Special Paper. 2006. p. 249–68.

10. Nowak H, Schneebeli-Hermann E, Kustatscher E. No mass extinction for land plants at the Permian–Triassic transition. Nat Commun. 2019 Dec;10(1):384.

11. Mishra S, Jha N, Stebbins A, Brookfield M, Hannigan R. Palaeoenvironments, flora, and organic carbon and nitrogen isotope changes across the non-marine Permian-Triassic boundary at Wybung Head, Australia. Palaeogeogr Palaeoclimatol Palaeoecol. 2019 Nov;534:109292.

12. Botha J, Smith RMH. Rapid vertebrate recuperation in the Karoo Basin of South Africa following the End-Permian extinction. J Afr Earth Sci. 2006 Aug 1;45(4):502–14.

13. Botha J, Abdala F, Smith R. The oldest cynodont: new clues on the origin and early diversification of the Cynodontia. Zool J Linn Soc. 2007;149(3):477–92.

14. Fröbisch J. Vertebrate diversity across the end-Permian mass extinction — Separating biological and geological signals. Palaeogeogr Palaeoclimatol Palaeoecol. 2013 Feb;372:50–61.

15. Roopnarine PD, Angielczyk KD, Weik A, Dineen A. Ecological persistence, incumbency and reorganization in the Karoo Basin during the Permian-Triassic transition. Earth-Sci Rev. 2019 Feb;189:244–63.

16. Bernardi M, Petti FM, Benton MJ. Tetrapod distribution and temperature rise during the Permian–Triassic mass extinction. Proc R Soc B Biol Sci. 2018 Jan 10;285(1870):20172331.

17. Allen BJ, Wignall PB, Hill DJ, Saupe EE, Dunhill AM. The latitudinal diversity gradient of tetrapods across the Permo-Triassic mass extinction and recovery interval. Proc R Soc B Biol Sci. 2020 Jun 24;287(1929):20201125.

18. Viglietti PA, Benson RBJ, Smith RMH, Botha J, Kammerer CF, Skosan Z, et al. Evidence from South Africa for a protracted end-Permian extinction on land. Proc Natl Acad Sci. 2021 Apr 27;118(17):e2017045118.

19. Huttenlocker AK, Botha-Brink J. Bone microstructure and the evolution of growth patterns in Permo-Triassic therocephalians (Amniota, Therapsida) of South Africa. PeerJ. 2014 Apr 8;2:e325.

20. Botha-Brink J, Codron D, Huttenlocker AK, Angielczyk KD, Ruta M. Breeding Young as a Survival Strategy during Earth's Greatest Mass Extinction. Sci Rep [Internet]. 2016 Jul;6(1). Available from: http://www.nature.com/articles/srep24053

21. Codron J, Botha-Brink J, Codron D, Huttenlocker AK, Angielczyk KD. Predator-prey interactions amongst Permo-Triassic terrestrial vertebrates as a deterministic factor influencing faunal collapse and turnover. J Evol Biol. 2017 Jan;30(1):40–54.

22. Botha J. The paleobiology and paleoecology of South African *Lystrosaurus*. PeerJ. 2020 Nov 24;8:e10408.

23. Nicolas M, Rubidge BS. Changes in Permo-Triassic terrestrial tetrapod ecological representation in the Beaufort Group (Karoo Supergroup) of South Africa. Lethaia. 2010 Mar;43(1):45–59.

24. Smith R, Rubidge BS, van der Walt M. Therapsid Biodiversity Patterns and Paleoenvironments of the Karoo Basin, South Africa (pp. 31-63) Roger Smith, Bruce Rubidge and Merrill van der Walt. In: The Forerunners of Mammals. Indiana University Press; 2012.

25. King GM, Cluver MA. The Aquatic *Lystrosaurus*: An Alternative Lifestyle. Hist Biol. 1991;4:323–41.

26. Jasinoski SC, Rayfield EJ, Chinsamy A. Comparative Feeding Biomechanics of *Lystrosaurus* and the Generalized Dicynodont Oudenodon. Anat Rec Adv Integr Anat Evol Biol. 2009 Jun 1;292(6):862–74.

27. Retallack GJ, Smith RMH, Ward PD. Vertebrate extinction across Permian–Triassic boundary in Karoo Basin, South Africa. Geol Soc Am Bull. 2003;115(9):1133.

28. Botha-Brink J. Burrowing in *Lystrosaurus*: preadaptation to a postextinction environment? J Vertebr Paleontol. 2017 Sep 3;37(5):e1365080.

29. Liu J, Angielczyk KD, Abdala F. Permo-Triassic tetrapods and their climate implications. Glob Planet Change. Forthcoming.

30. Botha-Brink J, Angielczyk KD. Do extraordinarily high growth rates in Permo-Triassic dicynodonts (Therapsida, Anomodontia) explain their success before and after the end-Permian extinction? Zool J Linn Soc. 2010 Oct 1;160(2):341–65.

31. Whitney MR, Sidor CA. Evidence of torpor in the tusks of *Lystrosaurus* from the Early Triassic of Antarctica. Commun Biol [Internet]. 2020;3(1). Available from: http://www.nature.com/articles/s42003-020-01207-6

32. Tripathi C, Puri SN. On the remains of *Lystrosaurus* from the Panchets of the Raniganj coalfield. Rec Geol Surv India. 1961;89:407–19.

33. Colbert EH. *Lystrosaurus* from Antarctica. Am Mus Novit. 1974;(2535):44.

34. Gubin Y, Sinitza S. Triassic terrestrial tetrapods of Mongolia and the geological structure of the Sain-Sar-Bulak locality. In: The Nonmarine Triassic. 1993. p. 169–70.

35. Liu J, Li J, Cheng Z. The *Lystrosaurus* fossils from Xinjiang and their bearing on the terrestrial permian triassic boundary. Vertebr Pal Asiat. 2002;40(4):267–75.

36. Sun A. Late Permian and Triassic Terrestrial Tetrapods of North China. Vertebr Palasiat. 1980;XVIII(2):11.

37. Ray S. *Lystrosaurus* (Therapsida, Dicynodontia) from India: Taxonomy, relative growth and Cranial dimorphism. J Syst Palaeontol. 2005 Jan;3(2):203–21.

38. Surkov MV, Kalandadze NN, Benton MJ. *Lystrosaurus georgi* , a dicynodont from the Lower Triassic of Russia. J Vertebr Paleontol. 2005 Jun 27;25(2):402–13.

39. Araújo R, Fernandez V, Polcyn MJ, Fröbisch J, Martins RMS. Aspects of gorgonopsian paleobiology and evolution: insights from the basicranium, occiput, osseous labyrinth, vasculature, and neuroanatomy. PeerJ. 2017 Apr 11;5:e3119.

40. Han F, Zhao Q, Liu J. Preliminary bone histological analysis of *Lystrosaurus* (Therapsida: Dicynodontia) from the Lower Triassic of North China, and its implication for lifestyle and environments after the end-Permian extinction. Fröbisch J, editor. PLOS ONE. 2021 Mar 18;16(3):e0248681.

41. Padian K, Lamm E-T, editors. Bone histology of fossil tetrapods: advancing methods, analysis, and interpretation. Berkeley: University of California Press; 2013. 285 p.

42. Köhler M, Marín-Moratalla N, Jordana X, Aanes R. Seasonal bone growth and physiology in endotherms shed light on dinosaur physiology. Nature. 2012 Jul;487(7407):358–61.

43. Castanet J, Croci S, Aujard F, Perret M, Cubo J, Margerie E de. Lines of arrested growth in bone and age estimation in a small primate: Microcebus murinus. J Zool. 2004 May 1;263(1):31–9.

44. Buffrénil V de, Quilhac A. Bone Tissue Types: A Brief Account of Currently Used Categories. In: Vertebrate Skeletal Histology and Paleohistology. S.l.: CRC PRESS; 2021. p. 147–82.

45. Francillon-Vieillot H, de Buffrénil V, Castanet J, Géraudie J, Meunier FJ, Sire JY, et al. Microstructure and Mineralization of Vertebrate Skeletal Tissues. In: Skeletal Biomineralization: Patterns, Processes and Evolutionary Trends [Internet]. American Geophysical Union; 1990. p. 175–234. Available from: http://dx.doi.org/10.1029/SC005p0175

46. Enlow H, Brown SO. A Comparative Histological Study of Fossil and Recent Bone Tissues. Part II. Tex J Sci. 1957;9(2):30.

47. Enlow H, Brown SO. A comparative histological study of fossil and recent bone tissues, Part III. Tex J Sci. 1958;10(2):187–230.

48. Castanet J, Newman DG, Girons HS. Skeletochronological Data on the Growth, Age, and Population Structure of the Tuatara, Sphenodon punctatus, on Stephens and Lady Alice Islands, New Zealand. 1988;14.

49. Castanet J. Time recording in bone microstructures of endothermic animals; functional relationships. Comptes Rendus Palevol. 2006 Mar;5(3–4):629–36.

50. Nacarino-Meneses C, Köhler M. Limb bone histology records birth in mammals. PLOS ONE. 2018 Jun 20;13(6):e0198511.

51. Sinsch U, Oromi N, Sanuy D. Growth marks in natterjack toad (Bufo calamita) bones: histological correlates of hibernation and aestivation periods. 2007;9.

52. Canoville A, Chinsamy A. Bone Microstructure of the Stereospondyl *Lydekkerina Huxleyi* Reveals Adaptive Strategies to the Harsh Post Permian-Extinction Environment: BONE MICROSTRUCTURE of LYDEKKERINA HUXLEYI. Anat Rec. 2015 Jul;298(7):1237–54.

53. Mchugh JB. Paleohistology of *Micropholis stowi* (Dissorophoidea) and *Lydekkerina huxleyi* (Lydekkerinidae) humeri from the Karoo Basin of South Africa, and implications for bone microstructure evolution in temnospondyl amphibians. J Vertebr Paleontol. 2015 Jan 2;35(1):e902845.

54. Ray S, Chinsamy A, Bandyopadhyay S. *Lystrosaurus murrayi* (therapsida, Dicynodontia): Bone Histology, Growth and Lifestyle Adaptations. Palaeontology. 2005 Nov 1;48(6):1169–85.

55. Yuan PL, Young CC. On the Occurrence of *Lystrosaurus* in Sinkiang. Bull Geol Soc China. 1934;13(1):575–80.

56. Young CC. On two skeletons of Dicynodontia. Bull Geol Soc China. 1935;(14):483–517.

57. Young CC. Additional Dicynodontia remains from Sinkiang. Bull Geol Soc China. 1939;(19):111–36.

58. Sun A-L. Preliminary report on a new species of *Lystrosaurus* of Sinkiang. Vertebr Pal Asiat. 1964;8:216–7.

59. Sun A-L. Permo-Triassic dicynodonts from Turfan, Sinkiang. Permo-Triassic Vertebr Foss Turfan Basin. 1973;53–68.

60. Cheng Z. Vertebrates. In: Permian and Triassic Strata and Fossil Assemblages in the Dalongkou Area of Jimsar, Xinjiang. 1986. p. 207–18. (People's Republic of China Ministry of Geology and Mineral Resources Geological Memoirs).

61. Cluver MA. The cranial morphology of the dicynodont genus *Lystrosaurus* [Doctoral dissertation]. [Stellenbosch]: Stellenbosch University; 1970.

62. Cosgriff JW, Hammer WR, Ryan WJ. The Pangaean Reptile, *Lystrosaurus maccaigi*, in the Lower Triassic of Antarctica. J Paleontol. 1982;56(2):371–85.

63. King G. Anomodontia. Encycl Paleoherpetology. 1988;1–74.

64. Lucas SG. Chinese Fossil Vertebrates. Columbia University Press; 2001. 390 p.

65. Li J, Sun A. Subclass Synapsida. In: The Chinese Fossil Reptiles and Their Kin. Sci. Press; 2008. p. 379–409.

66. Fröbisch J. Composition and similarity of global anomodont-bearing tetrapod faunas. Earth-Sci Rev. 2009 Aug;95(3–4):119–57.

67. Camp J, Liu J. The taxonomy and cranial morphology of Chinese *Lystrosaurus*. J Vertebr Paleontol. 2011;31(3).

68. Camp J. Morphological variation and disparity in *Lystrosaurus*. University of Iowa; 2010.

69. Rohlf FJ. tpsDig. Stony Brook, NY: Department of Ecology and Evolution, State University of New York at Stony Brook; 2010.

70. Kammerer CF, Deutsch M, Lungmus JK, Angielczyk KD. Effects of taphonomic deformation on geometric morphometric analysis of fossils: a study using the dicynodont *Diictodon feliceps* (Therapsida, Anomodontia). PeerJ. 2020 Oct 7;8:e9925.

71. Adams D, Collyer M, Kaliontzopoulou A, Sherratt E. Geomorph: Software for geometric morphometric analyses. 2016.

72. Adams DC, Collyer ML. Multivariate Phylogenetic Comparative Methods: Evaluations, Comparisons, and Recommendations. Syst Biol. 2018 Jan 1;67(1):14–31.

73. Schlager S. Morpho and Rvcg – Shape Analysis in R: R-Packages for Geometric Morphometrics, Shape Analysis and Surface Manipulations. In: Zheng G, Li S, Székely G, editors. Statistical Shape and Deformation Analysis [Internet]. Academic Press; 2017. p. 217–56. Available from: https://www.sciencedirect.com/science/article/pii/B9780128104934000110

74. Yang W, Wan M, Crowley JL, Wang J, Luo X, Tabor NJ, et al. Paleoenvironmental and paleoclimatic and cyclo- and chrono-stratigraphy of upper Permian–Lower Triassic fluvio-lacustrine deposits in Bogda Mountains, NW China – implications for diachronous plant evolution across Permian-Triassic boundary. Earth-Sci Rev. Forthcoming.

75. Yang W, Feng Q, Liu Y, Tabor N, Miggins D, Crowley JL, et al. Depositional environments and cyclo- and chronostratigraphy of uppermost Carboniferous–Lower Triassic fluvial–lacustrine deposits, southern Bogda Mountains, NW China — A terrestrial paleoclimatic record of mid-latitude NE Pangea. Glob Planet Change. 2010 Aug;73(1–2):15–113.

76. Lamm E-T. 4. Preparation and Sectioning of Specimens [Internet]. Bone Histology of Fossil Tetrapods. University of California Press; 2013. Available from: https://www.degruyter.com/document/doi/10.1525/9780520955110-006/html

77. Holliday CM, Ridgely RC, Sedlmayr JC, Witmer LM. Cartilaginous Epiphyses in Extant Archosaurs and Their Implications for Reconstructing Limb Function in Dinosaurs. Farke AA, editor. PLoS ONE. 2010 Sep 30;5(9):e13120.

78. Griffin CT, Stefanic CM, Lessner EJ, Riegler M, Formoso K, Koeller K, et al. Assessing ontogenetic maturity in extinct saurian reptiles. Biol Rev. 2020;56.

79. de Ricqlès A. Recherches paleohistologiques sur les os longs des tetrapodes; II, Quelques observations sur la structure des os longs des theriodontes. Ann Pale´ontologie. 1969;55(1):1–51.

80. Ray S, Botha J, Chinsamy A. Bone histology and growth patterns of some nonmammalian therapsids. J Vertebr Paleontol. 2004 Sep 10;24(3):634–48.

81. Ray S, Bandyopadhyay S, Bhawal D. Growth patterns as deduced from bone microstructure of some selected neotherapsids with special emphasis on dicynodonts: Phylogenetic implications. Palaeoworld. 2009 Mar;18(1):53–66.

82. Ray S, Botha-Brink J. Dicynodont Growth Dynamics and Lifestyle Adaptations. In: Forerunners of Mammals. Indiana University Press; 2012. p. 29.

83. Olivier C, Houssaye A, Jalil N-E, Cubo J. First palaeohistological inference of resting metabolic rate in an extinct synapsid, *Moghreberia nmachouensis* (Therapsida: Anomodontia). Biol J Linn Soc. 2017 Jun 1;121(2):409–19.

84. Marotti G. Static and dynamic osteogenesis. 2010;4.

85. Stein K, Prondvai E. Rethinking the nature of fibrolamellar bone: an integrative biological revision of sauropod plexiform bone formation. Biol Rev. 2014;89(1):24–47.

86. Ferretti M, Palumbo C, Contri M, Marotti G. Static and dynamic osteogenesis: two different types of bone formation. Anat Embryol (Berl). 2002 Dec 1;206(1–2):21–9.

87. Shapiro F, Wu J. Woven bone overview: structural classification based on its integral role in developmental, repair and pathological bone formation throughout vertebrate groups. Eur Cell Mater. 2019 Oct 1;38:137–67.

88. de Ricqlès A. Evolution of Endothermy; Histological Evidence. Evol Theory. 1974;1(2):51–80.

89. Prondvai E, Stein KHW, de Ricqlès A, Cubo J. Development-based revision of bone tissue classification: the importance of semantics for science. Biol J Linn Soc. 2014 Aug 1;112(4):799–816.

90. Mori R, Kodaka T, Sano T, Yamagishi N, Asari M, Naito Y. Comparative Histology of the Laminar Bone between Young Calves and Foals. Cells Tissues Organs. 2003;175(1):43–50.

91. Currey JD. Differences in the Blood-Supply of Bone of Different Histological Types. J Cell Sci. 1960 Sep 1;s3-101(55):351–70.

92. Currey JD. The many adaptations of bone. J Biomech. 2003 Oct;36(10):1487–95.

93. de Margerie E, Cubo J, Castanet J. Bone typology and growth rate: testing and quantifying ‘Amprino’s rule’ in the mallard (*Anas platyrhynchos*). C R Biol. 2002 Mar 1;325(3):221–30.

94. Urbanek A. Biotic crises in the history of Upper Silurian graptoloids: A Palaeobiological model. Hist Biol. 1993 Jul;7(1):29–50.

95. Harries PJ, Knorr PO. What does the ‘Lilliput Effect’ mean? Palaeogeogr Palaeoclimatol Palaeoecol. 2009 Dec;284(1–2):4–10.

96. Huttenlocker AK. Body Size Reductions in Nonmammalian Eutheriodont Therapsids (Synapsida) during the End-Permian Mass Extinction. PLOS ONE. 2014 Feb 3;9(2):e87553.

97. Huttenlocker AK, Botha-Brink J. Body size and growth patterns in the therocephalian *Moschorhinus kitchingi* (Therapsida: Eutheriodontia) before and after the end-Permian extinction in South Africa. Paleobiology. 2013;39(2):253–77.

98. Grine FE, Forster CA, Cluver MA, Georgi JA. Cranial Variability, Ontogeny, and Taxonomy of *Lystrosaurus* from the Karoo Basin of South Africa. In: Amniote Paleobiology. University of Chicago Press; 2006. p. 432–506.

99. Botha J, Smith RMH. *Lystrosaurus* species composition across the Permo–Triassic boundary in the Karoo Basin of South Africa. Lethaia. 2007;40(2):125–37.

100. Smith RMH, Botha-Brink J. Anatomy of a mass extinction: Sedimentological and taphonomic evidence for drought-induced die-offs at the Permo-Triassic boundary in the main Karoo Basin, South Africa. Palaeogeogr Palaeoclimatol Palaeoecol. 2014 Feb;396:99–118.

101. Botha J, Huttenlocker AK, Smith RMH, Prevec R, Viglietti P, Modesto SP. New geochemical and palaeontological data from the Permian-Triassic boundary in the South African Karoo Basin test the synchronicity of terrestrial and marine extinctions. Palaeogeogr Palaeoclimatol Palaeoecol. 2020 Feb;540:109467.

102. Kitching JW. The distribution of the Karroo vertebrate fauna: with special reference to certain genera and the bearing of this distribution on the zoning of the Beaufort Beds. University of the Witwatersrand; 1977. 1–131 p.

103. Kammerer CF, Viglietti PA, Hancox PJ, Butler RJ, Choiniere JN. A new kannemeyeriiform dicynodont (*Ufudocyclops mukanelai*, gen. et sp. nov.) from Subzone C of the *Cynognathus* Assemblage Zone, Triassic of South Africa, with implications for biostratigraphic correlation with other African Triassic faunas. J Vertebr Paleontol. 2019;39(2):e1596921.

104. Scotese CR, Wright N. PALEOMAP paleodigital elevation models (PaleoDEMS) for the Phanerozoic [Internet]. 2018. Available from: https://www.earthbyte.org/paleodem-resource- scotese-and-wright-2018/

105. Scotese CR. An Atlas of Phanerozoic Paleogeographic Maps: The Seas Come In and the Seas Go Out. Annu Rev Earth Planet Sci. 2021 May 30;49(1):annurev-earth-081320-064052.

106. Kidder DL, Worsley TR. Causes and consequences of extreme Permo-Triassic warming to globally equable climate and relation to the Permo-Triassic extinction and recovery. Palaeogeogr Palaeoclimatol Palaeoecol. 2004 Feb;203(3–4):207–37.

107. Kiehl JT, Shields CA. Climate simulation of the latest Permian: Implications for mass extinction. Geology. 2005;33(9):757.

108. Winguth AME, Shields CA, Winguth C. Transition into a Hothouse World at the Permian–Triassic boundary—A model study. Palaeogeogr Palaeoclimatol Palaeoecol. 2015 Dec 15;440:316–27.

109. Rey K, Amiot R, Fourel F, Abdala F, Fluteau F, Jalil N-E, et al. Oxygen isotopes suggest elevated thermometabolism within multiple Permo-Triassic therapsid clades. Elife. 2017;6:e28589.

110. Winguth A, Winguth C. Precession-driven monsoon variability at the Permian–Triassic boundary — Implications for anoxia and the mass extinction. Glob Planet Change. 2013 Jun 1;105:160–70.

111. Tabor NJ, Sidor CA, Smith RMH, Nesbitt SJ, Angielczyk KD. Paleosols of the Permian-Triassic: proxies for rainfall, climate change and major changes in terrestrial tetrapod diversity. J Vertebr Paleontol. 2017 Nov 29;37(sup1):240–53.

112. Sun Y, Joachimski MM, Wignall PB, Yan C, Chen Y, Jiang H, et al. Lethally Hot Temperatures During the Early Triassic Greenhouse. Science. 2012 Oct 19;338(6105):366–70.

113. Frank TD, Fielding CR, Winguth AME, Savatic K, Tevyaw A, Winguth C, et al. Pace, magnitude, and nature of terrestrial climate change through the end-Permian extinction in southeastern Gondwana. Geology [Internet]. 2021 May 19; Available from: https://doi.org/10.1130/G48795.1

114. Smith RMH. Changing fluvial environments across the Permian-Triassic boundary in the Karoo Basin, South Africa and possible causes of tetrapod extinctions. Palaeogeogr Palaeoclimatol Palaeoecol. 1995 Aug;117(1–2):81–104.

115. Ward PD. Altered River Morphology in South Africa Related to the Permian-Triassic Extinction. Science. 2000 Sep 8;289(5485):1740–3.

116. Viglietti PA, Smith RMH, Rubidge BS. Changing palaeoenvironments and tetrapod populations in the *Daptocephalus* Assemblage Zone (Karoo Basin, South Africa) indicate early onset of the Permo-Triassic mass extinction. J Afr Earth Sci. 2018 Feb;138:102–11.

117. Gastaldo RA, Kamo SL, Neveling J, Geissman JW, Looy CV, Martini AM. The base of the *Lystrosaurus* Assemblage Zone, Karoo Basin, predates the end-Permian marine extinction. Nat Commun [Internet]. 2020;11(1). Available from: http://www.nature.com/articles/s41467-020-15243-7

118. Roopnarine PD, Angielczyk KD. Community stability and selective extinction during the Permian-Triassic mass extinction. Science. 2015 Oct 2;350(6256):90–3.

119. Metcalfe I, Foster CB, Afonin SA, Nicoll RS, Mundil R, Xiaofeng W, et al. Stratigraphy, biostratigraphy and C-isotopes of the Permian–Triassic non-marine sequence at Dalongkou and Lucaogou, Xinjiang Province, China. J Asian Earth Sci. 2009 Nov;36(6):503–20.

120. Wan M, Yang W, Wang J. *Amyelon bogdense* sp. nov., a silicified gymnospermous root from the Changhsingian–Induan (?) in southern Bogda Mountains, northwestern China. Rev Palaeobot Palynol. 2019 Apr;263:12–27.

121. Wan M-L, Yang W, Wan S, Wang J. Wildfires in the Early Triassic of northeastern Pangaea: Evidence from fossil charcoal in the Bogda Mountains, northwestern China. Palaeoworld. 2021 Jul;S1871174X21000639.

Living Fast in the Triassic: New data on life history in *Lystrosaurus* (Therapsida: Dicynodontia) from northeastern Pangea

Short Title: Life history in *Lystrosaurus* from northeastern Pangea

Zoe T. Kulik[1*], Jacqueline K. Lungmus[2], Kenneth D. Angielczyk[3], and Christian A. Sidor[1]

~~Keywords: *Lystrosaurus*, bone histology, life history, Triassic, China, Jiucaiyuan Formation~~

~~Zoe T. Kulik* [zkulik@uw.edu]~~,1 Department of Biology and Burke Museum, University of Washington, Seattle, WA, United States of America ~~98195~~

~~Jacqueline Lungmus [lungmusj@si.edu]~~2 Smithsonian National Museum of Natural History Washington, D.C., United States of America ~~20560~~

[3]~~Kenneth D. Angielczyk [kangielczyk@fieldmuseum.org],~~ Negaunee Integrative Research Center, Field Museum of Natural History, Chicago, IL, United States of America ~~60605~~

* Corresponding author

E-mail: zkulik@uw.edu

Formatted: Superscript
Formatted: Superscript
Formatted: Superscript
Formatted: Superscript
Formatted: Line spacing: Double
Formatted: Line spacing: Double
Formatted: Superscript
Formatted: Superscript
Formatted: Superscript
Formatted: Superscript
Formatted: Line spacing: Double
Formatted: Superscript
Formatted: Superscript
Formatted: Superscript
Formatted: Line spacing: Double
Formatted: Superscript
Formatted: Line spacing: Double

Christian A. Sidor [casidor@uw.edu], Department of Biology and Burke Museum, University of Washington, Seattle, WA 98195

Formatted: Line spacing: Double

Abstract

Lystrosaurus was one of the few tetrapods to survive the Permo-Traissic mass extinction, the most profound biotic crisis in Earth's history. The wide paleolatitudinal range and incredible high abundance of *Lystrosaurus* during the EEarly Triassic provides a unique opportunity to investigate changes in growth dynamics and longevity following the mass extinction, yet most studies have focused only on species that lived in the southern hemisphere. Here, we present the long bone histology from twenty *Lystrosaurus* skeletal elements spanning a range of sizes that were collected in the Jiucaiyuan Formation of northwestern China. In addition, we compare the average body size of northern and southern Pangean Triassic-aged species and conduct cranial geometric morphometric analyses between of southern and northern taxa to begin investigating whether specimens from China are likely to be taxonomically distinct from South African specimens. We demonstrate that *Lystrosaurus* from China have larger average body sizes than their southern Pangean relatives and that cranial morphologies are distinctive. The osteohistological ostehostological examination revealed sustained, rapid osteogenesis punctuated by growth marks, in some, but not all, immature individuals from China. We find that the osteohistology of Chinese *Lystrosaurus* shares a similar growth pattern to with South African species that show sustained growth until death,. howeverHowever, bone growth arrests more

frequently in the Chinese sample. NeverthelessNonetheless, each none of the long bones sampled here do not indicate that maximum or asymptotic size was reached, suggesting that the maximum size of *Lystrosaurus* from the Jiucaiyuan Formation remains unknown.

Introduction

The Permo-Triassic mass extinction was the most devastating biotic crisis in Earth's history and caused communities to collapse in both the terrestrial and marine realms (1-3]. Fluctuating climates accompanied by an overall increase in global temperatures are hypothesized to have forced 70% of terrestrial vertebrate families to extinction [4-8], although plant extinctions were notably damped [9-11]. Recovery after the Permo-Triassic extinction has been a topic of intense research, but most studies in the terrestrial realm focus on taxonomic diversity and abundance [12-14], or changes in community structure and dynamics following the extinction [6, 3, 15-18]. Few studies have examined how the extremely changed environment of the earliest Triassic impacted growth rate, life history, and longevity of surviving taxa [19-22]. Importantly, most of the studies noted above rely heavily—if not exclusively—on data from the southern hemisphere, in particular the Karoo Basin of South Africa.

Lystrosaurus, a stoutly built dicynodont therapsid that ranged in maximum skull size from approximately 16–39 cm (*L. murrayi* and *L. maccaigi* from South Africa, respectively), is one of the hallmark survivors of the Permo-Triassic extinction due to its remarkable abundance in southern Pangean deposits (23-24, 18]. The success of *Lystrosaurus* has been variously attributed to its generalist diet of tough plant material (e.g., [25, 26]), broad habitat tolerances [27], a burrowing lifestyle [28], unusual thermal tolerances [29], and a developmentally plastic growth strategy that allowed it ~~the ability~~ to weather extreme ecosystem instability [30, 20, 22, 31]. These hypothesized advantages/exaptations for a post-extinction environment have been studied almost exclusively using specimens from South Africa, but *Lystrosaurus* is known from across Pangea: including Permo-Triassic aged deposits in Antarctica, Mongolia, Russia, China, India, and possibly Mozambique (e.g., [32-39]). The wide paleolatitudinal range of *Lystrosaurus* offers a rare opportunity to investigate differences in lifespan, survivorship, and body size in southern and northern Pangean populations that experienced markedly different environmental conditions in the wake of the Permo-Triassic extinction (e.g., [22, 40]).

Formatted: Line spacing: Double

Osteohistological Perspectives on Life History

Bone histology provides a wealth of information about an extinct animal's growth and life history. Mineralized bone matrix, vascular canal spaces, and cellular spaces persist in fossilized bone, making it possible to determine the relative rate of bone deposition as well as maturity of extinct organisms [41]. Cyclical slowdowns in skeletal growth rate leave histological markers, termed growth marks, in primary cortical bone. Annuli and lines of arrested growth (LAGs) are two types of cyclical growth marks that are deposited in annual or seasonal cycles [42-44]. Annuli represent periodic decreases in growth rate and are usually identified based on

specific, contextual changes in the bone tissue architecture, such as a dramatic decrease of vascular and cellular density, or a temporary shift to parallel-fibered or lamellar bone.~~(43) One~~ LAGs, on the other hand,~~particularly useful yet controversial undefined histological feature in determining aspects of an extinct animal's growth are hyper-mineralized lines of arrested growth (LAGs), which~~ represent periodic cessations in growth and appear as ~~cement~~ hyperminerlized lines in the primary cortical bone of most vertebrates when bone deposition ~~periodically~~ stops in unfavorable season~~al or annual cycles~~ s [44-49, 42]. In addition to extrinsically (i.e., environmentally or seasonally) induced ~~LAGs~~growth marks, growth can also arrest during periods of intense stress, such as birth, and during modulations to metabolic activity such as torpor [50, 51]. The periodicity of ~~LAGs~~ growth marks (i.e., the extent to which they are regularly spaced throughout the cortex of large, presumably mature individuals) helps to determine whether they represent extrinsic or intrinsic events.

Botha [22] recently conducted a detailed examination of growth and life history patterns in four species of *Lystrosaurus* across the Permian-Triassic boundary using fossils from South Africa. Using a combination of bone histology and body size data, Botha [22] found that Triassic *Lystrosaurus* individuals grew rapidly and had increased mortality at small sizes, as evidenced by the overwhelming abundance of small skulls known from Triassic rocks in the Karoo Basin. This post-extinction change in life expectancy is also inferred from the scarcity of ~~lines of arrested growth (LAGs)~~LAGs and annuli in the rapidly deposited cortical bone of Triassic specimens. In comparison, Permian individuals had larger average skull sizes and had numerous ~~LAGs~~ growth marks spaced throughout the cortex, suggesting prolonged periods of growth spanning multiple seasons or years [22]. Similar patterns were previously found across a wider

Formatted: Font color: Text 1

taxonomic range of South African therapsid species ~~by Botha-Brink et al.~~ [20] and lydekkerinid stereospondyls [52, 53]. The predominance of small *Lystrosaurus* (and other tetrapods) with rapidly deposited bone tissue suggests an overall shift in life history strategy across the Permian-Triassic ~~extinction~~ boundary to an early onset of reproductive maturity [20, 52, 53].

Histological data for *Lystrosaurus* outside of the South African Karoo Basin ~~of South Africa~~ are more limited, but critical to understanding the relationship between latitude (and by extension climate) and life history. Bones of *Lystrosaurus* from the Early Triassic of India lack LAGs and annuli, and have small skulls, consistent with the pattern seen in South Africa [37, 54]. A preliminary report of the bone histology from *Lystrosaurus* from the Turpan-Hami Basin of China suggested that subadult individuals record multiple LAGs [40]. However, in the absence of comparative body size data, it remains unclear if multiple LAGs in the Chinese specimens indicate prolonged periods of growth (i.e., multiple cycles or seasons of growth) or environmental instability (e.g., disruptions to the metabolic rate as a result of resource limitations). Here, we analyze bone histology from a size range of *Lystrosaurus* postcrania from the Jiucaiyuan Formation of ~~northwestern~~ Xinjiang, China (Fig 1) as well as body size data across Triassic-aged *Lystrosaurus* assemblages to investigate whether *Lystrosaurus* from northern Pangea had differing life histories than its southern Pangean relatives.

Formatted: Line spacing: Double

Figure 1. Size distribution of *Lystrosaurus* sp. postcrania ~~and ribs~~ from the Jiucaiyuan formation included in the histologic analysis. Twenty skeletal elements, with gray bars indicating associations of individuals, span three size classes inferred from femoral length: Size Class II

spans 47–63% maximum femoral size, Size Class III includes specimens that are ~ 70% maximum size, and Size Class III includes the largest partial femur. Scale bar = 5 cm.

Materials and Methods

Geometric Morphometric Analysis of Skull Shape

An important question to consider when comparing the bone histology of *Lystrosaurus* specimens from China and other regions is whether the same species occur in the different geographic areas. Although the ~~initial~~ first material of *Lystrosaurus* collected in Xinjiang was referred to the South African species *L. murrayi* [55], a total of seven endemic *Lystrosaurus* species also have been described [56-60]. Colbert [33]; also see [61-63], considered some Chinese specimens to represent *L. murrayi* and *L. curvatus*, and others to represent endemic species. More recent authors have been split over whether *Lystrosaurus* specimens from Xinjiang represent exclusively endemic species or a mixture of endemic and more cosmopolitan species, as well as the number of valid endemic species (e.g., [35, 64-67, 40]).

A comprehensive taxonomic revision of *Lystrosaurus* from Xinjiang is beyond the scope of this paper. However, to gain insight into whether Chinese *Lystrosaurus* specimens are likely to be taxonomically distinct from South African specimens, we conducted a preliminary two-dimensional geometric morphometric analysis of skull shape. The skulls were analyzed in three perspectives - anterior, dorsal, and lateral – following the methodology of [68]; also see [67]. The anterior shape analysis included 45 individual specimens, 15 from China and the remaining 30 from South Africa. The dorsal shape analysis included 107 specimens, 14 from China and 93 from South Africa. Lastly, the lateral analysis included 95 specimens, 9 from China and 86 from South Africa. Landmarks were collected using the tpsDig2ws software [69]. In the case of the

anterior and dorsal views, landmarks were digitized on only the left half of the skull. The lateral view digitization included a combination of Type I and Type II landmarks (12 total) with a single curve of six semilandmarks along the outer edge of the snout between landmarks number 1 and 11 (Fig 2). The anterior and dorsal views did not contain any semilandmarks, and have 10 and 13 landmarks, respectively. Because taphonomic deformation can complicate the interpretation of shape variation at low taxonomic levels in dicynodonts [70], data collection focused on specimens that appeared to show relatively little evidence of distortion. See the supplementary files for additional details of the morphometric analysis.

The coordinate data were processed and analyzed further with the Geomorph R package [71], where they underwent a generalized Procrustes superimposition. Principal components analyses were used to visualize major patterns of shape variation, and a Procrustes ANOVA was run on each dataset to assess the statistical relationship between shape and country of origin using the method of [72] and the Geomorph package in R. A canonical variates analysis was run using the CVA function of the Morpho package [73] in R to further explore the association between shape and geographic location, as well as the accuracy with which specimens could be classified using skull shape (see *Results*).

Figure 2. Results of the morphometric analysis comparing specimens of *Lystrosaurus* from China and South Africa. Principal components plots and histogram of canonical variates analysis results for the three perspectives (Anterior – A; Dorsal – B; Lateral – C). Skull drawings on the right correspond to the orientation for each analysis. Orange dots on the specimens show placement of landmarks used in the 2D geometric morphometric analysis (see S1 Supporting Information for more details). Blue dots represent specimens from China and red dots represent

specimens from South Africa. Arrows point to the warp grids showing corresponding deformation along each principal component axis. Histograms show frequency of a recovered canonical variate value and show the distinct differences between Chinese and South African specimens, with overlap only occurring in a few specimens in the lateral orientation.

Formatted: Line spacing: Double

Histological Sample

Twenty skeletal elements (humerus, femur, tibia, radius, ulna, fibula, and ribs; Table 1; Fig 1) of *Lystrosaurus* sp. Were selected for consumptive analysis from the collections of the Institute of Vertebrate Paleontology and Paleoanthropology (IVPP). The majority of the ~~All~~ ~~m~~fossils~~aterial~~ were~~as~~ recovered from strata approximately 30–50 meters above the base of the Jiucaiyuan Formation ~~from~~ in the South Taodonggou area, Turpan-Hami Basin, Xinjiang Uygur Autonomous Region, China. One element (IVPP V 27127) came from a nearby locality in Zhaobishan and was recovered about 312m above the base of the Jiucaiyuan Formation [74]. ~~T~~The Jiucaiyuan Formation overlies the Guodikeng Formation lithologically (=upper Wutonggou low-order cycle) and the Wutonggou low-order cycle, and its lower contact is characterized by a facies change from lacustrine to fluvial deposits [75, 74]. This facies change is interpreted as the boundary between the Permian and Triassic [74] and recent work suggests that the Jiucaiyuan Formation is late Induan-early Olenekian in age [74]. As noted above, the lack of clarity regarding Chinese *Lystrosaurus* taxonomy means that we were unable to assign species-level identifications. However, the majority of our histologic samples ~~are associations~~come from ~~of~~ four individuals. Skeletal elements are differentiated by specimen numbers followed by a letter, in the case of associated individuals (e.g., IVPP V #-a, -b, etc.) The remaining eight specimens are disarticulated elements collected from the same locality and are assigned to one

Formatted: Font: Italic

specimen number IVPP V_27124, differentiated by IVPP V_27124-1, -2, etc. In addition to the histologic sample, 50 femora were measured from Early Triassic specimens compiled from the literature [37, 22] and from personal observations to contextualize the range of sizes in this monodominant vertebrate assemblage (S2 Dataset). Additional body size proxies, including basal skull length (S2 Dataset), were measured from *Lystrosaurus* specimens housed in collections at the National Museum (NMQR), the Evolutionary Studies Institute (ESI), the Iziko South African Museum (SAM), the Field Museum of Natural History (FMNH), the American Museum of Natural history (AMNH), and the Burke Museum of Natural History and Culture (UWBM). Cranial and postcranial measurements were made using Neiko digital calipers.

TABLE 1. Postcranial element length and maximum midshaft diameter for the twenty skeletal elements of *Lystrosaurus* sp. from the Jiucaiyuan Formation that were histologically sampled. Asterisk indicates fragmentary or incomplete specimens with accompanying estimates based on complete specimens in this dataset.

	Spec. No.	Preserved Length (mm)	Estimated Length (mm)	<u>Max</u> Midshaft Diameter (mm)	Growth Marks	Size Class
**Femur**						
	IVPP V_27124-7	122.38		21~~19~~.~~2~~43	0	II
	IVPP V_27124-6	62.74*	129	16.22	0	II
	IVPP V_27126a	62*	162		0	II

Formatted: Line spacing: Double
Formatted Table
Formatted: Line spacing: Double
Formatted: Line spacing: Double
Formatted: Line spacing: Double
Formatted: Line spacing: Double

	IVPP V 27125a	182		28~~13.4~~	~~1~~2	III	**Formatted:** Line spacing: Double
	IVPP V 26548a	192		14.09	2	III	**Formatted:** Line spacing: Double
	IVPP V 27127	240*	255	33~~16~~.5~~7~~	0	IV	**Formatted:** Line spacing: Double
**Tibia**							**Formatted:** Line spacing: Double
	IVPP V 27126b	92			0	II	**Formatted:** Line spacing: Double
	IVPP V 27124-4	56.62*	94	15.86	0	II	**Formatted:** Line spacing: Double
	IVPP V 27124-3	53.26*	98	15.71	0	II	**Formatted:** Line spacing: Double
	IVPP V 26548b	148		10.47	2	III	**Formatted:** Line spacing: Double
	IVPP V 27125b	148*	212	10.36	2	III	**Formatted:** Line spacing: Double
**Fibula**							**Formatted:** Line spacing: Double
	IVPP V 27124-1	46.37*	86	~~10.12~~9.76	0	II	**Formatted:** Line spacing: Double
	IVPP V 27126c	86		5.8~~.~~9	0	II	**Formatted:** Line spacing: Double
	IVPP V 26548c	140		~~7.334~~9.02	2	III	**Formatted:** Line spacing: Double
	IVPP V 27125c	140		15.84	2-3	III	**Formatted:** Line spacing: Double
**Humerus**							**Formatted:** Line spacing: Double
	IVPP V 27124-8	151.77		23.96	1	III	**Formatted:** Line spacing: Double
**Radius**							**Formatted:** Line spacing: Double

	IVPP V_27126d	62			0	II
**Ulna**						
	IVPP V_27126e	75			0	II
	IVPP V_27124-2	47.32*	93	17.52	0	III
**Ribs**						
	IVPP V_27124-5	58.63*	N/A	48.76 - 56.33	EFS?	?

Thin Section Preparation

Thin sections were made following standard osteohistological techniques [76]. Eight of the specimens were sectioned at the University of Washington (UW); the remaining twelve were sectioned at the Institute of Vertebrate Paleontology and Paleoanthropology (IVPP). At the UW, mid-diaphyses were embedded in Epothin Epoxy/Resin 2, sectioned to a thickness of approximately 2 mm on a high-precision diamond-edged saw (Isomet 1000) and glued to glass slides using Devcon 2-Ton epoxy. Slides were ground on a Metaserv 3000 lapidary plate until the specimen was 80 µm thick or until optical clarity was reached. At the IVPP, midshafts were cut and embedded in polyester resin, sectioned into blocks using an Exact 300CP band saw, and glued to glass slides using Technovit 4000 resin. Slides were ground to optical clarity using an automated lapidary plate (Exakt 400CS). High magnification and composite images were taken using a Nikon Eclipse LV100POL microscope under plain and cross-polarized light with a lambda filter. Composite images were processed using Nikon NIS-Elements AR (version

5.20.02) imaging software. High-resolution images are available at Morphobank under project number P4023.

Ontogenetic maturity was assessed through bone tissue microstructure, articular surface texture and morphology, and size compared to other specimens of *Lystrosaurus* from the Jiucaiyuan Formation. The largest femur, estimated at 255 mm, is of comparable length to the largest known femur (*L. declivis* SAM-PK-K8038) from the Triassic of South Africa, which is 202 mm long. Therefore, we follow the size classes established by Botha [22] for southern Pangean *Lystrosaurus*, but note that our sample lacks Size Class I, or elements less than 40% maximum known size. The remainder of the elements were split into three size categories for analysis (Table 1; Fig 1). Size Class II includes the smallest and presumably most immature specimens in the current sample, ~~ranging in~~with femoral lengths that are approximately 120–160 mm long (47–63% of the maximum femur length observed), tibiae and fibulae that are less than 100 mm long, and associated forelimb elements~~, or 47–63% of the maximum femur length observed~~. All but the proximal tibia fragment (IVPP V 27124-3) have poorly formed joint surfaces and show pitting indicative of epiphyseal cartilage attachment ~~typical of~~in immature individuals [77]. By comparison, the joint surfaces of elements in the larger size classes are more robustly built and more completely ossified. The relative degree of epiphyseal ossification combined with histological features caused us to deviate slightly from Botha's [22] size classes by grouping specimens that are 40-65% maximum known size as Size Class II, rather than her 60% MKS cutoff. Our reasoning for this slight change is that the bone tissue is consistently and characteristically immature (i.e., highly vascularized plexiform to radial tissue without LAGs) in all of the associated elements that are 63% MKS. However, the incomplete tibia (IVPP V 27124-3, estimated length of 94 mm) that is estimated to be within Size Class II has a more developed

and ossified joint surface compared to similarly sized tibiae (IVPP V 27124-4, 94 mm in length) so it is likely that with more sampling, the split between Size Class II and III will be refined. Size Class III includes immature specimens that are approximately 70% maximum known size (again, based on femoral lengths from larger, complete, Jiucaiyuan specimens). Finally, Size Class IV includes a single femur with an estimated length of 255 mm (the specimen measures 240 mm and is missing the proximal shaft and head of the femur). To our knowledge, it is the largest femur of *Lystrosaurus* yet recovered from the Jiucaiyuan Formation, and is ~ 5 cm longer than the largest femur (discussed above) recovered from the Early Triassic of South Africa. Therefore, it presumably represents the most skeletally mature individual in the current sample. However, histological analysis will provide critical context for the relative maturity of this, and all other specimens sampled [78].

Bone Tissue Terminology

Bone tissue textures seen in *Lystrosaurus* and other therapsids vary widely from highly vascularized and disorganized woven-fibered bone to organized vascular canals in circumferential layers of parallel-fibered to lamellar bone [79-83, 30, 19]. The overall rate of bone growth affects the degree of organization of the extracellular matrix, with mineral fibers in a lamellar organization under slower growth, and disorganized woven-fibered textures under fast growth. Parallel-fibered bone is an intermediate tissue-type but it frequently occurs in the context of woven-fibered and lamellar bone. When large portions of the cortex consist of parallel-fibered bone, the mineralized extracellular matrix has an alternating anisotropic pattern that looks streaky under cross-polarized light. In many cases, the portion of woven- or parallel-fibered bone that sits between vascular canal spaces is so small that birefringent properties are difficult to

Formatted: Font: Italic

Formatted: Indent: First line: 0"

Formatted: Font: Italic

discern. When this is the case, it is possible to use the morphology and density of osteocyte lacunae (spaces where bone cells sit in living bone) to approximate the relative organization of mineralized fibers [84, 85]. Woven-fibered bone is statically-derived and includes large, globular, and often densely packed osteocyte lacunae within the mineralized matrix [86, 84]. In contrast, lamellar and parallel-fibered bone are dynamically-derived, and have lenticular or spindle-shaped osteocyte lacunae that are evenly distributed and parallel to the layers of the mineralized matrix [86, 84, 87]-. Importantly, in long bone cortices, the fiber network of parallel-fibered bone can either be aligned to the bone's long-axis (i.e., longitudinal fibers) or perpendicular to it (i.e., circular fibers) which can cause differences in lacunar morphology and fiber birefringence in polarized light depending on the plane of section [44]. When parallel fibers are perpendicular to the sectioned plane, they show mass isotropy or monorefringence, similar to woven-fibered bone [85]. Non-lamellar tissues (parallel- and woven-fibered bone) frequently incorporate large vascular spaces that later become infilled with concentric lamellae of parallel-fibered to lamellar bone as primary osteons. The resulting tissue is a fibrolamellar bone complex (sensu Francillon-Vieillot [45]) wherein a disorganized, nonlamellar matrix incorporates a network of centripetally infilled primary osteons. Ricqlès [88] coined much of the terminology used by paleohistologists and used a restricted definition of the fibrolamellar complex to apply only to bone tissue that strictly had woven-fibered matrix, rather than the combination of woven- and parallel-fibered bone used in other applications of the term. More recently, Stein and Prondvai [85] reinterpreted cortical bone structures using polarized-light microscopy of sauropod thin-sections cut from multiple planes. They concluded that the non-osteonal portion of cortical bone was largely parallel-fibered rather than woven and proposed new terminology for what they interpreted as highly organized primary bone (HOPB). They also proposed an additional term,

the woven-parallel complex, to combine the organized portions of HOPB with the more traditional terminology used in bone development and growth (for more, see [89]). The term woven-parallel complex describes a broad range of bone tissues with nonlamellar matrices and the term fibrolamellar complex is maintained as a subset of the woven-parallel complex where the cortical bone is densely vascularized with abundant woven-fibered bone forming the scaffold between vascular canals which is then surrounded by parallel-fibered or lamellar bone [89, 44]. This new terminology recognizes the developmental aspects of osteogenesis and the accumulation of osteoblasts within mineralizing osteoid. However, it may unnecessarily limit the role of woven-fibered bone to a transient tissue type typical of juvenile bone. Woven-fibered bone is known to be present in the typical development, maintenance, and repair of bone tissue throughout ontogeny in vertebrate groups and its abundance in cortical bone may depend on physiological processes rather than development alone [87]. In our histological descriptions below, we follow the recommendation [44, 89] to include lacunar morphology to estimate the proportion of woven- and parallel-fibered bone within the fibrolamellar and woven-parallel complexes and acknowledge the histovariability of parallel- and woven-fibered bone that can occur simultaneously and often in different proportions within the same section forming a spectrum of tissue types that can be difficult to define. ~~(44)~~

Results

Geometric Morphometric Analysis of Skull Shape

Anterior view: The first five principal components capture 81.41% of the total shape variance in anterior view (PC1 - 30.57%; PC2 - 22.03%; PC3 - 13.82%; PC4 - 8.71%; PC5 - 6.26%) (Fig 2A). Shape is a statistically significant predictor of geographic location for the

anterior view dataset (p = 0.001), and the canonical variates analysis correctly assigned specimen location 100% of the time.

PC1 primarily describes relative proportions of the snout. The subnarial region of the snout and caniniform process are dorsoventrally shorter relative to the height of the nasals and the anterior orbital margin in specimens with low PC1 scores, and there is greater lateral projection of the prefrontals relative to the caniniform process (Fig 2A). In contrast, the subnarial region and the caniniform process are proportionally much deeper relative to the anterior orbital margin and nasals in specimens with high PC1 scores, and the prefrontals and caniniform process are more closely aligned along a vertical axis. PC2 also captures aspects of skull widening and deepening, although in this case the differences are concentrated in the region of the nasals and prefrontals. Specifically, the prefrontal-nasal region is dorsoventrally shorter in specimens with low PC2 scores, and the dorsal and ventral extrema of the prefrontal are displaced laterally relative to the external naris and the lateral end of the nasofrontal suture. Specimens with high PC2 scores have dorsoventrally taller prefrontal-nasal regions, and the dorsal and ventral extrema of the prefrontal are more closely aligned with the external naris and the lateral edge of the nasofrontal suture. The Chinese and South African specimens are most clearly differentiated along PC2, with the Chinese specimens generally having lower PC2 scores.

Formatted: Line spacing: Double

Dorsal view: The first five principal components capture 79.75% of the shape variance in dorsal view (PC1 - 26.77%; PC2 - 23.57%; PC3 - 17.95%; PC4 - 6.18%; PC5 - 5.26%) (Fig 2B). Shape is a statistically significant predictor of geographic location for the dorsal view dataset (p = 0.001), and the canonical variates analysis correctly assigned specimen location 100% of the time.

Shape variation along PC1 is dominated by differences in the relative lengths of the interorbital region and the pineal foramen, and by the degree of lateral projection of the orbital rim. Specimens with low PC1 scores are characterized by an anteroposteriorly longer interorbital region and a shorter pineal foramen, and an orbital rim that does not project strongly laterally (Fig 2). The pineal foramen is relatively longer anteroposteriorly in specimens with high PC1 scores, and the anterior and posterior corners of the orbital rim (formed by the prefrontal and postorbital bar, respectively) project laterally away from the skull roof. PC2 also captures shape variation associated with relative proportions of the skull roof, as well as orbit shape. Specimens with low PC2 scores have an anteroposteriorly shorter pineal foramen and longer frontals, and the orbit is anteroposteriorly shorter, with anterior and posterior corners that are relatively aligned. The pineal foramen and orbit are relatively longer in specimens with high PC2 scores, and the posterior corner of the orbit (formed by the postorbital bar) projects farther laterally than the anterior orbital margin (formed by the prefrontal). As with the anterior view dataset, the Chinese and South African specimens are most clearly differentiated along PC2, with the Chinese specimens generally having lower PC2 scores.

Lateral view: The first five principal components capture 77.23% of the shape variance in lateral view (PC1 - 32.64%; PC2 - 18.57%; PC3 - 11.66%; PC4 - 8.58%; PC5 - 5.76%) (Fig 2C). Shape is a statistically significant predictor of geographic location ($p = 0.001$), and the canonical variates analysis had an overall classification accuracy of 95.78%, misclassifying two Chinese specimens in the dataset.

PC1 describes differences in the profile of the snout, position of the caniniform process relative to the orbit, and the anteroposterior dimensions of the orbit (Fig 2C). Specimens with

low PC1 scores have a lower, more anteriorly-convex snout profile with prefrontals that do not project much above the level of the skull roof, and the ventral edge of the caniniform process is located below the anterior orbital margin. The anteroposterior length of the orbit is relatively long. In contrast, specimens with high PC1 scores, have much taller, flatter snout profiles and more dorsally-projecting prefrontals, as well as anteroposteriorly shorter orbits and caniniform processes that are located anterior to the anterior orbital margin. PC2 also captures aspects of snout profile, including the degree of anterior projection of anteroventral corner of the premaxilla and the length of the caniniform process, as well as the dorsoventral height of the orbit. The tip of the premaxilla projects more anteriorly in specimens with low PC2 scores, the caniniform processes are relatively short, and the orbits are dorsoventrally shorter. Specimens with high PC2 scores have taller orbits, flatter snout profiles, and longer caniniform processes. There is little differentiation of the Chinese and South African specimens on the first three PC axes.

Taken together, our morphometric analyses suggest that there are consistent differences in skull shape between *Lystrosaurus* specimens from China and from South Africa, particularly in anterior and dorsal view. Based on these results, we tentatively suggest that the Chinese and South African populations were distinct at the species level. However, because our analyses do not directly address potential synonymies among the named Chinese species, and the fact that not all of the specimens we sampled histologically are associated with cranial material, we conservatively refer all of our histological samples to *Lystrosaurus* sp.

Formatted: Line spacing: Double

Osteohistology Overview

Lystrosaurus limb elements, regardless of size, have characteristically thick cortices. Cortical thickness (*K*) measures the proportional diameter of the medullary region relative to the

Formatted: Font: Italic

total cross-sectional diameter and remained nearly consistent at 0.63 and 0.64 in the smallest and largest femora in our dataset, respectively (see S1 Supporting Information). , with bone wall thicknesses ranging from 1.8 mm thick in the smallest femoral thin section, to 4 mm thick in the largest femoral thin section. Medullary cavities are typically infilled with trabecular bone that gradually transitions to a compact cortex. Similar to the bone histology of other dicynodonts and therapsids more broadly, the dominant bone tissue type in all sampled size classes of *Lystrosaurus* is well-vascularized cortical bone made up of varying proportions of woven- and parallel-fibered bone with laminar, plexiform, and longitudinally-oriented primary osteons, indicative of rapid osteogenesis woven[79, 80-83, 30, 19]. Longitudinal canals that frequently anastomose are arranged in circumferential layers in Size Class II whereas laminar vascular canal orientations are common in Size Class III. In Size Class III and IV, , fibrolamellar to laminar bone with a plexiform to longitudinal vascular orientation, indicative of rapid osteogenesis (78) (79) (80) (29) (81) (19) (82). Vvery little of the cortex is remodeled and when secondary osteons are present, they are restricted to the deepest portion of the cortex.

In the small individuals of *Lystrosaurus* studied here, rapidly deposited tissue is interrupted by either lines of arrested growth (LAGs or annuli. LAGs These growth marks are found in multiple regions of the cortex, including deep in the cortex and along the sub-periosteal margin. In some individuals, there is variability in the type of growth mark that is deposited among the skeletal elements sampled. For example, the first (i.e., osteologically deeper) growth mark of IVPP V 26548 varies from a LAG in the femur and tibia, to an annulus in the fibula. Interestingly, the largest and presumably most skeletally mature femur that was histologically sampled, shows uninterrupted cortical growth, suggesting that *Lystrosaurus* from the Jiucaiyuan Formation had a high intrinsic rate of growth that could periodically arrest.

Formatted: Not Highlight

Formatted: Not Highlight

Formatted: Font: (Default) Times New Roman, 12 pt

Formatted: Font: (Default) Times New Roman, 12 pt

Formatted: Not Highlight

Formatted: Highlight

Our results are consistent with a recent report of *Lystrosaurus* bone histology from the same formation with respect to the preponderance of highly vascularized, rapidly deposited cortical bone [40]. However, contrary to Han et al. [40], we do not find evidence of peripherally slowed growth rates in individuals that are as large (~60% MKS) and larger than the maximum size sampled in their study. Additionally, we pose an alternative interpretation to the assignment of LAGs in the laminar to plexiform tissue reported in ([40]:fig 2G-H, fig 3C, fig 5, fig 6D, G-H) as bright lines that appear in the context of periosteal ~~laminar~~ bone formation ([90]:fig3C; [91]:fig3). Finally, we report fewer instances of ~~LAGs~~growth marks, particularly in the smallest size class where no evidence of episodic growth marks is seen (contra [40]). When present, we can confidently trace 1 to 3 growth marks (as either LAGs or annuli) around the circumference of individuals that are approximately 70% maximum known size.

Formatted: Line spacing: Double

Size Class II

The smallest association of elements includes cranial and postcranial material (hind limb and forelimb elements) that was collected in situ. A femur, tibia, fibula, ulna, and radius were selected for consumptive sampling (composite images available on Morphobank under project number P4023). The femur measures 122.38 mm in length. When compared to femora from larger, presumably more mature individuals, this femur (IVPP V 27126a) is approximately 50% maximum known size. The bone tissue ~~microstructure~~ in each of the five associated limb elements is extremely well-vascularized with ~~plexiform to radial vascular canal~~lonsgitudinally-oriented primary osteons arranged in circumferential layers with some radial anastomoses (Fig 3). Woven fibered bone matrix and large, densely distributed osteocyte lacunae surround the primary vascular canals (Fig 3B, E). Vascular canal size and density remain consistently high

from the endosteal to the periosteal margin indicating that this individual grew rapidly until death (Fig 3D-E). There is no evidence of episodic growth ~~arrests~~ cycles (as either LAGs or annuli) in this individual.

Figure 3. Representative bone histology of Size Class II from the mid-shafts of *Lystrosaurus* sp. ~~forelimb and~~ hind limb elements. **A**, fibula (IVPP V 27124-1) consisting of well-vascularized ~~plexiform~~ tissue with longitudinally-oriented primary osteons in circumferential layers. **B**, 10X magnification of yellow square in **A**, arrows indicate ~~regions of woven bone matrix in the laminar, fibrolamellar~~bright lines in the extracellular matrix ~~complex that~~of periosteally accreted bone and should not be confused as ~~LAGs~~growth marks. **C**, 20X magnification of **B**. **D**, fibula (IVPP V 27126c) cross section consisting of ~~plexiform~~ longitudinally and radially oriented ~~canals~~primary osteons and dense osteocyte lacunae. **E**, 20X magnification of fibula (IVPP V 27126c)~~cross section of the ulna from the same individual (IVPP V27126c) similarly~~ shows woven-fibered bone (yellow arrows) in the peripheral cortex ~~a cortex of highly vascularized plexiform tissue~~ indicating rapid bone deposition at death. **F**, cross section of a proximal tibia (IVPP V 27124-3) with a slight decrease in vascular canal sizes towards the sub-periosteal edge, magnified in **G**. wb= woven-fibered bone; po= primary osteon. Scale bars = 1 mm in **A** & **F**, 500 µm in **D** & ~~, **E**,~~ **G**, 100 µm in **B**, **C** & **E**.

A right proximal fibula (IVPP V 27124-1), right proximal tibia (IVPP V 27124-3), distal tibia (IVPP V 27124-4), distal femur (IVPP V 27124-6) and complete femur (IVPP V 27124-7) were collected individually but are analyzed together here as immature individuals ~~as~~ because limb dimensions and histological features are indicative of sustained, active bone growth at

Formatted: Line spacing: Double

Formatted: Font: Not Bold

Formatted: Font: Bold

death. The complete femur is approximately 50% maximum known size, similar to IVPP V 27126a. In thin section, each of these disassociated elements has a large medullary cavity infilled with a loose trabecular network. Cortical tissue consists of ~~fibrolamellar~~ woven-fibered bone with ~~plexiform to~~ longitudinally ~~canal~~ orient~~ations~~ed primary osteons loosely arranged in circumferential rows. In the proximal tibia, vascular canal size decreases substantially in a regionalized band deep to the sub-periosteal edge but it rebounds to the typical highly vascularized tissue more peripherally, likely representing a temporary shift in growth rate (Fig 3F-G). In the fibula, there are regional differences in vascular canal size and organization that likely represent functional differences in bone apposition rates ~~(Currey 2003~~[92]~~)~~. For example, longitudinal vascular canals are organized into stacked, circumferential layers on the medial side of the proximal fibula (IVPP V 27124-1) (Fig 3B-C) ~~(IVPP V27124-1)~~ whereas the lateral side is less organized bone (Fig 3A). The amount of densely packed osteocyte lacunae in woven-fibered bone is higher in the less organized areas, but many large, globular lacunae are still apparent between primary osteons and in thin sheets in the more organized regions (Fig 3B-C). In more organized areas of the highly vascularized ~~fibrolamellar~~ tissue, the mineralized matrix surrounding the ~~reticular~~ vascular canals should not be confused ~~for~~ with lines of arrested growth (Fig 3B-C). At low magnifications, delineated bands of extracellular matrix separate the primary vascular space in periosteally accreted~~laminar~~ bone [91]~~)~~. However, at high magnifications, these ~~mineralized bands~~accretion lines, or bright lines (sensu [91]), do not form continuous rings around the cortex~~, nor are they hyper-mineralized, two of the most diagnostic characteristics of lines of arrested growth~~and should not be confused as growth marks or as slower forming tissues like parallel-fibered or lamellar bone [45]. Bright lines lack osteocyte lacunae and represent the saltatory activity of the periosteum when new bone is accreted [45, 91]. In specimens in Size

Class II, ~~H~~highly vascularized longitudinal canals in~~,~~ ~~reticular fibrolamellar~~woven- to parallel-fibered matrix ~~bone~~ continue~~s~~ to the sub-periosteal edg~~e in these specimens in Size Class II~~e, indicating that individuals died while actively growing.

Size Class III

A femur (IVPP V 27125a), tibia (IVPP V 27125b), and fibula (IVPP V 27125c) are associated as one individual that is approximately 70% maximum size based on femoral length. Unlike smaller sampled elements, the cortex is interrupted by two growth marks (Fig 4). In all associated elements, a deep cortical LAG is sandwiched between large vascular canals towards the endosteal margin and smaller vascular canals more peripherally (Fig 4A-B, lowermost arrows in F, K). In the fibula, the LAG is superficial to a region of immature secondary osteons that sit in the deep cortex (Fig 4J). The second of the two growth marks is more variable, in part due to the fragmentary nature of the subperiosteal edge of the femur. In the femur, a ~~a region of stacked, circumferentially organized, and~~ poorly vascularized annulus of parallel-fibered to lamellar tissue sits along the sub-periosteal ~~edge~~edge and is more apparent in thin areas of the cortex (Fig 4D). By contrast, thicker regions of the outer cortex show a mixture of longitudinal and circular ~~continuous laminar to fibrolamellar bone tissue~~primary osteons in circumferential layers that approaches a laminar organization that continues to the incomplete sub-periosteal edge (Fig 4C). In the tibia and fibula, the outer cortical growth mark is made up of two LAGs in the tibia, and an annulus of ~~stacked~~ avascular parallel-fibered ~~LAGs~~ tissue ~~that vary in number around the cross-section of~~in the fibula (Fig 4E, J-K). Interestingly, the mineral preservation of the tibia and fibula are such that the mineralized bone matrix is clearly visible between each circumferential layer of vascular canals (Fig 4F, K, L). These thin, mineralized lines of

extracellular matrix should not be confused for ~~LAGs~~ growth marks and instead are the mineralizing front of ~~laminar, fibrolamellar~~periosteally accreted bone ~~deposition and growth~~ [45, 90]. The vascular orientation shifts in the hind limb elements of this individual from ~~plexiform~~ reticular to laminar in the femur (Fig 4B), and from disorganized ~~longitudinal~~longitudinally oriented primary osteons to circumferentially organized longitudinal primary osteons in the tibia and fibula (Fig 4F, I). ~~in the middle to outer cortex.~~ In addition, ~~vascular canal size~~primary osteon size decreases~~s~~ towards the outermost periosteal margin but vascular canals remain~~s~~ open to the sub-periosteal edge (Fig 4F-G). The only exception to this pattern is in the thinnest region of the cortices, particularly in the fibula, where the sub-periosteal edge is avascular and ~~instead,~~ consists of well organized, ~~lamellar~~ parallel-fibered bone ~~tissue~~ (Fig 4D, I, K). However, this ~~lamellar~~ organized tissue is discontinuous around the cross section, suggesting that bone growth slowed at differential rates in regions of the outer cortex, but did not fully arrest.

Figure 4. Bone histology of associated hind limb elements of *Lystrosaurus* sp. representative of Size Class III. **A**, femur (IVPP V 27125a) with a deep cortical LAG in **B** (blue box in **A**). The outer cortex has regions of high vascularity seen in **C** (yellow box in **A**), and regions ~~of lower~~marked by a nearly continuous peripheral growth mark of parallel-fibered tissue ~~vascularity seen~~(denoted by yellow brackets) in **D** (orange box in **A**)~~which also has what appears to be the mineralization front of plexiform bone (denoted by yellow brackets throughout D)~~. **E**, tibia (IVPP V 27125b) has two cortical growth marks seen in **F** (yellow box in **E**; yellow arrows in **F**). The outer growth mark can variably be seen as ~~two~~ a double LAG~~s,~~ in **G** (green box in **E**; yellow arrows in **G**). **H**, the fibula (IVPP V 27125c) has a thinner cortex interrupted by ~~two~~ a two LAG~~s~~ seen in **I** (yellow box in **H**; yellow arrows in **I**). **J**, magnification of yellow box in **I**

Formatted: Line spacing: Double

Formatted: Font: Bold

shows the cross-cutting inner cortical LAG (arrow in **J**). **K**, thinner regions of the cortex appear avascular (purple box in **H**), with stacked mineralized lamellae of parallel-fibered bone (yellow bracket), whereas thicker regions (blue box in **H**) show peripheral pulses of vascularized growth, within the lamellated tissue, seen in **L** (yellow bracket). Scale bars = 5 mm in **A**, 1 mm in **E**, 2 mm in **H**, 500 µm in **B**, **C**, **F** & **I**, and 100 µm in **D**, **G**, **J** - **L**.

A slightly larger specimen (IVPP V 26548) is an association of hind limb and forelimb elements as well as at least one rib that was previously histologically sampled by Han et al. 2021[40]. The femur (IVPP V 26548a), tibia (IVPP V 26548b), and fibula (IVPP V 26548c) show plexiform tissue interrupted by two growth marks, with some variation in the outer growth mark, especially in the fibula where it splits into two LAGs (Fig 5). In the femur and tibia, the deeper growth mark is distinguishable as a dramatic reduction in vascular canal size, immediately followed by a return to the typical, large vascular canals in the middle of the cortex (Fig 5A, B, D). Throughout the middle cortex of the femur, the deeper growth mark changes in thickness. In thinner regions of the cortex, the growth mark appears as a cross-cutting LAG separating smaller vascular canals from larger canals more superficially. In the thicker regions of the cortex, the growth mark appears as an annulus, or region of little to no vasculature that is not accompanied by a hyper-mineralized line (Fig 5D). In both the femur and tibia, vascular canals reduce in size again towards the sub-periosteal margin, making up the second growth mark of an annulus of ~~stacked~~ ~~lamellar~~avascular parallel-fibered to lamellar tissue (Fig 5C). ~~In contrast, t~~The fibula shows a mid-cortical ~~growth mark~~annulus that is made up of ~~multiple stacked LAGs in a circumferential band of reduced vascularity~~avascular parallel-fibered to lamellar tissue (Fig 5G~~, I~~). Similar to the femur and tibia, there is a sub-periosteal reduction in vascular canal size with at

Formatted: Highlight

least two LAGs that can be traced around the entire cortex of the fibula, indicating variation in the type of growth mark deposited in hind limb elements of the same individual (Fig 5E, G, H). ~~We tentatively interpret the presence of stacked lamellar layers in this individual as a cessation in growth. However, s~~Small, simple vascular canals sit within the context of this ~~organized~~ peripheral lamellar tissue, which is atypical for an external fundamental system (EFS) indicative of somatic maturity, making it more likely that this lamellar tissue represents a temporary cessation in growth, similar to the annulus~~i~~ recorded in the deeper cortex.

Figure 5. Bone histology of associated elements of *Lystrosaurus* sp. representative of Size Class III. **A**, femur (IVPP V 26548a) has two growth marks. **B**, a deep, mid-cortical annulus (yellow box in **A**; inner yellow bracket in **B**), ~~stacked~~ avascular lamellar tissue makes up the second growth mark (outer yellow bracket in **B**). **C**, magnification of peripheral lamellar tissue, left panel reflects ~~(~~blue box in **A**, right panel reflects green box in **A**. ~~; yellow bracket in C).~~ **D**, first (osteologically deeper) annulus (yellow bracket) in plane and cross-polarized light with a lambda filter. **E**, magnification of blue box in **A** of outer cortical growth mark that varies in thickness around the periphery of the thin section, here consisting of two LAGs. **F**, associated fibula (IVPP V 26548c) highlights a similar arrangement of deep and more superficial growth marks consisting of, at times, numerous layers of lamellar tissue in an annulus. **G**, magnification of yellow box in **F** showing two growth marks; annulus (yellow bracket) and peripheral LAGs (yellow arrows). **H**, magnification of green box in **F** showing variation in the outer growth mark such that in some regions it appears as a thicker region of lamellar tissue with one cross-cutting LAG. **I**, ~~magnification of blue box in F; deep cortical annulus (bracket) in plane and cross polarized light with lambda filter~~high magnification image of the associated tibia (IVPP V

Formatted: Line spacing: Double

Formatted: Font: Bold

26548b) showing the presence of woven-fibered bone between primary osteons within the middle cortex. **J**, humerus with mid-cortical annulus of reduced vascularity. **K**, magnification of yellow box in **J** of ~~growth mark~~annulus (yellow bracket). **L**, high magnification of parallel-fibered bone making up the annulus in the humerus (orange box in J). **M**, ulna (IVPP V 27124-2) with a thinner overall cortex ~~and reduced vasculature~~made up of parallel-fibered and small amounts of woven-fibered bone. ~~**M**~~**N**, magnification of yellow box in ~~**L**~~ **M** highlighting the ~~disorganized~~ thin cortex in plane and cross-polarized light with a lambda filter. **O**, high magnification of outer cortex of the ulna showing small areas of woven-fibered bone (blue box in **M**). wb= woven-fibered bone; pfb= parallel-fibered bone. Scale bars = 1 mm in **A**, **B**, **F**, **J**, & ~~**L**~~**M**, 500 µm in **E**, **G** &~~, **I**,~~ **K**, and 100 µm in **C**, **D**, **H**, **I**, ~~**M**~~**L**, **N**, & **O**.

A complete humerus (IVPP V 27124-8) of comparable size to IVPP V 26548 [40], has an exceedingly thick cortex with one mid-cortical growth mark (Fig 5J). The medullary cavity is nearly infilled by a dense network of trabecular bone. The cortex increases in thickness in the anterodorsal region (up in Fig 5J) and consists of highly vascularized laminar ~~to fibrolamellar~~ bone in woven- and parallel-fibered bone matrix. Vascular canals have a circular ~~to plexiform~~ orientation and show a marked decrease in size coincident with a mid-cortical LAG (Fig 5K). The LAG is traceable around the entire cross-section and is followed by a pulse of highly vascularized ~~reticular~~ tissue with large canal spaces (Fig 5K). Vascular canal size reduces slightly towards the sub-periosteal margin which could indicate the beginning of another growth mark, similar to the mid-cortical growth mark.

Formatted: Font: Bold

Formatted: Font: Bold

Formatted: Font: Bold

Formatted: Font: Bold

Formatted: Font: Not Bold

Formatted: Font: Not Bold

Formatted: Font: Not Bold

A distal fragment of an ulna (IVPP V 27124-2; original complete length estimated to be 93mm) ~~A fragment of a distal ulna (IVPP V27124-2) is estimated to be 93 mm long and~~ has a thin cortex with a large medullary cavity infilled with a loose network of trabecular bone (Fig 5M~~L~~). The transition from trabeculae to compact cortical bone includes a region of enlarged resorption cavities and immature secondary osteons. The middle and outer cortex is composed of small longitudinal ~~and reticular~~ canals that sit in a parallel-fibered matrix where small regions of woven-fibered bone persist (Fig 5N, O). ~~where small, lenticular osteocyte lacunae are sparsely distributed.~~ Along the sub-periosteal margin, vascular canals are reduced but occasionally open to the bone edge, suggesting that this individual died while still growing (Fig 5M). The ~~overall construction of this element is suggestive of a slower growth rate when compared to other long bones~~proportion of parallel-fibered bone in this element is higher than in hind limb elements, however, there is no evidence to suggest somatic maturity or attainment of maximum size.

Size Class IV

Unlike the middle size class, the largest sampled femur (IVPP V 27127) does not have LAGs or annuli (Fig 6A). Despite cracks in ~~the~~ its cortex, the bone tissue architecture is easily discernible and consists of ~~longitudinal canals in plexiform to circular organization~~laminar canals in woven- and parallel-fibered matrix based on the lacunar morphologies at high magnification (Fig 6B, C). Woven-fibered ~~The~~ bone makes up more of the extracellular matrix in the deep and middle cortex whereas the outer cortex consists of parallel-fibered bone with small areas of woven-fibered bone (Fig 6C). ~~fiber orientation is less clear due to the poor preservation of this specimen but appears parallel-fibered in the deep cortex and woven in the outer cortex, of which, only a small portion remains intact (Fig 6A-C).~~ There is no indication of

transition to slowed bone deposition in the small regions of sub-periosteal bone that remain intact, suggesting a sustained rate of growth at death (Fig 6B).

Figure 6. Bone histology of the largest femur (IVPP V 27127) of *Lystrosaurus* sp. that represents Size Class IV and rib fragments of unknown size. **A**, the femur is cracked (yellow arrow is a large circular crack) ~~and incomplete~~ but preserves regions of the entire cortex (yellow box). **B**, the same view of the yellow box in **A** using cross-polarized light with a lambda filter shows ~~parallel-fibered bone in the mid cortex, a large crack (yellow arrow), and~~ well-vascularized, plexiform tissue along the sub-periosteal margin (yellow box in **A**~~**B**~~). **C**, left panel shows ~~magnification of yellow box in **B** shows sustained, rapid growth at death~~woven-fibered bone in the deep-middle cortex (blue box in **A**) and outer cortex, right panel (green box in **A**). ~~.~~ **D**, rib fragments (IVPP V 27124-5) have infilled medullary cavities, secondary osteons within the deep cortex and a largely avascular compact outer cortex consisting of organized lamellar tissue~~an EFS~~. **E**, magnification of yellow box in **D** showing ~~an EFS~~the peripheral lamellar tissue in the yellow bracket. **F**, magnification of the yellow box in **E** under plane and cross-polarized light with a lambda filter. **G**, magnification of blue box in **D** similarly shows that ~~an EFS is present~~peripheral lamellar tissue is present (yellow bracket). wb= woven-fibered bone. Scale bars = 10 mm in **A**, 1 mm in ~~**B** &~~ **D**, 500 µm in **B** & ~~**C** &~~ **G**, 100 µm in **C**, **E** & **F**.

Formatted: Line spacing: Double

Formatted: Font: Bold

Formatted: Font: Bold

Formatted: Font: Bold

Formatted: Font: Bold

Unknown Size Class

Four partial ribs (IVPP V 27124-5) belonging to an individual of unknown size class are preserved in a block of sediment (Fig 6D). In thin section, they have large, infilled medullary cavities that grade into thin, compact cortices. Scarce vascular canals and immature secondary

osteons sit in the deeper cortex whereas the outer cortex is avascular ~~with an EFS of stacked, circumferential LAGs~~and consists of parallel-fibered to lamellar tissue with well-organized osteocyte lacunae (Fig 6E-F~~G~~). Due to the fragmentary nature of this specimen and the fact that it is not associated with limb bones, it is unclear whether the peripheral lamellar tissue is indicative of a EFS or a temporary cessation in growth. Additionally, ribs grow at different rates than limbs and do not preserve the same record of life history information compared to a midshaft hind limb thin-section. ~~Osteocyte lacunae are well organized in this lamellar tissue (Fig 6F).~~

Discussion

All *Lystrosaurus* individuals sampled from the Jiucaiyuan Formation show abundant, highly vascularized primary bone tissue throughout the majority of the cortices of the hind limb, humerus, and radius. The ulna and ribs showed fewer vascular canals, overall thinner cortices, and more organized primary bone tissue. Three size classes were identified and histologically described as: II) a highly vascularized cortex of ~~radial~~ circumferential layers of radial and longitudinal ~~to plexiform~~ vascular canals in ~50% max known size individuals: III) highly vascularized plexiform tissue interrupted by one to two growth marks that range from one or two closely spaced LAGs ~~to~~ or annuli in ~70% maximum known size individuals; and IV) uninterrupted ~~plexiform~~ laminar tissue in a 100% maximum known size individual. These results force us to reconsider what an adult-sized specimen might look like for *Lystrosaurus* sp., as the current sample lacks histologic indicators of somatically maturity (e.g.,~~i.e.~~ an external fundamental system) [78]. Botha [22] found similar results for South African species of

Lystrosaurus, suggesting that species typified by small body size (viz. *L. murrayi*) were very likely capable of reaching much larger sizes, but never lived long enough to do so.

Han et al. [40] suggested that sub-adult *Lystrosaurus* from northern Pangea may have reached some level of somatic maturity inferred from parallel-fibered bone and peripheral LAGs in a dorsal rib. However, histological data from the femur, tibia, and fibula of the same individual indicate episodic cessations in growth instead of an overall pattern of slowed growth. The presence of slower growing bone tissue in the ribs likely reflects differential growth rates seen throughout the skeleton, rather than somatic maturity [93, 49].

Formatted: Line spacing: Double

Growth Marks

Unlike the bone histology reported from South African and Indian *Lystrosaurus*, in which growth marks are rare and often limited to a single LAG or annulus, we find one, and more commonly, two growth marks in immature *Lystrosaurus* from the Jiucaiyuan Formation. The number of growth marks is mostly consistent across associated elements from the same individual as well as across individuals of the same size class (Table 1). Interestingly, the type of growth mark, as either a LAG, a series of closely spaced and occasionally bifurcating LAGs, an annulus consisting of ~~stacked LAGs~~lamellar tissue, or an annulus consisting of ~~avascular~~ parallel-fibered to lamellar tissue, varied throughout the current sample. In most cases, the first (i.e., osteologically deeper) growth mark, or singular growth mark in the case of the humerus, was often recorded as an annulus accompanied by a reduction of vascular canal sizes. The outer cortical growth marks were more variable, and often varied within the same thin section.

We did not find evidence of growth marks in individuals in Size Class II. These individuals had characteristic juvenile bone histology (i.e., large radial to ~~reticular~~ longitudinal

vascular canals that frequently anastomose); therefore our analysis disagrees with the assignment of LAGs in juvenile *Lystrosaurus* by Han et al. [40]:fig 3. In some cases, the mineralization front can be mis-identified as a ~~LAG~~ growth mark in the context of periosteally deposited bone. Currey [91] and others (e.g., [90])~~)laminar or plexiform tissue~~, describe laminar bone formation where acretted circumferential layers of bone (made up of a woven scaffold that is later infilled with parallel-fibered or lamellar bone in the form of primary osteons) is bordered by the periosteum. As the periosteum jumps peripherally when new bone is accreted, a bright line is left between each circumferential layer and represents the place at which the periosteum was located before new bone was added.~~wherein one unit of laminar bone is constructed by a mineralized line upon which woven bone is deposited (89).~~ This mineralized line differs from a ~~LAG~~ growth mark as it is not continuous around the cortex and is not hyper-mineralized but appears as a bright line often devoid of osteocyte lacunae [90]. ~~LAGs~~Growth marks, on the other hand, are continuous ~~mineralized lines~~ around the cortex, unless ~~there is clear evidence for~~ secondary remodeling or cortical remodeling ~~that~~ causes ~~disrupts~~ discontinuities (i.e., by erosion~~des~~) ~~it~~.

All growth marks observed in our analysis come from Size Class III, which corresponds to individuals that are approximately 70% maximum known size. We can confidently trace up to two growth marks ~~(occasionally made up of multiple LAGs)~~ in the current Chinese sample, which is higher than reports from South African and Indian specimens of *Lystrosaurus*, which generally lack growth marks. However, the remainder of the cortical bone tissue in the current Chinese sample reflects the similarly high and consistent rate of bone growth reported from South African and Indian *Lystrosaurus* [22] [54]. A slight increase in the number of growth marks in northern Pangean *Lystrosaurus* could be explained by one or two possible factors that are not mutually exclusive: (i) by an increase in their lifespan or (ii) by environmental stressors.

Formatted: Font: (Default) Times New Roman, 12 pt

Formatted: Font: (Default) Times New Roman, 12 pt

Formatted: Font: (Default) Times New Roman, 12 pt

Formatted: Font: (Default) Times New Roman, 12 pt

Formatted: Font: (Default) Times New Roman, 12 pt

Formatted: Font: (Default) Times New Roman, 12 pt

There is a longstanding hypothesis that the Permo-Triassic extinction caused a Lilliput effect, a persistent reduction in body size, in surviving terrestrial lineages [94-97, 19]. This claim has been investigated histologically in therocephalians, which support an overall reduction in body size following the ~~end~~ extinction ~~Permian~~ [97, 19]. A Lilliput effect was also posited for *Lystrosaurus*, based in large part on the overwhelming abundance of small skulls in the Karoo Basin and museum collections in South Africa [98, 99, 20, 21]. However, Botha [22] later rejected a strict Lilliput effect for southern Pangean *Lystrosaurus* (based on fossils from South Africa and India) ~~based on~~because of the lack of mature histological tissue in all of the species examined [22]. Instead, she [22] suggested that the large proportion of small skulls in the Triassic was better explained by high mortality in immature individuals, as assessed by their long bone histology.

Formatted: Line spacing: Double

Body size

~~In order to~~To better understand how *Lystrosaurus* from the Jiucaiyuan Formation compare to congeners from more southerly parts of Pangea, we gathered body size data (based on basal skull length and femur length) from the literature [54, 22, 40] and personal observations ~~from~~ of Early Triassic-aged specimens collected in China, South Africa, and India. From this sample, it is clear that Chinese *Lystrosaurus* have a larger average body size (Fig 7, Fig 8). However, cranial size distributions suggest that both populations from South Africa and China had the potential to grow to similar maximum recorded sizes (Fig 7). The lack of large specimens of *Lystrosaurus* in South Africa aligns with previous interpretations for increased mortality at small sizes there by Botha [22]; also see [20]. Our data ~~also~~ provide evidence suggesting that northern Pangean *Lystrosaurus* could have reached larger sizes more readily or

easily. However, it is unclear the degree to which differences in sampling practices might affect the body size distributions seen here. A decade of detailed stratigraphic work targeting the Permo-Triassic boundary in South Africa has led to more comprehensive fossil sampling, which should provide a relatively unbiased view of the vertebrate fauna [100, 101]. However, historical collections from the Karoo likely systematically emphasized rare components of the assemblage (e.g., [102]). Likewise, the Permo-Triassic vertebrate record from Xinjiang has not been subject to the same intensive collecting as that of the Karoo Basin, and therefore it currently may not provide an unbiased view of body size distributions or relative abundances of taxa.

Figure 7. Interspecific body size distributions from Early Triassic *Lystrosaurus* show an abundance of large individuals from China. ~~South African and Chinese species~~Species from South Africa and China overlap in maximum basal skull length but individuals from China have larger average size. See S2 ~~Supplementary~~ Dataset for more information.

Formatted: Line spacing: Double

Figure 8. Interspecific femoral lengths from Early Triassic *Lystrosaurus* show a higher frequency of large sizes from China. Size Classes denote femora that are less than 40% maximum known size (MKS) in I, between 40-65% MKS in II, approximately 70-75% MKS in III, and greater than 79% in IV. See S2 Dataset for more information.

The presence of large individuals with episodic growth marks in all of the subadult-sized elements suggests that *Lystrosaurus* from the Jiucaiyuan Formation were able to reach large sizes through prolonged and sustained rapid growth, unlike Triassic *Lystrosaurus* from South Africa that rarely lived long enough to reach large body sizes despite growing rapidly (Fig 7).

There are several possible explanations for this pattern. First, physiological differences among the species of *Lystrosaurus* could be responsible. This proposal is difficult to test, as the alpha-taxonomy of Chinese *Lystrosaurus* is in need of revision and phylogenetic studies of the genus have typically only included *L. hedini* as a sole northern hemisphere representative (e.g., [103]). However, to the degree that patterns preserved in hard tissue histology can shed light on physiology, the current data suggests that *L. curvatus*, *L. declivis*, *L. maccaigi*, and *L. murrayi* are effectively indistinguishable [22, 31], which suggests a degree of consistency within the genus.

Climatic differences in the high-southern (60°S) versus mid-northern (45°N) paleolatitudes [104, 105] is another possible explanation for the observed difference in body size and inferred lifespan in *Lystrosaurus*. Paleoclimate reconstructions of the latest Permian to earliest Triassic show extreme increases in temperature and aridity [106-109], mega monsoons [110, 111], chemical weathering, soil erosion, and geochemical and isotopic signatures of mass wasting and greenhouse conditions [112, 8,7/30/2021 10:05:00 PM113]. Facies that span the terrestrial Permo-Triassic boundary of the Karoo Basin are interpreted as a change from meandering rivers to low-sinuosity braid-plains, resulting from increased aridity and a die-off of rooted plants that supported river morphologies [114, 115]. However, more recent evidence from isotopic and geochemical data produced differing estimates of the degree to which aridity increased in the Karoo Basin, complicating our understanding of seasonal variation in precipitation and resource availability [111, 116, 117]. ~~Nonetheless~~Nevertheless, conditions were disruptive enough to have destabilized terrestrial communities and caused a prolonged interval of faunal turnover in southern Pangea during the extinction interval [118, 15, 18].

The extreme environmental and climatic conditions of southern Pangea are not reflected in the paleoclimate reconstructions of the Juicaiyuan Formation [74]. Here, relatively stable subhumid to semiarid conditions are interpreted from cyclostratigraphic and pal~~s~~eosol analyses in the South Taodonggou study area [75, 74]. Furthermore, fluvial and lacustrine strata host a diversity of plant, root, and wood fossils that are occasionally preserved in situ and show signatures of wildfires during the Early Triassic [74, 119-121]. ~~and do not preserve cyclical growth marks when thin-sectioned (112) (113) (114)(73). The absence of growth rings in these woody pieces provides some evidence for stable environmental and climatic conditions.~~ In sum, evidence from the paleobotanical record of the Jiucaiyuan Formation suggests a lush and vegetated environment that hosted a diverse terrestrial fauna in earliest Triassic times [64, 40]. Taken together, there is mounting evidence from sedimentologic, isotopic, and cyclostratigraphic data that points to seasonal, semi-arid conditions in the Karoo Basin and stable, sub-humid conditions in the Turpan-Hami Basin.

The extreme environmental and climatic conditions inferred from the Karoo Basin likely truncated *Lystrosaurus* lifespans in the Early Triassic. Similar environmental constraints on maximum size and longevity are also known from Early Triassic therocephalians and terrestrial temnospondyls, which also lack ~~LAGs~~ growth marks [19, 52]. By contrast, we propose that the more favorable environmental conditions and vegetated landscape interpreted from the Jiucaiyuan Formation could have hosted longer-lived *Lystrosaurus* during the Early Triassic. Relatively stable environmental conditions could have allowed populations of *Lystrosaurus* to reach larger average body sizes than their southern Pangean relatives. The prevalence of multiple growth marks in some, but not all, immature individuals, further supports developmentally plastic growth in *Lystrosaurus* first proposed for southern Pangean populations [22]. It also

suggests that some individuals experienced periodic arrests of growth, perhaps associated with environmental disruptions, but that these disturbances may not have caused widespread mortality on the scale suggested by the South African record. Similarly, some individuals apparently experienced sufficiently stable conditions for them to grow to comparatively large size without interruption. Alternatively, the uninterrupted growth record of the largest specimen sampled here could also be due to the higher stratigraphic position of this individual; it is possible that upper Juicaiyuan rocks represent more favorable environmental conditions. Finally, the absence of peripheral parallel-fibered bone or consistent, circumferential lamellar tissue in the current sample indicates that the maximum size of *Lystrosaurus* from the Jiucaiyuan Formation remains unknown.

Formatted: Line spacing: Double

Conclusions

We present bone histology, cranial geometric morphometric analyses, and body size data for *Lystrosaurus* from the Jiucaiyuan Formation of northwestern China and report statistically different cranial morphologies, an extended lifespan, and larger average body size compared to *Lystrosaurus* living in southern Pangea during Early Triassic times. In addition, we report up to two growth marks in immature individuals, which is inconsistent with previous reports of *Lystrosaurus* life histories from the Turpan-Hami Basin [40]. We suggest that it is unlikely that the growth marks reported here represent regular, cyclical patterns and more likely represent instances where bone growth stopped due to unfavorable environmental conditions. Indeed, the absence of growth marks in the largest sampled individual indicates an intrinsically high rate of growth that persisted throughout ontogeny. In addition, the presence of two cessations in growth in individuals from Size Class III indicates that smaller, likely more immature, individuals may

have been more susceptible to fluctuations in environmental conditions. However, appositional bone growth rebounded to a rapid rate following these cessations, indicating a flexible physiology, as previously proposed for higher-latitude populations of *Lystrosaurus* [22, 31]. A better sample of large limb bones (>250 mm) as well as an analysis of tusk dentine deposition, are needed to clarify what impact the Early Triassic environment may have had on *Lystrosaurus* growth and development in northern Pangea.

Supporting Information

S1 Supporting Information. Figures and corresponding tables for cranial geometric morphometric analysis and bone wall thickness measurements. (PDF)

S2 Dataset. Basal skull length measurements (BSL) and femoral length measurements for Early Triassic *Lystrosaurus*. (Excel Spreadsheet)

Acknowledgements. — ~~This research was supported by National Science Foundation grants EAR 1714829 (to KDA) and EAR 1713787 (to CAS).~~ At the IVPP, we thank ~~Yi~~ YI Jian and ~~SHUKANG~~ SHUKANG Zhang for assistance with thin section preparation as well as ~~Jun~~ LIU Jun~~Liu~~ for access to field data and specimen collections. We also thank Alida Bailleul for helpful discussion and microscope access. Collecting and compiling the entirety of the comparative datasets would not have been possible if it weren't for the following museum curators, collection managers, colleagues, and friends who we deeply thank: Zaituna Skosan, Claire Browning, Christian Kammerer, Roger Smith, Jennifer Botha, Elize Butler, Bernhard Zipfel, Bruce Rubidge, Sifelani Jirah, Viktor Radermacher, Carl Mehling, and Mark Norell. For

Formatted: Font: Bold

Formatted: Font: Bold

Formatted: Font: Bold

Formatted: Font: Bold

Formatted: Font: Bold, Italic

Formatted: Font: Bold

Formatted: Font: Bold

Formatted: Font: 12 pt, Bold

their helpful discussion on earlier versions of this manuscript, we thank Bryan Gee, Savannah Olroyd, and Elliott Armour Smith. Helpful comments that greatly improved the quality of this work were given by Jennifer Botha and one anonymous reviewer. Finally, we are indebted to Wan Yang for collecting some of the fossils analyzed here, organizing fieldwork in 2019 and leading the multidisciplinary project that instigated this research.

References

1. Raup DM, Sepkoski JJ. Periodicity of extinctions in the geologic past. Proc Natl Acad Sci. 1984 Feb 1;81(3):801–5.
2. Erwin DH. The Permo–Triassic extinction. Nature. 1994 Jan;367(6460):231–6.
3. Chen Z-Q, Benton MJ. The timing and pattern of biotic recovery following the end-Permian mass extinction. Nat Geosci. 2012 Jun;5(6):375–83.
4. Benton M. Diversification and extinction in the history of life. Science. 1995 Apr 7;268(5207):52–8.
5. Benton MJ, Twitchett RJ. How to kill (almost) all life: the end-Permian extinction event. Trends Ecol Evol. 2003 Jul;18(7):358–65.
6. Sahney S, Benton MJ. Recovery from the most profound mass extinction of all time. Proc R Soc B Biol Sci. 2008 Apr 7;275(1636):759–65.
7. Sobolev SV, Sobolev AV, Kuzmin DV, Krivolutskaya NA, Petrunin AG, Arndt NT, et al. Linking mantle plumes, large igneous provinces and environmental catastrophes. Nature. 2011 Sep;477(7364):312–6.
8. Benton MJ, Newell AJ. Impacts of global warming on Permo-Triassic terrestrial ecosystems. Gondwana Res. 2014 May;25(4):1308–37.
9. Retallack GJ, Krull ES. Carbon isotopic evidence for terminal-Permian methane outbursts and their role in extinctions of animals, plants, coral reefs, and peat swamps. In: Greb SF, DiMichele WA, editors. Wetlands Through Time: Geological Society of America Special Paper. 2006. p. 249–68.
10. Nowak H, Schneebeli-Hermann E, Kustatscher E. No mass extinction for land plants at the Permian–Triassic transition. Nat Commun. 2019 Dec;10(1):384.
11. Mishra S, Jha N, Stebbins A, Brookfield M, Hannigan R. Palaeoenvironments, flora, and organic carbon and nitrogen isotope changes across the non-marine Permian-Triassic

boundary at Wybung Head, Australia. Palaeogeogr Palaeoclimatol Palaeoecol. 2019 Nov;534:109292.

12. Botha J, Smith RMH. Rapid vertebrate recuperation in the Karoo Basin of South Africa following the End-Permian extinction. J Afr Earth Sci. 2006 Aug 1;45(4):502–14.

13. Botha J, Abdala F, Smith R. The oldest cynodont: new clues on the origin and early diversification of the Cynodontia. Zool J Linn Soc. 2007;149(3):477–92.

14. Fröbisch J. Vertebrate diversity across the end-Permian mass extinction — Separating biological and geological signals. Palaeogeogr Palaeoclimatol Palaeoecol. 2013 Feb;372:50–61.

15. Roopnarine PD, Angielczyk KD, Weik A, Dineen A. Ecological persistence, incumbency and reorganization in the Karoo Basin during the Permian-Triassic transition. Earth-Sci Rev. 2019 Feb;189:244–63.

16. Bernardi M, Petti FM, Benton MJ. Tetrapod distribution and temperature rise during the Permian–Triassic mass extinction. Proc R Soc B Biol Sci. 2018 Jan 10;285(1870):20172331.

17. Allen BJ, Wignall PB, Hill DJ, Saupe EE, Dunhill AM. The latitudinal diversity gradient of tetrapods across the Permo-Triassic mass extinction and recovery interval. Proc R Soc B Biol Sci. 2020 Jun 24;287(1929):20201125.

18. Viglietti PA, Benson RBJ, Smith RMH, Botha J, Kammerer CF, Skosan Z, et al. Evidence from South Africa for a protracted end-Permian extinction on land. Proc Natl Acad Sci. 2021 Apr 27;118(17):e2017045118.

19. Huttenlocker AK, Botha-Brink J. Bone microstructure and the evolution of growth patterns in Permo-Triassic therocephalians (Amniota, Therapsida) of South Africa. PeerJ. 2014 Apr 8;2:e325.

20. Botha-Brink J, Codron D, Huttenlocker AK, Angielczyk KD, Ruta M. Breeding Young as a Survival Strategy during Earth’s Greatest Mass Extinction. Sci Rep [Internet]. 2016 Jul;6(1). Available from: http://www.nature.com/articles/srep24053

21. Codron J, Botha-Brink J, Codron D, Huttenlocker AK, Angielczyk KD. Predator-prey interactions amongst Permo-Triassic terrestrial vertebrates as a deterministic factor influencing faunal collapse and turnover. J Evol Biol. 2017 Jan;30(1):40–54.

22. Botha J. The paleobiology and paleoecology of South African *Lystrosaurus*. PeerJ. 2020 Nov 24;8:e10408.

23. Nicolas M, Rubidge BS. Changes in Permo-Triassic terrestrial tetrapod ecological representation in the Beaufort Group (Karoo Supergroup) of South Africa. Lethaia. 2010 Mar;43(1):45–59.

24. Smith R, Rubidge BS, van der Walt M. Therapsid Biodiversity Patterns and Paleoenvironments of the Karoo Basin, South Africa (pp. 31-63) Roger Smith, Bruce Rubidge and Merrill van der Walt. In: The Forerunners of Mammals. Indiana University Press; 2012.

25. King GM, Cluver MA. The Aquatic *Lystrosaurus*: An Alternative Lifestyle. Hist Biol. 1991;4:323–41.

26. Jasinoski SC, Rayfield EJ, Chinsamy A. Comparative Feeding Biomechanics of *Lystrosaurus* and the Generalized Dicynodont Oudenodon. Anat Rec Adv Integr Anat Evol Biol. 2009 Jun 1;292(6):862–74.

27. Retallack GJ, Smith RMH, Ward PD. Vertebrate extinction across Permian–Triassic boundary in Karoo Basin, South Africa. Geol Soc Am Bull. 2003;115(9):1133.

28. Botha-Brink J. Burrowing in *Lystrosaurus*: preadaptation to a postextinction environment? J Vertebr Paleontol. 2017 Sep 3;37(5):e1365080.

29. Liu J, Angielczyk KD, Abdala F. Permo-Triassic tetrapods and their climate implications. Glob Planet Change. Forthcoming.

30. Botha-Brink J, Angielczyk KD. Do extraordinarily high growth rates in Permo-Triassic dicynodonts (Therapsida, Anomodontia) explain their success before and after the end-Permian extinction? Zool J Linn Soc. 2010 Oct 1;160(2):341–65.

31. Whitney MR, Sidor CA. Evidence of torpor in the tusks of *Lystrosaurus* from the Early Triassic of Antarctica. Commun Biol [Internet]. 2020;3(1). Available from: http://www.nature.com/articles/s42003-020-01207-6

32. Tripathi C, Puri SN. On the remains of *Lystrosaurus* from the Panchets of the Raniganj coalfield. Rec Geol Surv India. 1961;89:407–19.

33. Colbert EH. *Lystrosaurus* from Antarctica. Am Mus Novit. 1974;(2535):44.

34. Gubin Y, Sinitza S. Triassic terrestrial tetrapods of Mongolia and the geological structure of the Sain-Sar-Bulak locality. In: The Nonmarine Triassic. 1993. p. 169–70.

35. Liu J, Li J, Cheng Z. The *Lystrosaurus* fossils from Xinjiang and their bearing on the terrestrial permian triassic boundary. Vertebr Pal Asiat. 2002;40(4):267–75.

36. Sun A. Late Permian and Triassic Terrestrial Tetrapods of North China. Vertebr Palasiat. 1980;XVIII(2):11.

37. Ray S. *Lystrosaurus* (Therapsida, Dicynodontia) from India: Taxonomy, relative growth and Cranial dimorphism. J Syst Palaeontol. 2005 Jan;3(2):203–21.

38. Surkov MV, Kalandadze NN, Benton MJ. *Lystrosaurus georgi* , a dicynodont from the Lower Triassic of Russia. J Vertebr Paleontol. 2005 Jun 27;25(2):402–13.

39. Araújo R, Fernandez V, Polcyn MJ, Fröbisch J, Martins RMS. Aspects of gorgonopsian paleobiology and evolution: insights from the basicranium, occiput, osseous labyrinth, vasculature, and neuroanatomy. PeerJ. 2017 Apr 11;5:e3119.

40. Han F, Zhao Q, Liu J. Preliminary bone histological analysis of *Lystrosaurus* (Therapsida: Dicynodontia) from the Lower Triassic of North China, and its implication for lifestyle and environments after the end-Permian extinction. Fröbisch J, editor. PLOS ONE. 2021 Mar 18;16(3):e0248681.

41. Padian K, Lamm E-T, editors. Bone histology of fossil tetrapods: advancing methods, analysis, and interpretation. Berkeley: University of California Press; 2013. 285 p.

42. Köhler M, Marín-Moratalla N, Jordana X, Aanes R. Seasonal bone growth and physiology in endotherms shed light on dinosaur physiology. Nature. 2012 Jul;487(7407):358–61.

43. Castanet J, Croci S, Aujard F, Perret M, Cubo J, Margerie E de. Lines of arrested growth in bone and age estimation in a small primate: Microcebus murinus. J Zool. 2004 May 1;263(1):31–9.

44. Buffrénil V de, Quilhac A. Bone Tissue Types: A Brief Account of Currently Used Categories. In: Vertebrate Skeletal Histology and Paleohistology. S.l.: CRC PRESS; 2021. p. 147–82.

45. Francillon-Vieillot H, de Buffrénil V, Castanet J, Géraudie J, Meunier FJ, Sire JY, et al. Microstructure and Mineralization of Vertebrate Skeletal Tissues. In: Skeletal Biomineralization: Patterns, Processes and Evolutionary Trends [Internet]. American Geophysical Union; 1990. p. 175–234. Available from: http://dx.doi.org/10.1029/SC005p0175

46. Enlow H, Brown SO. A Comparative Histological Study of Fossil and Recent Bone Tissues. Part II. Tex J Sci. 1957;9(2):30.

47. Enlow H, Brown SO. A comparative histological study of fossil and recent bone tissues, Part III. Tex J Sci. 1958;10(2):187–230.

48. Castanet J, Newman DG, Girons HS. Skeletochronological Data on the Growth, Age, and Population Structure of the Tuatara, Sphenodon punctatus, on Stephens and Lady Alice Islands, New Zealand. 1988;14.

49. Castanet J. Time recording in bone microstructures of endothermic animals; functional relationships. Comptes Rendus Palevol. 2006 Mar;5(3–4):629–36.

50. Nacarino-Meneses C, Köhler M. Limb bone histology records birth in mammals. PLOS ONE. 2018 Jun 20;13(6):e0198511.

51. Sinsch U, Oromi N, Sanuy D. Growth marks in natterjack toad (Bufo calamita) bones: histological correlates of hibernation and aestivation periods. 2007;9.

52. Canoville A, Chinsamy A. Bone Microstructure of the Stereospondyl *Lydekkerina Huxleyi* Reveals Adaptive Strategies to the Harsh Post Permian-Extinction Environment: BONE MICROSTRUCTURE of LYDEKKERINA HUXLEYI. Anat Rec. 2015 Jul;298(7):1237–54.

53. Mchugh JB. Paleohistology of *Micropholis stowi* (Dissorophoidea) and *Lydekkerina huxleyi* (Lydekkerinidae) humeri from the Karoo Basin of South Africa, and implications for bone microstructure evolution in temnospondyl amphibians. J Vertebr Paleontol. 2015 Jan 2;35(1):e902845.

54. Ray S, Chinsamy A, Bandyopadhyay S. *Lystrosaurus murrayi* (therapsida, Dicynodontia): Bone Histology, Growth and Lifestyle Adaptations. Palaeontology. 2005 Nov 1;48(6):1169–85.

55. Yuan PL, Young CC. On the Occurrence of *Lystrosaurus* in Sinkiang. Bull Geol Soc China. 1934;13(1):575–80.

56. Young CC. On two skeletons of Dicynodontia. Bull Geol Soc China. 1935;(14):483–517.

57. Young CC. Additional Dicynodontia remains from Sinkiang. Bull Geol Soc China. 1939;(19):111–36.

58. Sun A-L. Preliminary report on a new species of *Lystrosaurus* of Sinkiang. Vertebr Pal Asiat. 1964;8:216–7.

59. Sun A-L. Permo-Triassic dicynodonts from Turfan, Sinkiang. Permo-Triassic Vertebr Foss Turfan Basin. 1973;53–68.

60. Cheng Z. Vertebrates. In: Permian and Triassic Strata and Fossil Assemblages in the Dalongkou Area of Jimsar, Xinjiang. 1986. p. 207–18. (People's Republic of China Ministry of Geology and Mineral Resources Geological Memoirs).

61. Cluver MA. The cranial morphology of the dicynodont genus *Lystrosaurus* [Doctoral dissertation]. [Stellenbosch]: Stellenbosch University; 1970.

62. Cosgriff JW, Hammer WR, Ryan WJ. The Pangaean Reptile, *Lystrosaurus maccaigi*, in the Lower Triassic of Antarctica. J Paleontol. 1982;56(2):371–85.

63. King G. Anomodontia. Encycl Paleoherpetology. 1988;1–74.

64. Lucas SG. Chinese Fossil Vertebrates. Columbia University Press; 2001. 390 p.

65. Li J, Sun A. Subclass Synapsida. In: The Chinese Fossil Reptiles and Their Kin. Sci. Press; 2008. p. 379–409.

66. Fröbisch J. Composition and similarity of global anomodont-bearing tetrapod faunas. Earth-Sci Rev. 2009 Aug;95(3–4):119–57.

67. Camp J, Liu J. The taxonomy and cranial morphology of Chinese *Lystrosaurus*. J Vertebr Paleontol. 2011;31(3).

68. Camp J. Morphological variation and disparity in *Lystrosaurus*. University of Iowa; 2010.

69. Rohlf FJ. tpsDig. Stony Brook, NY: Department of Ecology and Evolution, State University of New York at Stony Brook; 2010.

70. Kammerer CF, Deutsch M, Lungmus JK, Angielczyk KD. Effects of taphonomic deformation on geometric morphometric analysis of fossils: a study using the dicynodont *Diictodon feliceps* (Therapsida, Anomodontia). PeerJ. 2020 Oct 7;8:e9925.

71. Adams D, Collyer M, Kaliontzopoulou A, Sherratt E. Geomorph: Software for geometric morphometric analyses. 2016.

72. Adams DC, Collyer ML. Multivariate Phylogenetic Comparative Methods: Evaluations, Comparisons, and Recommendations. Syst Biol. 2018 Jan 1;67(1):14–31.

73. Schlager S. Morpho and Rvcg – Shape Analysis in R: R-Packages for Geometric Morphometrics, Shape Analysis and Surface Manipulations. In: Zheng G, Li S, Székely G, editors. Statistical Shape and Deformation Analysis [Internet]. Academic Press; 2017. p. 217–56. Available from: https://www.sciencedirect.com/science/article/pii/B9780128104934000110

74. Yang W, Wan M, Crowley JL, Wang J, Luo X, Tabor NJ, et al. Paleoenvironmental and paleoclimatic and cyclo- and chrono-stratigraphy of upper Permian–Lower Triassic fluvio-lacustrine deposits in Bogda Mountains, NW China – implications for diachronous plant evolution across Permian-Triassic boundary. Earth-Sci Rev. Forthcoming.

75. Yang W, Feng Q, Liu Y, Tabor N, Miggins D, Crowley JL, et al. Depositional environments and cyclo- and chronostratigraphy of uppermost Carboniferous–Lower Triassic fluvial–lacustrine deposits, southern Bogda Mountains, NW China — A terrestrial paleoclimatic record of mid-latitude NE Pangea. Glob Planet Change. 2010 Aug;73(1–2):15–113.

76. Lamm E-T. 4. Preparation and Sectioning of Specimens [Internet]. Bone Histology of Fossil Tetrapods. University of California Press; 2013. Available from: https://www.degruyter.com/document/doi/10.1525/9780520955110-006/html

77. Holliday CM, Ridgely RC, Sedlmayr JC, Witmer LM. Cartilaginous Epiphyses in Extant Archosaurs and Their Implications for Reconstructing Limb Function in Dinosaurs. Farke AA, editor. PLoS ONE. 2010 Sep 30;5(9):e13120.

78. Griffin CT, Stefanic CM, Lessner EJ, Riegler M, Formoso K, Koeller K, et al. Assessing ontogenetic maturity in extinct saurian reptiles. Biol Rev. 2020;56.

79. de Ricqlès A. Recherches paleohistologiques sur les os longs des tetrapodes; II, Quelques observations sur la structure des os longs des theriodontes. Ann Pale´ontologie. 1969;55(1):1–51.

80. Ray S, Botha J, Chinsamy A. Bone histology and growth patterns of some nonmammalian therapsids. J Vertebr Paleontol. 2004 Sep 10;24(3):634–48.

81. Ray S, Bandyopadhyay S, Bhawal D. Growth patterns as deduced from bone microstructure of some selected neotherapsids with special emphasis on dicynodonts: Phylogenetic implications. Palaeoworld. 2009 Mar;18(1):53–66.

82. Ray S, Botha-Brink J. Dicynodont Growth Dynamics and Lifestyle Adaptations. In: Forerunners of Mammals. Indiana University Press; 2012. p. 29.

83. Olivier C, Houssaye A, Jalil N-E, Cubo J. First palaeohistological inference of resting metabolic rate in an extinct synapsid, *Moghreberia nmachouensis* (Therapsida: Anomodontia). Biol J Linn Soc. 2017 Jun 1;121(2):409–19.

84. Marotti G. Static and dynamic osteogenesis. 2010;4.

85. Stein K, Prondvai E. Rethinking the nature of fibrolamellar bone: an integrative biological revision of sauropod plexiform bone formation. Biol Rev. 2014;89(1):24–47.

86. Ferretti M, Palumbo C, Contri M, Marotti G. Static and dynamic osteogenesis: two different types of bone formation. Anat Embryol (Berl). 2002 Dec 1;206(1–2):21–9.

87. Shapiro F, Wu J. Woven bone overview: structural classification based on its integral role in developmental, repair and pathological bone formation throughout vertebrate groups. Eur Cell Mater. 2019 Oct 1;38:137–67.

88. de Ricqlès A. Evolution of Endothermy; Histological Evidence. Evol Theory. 1974;1(2):51–80.

89. Prondvai E, Stein KHW, de Ricqlès A, Cubo J. Development-based revision of bone tissue classification: the importance of semantics for science. Biol J Linn Soc. 2014 Aug 1;112(4):799–816.

90. Mori R, Kodaka T, Sano T, Yamagishi N, Asari M, Naito Y. Comparative Histology of the Laminar Bone between Young Calves and Foals. Cells Tissues Organs. 2003;175(1):43–50.

91. Currey JD. Differences in the Blood-Supply of Bone of Different Histological Types. J Cell Sci. 1960 Sep 1;s3-101(55):351–70.

92. Currey JD. The many adaptations of bone. J Biomech. 2003 Oct;36(10):1487–95.

93. de Margerie E, Cubo J, Castanet J. Bone typology and growth rate: testing and quantifying ‘Amprino’s rule’ in the mallard (*Anas platyrhynchos*). C R Biol. 2002 Mar 1;325(3):221–30.

94. Urbanek A. Biotic crises in the history of Upper Silurian graptoloids: A Palaeobiological model. Hist Biol. 1993 Jul;7(1):29–50.

95. Harries PJ, Knorr PO. What does the ‘Lilliput Effect’ mean? Palaeogeogr Palaeoclimatol Palaeoecol. 2009 Dec;284(1–2):4–10.

96. Huttenlocker AK. Body Size Reductions in Nonmammalian Eutheriodont Therapsids (Synapsida) during the End-Permian Mass Extinction. PLOS ONE. 2014 Feb 3;9(2):e87553.

97. Huttenlocker AK, Botha-Brink J. Body size and growth patterns in the therocephalian *Moschorhinus kitchingi* (Therapsida: Eutheriodontia) before and after the end-Permian extinction in South Africa. Paleobiology. 2013;39(2):253–77.

98. Grine FE, Forster CA, Cluver MA, Georgi JA. Cranial Variability, Ontogeny, and Taxonomy of *Lystrosaurus* from the Karoo Basin of South Africa. In: Amniote Paleobiology. University of Chicago Press; 2006. p. 432–506.

99. Botha J, Smith RMH. *Lystrosaurus* species composition across the Permo–Triassic boundary in the Karoo Basin of South Africa. Lethaia. 2007;40(2):125–37.

100. Smith RMH, Botha-Brink J. Anatomy of a mass extinction: Sedimentological and taphonomic evidence for drought-induced die-offs at the Permo-Triassic boundary in the main Karoo Basin, South Africa. Palaeogeogr Palaeoclimatol Palaeoecol. 2014 Feb;396:99–118.

101. Botha J, Huttenlocker AK, Smith RMH, Prevec R, Viglietti P, Modesto SP. New geochemical and palaeontological data from the Permian-Triassic boundary in the South African Karoo Basin test the synchronicity of terrestrial and marine extinctions. Palaeogeogr Palaeoclimatol Palaeoecol. 2020 Feb;540:109467.

102. Kitching JW. The distribution of the Karroo vertebrate fauna: with special reference to certain genera and the bearing of this distribution on the zoning of the Beaufort Beds. University of the Witwatersrand; 1977. 1–131 p.

103. Kammerer CF, Viglietti PA, Hancox PJ, Butler RJ, Choiniere JN. A new kannemeyeriiform dicynodont (*Ufudocyclops mukanelai*, gen. et sp. nov.) from Subzone C of the *Cynognathus* Assemblage Zone, Triassic of South Africa, with implications for biostratigraphic correlation with other African Triassic faunas. J Vertebr Paleontol. 2019;39(2):e1596921.

104. Scotese CR, Wright N. PALEOMAP paleodigital elevation models (PaleoDEMS) for the Phanerozoic [Internet]. 2018. Available from: https://www.earthbyte.org/paleodem-resource- scotese-and-wright-2018/

105. Scotese CR. An Atlas of Phanerozoic Paleogeographic Maps: The Seas Come In and the Seas Go Out. Annu Rev Earth Planet Sci. 2021 May 30;49(1):annurev-earth-081320-064052.

106. Kidder DL, Worsley TR. Causes and consequences of extreme Permo-Triassic warming to globally equable climate and relation to the Permo-Triassic extinction and recovery. Palaeogeogr Palaeoclimatol Palaeoecol. 2004 Feb;203(3–4):207–37.

107. Kiehl JT, Shields CA. Climate simulation of the latest Permian: Implications for mass extinction. Geology. 2005;33(9):757.

108. Winguth AME, Shields CA, Winguth C. Transition into a Hothouse World at the Permian–Triassic boundary—A model study. Palaeogeogr Palaeoclimatol Palaeoecol. 2015 Dec 15;440:316–27.

109. Rey K, Amiot R, Fourel F, Abdala F, Fluteau F, Jalil N-E, et al. Oxygen isotopes suggest elevated thermometabolism within multiple Permo-Triassic therapsid clades. Elife. 2017;6:e28589.

110. Winguth A, Winguth C. Precession-driven monsoon variability at the Permian–Triassic boundary — Implications for anoxia and the mass extinction. Glob Planet Change. 2013 Jun 1;105:160–70.

111. Tabor NJ, Sidor CA, Smith RMH, Nesbitt SJ, Angielczyk KD. Paleosols of the Permian-Triassic: proxies for rainfall, climate change and major changes in terrestrial tetrapod diversity. J Vertebr Paleontol. 2017 Nov 29;37(sup1):240–53.

112. Sun Y, Joachimski MM, Wignall PB, Yan C, Chen Y, Jiang H, et al. Lethally Hot Temperatures During the Early Triassic Greenhouse. Science. 2012 Oct 19;338(6105):366–70.

113. Frank TD, Fielding CR, Winguth AME, Savatic K, Tevyaw A, Winguth C, et al. Pace, magnitude, and nature of terrestrial climate change through the end-Permian extinction in southeastern Gondwana. Geology [Internet]. 2021 May 19; Available from: https://doi.org/10.1130/G48795.1

114. Smith RMH. Changing fluvial environments across the Permian-Triassic boundary in the Karoo Basin, South Africa and possible causes of tetrapod extinctions. Palaeogeogr Palaeoclimatol Palaeoecol. 1995 Aug;117(1–2):81–104.

115. Ward PD. Altered River Morphology in South Africa Related to the Permian-Triassic Extinction. Science. 2000 Sep 8;289(5485):1740–3.

116. Viglietti PA, Smith RMH, Rubidge BS. Changing palaeoenvironments and tetrapod populations in the *Daptocephalus* Assemblage Zone (Karoo Basin, South Africa) indicate early onset of the Permo-Triassic mass extinction. J Afr Earth Sci. 2018 Feb;138:102–11.

117. Gastaldo RA, Kamo SL, Neveling J, Geissman JW, Looy CV, Martini AM. The base of the *Lystrosaurus* Assemblage Zone, Karoo Basin, predates the end-Permian marine extinction. Nat Commun [Internet]. 2020;11(1). Available from: http://www.nature.com/articles/s41467-020-15243-7

118. Roopnarine PD, Angielczyk KD. Community stability and selective extinction during the Permian-Triassic mass extinction. Science. 2015 Oct 2;350(6256):90–3.

119. Metcalfe I, Foster CB, Afonin SA, Nicoll RS, Mundil R, Xiaofeng W, et al. Stratigraphy, biostratigraphy and C-isotopes of the Permian–Triassic non-marine sequence at Dalongkou and Lucaogou, Xinjiang Province, China. J Asian Earth Sci. 2009 Nov;36(6):503–20.

120. Wan M, Yang W, Wang J. *Amyelon bogdense* sp. nov., a silicified gymnospermous root from the Changhsingian–Induan (?) in southern Bogda Mountains, northwestern China. Rev Palaeobot Palynol. 2019 Apr;263:12–27.

121. Wan M-L, Yang W, Wan S, Wang J. Wildfires in the Early Triassic of northeastern Pangaea: Evidence from fossil charcoal in the Bogda Mountains, northwestern China. Palaeoworld. 2021 Jul;S1871174X21000639.

Formatted: Line spacing: Double

Dear Editors and Reviewers,

Thank you for your reviews and feedback. We have revised a large portion of the manuscript in response to Reviewer 1's comments and concerns and have included addition stratigraphic information and relevant discussion in response to Reviewer 2. Please find a detailed list of our changes to the text, figures, and supplemental information below.

Kind regards,

Zoe Kulik

Comments from the Journal

1. Please ensure that your manuscript meets PLOS ONE's style requirements, including those for file naming. The PLOS ONE style templates can be found at https://journals.plos.org/plosone/s/file?id=wjVg/PLOSOne_formatting_sample_main_body.pdf and https://journals.plos.org/plosone/s/file?id=ba62/PLOSOne_formatting_sample_title_authors_affiliations.pdf

Done

2. We note that you have stated that you will provide repository information for your data at acceptance. Should your manuscript be accepted for publication, we will hold it until you provide the relevant accession numbers or DOIs necessary to access your data. If you wish to make changes to your Data Availability statement, please describe these changes in your cover letter and we will update your Data Availability statement to reflect the information you provide.

We have unchecked the box that states URLs will be available after publication. All of the relevant data including accession numbers and one URL (that directs to supplemental images hosted on Morphobank) are within the manuscript and its supplemental files. We have no further data to make available and do not need to make a change to our Data Availability statement.

3. Please include captions for your Supporting Information files at the end of your manuscript, and update any in-text citations to match accordingly. Please see our Supporting Information guidelines for more information: http://journals.plos.org/plosone/s/supporting-information.

Done

Comments from Reviewer 1:

Reviewer #1: This paper focuses on the osteohistology of Lystrosaurus from the Jiucaiyuan Formation of northwestern China and thus, gives a (relatively) new perspective on Lystrosaurus life history from a northern hemisphere perspective. Lystrosaurus is an ideal study taxon for examining life history changes during mass extinction events as it survived the end-Permian mass extinction to become the most abundant vertebrate in the post-extinction ecosystem. Thus, any new data on Lystrosaurus is globally relevant. This paper is very similar to a recently published paper on Chinese Lystrosaurus osteohistology by Han et al. 2020 even finding very similar conclusions, so the value in this research is not necessarily novel data, but increasing the sample size of Chinese Lystrosaurus to support Han et al.'s 2020 findings. The authors do, however, dispute some of the details of Han et al.'s 2020 paper (which I partially agree with - they may have misidentified a few growth marks, especially in the youngest individuals, but they are correct for the most part) and the authors in this paper provide a very good dataset on the basal skull length of Lystrosaurus skulls from China.

The paper is very well written and detailed and although very similar to the Han et al. 2020 paper, it does increase the sample size of Chinese Lystrosaurus and so should be published once the osteohistological details have been sorted out. There are some issues with identifications and descriptions that need to be fixed or clarified before the paper can be ready for publication.

I have provided numerous comments on the pdf itself, but there are some issues that need to be explained here and corrected in the manuscript.

1. Despite Prondvai et al. 2014 updating bone tissue terminology very few paleohistologists accepted the change in terminology (including myself) until the recent publication of a new osteohistology textbook by Buffrenil et al. 2021. This book finally accepts that the osteohistology terminology should be updated and more correct definitions be attributed to bone tissue types from a developmental perspective. This gives more meaning to the descriptions. The book is very new and only became available during the submission of this paper so it is unlikely the authors have seen it or even have access to it. They are welcome to contact me as I have a pdf copy that I can send them, but this and the Prondvai reference really should be used to describe the bone tissues. I'm not judging, I've been remiss myself, but we need to start using the correct terminology now and this will correct or update both the Botha 2020 paper and the Han et al. 2020 paper on what Lystrosaurus bone histology really looks like. I'm sending you a table of definitions (see attachment) that will help you to identify the bone tissue types in the various ontogenetic stages. I can see certain aspects of the extracellular matrix in the high magnification images but neither the figures nor the

images on Morphobank are high enough resolution for me to confirm static or dynamic osteogenesis – and you need this to decide what bone tissue type you are looking at. The term fibrolamellar bone is no longer used. Instead bone tissue types have been replaced with woven bone, parallel-fibered bone and the woven-parallel complex. Typically only embryonic or neonates have pure woven bone, which is SO (static osteogenesis) derived. Thus, what you are looking at in the Lystrosaurus bone tissues is the woven-parallel complex, which consists of both SO and DO (dynamic osteogenesis) derived bone tissues. This is where the PFB (parallel-fibered bone) as the lamellae around the primary osteons and within the extracellular matrix is deposited on a scaffold of woven bone. The term fibrolamellar complex (FLC) has been retained to describe tissues that are predominantly woven, but have the PFB component in the form of primary osteons. This is highly vascularized fast growing tissue that you will only see in young juveniles, but falls under the woven-parallel complex. Thus you can still use FLC if you explain what you mean, but from the images I am unsure if you do have FLC in any of your age classes. You may have had it in Age Class I (but you don’t have this), but I am unable to see enough of the osteocytes to see if you have predominantly SO or an equal amount of SO-DO in your Age Class II. In order to prove FLC you will need a very high magnification image of woven bone and it’s possible it’s present in the inner cortex of the bones in Figure 3, but unfortunately Fig 3B and C do not provide enough of the cortex to know if it’s FLC or WPC. These two images show WPC, for example in Fig 3C just under the letter C there is a nodule of woven bone, but unless I saw more of the cortex at this magnification, I cannot tell from your images if you have FLC or WPC. To prove FLC you need to replace Fig 3C with something showing more woven bone. I realise you had a point to 3C, so it might be more useful to replace Fig 3B with tissues showing a higher degree of SO. This follows through all of your age classes – you are not likely to find FLC in your older individuals, even though they are still far from being fully grown, it is likely that you have WPC as the dominant bone tissue type. This is the case for the South African and Indian Lystrosaurus. If it is difficult to redo some of the figures you could add several images to your Morphobank data – high magnification images. The reason why it’s important to get this description right is because, for example, you mention parallel-fibered bone in one of your elements – for an overall change from WPC to PFB, which has not been found in any Lystrosaurus specimens studied to date, would imply that you’ve found an individual that is showing the possible onset of reproductive maturity, or at least that it’s a late subadult, which is older than any Lystrosaurus specimen found to date. I do not see your parallel-fibered bone in the ulna, it still looks like WPC but with a much lower degree of the woven component, i.e. there is still static osteogenesis. The vascular canals in Figure 5M still looks like WPC and not PFB. Thus, if you’re right it has far reaching implications, but you have not shown enough evidence for a change from WPC to PFB. Another reason why it’s important to be absolutely sure you’re dealing with FLC (a subcategory of WPC) is that it indicates very high growth rates compared to WPC (although WPC still indicates high growth rates, depending on the proportion of SO). So if you still have FLC in your older age classes it’s suggesting that these animals were growing faster than Lystrosaurus in other parts of the world or were younger – so again it’s important to differentiate.

Prondvai, E., K. H. Stein, A. de Ricqles and J. Cubo. 2014. Development-based revision of bone tissue classification: the importance of semantics for science. Biological Journal of the Linnean Society 112, 799–816.

Buffrénil, de V., A. de Ricqlès, L. Zylberberg and K. Padian (eds.), Comparative skeletal histology and paleohistology, CRC Press, Taylor and Francis, Boca Raton, 824 pp. (the chapter by Buffrenil and Quilhac on bone tissue types).

I think the paper would benefit from some polarized light images showing the woven look.

2. A very big issue are the vascular orientations. To clear something up that I see in a couple of places in the text, the phrase – laminar to fibrolamellar bone – is incorrect. Laminar bone is a kind of vascular orientation found within the fibrolamellar complex (previously known as FLB), so it IS FLC. I have seen descriptions written like this in papers before, but it is incorrect. The old Francillon et al 1990 book of definitions or the latest osteohistology book by Buffrenil et al 2021 gives you all the definitions of various vascular orientations within the woven-parallel complex. According to the images shown in this paper the younger individuals contain predominantly longitudinally-oriented primary osteons with perhaps some short anastomoses (not enough to be reticular), and as the individuals become older a predominance of plexiform or laminar bone appears. This is important because – although there is an overlap – the rule of thumb is that longitudinal canals indicate faster growth rates than laminar bone – and this would make sense as the older individuals become more laminar. There is also the hypothesis – based on extant studies – that laminar bone is stronger, which you would expect in the older individuals. Most of the vascular orientations for each bone are incorrect and this needs to be corrected. I won’t give you definitions to all the vascular orientations here.

3. The first definition of a growth mark is described as a LAG and annuli are not mentioned until they are described in the description part of the paper, with no definition. Growth marks include both annuli and LAGs (and even changes in vascular orientation), both annuli and LAGs need to be defined when they are first introduced as the bones contain both annuli and LAGs.

4. Lastly I would not suggest you identify the peripheral regions as an EFS in the places mentioned - you can mention it as a possibility but, particularly if you are basing this on ribs – or at least say it is just as likely (more than likely really) that you’re seeing a temporary decrease or cessation in growth. As one is generally dealing with a rib fragment, one doesn’t even know from where along the rib the section is taken, so the histology can change along the bone. You might tend to find more growth marks in ribs, but you really do not know if you’re dealing with an EFS or not – and you make this case in your discussion – that you haven’t found any senescent individuals yet. So I would suggest that you represent these regions as temporary changes in growth, but

say that it's not impossible they represent an EFS (so change it around, because it is highly likely you are not looking at an EFS).

We have read the suggested publications and chapters from the recent textbook and have added a Bone Histology Terminology section to the Materials and Methods section to add additional information relevant to the recent changes in terminology. We have also updated the histological descriptions throughout and added high magnification images to Figs 3, 5, and 6 to address the majority of Reviewer 1's questions and comments. Details of our changes are explained below.

Line 79: annuli also represent annual changes in growth and you need to include them here too, even if your samples only show LAGs, the SA samples also have annuli, so you need to explain to the reader that you are talking about growth marks (which includes LAGs and annuli)

The Osteohistological Perspectives on Life History section has been expanded to include descriptions and definitions for both annuli and LAGs.

Line 92: you need to refer to growth marks (general term) here - not just LAGs, as it's the scarcity of annuli AND LAGs. Here you are only referring to one type of growth mark, annuli are still deemed annual

Corrected for clarity

Table 1 Column 5: this is the first time you mention growth marks - you haven't defined this in the text. This is why you need to define growth marks - not just LAGs when you first mention them

Updated definitions in Osteohistological Perspectives on Life History section

Line 323: how did you measure this? This is important for comparisons as you will notice that Fig 3F, all in Fig 4, Fig 5 and fig 6 have a gradual transition between medullary cavity and compact cortex. One needs to be consistent in defining where the edge of the medullary cavity is, which is not possible in Lystrosaurus (juding it by eye). You need to

explain exactly how you measured these sections - from open medullary cavity, from where there was 50% more bone than cavity space, from where you deemed the cortex to be compact (which can be subjective). You need to show a diagram, at least, if you going to do it by eye. Otherwise, in order to do it properly you need to use ImageJ or Bone Profiler to get K and thus the cortical thickness

Based on your recommendation, we have replaced the bone wall thickness measurements with cortical thickness (K). This resulted in some updated measurements and corrections of clerical errors to Table 1. We attempted to use BoneJ to determine the relative porosity in the gradual transition of trabecular medullary infilling to cortical bone but dark minerals that infilled the medullary and vascular spaces resulted in unreliable values. We proceeded to use a visual estimation of the transition to compact cortical bone and include two supplemental figures that illustrate where our measurements were taken (S1 Supplementary Information SF5 and SF6).

Line 326: I will give you a detailed explanation in my overall comments as to why I've changed the details here to reflect more up to date terminology

Thank you for these detailed comments.

Line 326: you're mixing up vascular canal orientation - laminar is a type of vascular orientation, you can have laminar or plexiform bone but you cannot have laminar bone with a plexiform orientation

Edited for clarity

Line 330: this is why you need to define growth marks earlier - this is the first time you mention annuli and you don't define what they are

Updated to include both, see above

Line 357: you need to prove that the extracellcular matrix has a woven component. Looks for areas between the osteons - outside of the PFB lamellae that have bunched, large rounded osteocyte lacunae. They will look like nodules or bunches. You need to show a

high magnification image of these osteocytes to prove this is not PFB. you could use 3C and point to the area just below the C - otherwise in B at the lower left corner there is some woven bone, otherwise you can take a new image

Added text labels to Fig 3B and replaced one of the images with a high-magnification image illustrating woven-fibered bone in an associated fibula (Fig 3E).

Line 361: rephrase - an annulus is not a growth arrest, but a growth decrease, you can say growth cycles

Rephrased

Line 363: none of these elements are showing laminar or plexiform tissue (you see it in the older individuals but not here), here the dominant vascularization is longitudinally-oriented primary osteons in circumferential rows, with some short anastomoses - but they're not laminar or plexiform - redo the vascularization here

Done

Line 365: you need to say this is the yellow square in A.

Done

Line 365: be careful here - your arrows aren't actually pointing to woven bone. I understand what you're meaning here and you are correct, but the arrows are just pointing to areas where the osteocyte lacunae are not preserved - and may be woven or parallel-fibred. In 3C there is a woven patch just under the letter C in the image - but your arrows are pointing to something we do not know. A woven-parallel complex has both woven and parallel components

Edited figure caption and accompanying text. We refer to these lines as bright lines in the text and have updated the figure caption for clarity.

Line 369: no same as D - this is not plexiform

This image has been replaced to show a high magnification image of woven-fibered bone matrix.

Line 381: no - see my comments in your Figure 3 caption. Yes you are seeing fast growth but this is a woven-fibered complex, and there is no plexiform bone here

Edited

Line 385: This band seems to run around most of the cortex and is likely a temporary decrease in growth rate (and you need to introduce the possibility that it could be annual). Growth marks needn't be narrow annuli of PFB of LB, but they are can also be represented by changes in vascular canal orientation or a temporary decrease in vascular size - this has to mean a temporary decrease in growth rate

With respect to the smaller vascular canals in the proximal tibia, we have edited the text to refer to this temporary decrease in canal sizes as a temporary shift in growth rate. We do not interpret the differences in vascular canal size or orientations as temporary decreases in growth rate in various quadrants of the fibula and maintain that the different tissue textures seen in Fig 3A are not indicative of shifting bone growth rates in annual or cyclical patterns.

Line 388: and what is the woven versus parallel-fibered component - is there more PFB in the organized region and more woven bone in the less organized region, or does the proportion remain the same and it's only the vascular canal orientation that changes? I can't see from the Figure

Edited to add a description of the proportion of woven-fibered bone in this element.

Line 393: you need to add here why they shouldn't be mistaken for annuli either - i.e. if you don't see a cement line, why shouldn't you call them annuli? so you need to point out that these bright lines do not contain bands of slower forming tissues like parallel-fibered or lamellar bone.

Good point. We have added more to the description.

Line 393: not reticular - you need to check your definitions, I'll explain in the overall comments - Your Stage II mostly comprises longitudinally-oriented primary osteons in circumferential rows - Fig 3A, 3F. Definitely not enough anastomoses to call this reticular and the canals are not circumferentially arranged (for laminar or plexiform). Longitudinal canals arranged circumferentially is different to canals arranged circumferentially around the bone

'Reticular' and 'plexiform' have been replaced with 'longitudinally-oriented primary osteons' throughout.

Line 407: no - laminar is a kind of vascularization that forms part of the traditional fibrolamellar complex. You cannot have laminar to fibrolamellar bone - this is comparing apples to oranges. The vascularization is not truely laminar yet like you get in Figure 5J. The canals are a mixture of sub-laminar and longitudinally-oriented primary osteons in circumferential rows

Edited for clarity. It is our preference to refer to this tissue as a mixture of longitudinal and circular canals that approaches a laminar organization.

Line 408: what bone tissue makes up the annulus? PFB or LB?

Edited to add: parallel-fibered. (None of the osteocyte lacunae are lenticular-shaped so it seems these lacunae were cut orthogonally)

Line 414: hind limb

Done

Line 414: check these orientations - you haven't been accurate about the previous descriptions and the resolution of the renders are not high enough for me to see clearly what's happening in E and H - but F and G look sub-reticular not plexiform . There are longitudinal canals at the subperiosteal surface of F and I and L

We have updated the vascular orientation to reflect differences in each of the 3 elements, rather than try to combine the differences in one sentence.

Line 420: then it's probably the PFB part of the WPC where woven bone is becoming discontinuous

Edited to replace lamellar with parallel-fibered bone.

Line 425: This definitely looks like a LAG in the inner yellow bracket - how far does it go? I cannot see if the lines are cement lines or lamellae - but either way if they go most of the way around the cortex they are likely growth marks and should be pointed out in this image

Updated figure caption and accompanying text. The thickness of the avascular PFB region is pretty variable and the subperiosteal edge is not complete around the entire section, but the parallel-fibered tissue goes most of the way around the cortex and is certainly a growth mark.

Line 427: it's a double LAG - there's no bone tissue between them and they likely represent one season

Agreed.

Line 429: The image isn't quite high resolution enough - but it looks like what you're seeing is the PFB component of the WPC increasing towards the outer cortex. As these are older individuals you should see a progressive decrease in the amount of woven bone and an increase in PFB towards the outer cortex. If that outer region in K goes around the whole cortex then it's likely a growth mark (annulus)

Yes, there is a shift towards more organized parallel-fibered bone in some regions of the outermost cortex, but it is likely not an annulus. In the vascularized regions (4L), the mineralized lines wrap around primary osteons rather than cross-cutting, so we are interpreting these lines as the mineralizing front of periosteally deposited bone.

Line 450: you need a higher magnification of Fig 5I showing the "multiple stacked LAGs". The current image shows a cycle from too low magnification so one cannot see that there are LAGs - from this it looks like an annulus. If you zoom in on your yellow bracket more you can show them properly

Edited to refer to growth mark as an annulus.

Line 454: no take this out - you've just said in the next sentence what it most likely represents. These animals are no way near fully grown

Done

Line 456: exactly

Thank you

Line 472: now this is proper laminar vascularization - do you see how the canals are more connected to one another circumferentially compared to the younger elements where the vascular canals are more longitudinal with less connections?

Agreed

Line 480: are you absolutely sure there isn't one closer to the medullary cavity? You don't show a close up so I can't tell from the overall render, it's either just a change in colour or it could be an annulus - just double check

It is just a color change.

Line 482: laminar bone IS fibrolamellar bone. Yes it's laminar but that refers to the vascularization. You would call a woven-parallel complex with laminar vascularization. I'll explain in my overall review

Edited

Line 483: no - they are laminar, just like you said

Edited

Line 492: Are you absolutely sure you are only seeing dynamic osteogenesis and there is no static osteogenesis? You need to be really sure about this before you call it PFB - this has huge implications, because none of your other elements show this and neither do the South African samples, so if you're right you seeing the beginning of what is probably reproductive maturity - but you have to be absolutely sure - this is very important

We have updated the figures to show a region in the outer cortex that reflects the overall tissue type that is largely parallel-fibered but has small areas of woven-fibered bone in the ulna. The osteocyte lacunae are less densely packed than in the hind limb elements but do show areas of static osteogenesis.

Line 496: no but if you're seeing an overall change from the woven-parallel complex to parallel-fibered bone then you could be seeing reproductive maturity. But the amount of woven bone decrease dramatically with age, but if there are still patches of it, then it's not PFB like you've said above. I'm afraid I cannot see the osteocytes well enough in M to help you here, and you'd need to check around the whole of the outer cortex

The change is not dramatic enough to suggest maturity, rather, there is just less woven-fibered bone

Line 501: This bone shows continued rapid growth but is bigger and presumably older than the ulna in Size Class III - which is why I'm hesitant to call that PFB in the ulna. You need to check this as it has important repercussions. If you're right then it suggests a decoupling of size and reproductive maturity. It is more likely that the ulna is woven-parallel all the way to the edge

Edited above (see line 492)

Line 503: OK - so this makes no sense. The inner cortex represents younger ontogeny and shouldn't have been growing slower than the outer cortex which represents the older

ontogeny. This all looks like woven-parallel complex, where you'll have varying degrees of woven versus parallel-fibred bone.

The birefringence under cross-polarized light was really difficult to diagnose. After looking more closely at the osteocyte lacunae, we see woven- and parallel-fibered bone throughout the middle and outer cortex. There seems to be more parallel-fibered bone in the outer cortex compared to the deep and middle cortex, but not an overall shift from one tissue type to another.

Line 511: no it doesn't - and if it's laminar vascularization this is not going to be PFB but rather WPC

Corrected, see above

Line 515: you don't know this - as you've seen, a thick slower growing region at the sub-periosteal surface is likely a seasonal annulus. Yes it's not impossible it's an EFS, but if you nor Han nor myself have ever found fully grown individuals of Lystrosaurus - what is more likely? That this individual represents a fully grown animal, or that it represents a temporary decrease in growth rate? You need to put both possibilities out there and the latter is far more likely

We have removed references to an EFS and have added to the description to explain the uncertainty raised by your comments.

Line 517: same comment - you don't know this is an EFS

We have removed references to an EFS.

Line 526: I don't see a stack of LAGs - I see a region of PFB that may or may not represent an EFS - you really need to rather think of the more likely possibility (as explained above) that these bones do not represent fully grown individuals. The only way we will find definitive evidence of fully grown Lystrosaurus is if we find it in the limb bones. I really

would not base this on the ribs, as I've seen many ribs like this where the associated limb bones show that the animal is still growing

Perhaps the emphasis was lost here. We agree that ribs alone, and especially fragmentary ones, do not indicate whether an animal is fully grown since ribs grow at different rates than limbs. We have edited the description to explain the uncertainty raised by your comment.

Line 534: I disagree with your vascular orientations - see earlier comments

Edited throughout

Line 537: I would call this more laminar

Edited

Line 558: no - an annulus cannot consist of stacked LAGs. I was wondering what you were meaning by stacked LAGs in the description. There's no high enough magnification for me to confirm - but what I think youré actually seeing is an annulus consisting of lamellar tissue - and the "stacked LAGs"are the individual lamellae. You can have an annulus and a LAG together - where the annulus occurs before or after the LAG - but you cannot have an annulus consisting of multiple LAGs. If you are indeed seeing LAGs and not lamellae, you need to show a high magnification image of this, so far I haven't seen any evidence of this

Edited throughout

Line 558: avascular what? tissue? lamellar tissue? parallel-fibered tissue? It can be avascular but what tissue type is it?

Edited to add: parallel-fibered to lamellar tissue

Line 568: you need to cite Buffrenil et al 2021 for this - they give a detailed account (first given by de Ricqles) regarding the "unit". Woven bone is deposited first as a scaffold on which parallel-fibered bone (in the form of primary osteons) is deposited. So it's not really that bone

is deposited first and then woven bone is deposited - it's more the other way around, this is why you need this book. You can also look at Prondvai et al 2014

The type of bone deposition we are referring to here is the accretion of periosteal bone, rather than the formation of primary osteons. Currey (1960) and others (e.g., Mori et al., Cells Tissues Organs. 2003) describe laminar bone formation where each subsequent circumferential layer of bone (made up of a woven scaffold that is later infilled with PFB or LB) is bordered by the periosteum. As the periosteum jumps peripherally when new bone is accreted, a bright line is left between each circumferential layer and represents the place at which the periosteum was located before new bone was deposited.

601: I'm not sure I understand your meaning of maximum sizes here. We don't know what the maximum size of any of these species was, so how can one say they could grow to similar MAXIMUM sizes? Please clarify

Edited to add: maximum recorded size

Line 656: no it doesn't - unless you can provide evidence that you get plants/trees that take multiple years to reach maturity and do not produce growth rings? Otherwise isn't this more likely that the wood fossils represent immature plants that are less than a year old. If you are correct please provide references that show that trees can grow multiple years without laying down growth rings.

We have removed this statement based on updated reports of fossil wood with growth marks and charcoal from the study area.
Wan M-L, Yang W, Wan S, Wang J. Wildfires in the Early Triassic of northeastern Pangaea: Evidence from fossil charcoal in the Bogda Mountains, northwestern China. Palaeoworld. 2021 Jul

Line 668: agreed

No change

Line 711: italix

Done

Comments from Reviewer 2:

IVPP V 27127=19SS26 This is a specimen from Shanshan, not Turfan!
It is the only one from the upper portion of the Jiucaiyuan Formation. It should be caution to discuss it. Because it is so different from others!
Also, could IVPP V 26548 represent an adult?
Do you have the original photo of 19SS26, I check the preserved part is less than 20 cm, not 240mm (L241)?

We have updated stratigraphic information for V 27127. It does come from higher in the section which we have added to the discussion section as a possible explanation for why the bone tissue is different from some of the smaller specimens. We have an original photo with a scale bar from the field for 19SS26 and, while incomplete, it is over 25cm long. For specimen IVPP V 26548, we do not see evidence of somatic maturity (i.e., EFS) which makes it extremely unlikely that this individual was an adult.

Abbrev, for IVPP V appears first in Fig 1, anterior to L172

We don't believe we need an explanation of the abbreviation in this figure based on the explanation currently in the text.

L182 Chang the format as IVPP V 27126.1 not – (a blank after V)
Table 1 For same specimen, you need not add letter behind it.

We changed the formatting of the specimen numbers throughout. Our preference is to keep the letter following each element from associations of the same specimen for clarity.

Fig.1 move below the paragraph begin from L 170

Change Size Distribution Figure to FIG 2. Put it after Geometric Morphometric Fig

Our preference is to keep the limb size distribution as Fig 1 since the emphasis of this paper is on the bone histology of that sample that is first introduced in the Introduction section.

L 30 'Formation of northwestern China' to 'Formation of Xinjiang, China'

Done

L62 Lystrosaurus has a low preferred temperature than other tetrapods (Liu et al., submitted)
Liu, J., Angielczyk, K.D. and Abdala, F., 2021. Permo-Triassic tetrapods and their climate implications. Global and Planetary Change.
It is almost accepted now.

This manuscript was still in review at the time we submitted the manuscript. However, a revised version has been resubmitted, and we now cite the paper and note that the unusual inferred thermal tolerances of Lystrosaurus could have contributed to its survival.

L109 northwestern to 'Xinjiang,'

Done

shows uninterrupted cortical growth, suggesting
336 that Lystrosaurus from the Jiucaiyuan Formation had a high intrinsic rate of growth that could
337 periodically arrest.

No change

L702 For Chinese names, you can list like YI Jian, LIU Jun, but all in same sequence

Done, thank you

L711 Lystrosaurus change to italicize

Done

Composite thin section images are available on Morphobank

Click here to download Data Review URL
http://morphobank.org/permalink/?P4023
