# Supplementary material for: Living fast in the Triassic: New data on life history in Lystrosaurus (Therapsida: Dicynodontia) from northeastern Pangea
Source: PLoS One. 2021 Nov 5;16(11):e0259369. doi: 10.1371/journal.pone.0259369 (PMC8570511; doi:10.1371/journal.pone.0259369)
Supplement: Attachment — Submitted filename: Bone tissue definitions.docx [file pone.0259369.s004.docx]

Bone tissue definitions:

Here’s a table to help you identify what bone tissue types you’re dealing with. Unfortunately I cannot help you further unless I see several images of the cortex to identify what is the predominant bone tissue type (and I can’t see this from the images provided).

| Woven Bone (WB) | Derived from static osteogenesis (SO) differentiating cells that surround themselves with a matrix at a place where they are already present  **Collagen fibre orientation:**  Woven-fibred extracellular matrix where the mineralized fibres are largely disorganized with no specific relation to adjacent vascular spaces  Large-scale optical behaviour isotropic (dark) in polarized light (monorefringent)  **Morphology and spatial distribution of osteocyte lacunae:**  Abundant large, globular or rounded osteocytes randomly oriented and irregularly distributed throughout the matrix  Osteocyte canaliculi radiate in all directions  **Vascularization:**  Vascular spaces large, no osteonal development, generally longitudinally-oriented  **Occurrence:**  Primary bone  Usually restricted to embryos or neonates |
| --- | --- |
| Parallel-fibred bone (PFB) | Derived from dynamic osteogenesis (DO) the cells move to position themselves along bone surfaces where they synthesise lamellar and parallel-fibred bone  **Collagen fibre orientation:**  Lamellar or non-lamellar parallel-fibred extracellular matrix where the mineralized fibres are highly organized  Large-scale optical behaviour anisotropic (bright) in polarized light (birefringent) if the collagen fibres are cut along the longitudinal axis of the bone (parallel to collagen fibres), but isotropic (dark) if the fibres are cut transversely  **Morphology and spatial distribution of osteocyte lacunae:**  Acellular or cellular  Relatively low density of spindle-like, elongated or ellipsoid osteocytes oriented parallel to the general direction of the collagen fibres and evenly/regularly spaced throughout the matrix  Osteocyte canaliculi perpendicular to the walls of the lacunae  **Vascularization:**  Avascular, longitudinally-oriented simple canals or primary osteons  **Occurrence:**  Primary or secondary bone, primary trabeculae formed during endochrondral ossification, lamellae in primary and secondary osteons  Various vertebrate groups, usually mid to late ontogeny |
| Woven-Parallel Complex (WPC) | Derived from a combination of static-dynamic osteogenesis (SO-DO)  **Collagen fibre orientation:**  The woven component is the SO-derived woven bone that forms trabeculae or nodules between the vascular canals and the parallel component is the lamellar or non-lamellar parallel-fibred DO-derived bone tissue that is deposited on the woven scaffold centripetally around the vascular canal to form a primary osteon  WB<PFB:  less woven bone than parallel-fibred bone, woven bone patchy, discontinuous, limited to thin lines or nodules between vascular canals (also known as uncommon parallel-fibred bone, Houssaye et al 2013 or coarse parallel-fibred bone, Klein et al 2016)  WB=PFB  roughly equal amounts of woven bone and parallel-fibred bone  WB>PFB  more woven bone than parallel-fibred bone, woven bone is highly abundant between closely spaced, abundant primary osteons (also known as fibrolamellar complex [FLC] or historically fibrolamellar bone)  **Morphology and spatial distribution of osteocyte lacunae:**  Abundant large, globular or rounded osteocytes randomly oriented and irregularly distributed throughout the matrix  Osteocyte canaliculi radiate in all directions  Relatively low density of spindle-like, elongated or ellipsoid osteocytes oriented parallel to the general direction of the collagen fibres and evenly/regularly spaced throughout the matrix  Osteocyte canaliculi perpendicular to the walls of the lacunae  **Vascularization:**  WPC with simple vascular canals  WPC with longitudinally-oriented primary osteons  WPC with longitudinally-oriented primary osteons with short anastomoses  WPC with laminar primary osteons  WPC with plexiform primary osteons  WPC with reticular primary osteons  WPC with radial primary osteons  **Occurrence:**  Primary or secondary bone, cortical or spongy bone, medullary bone, pathologic bone  Various vertebrate groups, may occur in early, mid- or late ontogeny. The amount of woven bone tends to decrease with increasing ontogeny and may rely on other factors e.g. phylogeny |
